# A review of *Coelostegus prothales* Carroll and Baird, 1972 from the Upper Carboniferous of the Czech Republic and the interrelationships of basal eureptiles

**DOI:** 10.1371/journal.pone.0291687

**Published:** 2023-09-21

**Authors:** Jozef Klembara, Marcello Ruta, Jason Anderson, Taran Mayer, Miroslav Hain, Daniel Valaška

**Affiliations:** 1 Department of Ecology, Laboratory of Evolutionary Biology, Faculty of Natural Sciences, Comenius University in Bratislava, Bratislava, Slovakia; 2 Institute of Measurement Science, Slovak Academy of Sciences, Bratislava, Slovakia; 3 Department of Life Sciences, Joseph Banks Laboratories, University of Lincoln, Lincoln, United Kingdom; 4 Faculty of Veterinary Medicine, University of Calgary, Calgary, Alberta, Canada; 5 Department of Biological Sciences, University of Calgary, Calgary, Alberta, Canada; 6 Scanstudio s.r.o., Bratislava, Slovakia; Chinese Academy of Sciences, CHINA

## Abstract

We redescribe the holotype and only known specimen of the early eureptile *Coelostegus prothales* from the Upper Carboniferous of the Czech Republic using photogrammetric scanning and a virtual 3D rendition of its skull. New information is available on several skull and lower jaw bones, including the postorbital, supratemporal, tabular, postparietal, angular, and prearticular. The new data also permit the correct identification of previously undetected or mis-identified elements (e.g., supratemporal; quadratojugal; angular). We provide an amended diagnosis of *Coelostegus* and a new reconstruction of the skull in dorsal and lateral views. To evaluate the affinities of *Coelostegus*, we code this taxon in two recently published taxon-character matrices. Parsimony and Bayesian analyses do not permit firm conclusions on the phylogenetic position of *Coelostegus* or, indeed, the status and extrinsic relationships of protorothyridid amniotes. *Coelostegus* emerges either as the sister taxon to the recently redefined Diapsida (Araeoscelidia; Varanopidae; Parareptilia; Neodiapsida), as one of the most basal protorothyridids, or as a derived stem-group amniote in various parsimony-based analyses, or as the basalmost protorothyridid in one Bayesian analysis, with protorothyridids forming a paraphyletic array relative to Diapsida. We review the cranial similarities and differences between *Coelostegus* and other protorothyridid genera and discuss the implications that various phylogenetic results have for our understanding of early amniote relationships.

## Introduction

Eureptilian amniotes [1] traditionally consist of two families, the Captorhinidae and the Protorothyrididae, and the diverse clade Diapsida, the latter including extant reptiles and birds and all those fossil amniotes that are more closely related to those two groups than they are to extant mammals. The monophyletic captorhinids are a late Carboniferous to late Permian group of small to large-sized tetrapods, several of which are characterized by sturdy limbs, stocky trunks, subtriangular skulls, multiple tooth rows, and downturned snouts [1, 2]. This group is anatomically and ecologically diverse and exhibits adaptations to a variety of diets. Captorhinids have undergone extensive revisions during the last few decades, particularly in conjunction with important new fossil discoveries. The small and superficially lizard-like protorothyridids range from the Pennsylvanian (late Bashkirian–Moscovian) to the Cisuralian (Asselian). This group is of remarkable zoological interest as it includes the stratigraphically oldest and undisputed amniotes [3, 4]. Six of the seven known genera are monotypic (*Anthracodromeus*, *Brouffia*, *Cephalerpeton*, *Coelostegus*, *Hylonomus*, and *Paleothyris*) and one (*Protorothyris*) includes two species [1–5]. Though the status of protorothyridids is uncertain, the prevailing consensus is that they are non-monophyletic [6]. Our knowledge of protorothyridids still relies for the most part upon their original descriptions, most of which are outdated [4]. One of the most elusive and least known protorothyridids, *Coelostegus prothales* from the Middle Pennsylvanian of the Czech Republic, is the subject of the present work.

In their seminal study on the phylogeny of early eureptiles, Müller and Reisz [6] included all known protorothyridid genera. Maximum parsimony and Bayesian inference analyses recovered most of them (except for *Coelostegus*) as a paraphyletic array relative to diapsids (represented by Araeoscelidia in their data matrix), immediately crownward of captorhinids, and with *Coelostegus* as the sister taxon to all remaining eureptiles. The monophyly of eureptiles, however, was weakly supported and the position of *Coelostegus* was unstable. Müller and Reisz attributed this lack of stability to the unusual combination of traits in this taxon. Thus, whereas the elongate squamosals and nasals and the enlarged supratemporals set *Coelostegus* apart from other basal eureptiles, the absence of a contact between the postorbital and supratemporal points to eureptilian affinities. These findings prompted Müller and Reisz to conclude that a re-examination of *Coelostegus* is timely.

The extrinsic relationships of the protorothyridids and the taxonomic composition and status of eureptiles have remained largely unchanged in most studies published since Müller and Reisz’s work. However, some recent analyses have retrieved novel and often irreconcilable branching patterns near the roots of the amniote crown group, challenging the status of several long-established groups and proposing alternative hypotheses of relationship for various clades and grades of primitive amniotes. Ford and Benson [7] introduced Neoreptilia for a clade consisting of Parareptilia plus Neodiapsida (= Diapsida minus Araeoscelidia). Their uncalibrated Bayesian analysis placed Captorhinidae, Protorothyrididae (a paraphyletic group with *Hylonomus*, *Anthracodromeus*, *Paleothyris*, and *Protorothyris* are successive more crownward genera), Araeoscelidia (*Araeoscelis* and *Petrolacosaurus*), and Varanopidae (traditionally a clade within Synapsida), in order of increasing phylogenetic proximity to Neoreptilia. Their fossilized birth/death Bayesian analysis found *Hylonomus* and a clade consisting of (*Palaeothyris* + (*Anthracodromeus* + *Protorothyris*)) as successive more crownward outgroups to their newly defined Diapsida, that is (Araeoscelidia + (Varanopidae + Neoreptilia)). Their parsimony analysis found a similar topology, except that *Anthracodromeus*, *Hylonomus*, and (*Paleothyris* + *Protorothyris*) were collapsed in a tetrachotomy with diapsids in their strict consensus tree.

Another recent, large-scale study, by Simöes et al. [8], removed araeoscelidians from diapsids and placed them on the amniote stem as sister group to a clade consisting of protorothyridids (represented by *Protorothyris*) plus captorhinids. In their Bayesian analysis, this broader araeoscelidian-captorhinid-protorothyridid clade, in turn, formed the sister group to crown amniotes. In addition, varanopids reverted to their traditional position among synapsids, whilst parareptiles were paraphyletic relative to neodiapsids.

The aim of this paper is fourfold: (1) to redescribe the only known specimen of the protorothyridid *Coelostegus prothales*, based upon new data from photogrammetric scanning, and compare it with other protorothyridids; (2) to present a new diagnosis for this taxon; (3) to produce a new reconstruction of its skull in dorsal and lateral views; (4) to assess its phylogenetic position. As regards our fourth point, we have not produced an overarching synthesis of recently published taxon-character datasets, as this is part of ongoing research. For this reason, we present only interim solutions to the wider issues of eureptile monophyly, the status of protorothyridids, and the affinities of *Coelostegus*.

## Brief historical background

*Coelostegus prothales* belongs to the diverse tetrapod fauna from Nýřany, Czech Republic (Middle Pennsylvanian). The importance of this fauna, and that of the coeval site of Třemošná, lies in the fact that several of its constituent taxa have been implicated in discussions on amniote ancestry [9]. The history behind the discovery, descriptions, and revisions of these taxa was narrated by Dr Andrew R. Milner (in [10]) and only a few relevant points are presented here. The taxonomic history of *Coelostegus* is inextricably linked to that of another Nýřany tetrapod, the ‘reptiliomorph’ *Gephyrostegus bohemicus* [9–11], traditionally considered to represent an intermediate level of morphological organization between two other putative stem amniote groups, the anthracosaurs and the seymouriamorphs [11]. In their extensive revision of *Gephyrostegus*, Brough and Brough [6] expanded the hypodigm of *G*. *bohemicus* by adding four Nýřany specimens to it. Two of these belong to the amniote-like *Solenodonsaurus janenschi* [12]. The remaining two specimens were labelled as I and II by Brough and Brough. Following a new revision of *Gephyrostegus*, Carroll [9] concluded that *Solenodonsaurus* should be re-instated as a distinct taxon and that Specimens I and II should be regarded as separate amniote taxa. Subsequently, Carroll and Baird [3] formally named and described Brough and Brough’s Specimen II as *Coelostegus prothales*, while Specimen I became the holotype of *Brouffia orientalis* [10].

## Material and methods

### Specimen preservation

**Fig 1:**
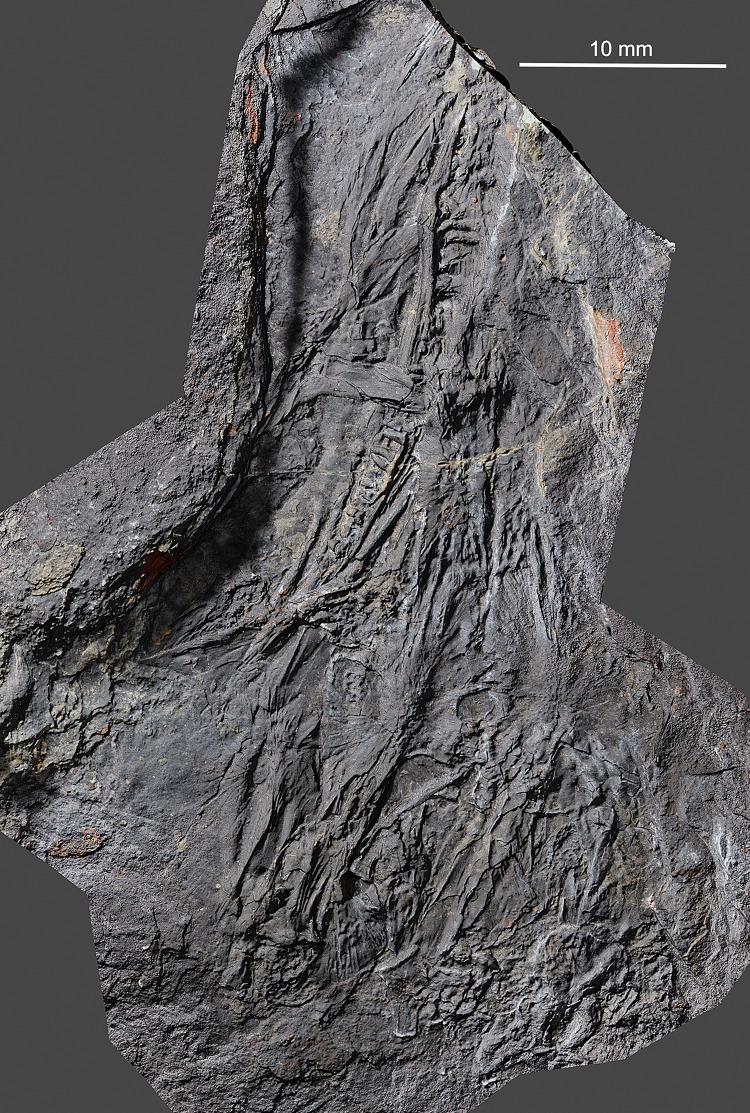
*Coelostegus prothales* Carroll and Baird, 1972. Virtual 3D model of skull.

**Fig 2:**
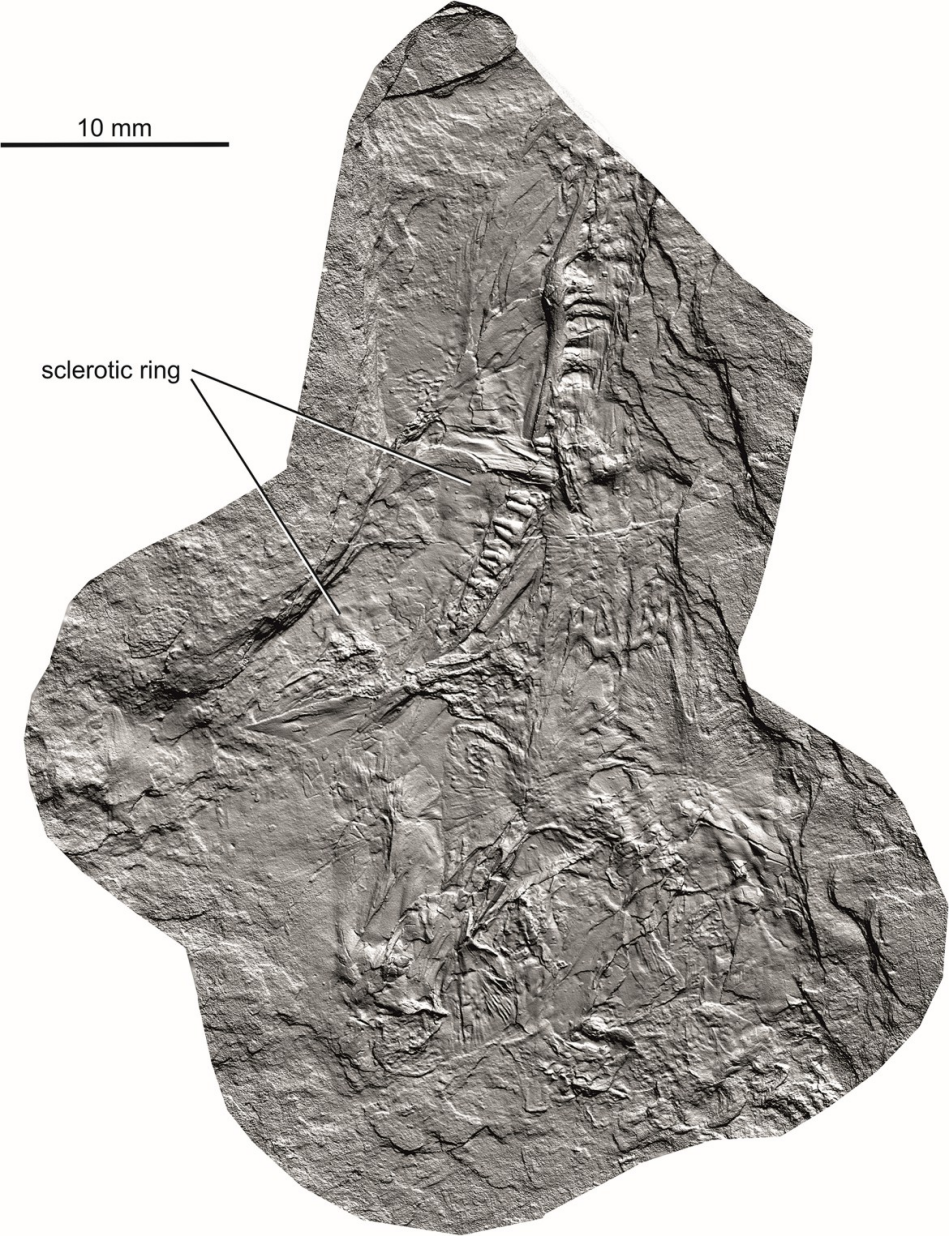
*Coelostegus prothales* Carroll and Baird, 1972. Virtual 3D model of skull without colours (textures).

**Fig 3:**
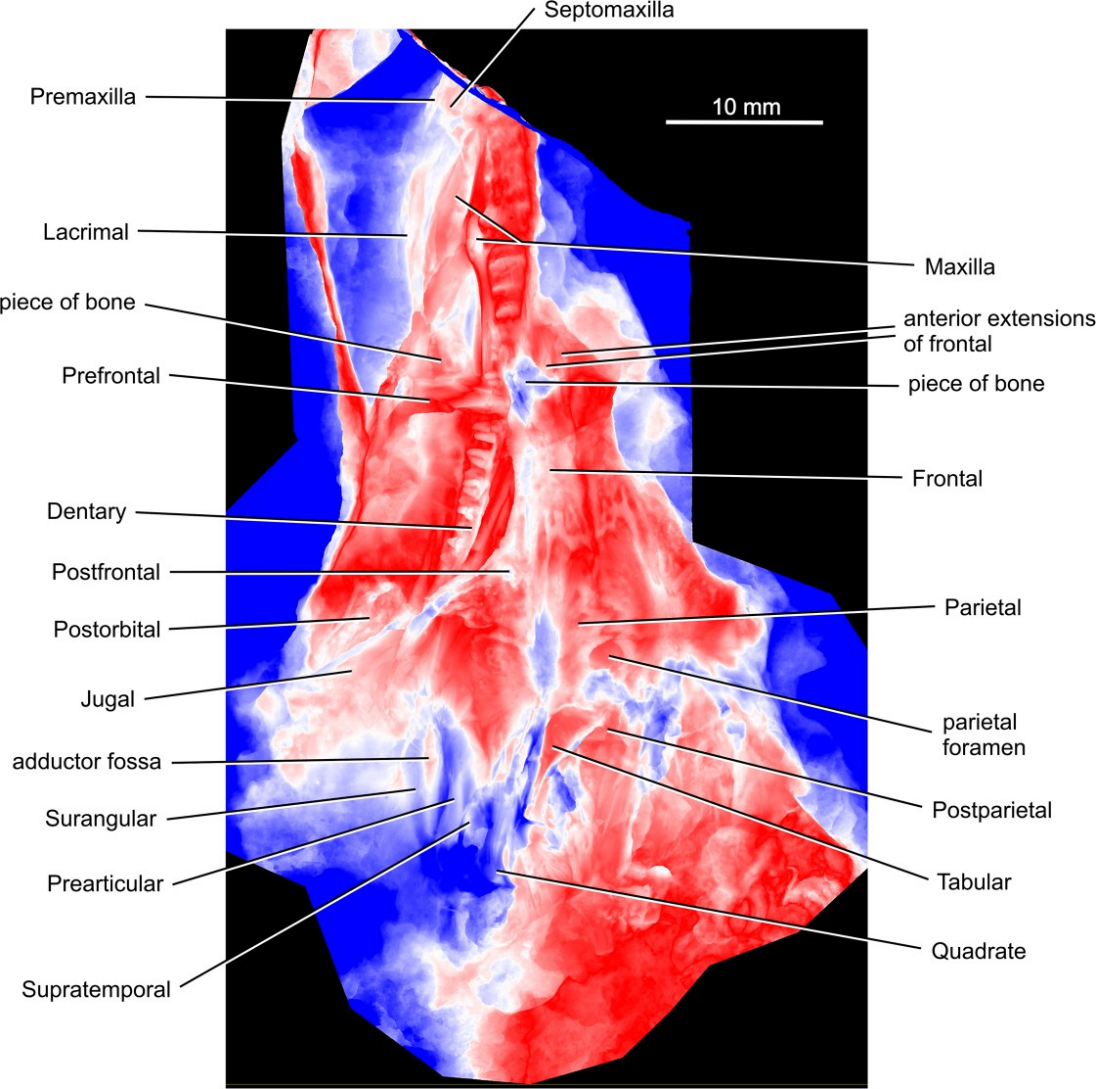
*Coelostegus prothales* Carroll and Baird, 1972. Color scale digital elevation model of the skull.

**Fig 4:**
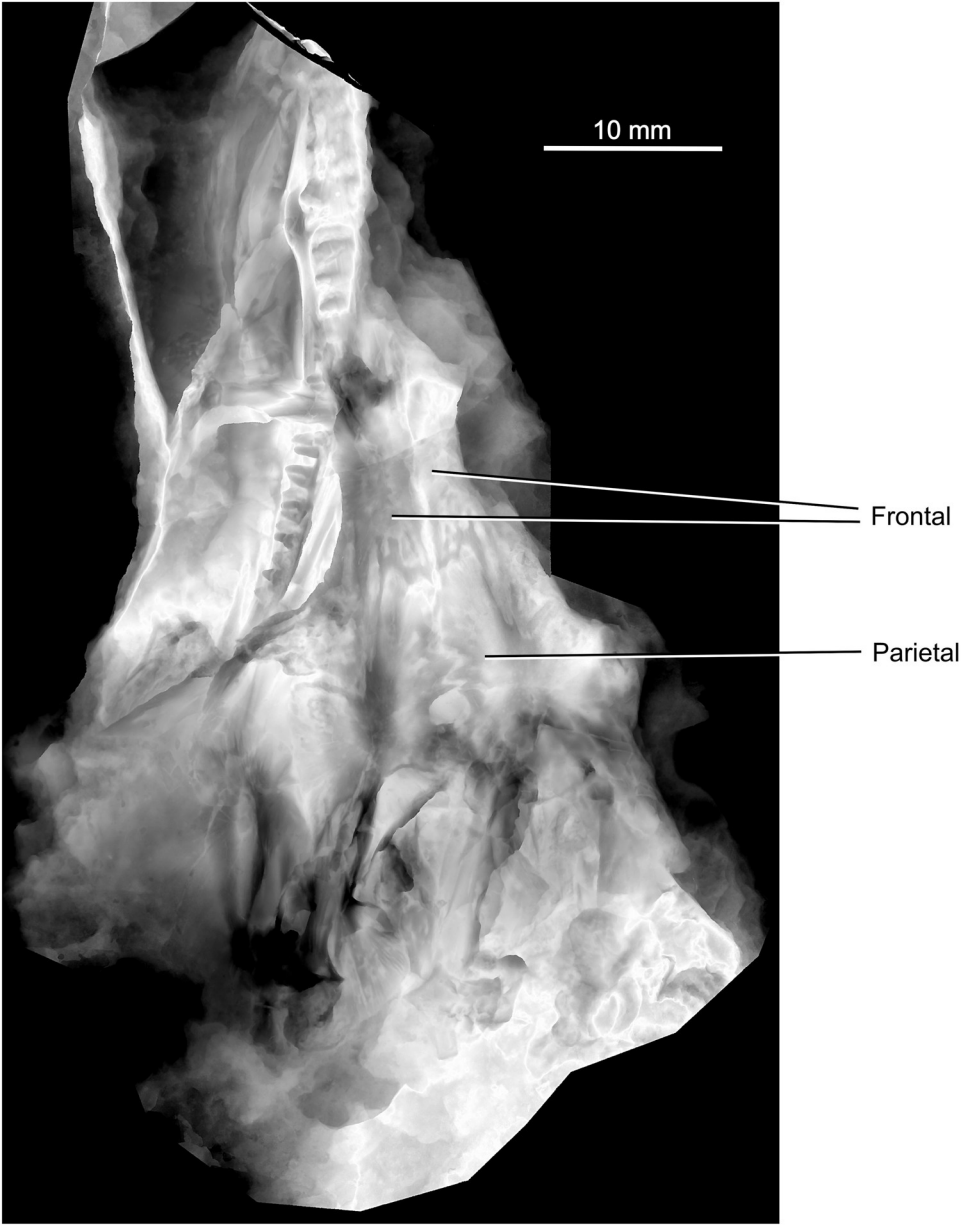
*Coelostegus prothales* Carroll and Baird, 1972. Grey scale digital elevation model of the skull.

**Fig 5:**
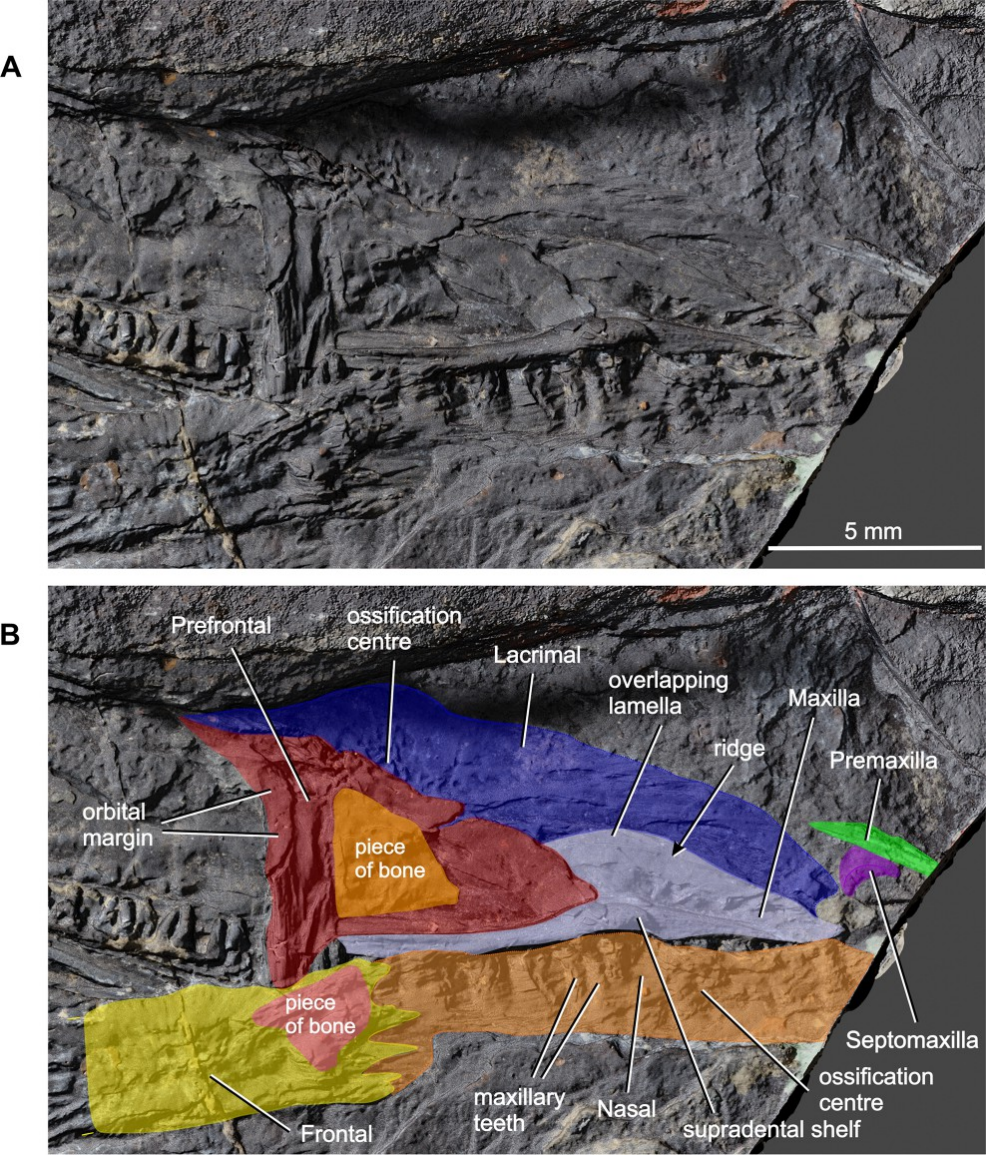
*Coelostegus prothales* Carroll and Baird, 1972. Virtual 3D model of the anterior portion of the skull as preserved (**A**) and with colour-coded individual bones (**B**).

**Fig 6:**
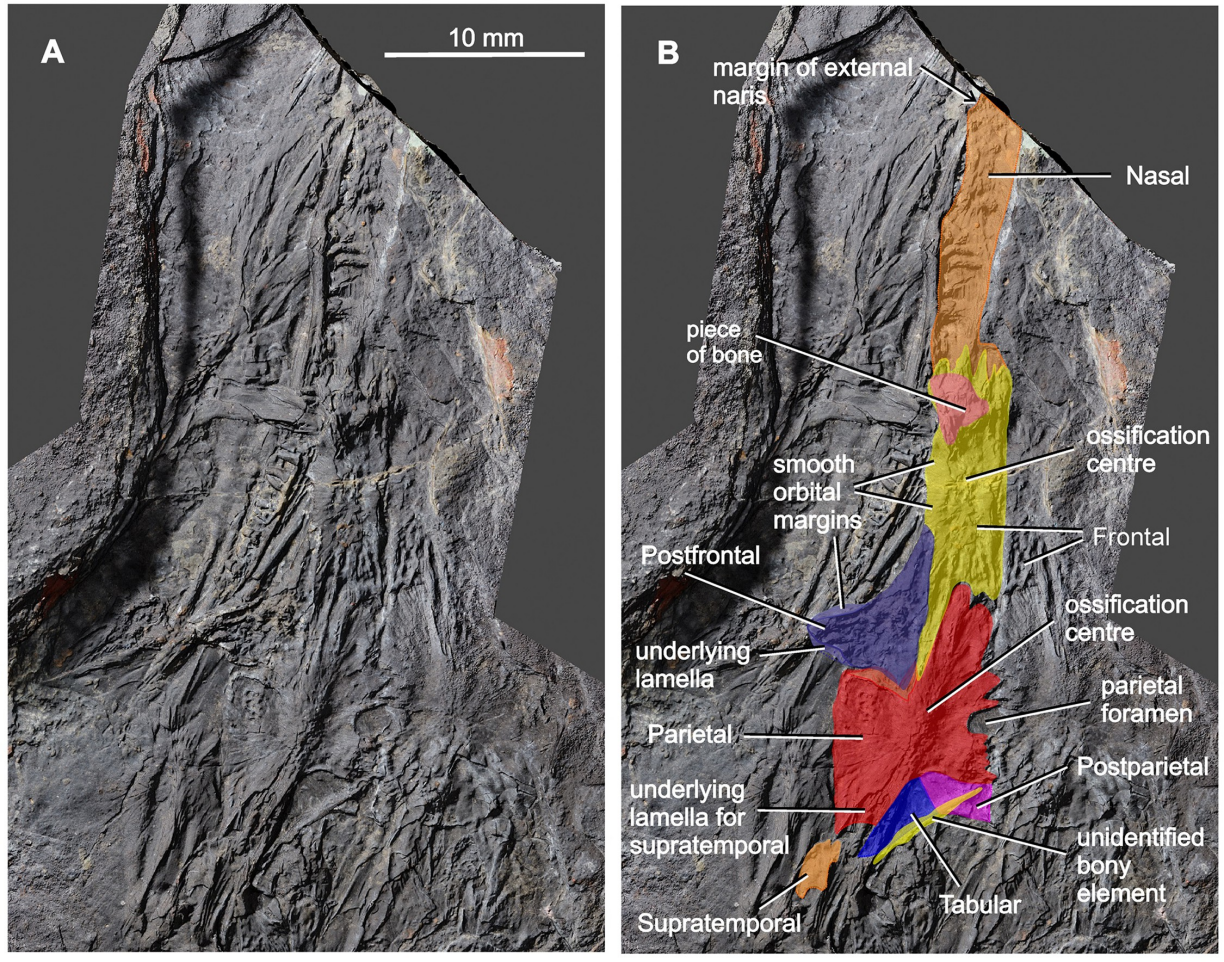
*Coelostegus prothales* Carroll and Baird, 1972. Virtual 3D model of the skull as preserved (**A**) and with colour-coded individual bones (**B**).

**Fig 7:**
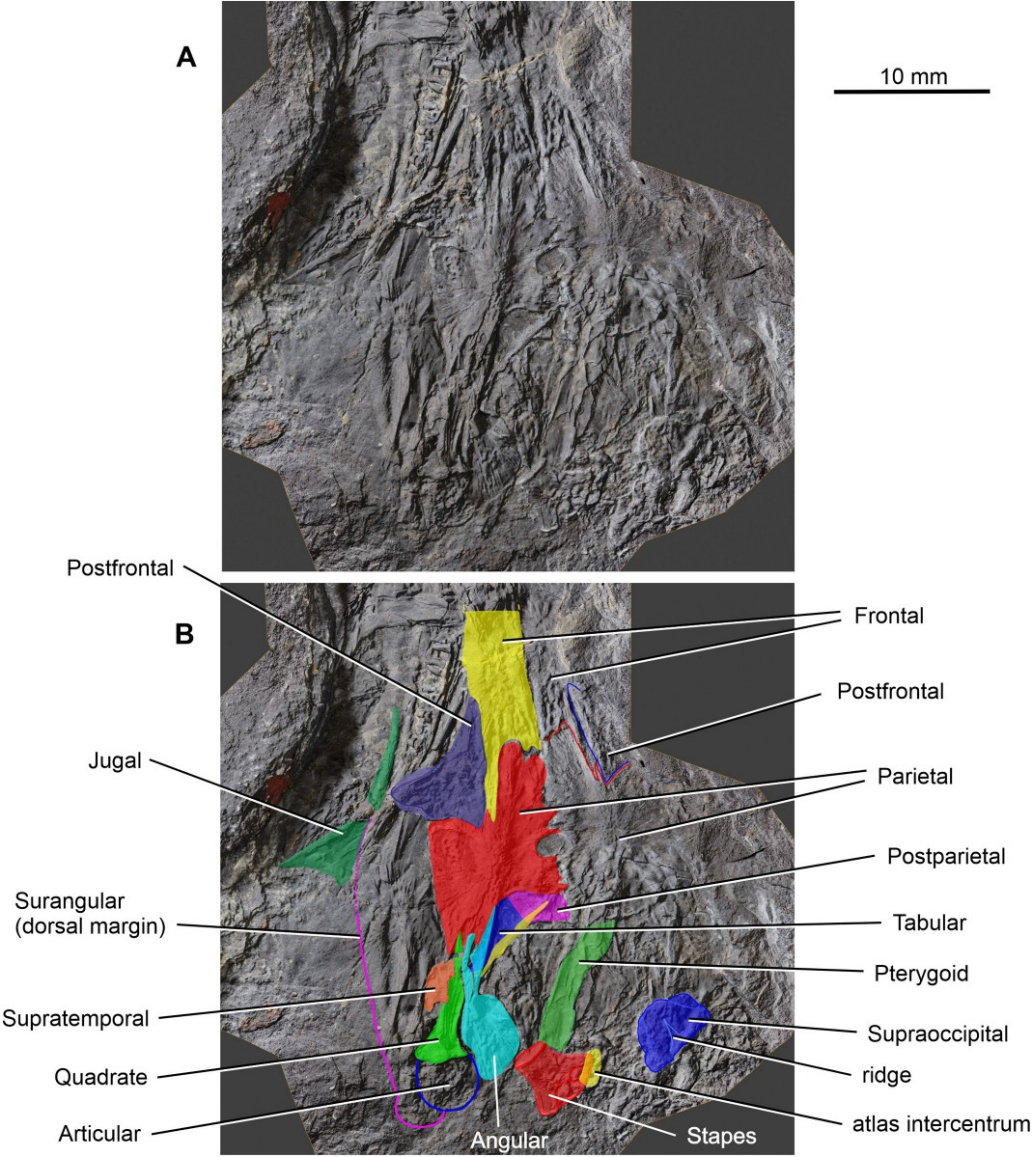
*Coelostegus prothales* Carroll and Baird, 1972. Virtual 3D model of the posterior half of the skull showing the skull roof and lower jaw elements as preserved (**A**) and with colour-coded individual bones (**B**).

**Fig 8:**
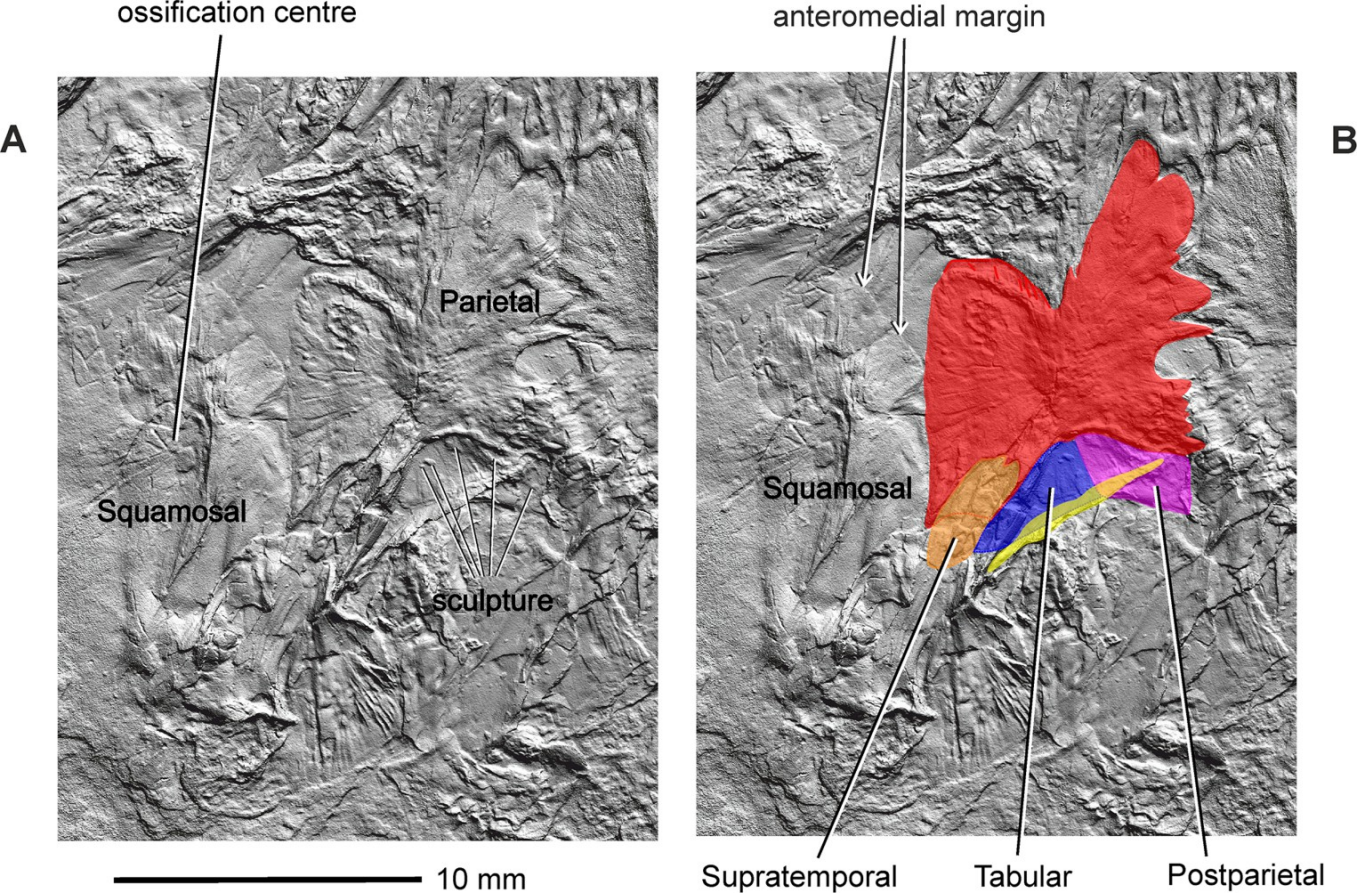
*Coelostegus prothales* Carroll and Baird, 1972. Virtual 3D model of the posterior portion of skull roof as preserved (**A**) and with colour-coded individual bones (**B**).

**Fig 9:**
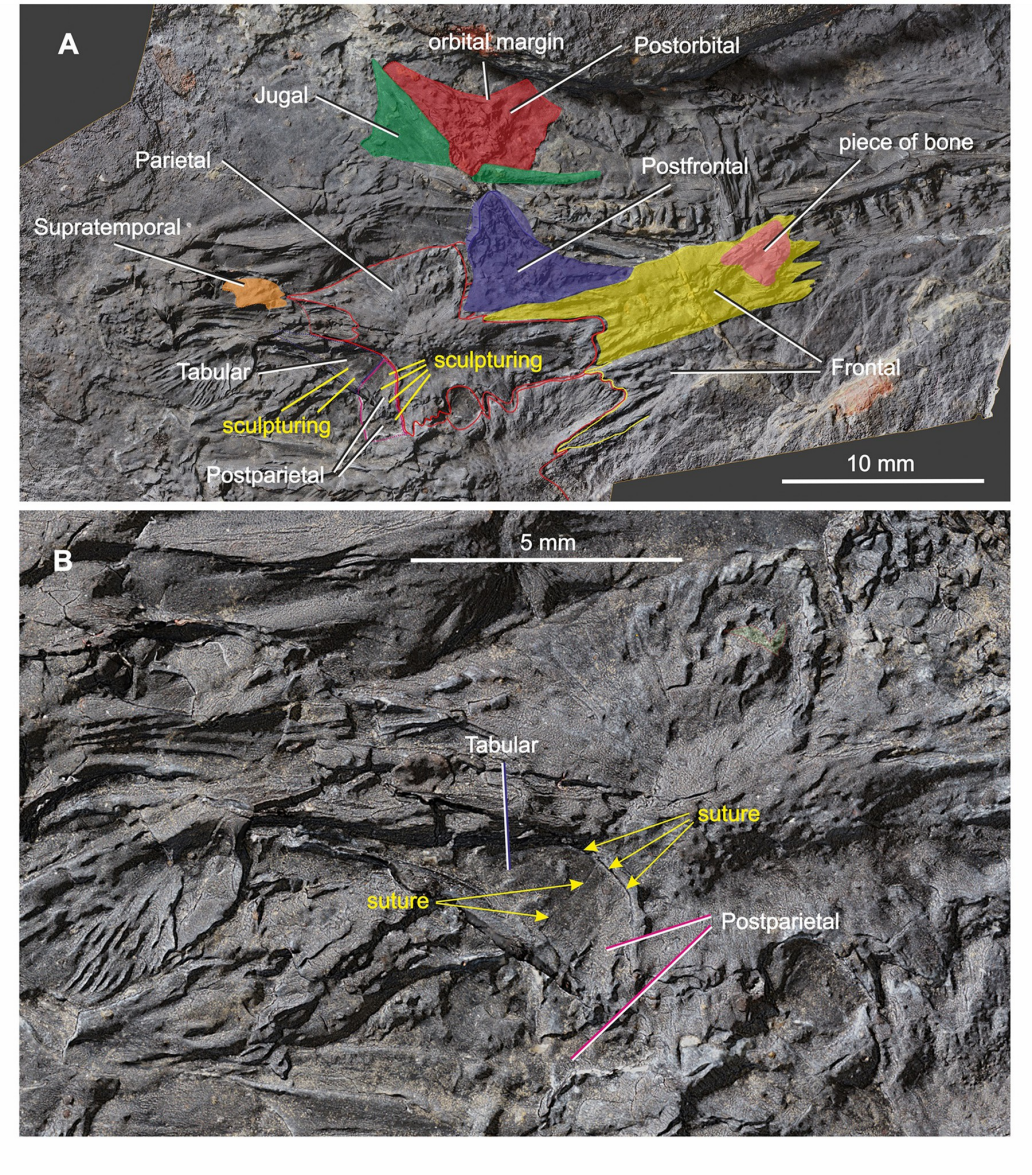
*Coelostegus prothales* Carroll and Baird, 1972. Virtual 3D model of the skull showing the detail morphology of the tabular and postparietal as preserved (**A**) and with colour-coded individual bones (**B**).

**Fig 10:**
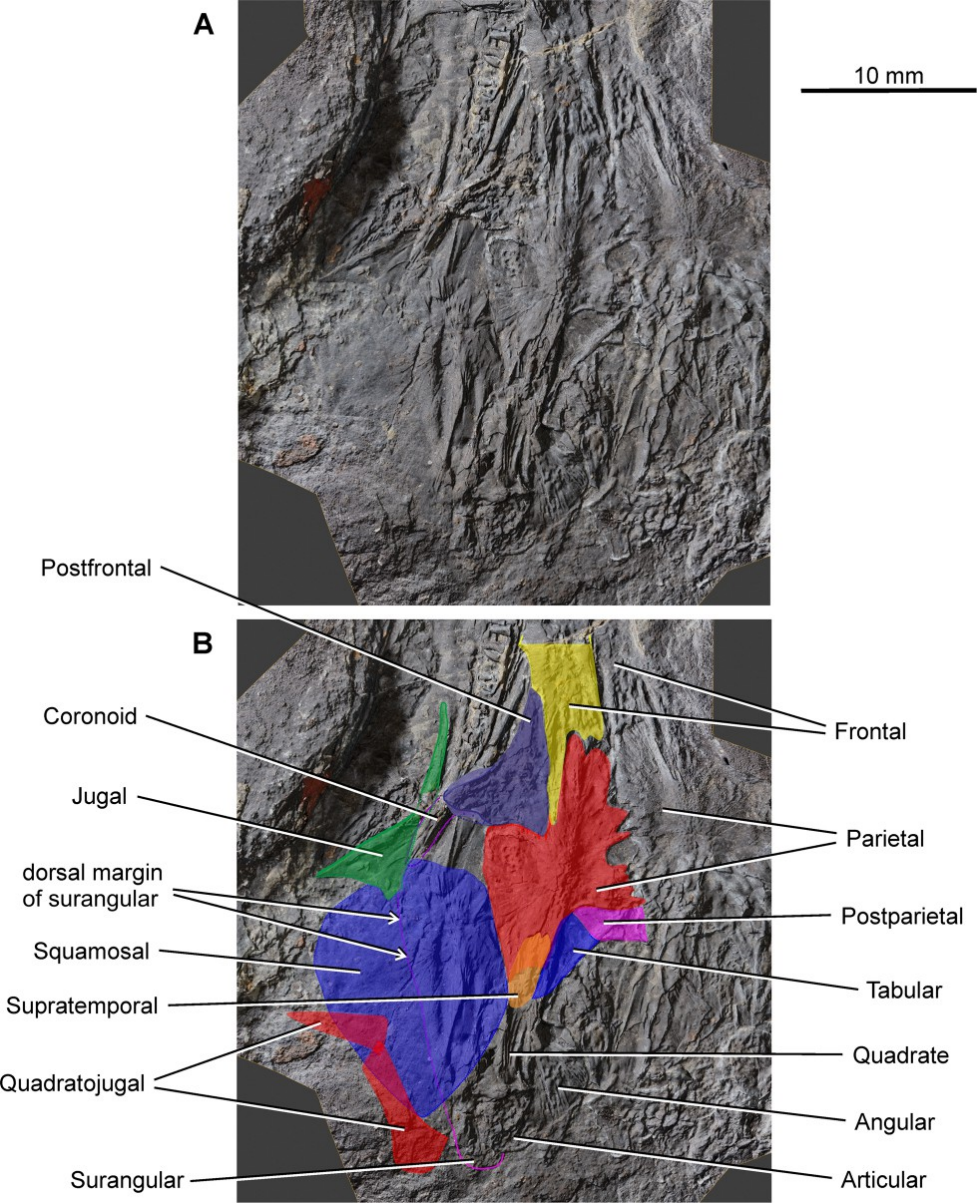
*Coelostegus prothales* Carroll and Baird, 1972. Virtual 3D model of the posterior portion of the skull showing the check bones relative to skull-table elements as preserved (**A**) and with colour-coded individual bones (**B**). In **B**, the shape of the supratemporal is reconstructed.

**Fig 11:**
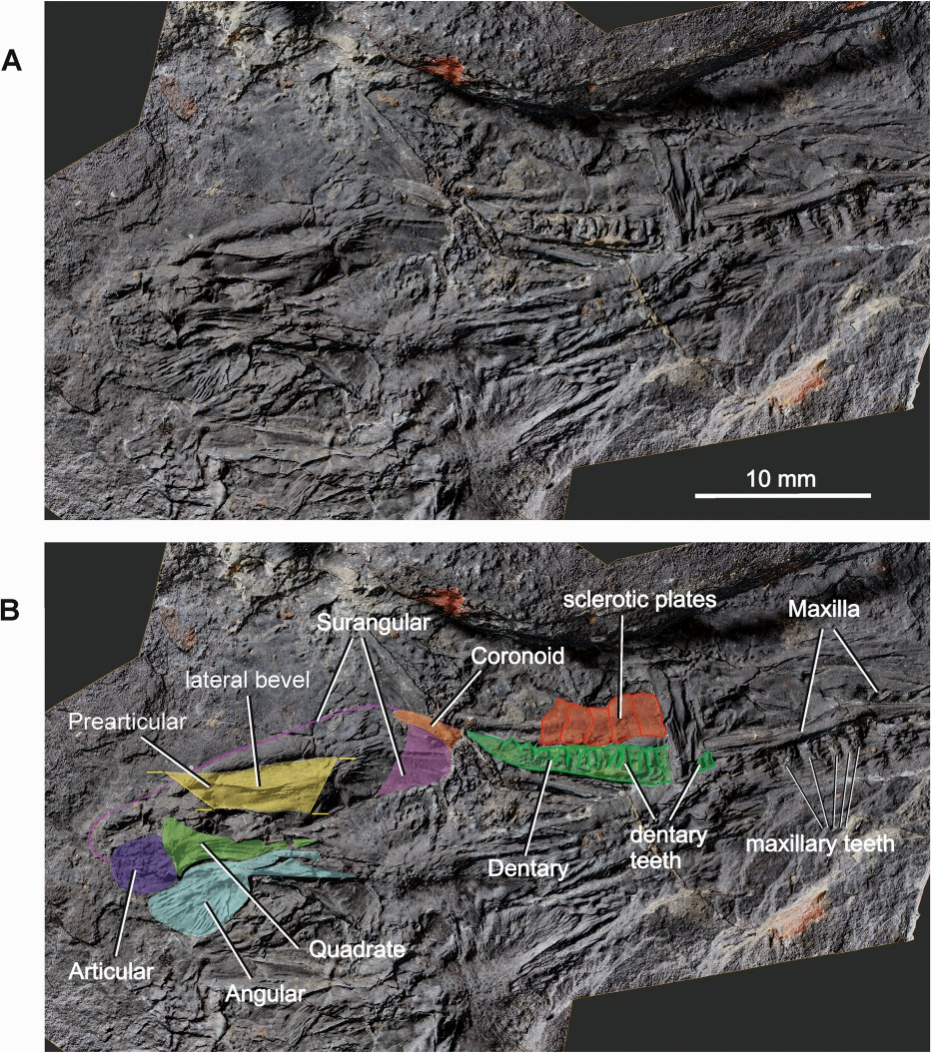
*Coelostegus prothales* Carroll and Baird, 1972. Virtual 3D model of the skull showing the lower jaw elements as preserved (**A**) and with colour-coded individual bones (**B**).

**Fig 12:**
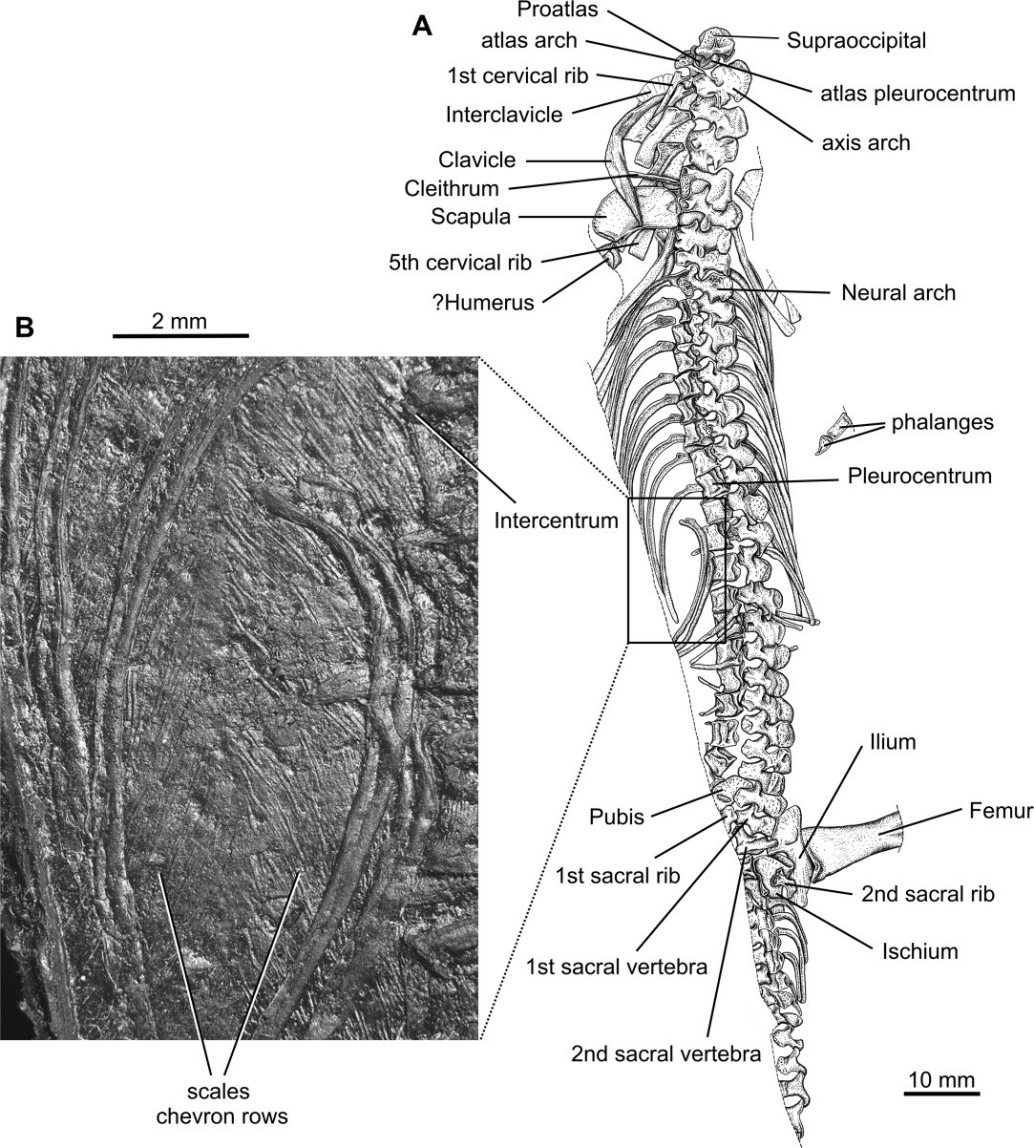
*Coelostegus prothales* Carroll and Baird, 1972. **A**. Drawing of the postcranial skeleton. **B**. Photograph of ventral scales.

The holotype of *Coelostegus prothales* consists of a partially articulated and incomplete skeleton preserved as an external mold on a single block of matrix (Figs 1–12) with no counterpart. The impressions of the skull and postcranium reveal fine anatomical details, despite a substantial amount of diagenetic compression. The skull is exposed in dorsal view. Most of its left-hand side is preserved, although heavily disarticulated in places (e.g., in its anteriormost part). Also visible are some bones from the right-hand side, an incomplete left sclerotic ring, the posterior half of the left lower jaw, including the rearmost portion of the left dentary, the unpaired supraoccipital, and the right stapes (Figs 1–4). The left jugal, maxilla, prefrontal, lacrimal, and lower jaw are the only elements preserved in internal view. Both the pectoral girdle (mostly from the left-hand side) and the pelvic girdle (presumably including elements from both sides) are also visible. The vertebrae and ribs from the trunk and proximal tail region are either fully articulated or only slightly displaced, and several of them are complete. The vertebrae have been rotated by 90 degrees due to compaction, such that their left-hand sides are exposed. Most limb elements and most of the tail are missing. An extensive cover of ventral scales is also preserved (Figs 1–12). The degree of ossification indicates that the holotype belonged to an immature individual [3].

### Photogrammetric scanning, specimen visualization, and reconstruction

To obtain high-resolution 3D renditions of the individual cranial bones, we used photogrammetric scanning (Figs 1–11), utilizing the following automated scanning workflow. The specimen was placed on a Cognysis Rotary Table to enable rotation around the Z axis (here, the axis of greater elongation) during scanning. Four studio flashes were used to produce adequate specimen illumination. To eliminate glares and shadows, this whole setup was placed in a lightbox. Digital images were captured from several angles using a digital full-frame Canon 5DSR camera equipped with a Canon MP-E 65mm macro lens and a 50Mpix CMOS sensor. The camera was placed on a Cognysis Macro Rail to ensure that the entire specimen surface was in focus. The digital images were processed using the Helicon Focus image stacking software. High-resolution 3D models of the individual cranial bones were built from stacked images in the Agisoft Photoscan photogrammetric software and rendered through CloudCompare and Blender.

**Fig 13:**
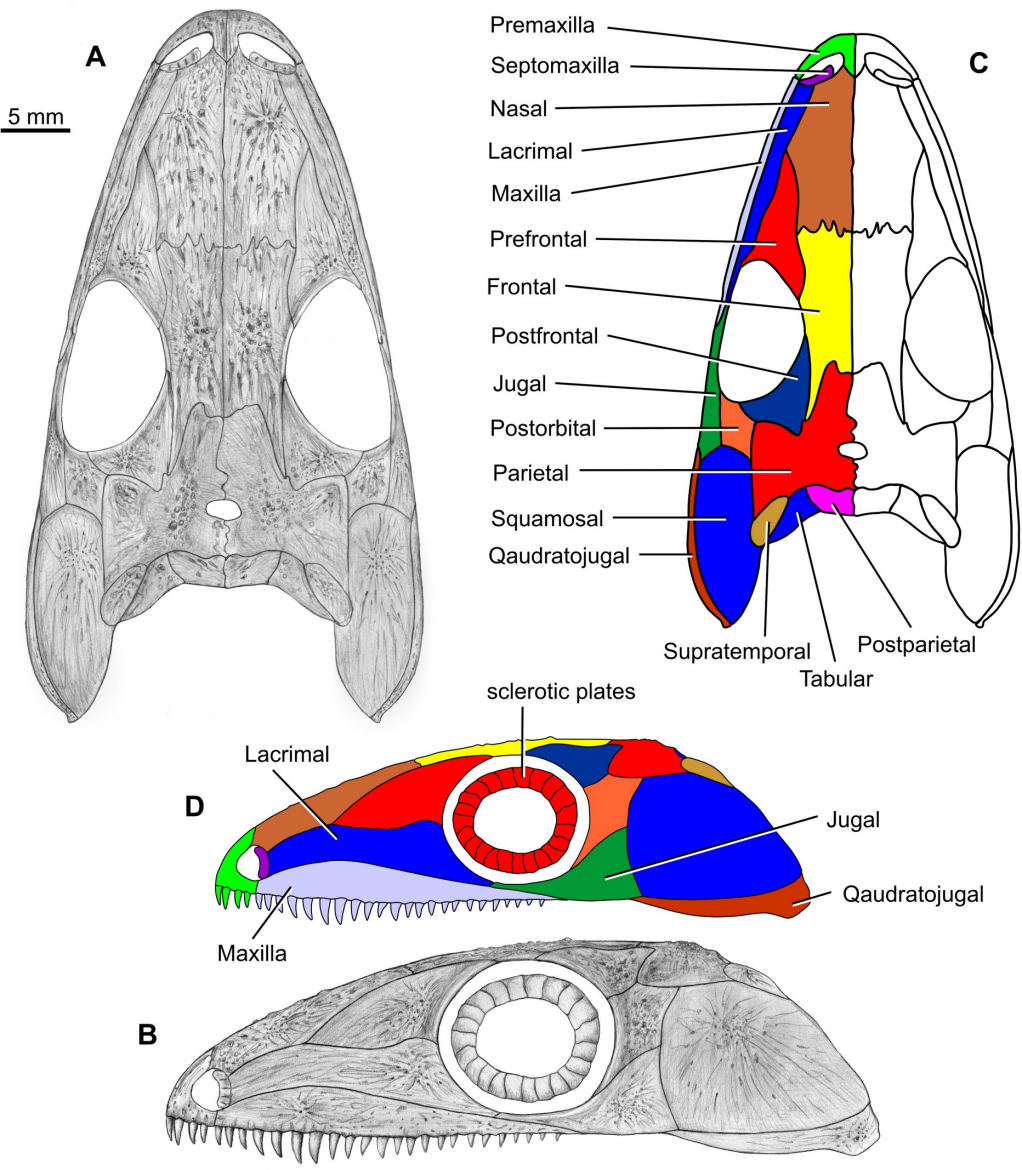
*Coelostegus prothales* Carroll and Baird, 1972. Reconstruction of the skull in dorsal (**A**) and left lateral (**B**) views alongside colour-coded and labelled skull diagrams (**C**, **D**).

Although the preservation of the specimen is challenging, it was possible to trace the complete outlines of most skull bones by varying the angle of illumination during photography (Figs 1–4). To assist the reader with the interpretation of the material, we use transparent color-coded layers to identify the extent and position of individual bones on the photographs (Figs 5–11). The cranial reconstruction (Fig 13) is based upon a scaled-up wax plasticine replica of the skull, with individual bones modelled at 10x natural size and held in place with metal bars.

### Phylogenetic analysis

To evaluate the phylogenetic position of *Coelostegus*, we included this taxon in the recently published cladistic data matrices by Ford and Benson [7] (S1 Appendix) and Simöes et al. [8] (S2 Appendix) and carried out maximum parsimony tree searches on both matrices using equally weighted characters. In the case of the Simöes et al. dataset, tree searches proved to be extremely time-consuming. For this reason, parsimony tree searches were stopped when 3·10^5^ most parsimonious trees were obtained. In the case of the Ford and Benson dataset, we also ran parsimony analyses under two character-weighting regimes, that is, simple re-weighting and implied weighting. Furthermore, we also carried out an uncalibrated Bayesian analysis. These additional analyses were not performed with the Simöes et al. dataset, again due to its memory-intensive requirements.

A small modification of the Ford and Benson matrix consisted in the addition of a new character (295 here), as follows: posteromedial lappet of the postfrontal: absent (0); present (1). This character describes a peculiar configuration of the posteromedial corner of the postfrontal in some taxa, whereby this part of the bone extends into a lappet-like process of variable proportions, accommodated by a notch or groove in the parietal. The process in question has been observed and described in various amniotes, but we are not aware of works that have commented upon its possible phylogenetic utility or functional relevance.

For both datasets, we carried out parsimony analyses in PAUP* v. 4.0a.169 [13], with all characters treated initially as having equal unit weight. Tree searches employed 50,000 random stepwise addition sequences using a tree bisection-reconnection branch-swapping algorithm, retaining one tree in memory at each step. We used all trees saved from this run as input for a new round of branch swapping, saving multiple trees. This procedure was repeated ten times. The conflict among the equally parsimonious trees was summarised with a strict consensus and an agreement subtree (a pruned tree that consists of the largest subset of taxa for which all the most parsimonious trees agree upon mutual relationships). To evaluate node support, we bootstrapped and jackknifed [14, 15] the two datasets, employing in each case 100,000 replicates of character resampling under the fast stepwise addition option in PAUP*, with 50% of character deletion under jackknifing.

For the Ford and Benson dataset, a parsimony analysis using simple re-weighting employed the maximum value (best fit) of the rescaled consistency indices of characters, such as was obtained from the initial analysis with equally weighted characters. For the parsimony analysis with implied weights [16], we assigned a value of 12 to the constant of concavity *K*, following recommendations in [17]. Lastly, the non-calibrated Bayesian analysis of the Ford and Benson dataset (S3 Appendix) employed identical settings to those used by those authors, except for the number of generations (10^7^ in the present study). The Bayesian analysis was undertaken in MrBayes v. 3.2.6 [18] (for analysis output, see S4 Appendix).

## Results

### Systematic paleontology

Tetrapoda Jaekel, 1909 [19]

Eureptilia Olson, 1947 [1]

*Coelostegus* Carroll and Baird, 1972 [3]

#### Generic diagnosis

As for the only species, *Coelostegus prothales* Carroll and Baird, 1972 [3].

#### Type species

*Coelostegus prothales* Carroll and Baird, 1972 [3].

*Coelostegus prothales* Carroll and Baird, 1972 [3]

(Figs 1–13)

*Gephyrostegus bohemicus* Jaekel, 1902 [19]; Brough and Brough, 1967:147–148; specimen II, Figs 2C and 10C [11].

*Coelostegus prothales* Carroll and Baird, 1972:341, Fig 7 [3].

#### Holotype

M 4909 (formerly Č.G.H. 3027; Č.G.H.), National Museum, Prague, Czech Republic) (Figs 1–4).

#### Locality, Horizon, and Age

Nýřany ‘Gaskohle’ from Nýřany Colliery, near Nýřany, south-west of Plzeň, Czech Republic; apex of Lower Grey Beds of the Plzeň-Manětín Basin; Asturian (Upper Westphalian D), Moscovian, Late Carboniferous (Middle Pennsylvanian).

#### Revised diagnosis

Amended after Carroll and Baird [3]. Supratemporals subelliptical in outline, extending for less than half of their length (measured along oblique axis of bone) onto adjacent squamosal. Tabular and postparietal accommodated by deeply embayed posterior margin of parietal. Parietal with extensive lateral lappet. Anterior margin of lappet slightly convex and obliquely orientated. Pre-pineal region of parietal corpus subtrapezoidal. Postfrontal with broad, rounded triangular posteromedial lappet. Dorsal margin of lacrimal with low, convex lappet immediately anterior to orbit margin. Lacrimal deepest at level of lappet. Dermal sculpture on frontal, postfrontal, and postorbital characterized by deep grooves. Frontals shorter than nasals and with elongate posterolateral processes extending posterior to orbits. Nasals expanded laterally immediately in front of their mid length. Numerous small posterior maxillary teeth. Slightly enlarged maxillary teeth forming a “caniniform region”. Twenty-nine presacral vertebrae. Two sacral vertebrae. Vertebral centra relatively short. Scapular and coracoid separate (possibly due to immaturity). Neural arches and centra unfused.

### Description

#### Skull roof

Although not mentioned by Carroll and Baird [3], the premaxilla features in their Fig 14B. It is visible at the anterior end of the snout (Figs 3 and 5) and is preserved as a narrow, slender, and rod-like splint of bone with an acuminate tip, possibly representing an incomplete nasal ramus. The ventral ramus is missing. From its tip, the nasal ramus becomes gradually wider before narrowing slightly at its mid-length. After a short tract, the lateral margin of the ramus curves laterally. In contrast, its medial margin is straight throughout its length. The anterior and middle one-thirds of the preserved portion of the nasal ramus bear anteroposteriorly orientated grooves and ridges. One ridge running parallel to the medial margin of the ramus stands out due to its unusual thickness. The bone surface between adjacent ridges is smooth. In the absence of additional material, it is difficult to ascertain whether this ridged surface is external or internal. If it is indeed external, then its sculpture differs from that of the other skull roof bones. Based upon this observation, we are inclined to conclude that the premaxilla is from the right-hand side and preserved in internal aspect.

**Fig 14:**
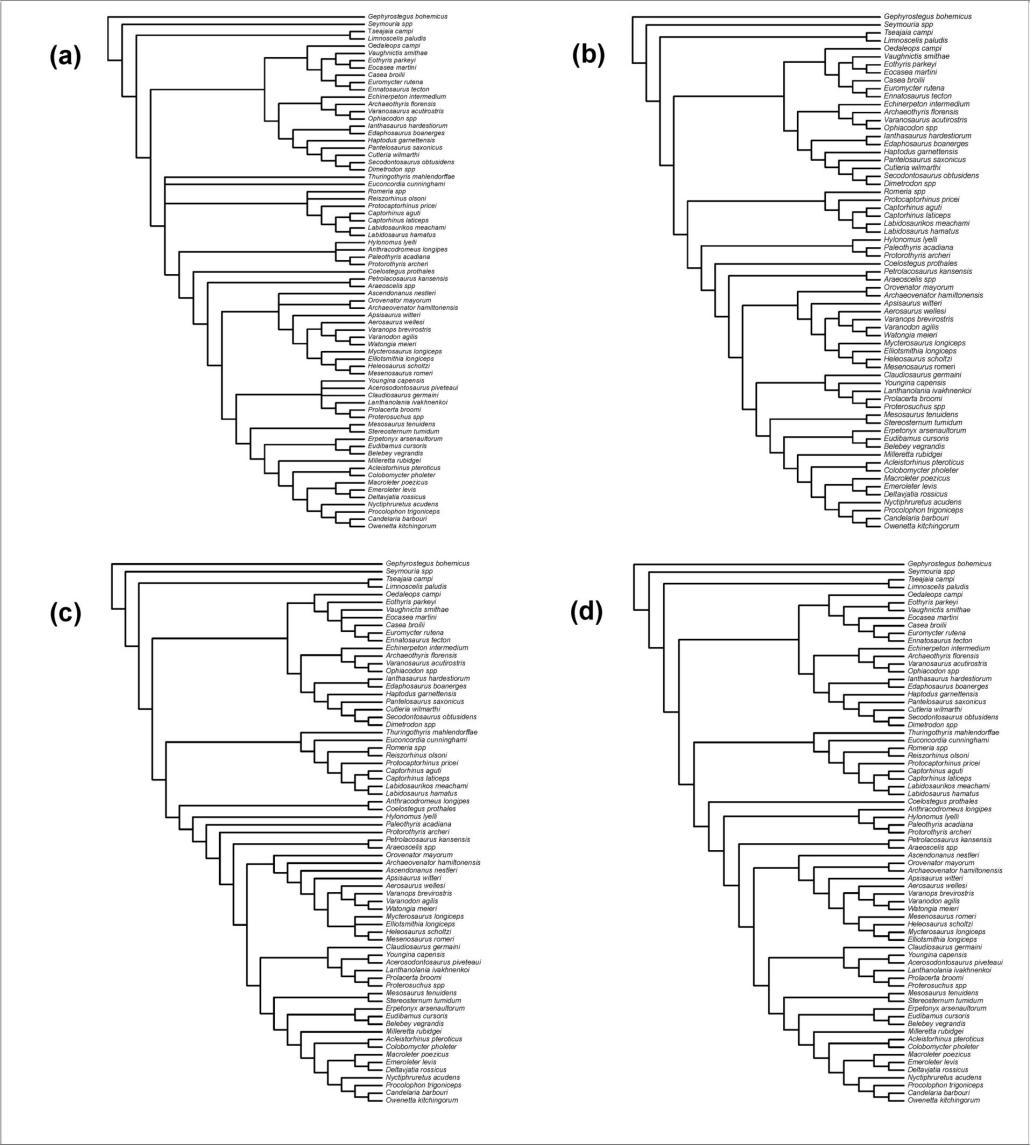
Interrelationships of *Coelostegus prothales*. **A**. Strict consensus of 32 shortest trees from a maximum parsimony analysis of Ford and Benson’s [7] dataset with all characters having equal unit weight. **B**. Agreement subtree of the 32 shortest trees. **C**. Single most parsimonious tree obtained after re-weighting characters by the largest value of their rescaled consistency index. **D**. Single most parsimonious tree obtained under implied weighting using *K* = 12 for Goloboff’s constant of concavity [16, 17].

The left septomaxilla is tucked between the premaxilla laterally and the left maxilla and left lacrimal immediately posterior to it (Figs 3 and 5). It is a smooth, curled sheet of bone with a semilunar profile and a slightly thickened posterior margin, which is likely to represent part of the rim of the external naris [3]. The lightly sculptured dorsal surface of this margin suggests that it participated in the formation of the skull roof.

Most of the left nasal is preserved in dorsal view, and only its anteriormost portion is missing (Figs 5 and 6). As preserved, the nasal is longer than the frontal (excluding the posterolateral process of the latter). Carroll and Baird [3] regarded this condition as one of the diagnostic features of *Coelostegus prothales*. The slightly disrupted lateral margin of the nasal is appressed against the ventral wall of the supradental shelf of the maxilla. Its posteromedial margin is partially covered in sediment and the anteromedial part of its sculptured surface has been eroded away. The centre of ossification lies slightly anterior to the mid-length of the bone (Fig 5) and consists of distinct and irregularly shaped tubercules. Radiating out from the centre of ossification and extending to the periphery of the bone are low, broad, and smooth-surfaced ridges alternating with narrow grooves. The morphology of the ridges is best observed in the posterior half of the nasal. In its anterior part, the ridges and grooves are orientated anteroposteriorly. Part of the external naris is visible in the form of a smooth, rounded notch in the anterolateral corner of the nasal, presumably receiving the adjacent septomaxilla in life.

The left prefrontal is a large triangular bone (Figs 3 and 5). Like the maxilla and lacrimal, it is flipped mediolaterally so that its medial margin is orientated towards the left-hand side of the skull and its internal surface is exposed. The central part of this surface is covered by a bony fragment that we have not been able to identify. Irregular grooves and ridges of varying thickness, visible immediately anterior to this bony fragment, traverse the internal surface anterolaterally. An elongate, shallow depression runs parallel to the lateral margin. The slightly thickened orbital margin shows a smooth texture. Anterior to the triple sutural joint between the prefrontal, frontal, and nasal, the prefrontal forms a robust, triangular sheet of bone wedged between the lacrimal and the nasal and extending as far anteriorly as the nasal mid-length. The stout, subtriangular posterior ramus forms a large proportion of the anteromedial quarter of the orbit margin and narrows abruptly to a point posteriorly. Its medial margin is straight in its anterior half and gently convex in its posterior half and meets the orbit margin slightly anterior to the orbit mid-length. The posteroventral ramus is much shorter and more robust than the posteromedial ramus.

The left frontal is preserved in its entirety. In contrast, only the middle and posterior one-thirds of the right frontal are visible (Figs 3–6 and 9). The frontal is elongate and rectangular, with a deeply sculptured central area covered in pits and a system of radiating ridges and grooves. Its posterolateral corner forms a sharp, elongate process directed slightly posterolaterally and narrowly wedged between the parietal and the postfrontal. The extremity of the process extends slightly posterior to the mid-point of the pre-pineal portion of the inter-parietal suture and approaches the anteromedial corner of the parietal lappet. The anterior and posterior margins of the frontals form deeply interdigitating sutures with the nasals and parietals, respectively (Figs 3 and 4), while their medial and lateral margins are smooth. The frontals contribute to the posterior one-third of the anterodorsal quadrant of the orbit margin.

The triangular postfrontal is preserved in close proximity to the frontal and parietal (Figs 3, 6 and 7 and 9–11). Its external surface shows an irregularly tuberculate sculpture accompanied by deep grooves, and its orbital margin becomes increasingly less deep in its anterolateral portion. The postfrontal extends anteriorly just under halfway along the length of the frontal and its anterior extremity fits into a small recess at the posterior extremity of the orbital margin of the frontal. The broad posteromedial portion of the postfrontal forms a tongue-like process situated between the posterior extremity of the posterolateral process of the frontal and the anterior margin of the parietal lappet (see description of parietal below). The posterolateral portion of the postfrontal forms a stout and abbreviated process, with a disrupted underlying lamella along its free margin.

The parietal has a distinctive, albeit not unique, morphology (Figs 3 and 4 and 6–10). It is divided into a main corpus and a lateral lappet. The boundary between these two regions coincides with a parasagittal line passing through the triple sutural joint between parietal, frontal, and postfrontal. A narrow gap visible between the postfrontal and parietal exposes part of the underlying lamella of the parietal. The main corpus is subtrapezoidal and approximately 40% as wide as long. Its pre-pineal portion is flanked by the posterolateral processes of the frontals and narrows slightly anteriorly, forming an irregularly sinuous suture with the frontal. Its post-pineal portion forms a wide rectangular strip with a gently concave posterior margin. There is some evidence that such margin produced a small, posteriorly projecting triangular process with its antimere (Fig 5).

The broad lateral lappet narrows in a lateromedial direction, due to the oblique course of its anterior and posterior margins. Its lateral margin forms a nearly straight suture with the postorbital and the squamosal. The suture between the anterior margin of the lappet and the postfrontal has a smoothly curved, anteriorly convex profile. Medially, and close to the main corpus, this margin produces a small inflection point around which its curvature changes abruptly. Lateral to the inflection is a deep and anteriorly concave embayment that accommodates the posteromedial, tongue-like process of the postfrontal. Medial to the inflection is a small, posteriorly concave recess that is adjacent to the posterior extremity of the posterolateral process of the frontal. The posterior corner of the parietal lappet is occupied by a subelliptical depression, the greater axis of which is orientated in an anteromedial to posterolateral direction, and which accommodated the supratemporal in life (Fig 5). The peripheral area of this depression is deep, and the visible part of its surface is occupied by faint longitudinal striations. Further posteriorly, the depression is overlapped by the ascending process of the quadrate and by fragments of the angular (Fig 7).

The centre of ossification of the parietal is situated between the lateral lappet and the main corpus of the bone. It consists of a cluster of deep pits surrounded by radiating grooves and ridges that diverge to the outer margins, and is especially pronounced in the anterolateral and posterolateral portions of the lappet. The inter-parietal suture is markedly interdigitating. The parietal foramen forms a transversely elongate elliptical opening, and its posterior rim occurs within the posterior one-third of the inter-parietal suture.

The left supratemporal, tabular, and postparietal are visible along the posterior part of the skull. There is no trace of their right antimeres (Figs 4 and 6–10). According to Carroll and Baird (1972:342), “… the supratemporal, tabular, and postparietal have slipped from the parietal [embayment] and are mixed with displaced elements of the palate, occiput, and cervical vertebrae” [3]. However, we can confirm this scenario only in the case of the left supratemporal. The incomplete supratemporal is preserved as a small fragment immediately posterolateral to the subelliptical depression on the parietal lappet (Figs 6–10). The anterior part of this fragment, including the profile of its anterior margin, matches the parietal depression in size and proportions. Carroll and Baird (1972:Figs 7 and 14B) [3] portrayed the supratemporal as a proportionally much larger element than in our reconstruction (Fig 13), comparable in size to the combined postparietal and tabular, and with a pyriform outline, being narrower anteriorly and increasing rapidly in width in its middle and posterior one-thirds. This is because Carroll and Baird’s ‘supratemporal’ also included a large posterior section of a bony element that we interpret as the angular (see below).

Immediately behind the embayed and slightly thickened posterior margin of the left parietal are two bony elements, both featuring smooth and undamaged margins and a well-preserved dermal sculpture (Fig 9). Photogrammetry has revealed a narrow, almost straight, and obliquely orientated discontinuity between the two elements. We interpret this discontinuity as a sutural seam separating the left tabular (lateral element) from the left postparietal (medial element) (Figs 3 and 6–10). The tabular is shaped like an acute triangle (Figs 3 and 6–10) and is comparable in length with the adjacent supratemporal. Its gently curved anterior and posterior margins follow an oblique course and converge into a posterolateral acuminate process. The anteromedial portion of the tabular is overlapped by a narrow bony strip that is morphologically part of the angular (Fig 7). The sculpture on the dorsal surface of the bone consists for the most part of low tubercles (Figs 8 and 9). The suture with the postparietal is straight. Although its posteriormost portion is not clearly visible, it probably turns slightly posteromedially. The anterior end of this suture is almost aligned with the parietal lappet. Close to the posterior margin of the tabular is a tall and sharp bony ridge. This ridge is not morphologically part of the tabular. Rather, it belongs to an unidentified element that protrudes dorsally, disrupting the posterior margin of the tabular and partially fracturing the postparietal. We tentatively interpret this element as a small section of the quadrate ramus of the pterygoid (Figs 3 and 4 and 6–10).

The subtrapezoidal postparietal is wider than long (Figs 6–10). The median suture with its right antimere is not discernible but may be represented by a thin line visible immediately posterior to, and slightly to the right of, the post-pineal portion of the inter-parietal suture. It is overlapped by a bony fragment originally interpreted by Carroll and Baird (1972:Fig 14B) as the postparietal [3], and the identity of which remains elusive. The smooth posterior margin of the postparietal is slightly inclined posteroventrally. The sculpture on the dorsal surface of the bone is similar to that of the tabular (Figs 8 and 9). The anterior margin of the postparietal forms a distinct and slightly concave (posteriorly) suture with the medialmost portion of the thickened posterior margin of the parietal (Fig 9B).

The left maxilla is slightly displaced from its anatomical position and exposed in internal view. It is folded underneath the skull roof and visible between the left lacrimal laterally and the left nasal medially (Figs 3 and 5 and 11). Its anterior half is visible, whereas most of its posterior portion is overlapped by the prefrontal. Its anterodorsal extremity shows a small notch for the external naris, presumably receiving the septomaxilla in life. Several of the more anterior marginal teeth are concealed underneath the nasal. The maxilla attains its greatest dorsoventral depth approximately at the boundary between the anterior and middle one-third of its length and decreases uniformly in height posteriorly. Its posterior extremity is visible immediately anterior to the posterior margin of the orbit. A distinct ridge runs along the dorsal margin of the internal surface of the bone (Fig 5). The area situated dorsal to this ridge presumably represents the articulating surface for the lacrimal. This would indicate that the dorsal portion of the maxilla overlapped a narrow longitudinal strip of the lower external surface of the lacrimal. The supradental shelf is well-preserved along the entire length of the maxilla and appears considerably expanded mediolaterally in the region that corresponds to the insertion of the largest marginal teeth.

Lateral to the maxilla is the left lacrimal, preserved in internal view (Figs 3 and 5). It forms an anteroposteriorly elongate plate extending from the external naris anteriorly up the anteroventral portion of the orbit margin posteriorly. Various portions of the lacrimal are concealed by surrounding elements and/or matrix. Despite this, its general outline and proportions can be reconstructed. Its anteriormost portion is partially covered by a small lump of sediment, but its notched anterior margin, which contributes to the external naris, is discernible. Its posteroventral portion is partly overlapped by the prefrontal. Lastly, most of its anterior half is visible, except for a small anterior section of its ventral margin, which is overlapped by the maxilla (see above; Fig 5). The anterior half of the lacrimal forms an elongate and narrow plate with a gently convex dorsal margin. Its posterior half deepens slightly immediately in front of the orbit. At this level, the dorsal margin exhibits a smaller, but more pronounced convexity. The suborbital portion of the lacrimal decreases rapidly in height and terminates in a narrow bar that extends posteriorly almost to the level of the orbit mid-length. The surface of the anterior half of the lacrimal is traversed by anteroposteriorly orientated grooves and ridges of varying thickness. Except for its ventralmost portion, the well-preserved centre of ossification is clearly visible in the posterior portion of the bone (Fig 5). It consists of distinct tubercles and short ridges, from which a system of elongate grooves and ridges radiate out towards the dorsal margin of the bone.

According to Carroll and Baird, 1972:p. 343), “… the postorbital is not preserved, but its posterior extent may be judged by an area for its reception on the anterior portion of the squamosal” [3]. However, photogrammetry has helped identify the left postorbital in the form of a stout bony element displaced laterally from its anatomical position and slightly rotated anti-clockwise, such that its thickened orbital margin faces laterally (Fig 9). The postorbital has a vaguely chevron-shaped outline and is markedly asymmetrical in a mediolateral direction. The posterior half of the postorbital forms a robust, broadly triangular process. The nearly straight posteromedial margin of this process contacts the parietal lappet along an anteroposteriorly orientated suture. Its slightly longer and gently concave posterolateral margin is sutured with the dorsal half of the anterior margin of the squamosal. Anterodorsomedially, the postorbital forms a short suture with the postfrontal (this suture is proportionally longer in Carroll and Baird’s reconstruction [3]) but does not form a distinct process. Anteroventrolaterally, the portorbital is drawn into a long, rapidly tapering process (not identified by Carroll and Baird [3]), the posterior margin of which is overlapped by the slanting dorsal margin of the jugal corpus (see below). The irregularly pustulose postorbital sculpture occupies the central portion of the external surface of the bone and extends for a short distance along its anteroventrolateral process.

As in the case of the maxilla and prefrontal, the jugal (Figs 7 and 9 and 10) has shifted from its anatomical position and is preserved in internal view. Its posteroventral portion overlaps a small section of the posterodorsal region of the lower jaw as well as part of the squamosal. The jugal is almost completely exposed and has a vaguely cleaver-shaped outline in lateral view. The jugal consists of an anterior suborbital process and a posterior corpus. The suborbital process narrows gradually anteriorly and probably formed a very short suture with the lacrimal, just anterior to the mid-length of the orbit. Its straight, slightly oblique lower margin contacts the posterior portion of the dorsal margin of the maxilla. Its gently concave upper margin forms the largest contribution to the ventral half of the orbit. The subtrapezoidal corpus of the jugal participates in the formation of the ventral margin of the skull roof. Its dorsal margin is moderately convex and slopes along a posterodorsal to anteroventral direction. Its gently concave posterior margin contacts the lower half of the anterior margin of the squamosal.

The left squamosal is represented by a large, thin, subquadrangular sheet of bone tightly appressed against the posteroventral portion of the left jugal and most of the left surangular and prearticular (Figs 1–4). It is visible lateral and posterior to the parietal (Fig 10) and is displaced slightly posteriorly. As a result, a small gap occurs between the squamosal and the postfrontal. The thin, anterior portion of the squamosal is overlapped by the posteroventral portion of the jugal, and its ventral margin covers the posterior half of the quadratojugal. Medially, the straight suture between the squamosal and the lateral lappet of the parietal is clearly traceable. The squamosal projects behind the posterior margin of the skull table. Its sculpture, particularly pronounced in the anterodorsomedial portion of its external surface, consists of a small, pit-covered area surrounded by radiating grooves (Figs 8 and 10). Such grooves approach the periphery of the bone and aid in establishing its size and proportions (Fig 10).

Carroll and Baird [3] described the quadratojugal as a narrow bone with pointed anterior and posterior extremities. We identified the left quadratojugal in the form of a low relief impression near the posteroventral margin of the squamosal (Fig 10). The bone is broken into two fragments of approximately equal length. The fracture appears to have followed a nearly straight course. The anterior fragment has the shape of a narrowly acute triangle with an acuminate vertex pointing sideways (as preserved) and is covered in longitudinal grooves and ridges. We interpret this fragment as the anterior extremity of the quadratojugal. The shape of the subrectangular posterior fragment is uncertain due to very poor preservation. Through a series of repeated observations under different illumination settings, we were able to discern in part its overall shape. Its squarish anterior end (as preserved) matches the posterior end of the anterior fragment in shape and proportions. Assuming that the orientation of this posterior fragment approximates its actual anatomical position, its anterodorsal portion slightly overlaps the posteroventral end of the anterior fragment. Further posteriorly, the posterior fragment forms a strip of nearly constant width with parallel ventral and dorsal (left and right, respectively, as preserved) margins, and is in turn overlapped by the ventral portion of the squamosal. If our interpretation is correct, then the posterior fragment appears to widen dorsoventrally at its posterior extremity, which protrudes slightly behind the squamosal.

#### Sclerotic ring

At least ten, nearly undisrupted sclerotic plates are visible along the dorsal margin of the left orbit (Figs 2 and 11). The plates are subrectangular and overlap each other in a clockwise direction, each plate covering a shallow and smooth depression on the centripetal portion of the adjacent plate.

#### Palate

Various elements from the palate, braincase, and anterior vertebrae are visible posterior to the skull table. A portion of the quadrate ramus of one of the pterygoids, in the form of a featureless rectangular bony strip, is the only palatal element that can be identified with certainty (Fig 7), but it is not morphologically informative.

#### Ossifications of palatoquadrate and stapes

As preserved, the left quadrate lies immediately to the right of the posterodorsal margin of the squamosal and to the left of the angular. It is presumably exposed in posterior view (Figs 7 and 11) with its greater axis (direction of elongation of the ascending process) topographically aligned with the lateral margin of the left parietal lappet. The quadrate consists of a ventral condyle and an ascending process. Only the posterodorsal aspect of the condyle is visible. It has a subtriangular outline and overlies the glenoid surface of the articular, matching the latter in width. Although the condylar surface cannot be observed, the profile of its posterior margin (distal edge of the condylar region, as preserved) gives some indication of its proportions. Thus, the margin is divided into a longer and straight medial portion and a shorter and very slightly concave lateral portion. These two portions delimit a concave angle of ∼150 degrees between them, presumably indicating a size difference between the condyles. The lateral portion wraps around the lateral corner of the condylar region along a smooth curve. In contrast, the medial portion extends into a pointed medial ‘process’. The condyle merges indistinctly into the ascending process. The transition between these two regions is delimited by concave lateral and medial margins, the former being slightly shallower than the latter. The two margins are approximately parallel to each other for most of the length of the process and converge rapidly towards each other at its dorsal extremity. Further dorsally, the ascending process has subparallel margins with a vaguely sigmoid course. A large subcentral portion of the posterior surface of the process bears deep, subvertical, and slightly medially concave striations, probably for muscle insertion.

The right stapes is a large, squat element visible immediately posterior to the quadrate ramus of the pterygoid (Fig 7). Its footplate is broad but not greatly enlarged relative to the columella. The preserved portion of the columella is short and wide with a flat facet at its free extremity. The dorsal process forms a robust cylindrical structure. No other morphological details can be discerned.

#### Neural endocranium

The supraoccipital is the only recognizable element of the braincase. It is relatively small, about as wide as the postpineal portion of the parietal corpus, with a vaguely semicircular outline, as preserved (Fig 7). The dorsal rim for the foramen magnum is thick. A distinct but low, mid-longitudinal keel occurs dorsal to the foramen magnum. The dorsal surface of the supraoccipital is covered in small and irregular rugosities for muscle insertion. Adjacent to the dorsolateral margins of the bone are two oblique, elongate, and slightly recessed areas for the articulation with the skull table. The position and orientation of these areas suggest that the supraoccipital may have shown a gentle posteroventral slope in life.

#### Lower jaw

A partially preserved left lower jaw ramus, largely visible in internal view (Fig 11), occurs along the left-hand side of the skull table. Despite compaction, its general outline can be traced, and morphological details of various bones can be recognized in places. The anterior portion of the jaw is observed in medial view. It is easily identifiable by several anterior dentary teeth lying in proximity to the maxillary teeth. The posterior portion of the dentary is visible through the orbit and orientated slightly dorsomedially, as shown by the pattern of implantation of the marginal teeth (Figs 3 and 11). It tapers smoothly to a point posteriorly and appears only slightly displaced in relation to more posterior jaw elements. The rearmost tract of the dentary is toothless, and its length corresponds to that of the two most posterior teeth. However, it is impossible to ascertain whether the non-dentigerous portion of the bone extended further posteriorly and/or whether it overlapped the lateral surface of the surangular. Directly posterior to the dentary, and aligned with it, is a slender and subrectangular bony shard. Based upon its position and orientation, it is interpreted as a portion of the posterodorsal ramus of the posterior coronoid (Fig 11). It is appressed against most of the anterodorsal margin of the surangular and terminates a short distance below the dorsalmost point of the surangular crest. Preservation does not allow us to establish whether the coronoid simply overlaps the surangular or is firmly sutured with it, and whether it is partially exposed in lateral aspect. The adductor fossa is fully exposed in medial view (Figs 3 and 11). Its approximate anteroposterior extension and overall proportions can be established in part from the arrangement of the surrounding bones. The surangular is recognizable because of the prominent convexity of its dorsal margin. From the apex of the convexity (‘surangular crest’), the dorsal margin forms a gentle, longer posterodorsal slope and a steeper, shorter anterodorsal slope (Fig 11). The articular is a squarish element preserved in dorsal view (glenoid surface) immediately posterior to the quadrate. It has a round posterior profile and an unfinished and coarsely granular glenoid surface (Figs 7 and 11), but no other features are discernible. A large portion of the posterior part of the angular is exposed in internal view (Figs 7 and 11) and flanks the articular and quadrate. The observed portion of the medial surface of the angular is traversed by sharp ridges and irregular depressions of variable length and depth, arranged anteroposteriorly and approximately parallel to one another along a dorsal strip of the medial surface of the bone, and forming a radial pattern more ventrally. More anteriorly, the angular is heavily disrupted and the only preserved part consists of short, narrow bony splinters that overlap the anterolateral portion of the tabular and a small section of the posterior and middle portions of the parietal embayment for the supratemporal (Fig 7). A large bony fragment in the posterior portion of the lower jaw (Figs 3 and 11), visible slightly below the dorsal margin of the surangular, may belong to the prearticular. Its exposed surface (possibly lateral) carries a longitudinal, slightly protruding, and beveled ridge.

#### Dentition

All preserved marginal teeth are conical to peg-like in shape and vary greatly in size (Figs 2–5 and 11). In general, the larger teeth appear more conical while the smaller ones appear peg-like. Tooth implantation is typically pleurodont. A precise tooth count is not possible as both the dentary and the maxilla are partially covered by surrounding bones. In the anterior portion of the maxilla, it is possible to observe several small tooth sockets, with up to six small teeth estimated to have been present. Posterior to these small teeth, the median shelf of the maxilla thickens considerably, and five larger, conical teeth (“caniniform region”) are visible through the nasal (Figs 2 and 5 and 11). Although there is room for about 12 teeth in the preserved portion of the maxilla, it is estimated that only about half of the original maxillary teeth (actual teeth plus tooth positions) are observed. The more anterior dentary teeth are visible immediately lateral to the anterior portion of the left frontal (Figs 3 and 4 and 11). More posteriorly, a row of dentary teeth can be seen through the orbit (Figs 3 and 11).

#### Postcranial skeleton

Most of the axial skeleton, including the proximal portion of the tail (Fig 12A), is preserved in articulation. It has an estimated snout-vent length of ∼200 mm. Ribs are present throughout the column. Because the original description of *Coelostegus* provided a detailed account and reconstruction of the postcranial skeleton [3, 6], only brief comments are provided.

#### Vertebrae

In total, 43 vertebrae are preserved. Of these, 29 are presacral and include five which are identifiable as cervicals. Assuming the occurrence of two sacral vertebrae, we estimate that 12 caudal vertebrae are preserved, although the tail would have been much longer in life (Fig 12A). Albeit somewhat disarticulated, most elements of the atlas-axis complex are recognizable behind the skull table and their morphology appears to be typical of basal eureptiles (Figs 7 and 12A). The atlas complex consists of numerous unfused elements, most of which can be identified (Fig 12A). The largest element is the axis arch. The atlas pleurocentrum is a robust and horseshoe-shaped bone partly underlying the supraoccipital. One half of the atlas arch lies adjacent to the atlas pleurocentrum. A zygapophysis and the distinctive, posteriorly directed neural spine are also visible. The other half of the atlas arch may be preserved underneath its antimere, but its identification is dubious. A thin, oval bone wedged in the notochordal recess of the atlas pleurocentrum may represent the proatlas. A short distance away, the atlas intercentrum, possibly preserved in ventral view, underlies the stapes (Fig 7). The atlas pleurocentrum and the axis intercentrum are usually fused in early eureptiles [10, 20]. The unfused condition in *Coelostegus* may be unusual but may also simply reflect immaturity. Two of the three elements of the axis can be recognized. The axis arch, with its slightly enlarged neural spine, is present just posterior to the atlas arch and atlas pleurocentrum and is articulated with the rest of the spinal column. The axis pleurocentrum and the centra of the several following vertebrae appear to be missing, presumably due to preparation.

Posterior to the atlas and axis, the vertebrae are typically holospondylous, with blocky centra of similar length and width (Fig 12A). Minute intercentra, visible in several places along the trunk, are accommodated by the slightly beveled ventral edge of the following pleurocentrum. The neural arches are not fused to the centra. Some of the neural spines show rough, unfinished surfaces and evidence of slight displacement of their left and right halves, particularly in the posterior region of the axial skeleton, suggesting that they were not fully fused. The pedicles are sturdy but not swollen, and their area of attachment extends along the full length of the centrum. Small transverse facets for the rib articulation are visible on many of the arches, located low on the pedicle, and either adjacent to or overlapping the suture between the arch and the centrum. The last several presacral vertebrae have very reduced ribs and little or no evidence of a transverse process. The shape of the neural spines ranges from square to rounded in lateral aspect, although the margins are frequently indistinct and irregular. Their anteroposterior length is similar to that of the centra. In well-preserved regions of the vertebral column, the vertebral surface exhibits a finely pitted and “woven” texture. In general, there is very little morphological variation along the vertebral column, and regions can largely only be differentiated by their associated ribs (Fig 12A). The first sacral vertebra is distinguished by a much larger transverse facet than those of the posterior presacral vertebrae and is in articulation with the proximal portion of a sacral rib. The neural arches in the caudal region have smaller neural spines than the more anterior vertebrae, but preservation is generally poor in this area and as a result, this size reduction may be an artifact.

#### Ribs

Five cervical ribs are preserved, all carrying short, straight, and broad spatulate extremities (Fig 12A). The first two cervical ribs are very small, thin elements with a broad separation between the capitular and tubercular heads, and presumably belonged to the atlas and axis. The more posterior cervical ribs are larger, with wider blade-like extremities, and have only a slight indentation between the two heads. The thoracic ribs have small heads with a very small secondary articulation (Fig 12A). The first thoracic ribs (sixth pair) are somewhat shorter and straighter than the following thoracic ribs but are at least twice as long as the cervical ribs. The 7^th^ to 16^th^ pairs are long, slender, and curved. Posterior to this level, the ribs become shorter, and the last four (24^th^ to 27^th^) are extremely abbreviated. Two sacral ribs are preserved. The more anterior of the two is incomplete (Fig 12A) and occurs in articulation with the first sacral vertebra, while the more posterior rib is somewhat smaller and displaced posteriorly. It is short and stout, with a broad distal articulation, and distinct and well-spaced heads. The shape and proportions of the ilium make it unlikely that additional pairs of sacral ribs were present. The first four pairs of caudal ribs are relatively long (approximating the combined length of three centra) and strongly curved (Fig 12A). More posteriorly, the ribs become very short and straight vestiges, less than the length of a single centrum.

#### Pectoral girdle

The left clavicle is preserved lateral to the cervical ribs (Fig 12A). It has a flat, broad, and subtriangular blade, with striations running parallel to its greater axis and a thickened anterior edge. The flat, subtriangular clavicular process ends in an acuminate dorsal tip. The displaced cleithrum runs perpendicular to the clavicle and is visible between the clavicle and the vertebrae. It is shaped like a narrow rod, with a grooved posterior edge. The interclavicle underlies the other elements of the pectoral girdle, making its precise outline difficult to trace (Fig 12A). Its fimbriated anterior edge is visible just anterior to the clavicle. It is a smooth, very thin plate with a curved anterior margin and a few radiating striations. Smooth bony areas underlying the cervical vertebrae, extending as far back as the scapula, presumably represent portions of the interclavicle’s inner surface. A single scapular ossification is visible underlying the posterior end of the clavicle (Fig 12A). It has a simple, almost semi-circular shape, with a thickened supraglenoid buttress along the posterior margin. The upper portion of a small glenoid fossa is visible at its posterolateral corner. No coracoid ossification can be seen.

#### Forelimb

Very few details of the forelimb are available (Fig 12A). Part of the head of the presumed left humerus is visible adjacent to the scapulocoracoid. Along the right-hand side of the skeleton are two articulated phalanges, an ungual, and the next most distal phalanx, which were not previously described. The ungual is shorter than the proximal phalanx, about two-thirds of its length, and has a tapered, teardrop-like shape with a slight curvature at the tip.

#### Pelvic girdle

The elements of the pelvic girdle are unfused and slightly disrupted (Fig 12A). The right ilium is exposed in medial view. It is small but sturdy, and with a stout dorsal process that has room for two sacral ribs. About half of the presumed left pubis can be identified in the form of a featureless, flat bony sheet. The ischium is an elongate element with an unfinished ventral margin.

#### Hind limb

As with the forelimb, the information available for the hind limb is scanty, as only the proximal portion of a femur is visible (Fig 12A). The surface of the femur is finely textured with small, regular pits and a “woven” texture indicating an immature stage. The curvature of the femur, as well as the texture of its surface, indicate that the narrowest point of the shaft is preserved. Assuming this preserved portion is close to the shaft midpoint, the complete femur would have been about 20 mm long.

#### Scales

The ventral scales (osteoderms) are preserved in articulation and visible along most of the body length (Fig 12B). They are narrow and imbricated, about 3 mm long and 0.7 mm wide. Their external surface carries a subcentral longitudinal ridge. On either side of the ridge, the surface of the scale is smooth. The scales form posteriorly directed rows of chevrons meeting along the ventral midline and are displaced to the left of the vertebral column.

### Skull reconstruction

As reconstructed, the skull roof of *Coelostegus prothales* has a smooth parabolic profile in dorsal aspect, with a width (SW) to length (SL) ratio of ∼69% (SW = distance between the lateralmost points of the quadratojugals; SL = mid-sagittal distance between the anterior extremity of the inter-premaxillary suture and the posterior extremity of the inter-postparietal suture) and with a SL of ∼45mm. The skull table is abbreviated, with a length (STL) to width (STW) ratio of ∼66% (STW = distance between the lateral points of the tabulars; STL = mid-sagittal distance between the anterior extremity of the inter-parietal suture and the posterior extremity of inter-postparietal suture). The length of the orbits (measured para-sagittally) is ∼30% of SL. Their mid-point occurs approximately halfway between the anterior extremity of the inter-premaxillary suture and the lateral projections of the quadratojugals. The external nostrils are proportionally much larger and more closely spaced than in the original reconstruction, and the pineal foramen has a distinct subelliptical outline.

Our reconstruction differs from Carroll and Baird’s [3] in several details. Anterior and posterior to the triple sutural joints between the nasals, prefrontals, and lacrimals, the nasals appear broader and more abbreviated than in the original reconstruction and reveal distinctly more concave sutural margins with the prefrontals. The frontals are shorter and wider than in Carroll and Baird’s illustration, and their contributions to the orbit margins are slightly smaller. Their posterolateral processes are noticeably longer and narrower and terminate in a more acuminate extremity situated only slightly posterior to the posterior margins of the orbits. Excluding such processes, the frontal-parietal suture is situated well in front of the posterior margins of the orbits. The parietal lappets are considerably wider and shorter than in Carroll and Baird’s reconstruction and their posterolateral extremities are approximately aligned with the posterior margins of the postparietals. The lappets do not project into robust posterolateral processes. Other minor differences between Carroll and Baird’s reconstruction and our own relate to the shape and proportions of the postfrontals and maxillae. Thus, the morphology of the postfrontal indicates that its posteromedial process is short, broad, and lappet-like and extends only slightly behind the extremity of the posterolateral process of the frontal. Finally, the maxilla is less deep than in the original illustration, and its dorsal margin has a low and smoothly convex profile anteriorly.

### Phylogenetic results

The maximum parsimony analysis of the Ford and Benson data matrix with all characters receiving equal unit weights yields 32 trees at 1587 steps (C.I. = 0.2319; R.I. = 0.5903; all characters are informative). In the strict consensus tree (Fig 14A), *Romeria*, *Reiszorhinus*, *Protocaptorhinus*, *Captorhinus*, *Labidosaurikos*, and *Labidosaurus* form a clade. This clade is placed in a tetrachotomy with *Thuringothyris*, *Euconcordia*, and all other non-synapsid amniotes. Protorothyridids are paraphyletic, with *Coelostegus* as the sister taxon to diapsids. All the remaining protorothyridids emerge as the sister group to the *Coelostegus*-diapsid clade, and with *Hylonomus* and *Anthracodromeus* collapsed in a trichotomy with (*Paleothyris* + *Protorothyris*). The agreement subtree obtained from the 32 shortest trees is shown in Fig 14B. In the 50% bootstrap and jackknife majority-rule consensus topologies (S1 and S2 Figs), protorothyridids (as a clade) receive very low support (2%) and their placement crownward of captorhinids and as sister group to all other taxa (araeoscelidians plus crown amniotes) is similarly tenuous (4%).

When characters are reweighted by the greatest value of their rescaled consistency index, PAUP* produces a single tree (Fig 14C) in which monophyletic captorhinids and paraphyletic protorothyridids are successively more closely related to diapsids. In this tree, *Anthracodromeus* and *Coelostegus* (as sister taxa) are the most plesiomorphic of all protorothyridids. *Hylonomus*, *Paleothyris*, and *Protorothyris* form a paraphyletic array, in that order, relative to diapsids. In the single tree from the implied weights analysis (Fig 14D), protorothyridids are once again paraphyletic. Among them, *Coelostegus* is the most plesiomorphic genus, followed by a clade formed by (*Hylonomus* + (*Anthracodromeus* + (*Paleothyris* + *Protorothyris*))).

The Bayesian analysis of the Ford and Benson data matrix (S3 Fig) retrieves *Coelostegus*, *Hylonomus*, *Anthracodromeus*, *Paleothyris*, and *Protorothyris*, in that order, as a paraphyletic array relative to neoreptiles. The node subtending *Coelostegus* and all more derived taxa has a low posterior probability (37%), which underscores Müller and Reisz’s [6] original observations about the relative instability of this taxon.

**Fig 15:**
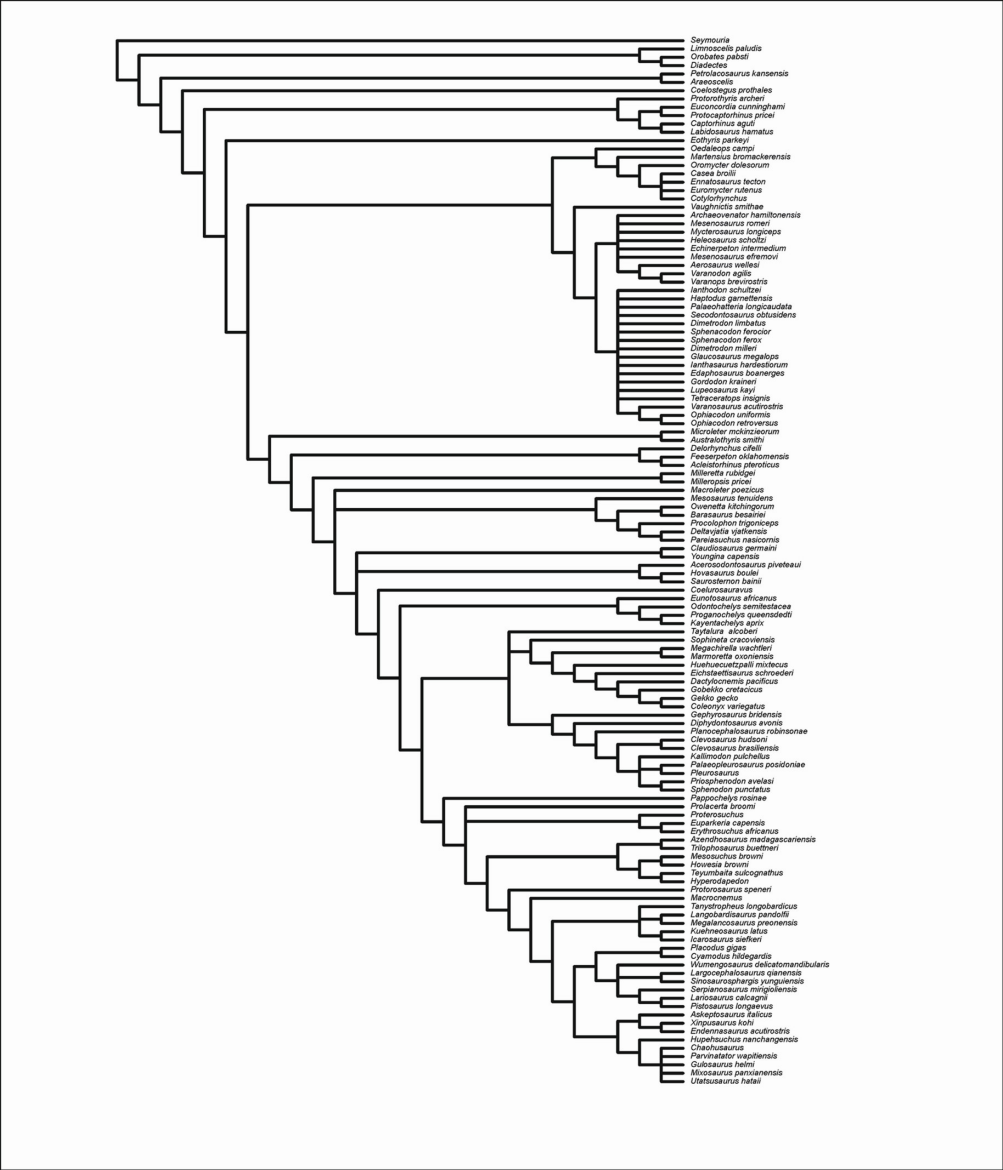
Interrelationships of *Coelostegus prothales*. Strict consensus of 3·10^5^ shortest trees from a maximum parsimony analysis of Simöes et al.’s [8] dataset with all characters having equal unit weight.

The 3·10^5^ trees obtained from the parsimony analysis of the Simöes et al. [8] matrix (equally weighted characters) are 1717 steps long (C.I. = 0.2132, excluding 24 characters that are uninformative; R.I. = 0.6799). In the strict consensus of those trees, *Coelostegus* is placed on the amniote stem, immediately crownward of araeoscelidians and anticrownward of the *Protorothyris*-captorhinid clade (Fig 15). In the 50% bootstrap and jackknife majority-rule consensus topologies (S4 and S5 Figs), the *Protorothyris*-captorhinid clade, *Coelostegus*, and araeoscelidians are arranged on the amniote stem in the reverse order in which they appear in the strict consensus. Support for the ingroup is only moderate (61% bootstrap; 59% jackknife), while the placement of *Coelostegus* is invariably weak (6%), as is the support for the araeoscelidian-crown amniote clade (4%).

## Discussion

### General remarks

Photogrammetric scanning has revealed cranial elements that were not identified in the original description of *Coelostegus* [3], including the premaxilla, postorbital, supratemporal, tabular, postparietal, and angular. The supraoccipital is more clearly discernible and its outline is better defined than previous illustrations suggest [3]. In the case of the postcranial skeleton, the axis intercentrum and phalanges are newly recognized, and the ventral scales are shown in full. In addition, photogrammetry has enabled a detailed account of the morphology of individual skeletal elements. Despite this, however, certain bones identified by Carroll and Baird [3] appear indeterminate to us (e.g., the portion of the left exoccipital adjacent to the tabular).

As noted by Carroll and Baird [3], several skeletal features suggest immaturity, such as the unconsolidated atlas-axis complex, the weakly ossified scapulocoracoid, vertebrae, and pelvis, and the woven texture observed on several vertebrae and on the femoral surface. If the holotype of *Coelostegus* did belong to an immature individual, then this animal, as an adult, might have been one of the largest eureptiles of its time.

### Cranial comparisons with other taxa

It is instructive to compare the skull morphology of *Coelostegus* to that of other protorothyridids. Most taxa in this group need comprehensive revision. Pending their full reassessment, therefore, the comparisons expounded below should be considered to be preliminary. The skull of *Coelostegus prothales* is broadly similar to that of other basal reptiles from the Pennsylvanian [1, 3], but it is also distinctly larger. Its size is striking, especially considering that the specimen may belong to an immature individual. The most distinctive feature of the skull of *Coelostegus* is the deep posterior embayment of the parietals, which is absent in all other Pennsylvanian protorothyridids (*Paleothyris*, *Hylonomus*, *Anthracodromeus*, and *Cephalerpeton)* [1, 3]. A similar embayment occurs in the Lower Permian *Romeria* (a captorhinid) and in *Protorothyris* [4]. However, unlike in those two taxa, the embayment of *Coelostegus* is not bordered by distinct posteromedian extensions of the parietals.

The second protorothyridid described from Nýřany, *Brouffia orientalis* [3], is very similar to *Coelostegus* in several respects, albeit smaller. A revised description of *Brouffia* is part of ongoing research by us. However, a few remarks are warranted. Despite overall similarities between *Coelostegus* and *Brouffia*, these two genera can be distinguished by subtle details of numerous cranial bones. For ease of exposition, the following comparisons highlight the condition of *Coelostegus* first. The squamosals of *Coelostegus* are comparatively deeper and longer than those of *Brouffia*, and project well behind the posterior margin of the skull table. The conjoined parietals are distinctly wider than long and show a more pronounced separation between the corpus and the lateral lappets. The frontals are stouter and with a comparatively slightly larger contribution to the orbit margins. In addition, the posterolateral processes of the frontals are proportionally longer in *Coelostegus* than in *Brouffia*, taper sharply to a point, and are deeply wedged between the parietals and the postfrontals. Finally, the frontal-parietal sutures of *Coelostegus* occur well in front of the posterior margins of the orbits.

*Coelostegus* differs from *Anthracodromeus* in the morphology of the parietals. In *Coelostegus*, the medial part of the posterior margin of the parietal is gently concave. In *Anthracodromeus*, this margin projects strongly posteriorly and as a result, the conjoined parietals form a posteriorly convex, posterior lappet. Furthermore, the lateral lappets are comparatively shorter in *Anthracodromeus* than in *Coelostegus* and less well delimited from the parietal corpus. Lastly, the pineal foramen of *Anthracodromeus* is only slightly anterior to the midpoint of the inter-parietal suture.

*Coelostegus* differs from *Cephalerpeton* in possessing comparatively wider nasals and frontals, the former slightly exceeding the latter in length. As restored [3], the skull of *Cephalerpeton* features wide parietals which extend slightly lateral to the lateralmost points of the orbit margins. Immediately in front of the orbit, the prefrontal and lacrimal of *Cephalerpeton* show a distinct interlocking pattern, whereby the posterior one-third of the dorsal margin of the lacrimal produces a broad process fitting into a wide recess of the prefrontal [3, 21]. The process is narrowly separated from the orbit margin by a slender and short posteroventrolateral process of the prefrontal. *Coelostegus* shows a similar process, but this appears less pronounced and smaller than in *Cephalerpton* and is not separated from the orbit margin by an intervening process of the prefrontal.

*Coelostegus* differs from *Protorothyris* in the general proportions of the skull and in the morphology of various cranial elements. The orbits of *Coelostegus* are shorter than those of *Protorothyris* in relation to total skull length, its external nostrils are larger, and the pineal foramen is distinctly wider than long. Unlike in *Coelostegus*, the frontals of *Protorothyris* are longer than the nasals and narrow abruptly in the anterior one-fifth of their length, so that they appear wedged between the broadly triangular posterolateral processes of the nasals. The deeply concave posterior margins of the parietals of *Protorothyris* delimit a narrow, triangular posterior projection of the skull table and contribute to the formation of stout posterolateral processes that accommodate the supratemporals. The postfrontals of *Protorothyris* show broader, more smoothly convex posteromedial lappets than those of *Coelostegus*. Also, the parietals of *Protorothyris* are not deeply wedged between the frontals, the frontals lack posterolateral processes, and the frontal-parietal suture lies approximately at the level of the posterior margins of the orbits. The parietal lappets of *Protorothyris*, while conspicuous, are longer than wide and less distinctly separated from the parietal corpus than their homologues in *Coelostegus*. As restored, the squamosals of *Protorothyris* are squarish in lateral aspect and less expanded anteriorly than those of *Coelostegus*. The suborbital ramus of the jugal is deep and borders nearly the entire ventral orbit margin in *Protorothyris*. In *Coelostegus*, the ramus is slender and terminates slightly anterior to the midpoint of the orbit length. The dorsal margin of the maxilla of *Protorothyris* appears irregular, in contrast to the smooth profile of the maxilla of *Coelostegus*. Lastly, in *Protorothyris* the lacrimal features a narrow, triangular dorsal process immediately in front of the orbit, similar to that reported in *Cephalerpeton* and *Coelostegus*.

*Coelostegus* differs from *Paleothyris* primarily in the proportions of the mid skull roof bones. As restored [3], *Paleothyris* has comparatively shorter and narrower nasals, very elongate and strap-shaped frontals, and subtrapezoidal parietals, the latter forming a poorly pronounced and posteriorly protruding midline ‘peak’. No distinct lateral lappets are present. In addition, the postfrontals contact the anterolateral margins of the parietals along a smoothly curved suture, but without forming posteromedial lappets. The distance between the posterior margin of the orbits and the anteriormost point of the squamosal is distinctly shorter in *Paleothyris* than in *Coelostegus*, and the lacrimal contribution to the orbit extends dorsal to the mid-height of the anterior orbit margin in the former.

Comparisons between *Coelostegus* and *Hylonomus* are necessarily limited, due to the incomplete nature of the latter [5]. Compared to *Coelostegus*, *Hylonomus* shows a greater contribution of the frontal to the orbit margin, a poorly pronounced posterolateral lappet of the postfrontal, no lateral lappets of the parietals, and a strap-like lacrimal that increases smoothly in depth anteroposteriorly.

### Comments on the affinities of *Coelostegus*

The results obtained from various treatments of Ford and Benson’s [7] and Simöes et al.’s [8] datasets do not provide unequivocal evidence for the affinities of *Coelostegus*. As in Müller and Reisz’s [6] study, the position of this tetrapod is weakly supported, regardless of its placement in various analyses. This uncertainty reflects the mosaic of plesiomorphic and apomorphic characteristics of *Coelostegus*, the uncertain polarity of several traits near the roots of crown amniotes, the amount of unknown character states, as well as the use of non-overlapping character-taxon sets in different studies. In this context, it is instructive to note that a number of skull features observed in several protorothyridids also occur within diadectomorphs, albeit in a modified form, and are also variably expressed in several early diverging crown amniotes. For instance, many diadectomorphs, including *Limnoscelis*, *Tseajaia*, and *Diadectes*, show a greatly expanded lateral lappet of the parietal, a variably developed posteromedial process of the postfrontal that overlaps the parietal, and a recess on the posterolateral corner of the parietal that receives the supratemporal [22, 23].

In the parsimony analysis of the Ford and Benson [7] matrix with equally weighted characters, the placement of *Coelostegus* as the sister taxon to Diapsida is supported solely by homoplasies. Using the first of the 32 most parsimonious trees as an example, the *Coelostegus*-diapsid clade is supported by 15 homoplastic state changes, three of which are unambiguous (that is, they occur under both the accelerated and the delayed optimization of state changes) and four of which represent reversals. Similarly, in the first of the 3·10^5^ trees obtained from the equally weighted parsimony analysis of the Simöes et al. [8] matrix, the grouping of *Coelostegus* with *Protorothyris*-captorhinids-crown amniotes is supported by homoplastic state changes (seven in total), two of which are unambiguous and four of which represent reversals.

Lastly, as a preliminary approach to evaluating the effects of non-overlapping taxon datasets, we ran two additional, equally weighted parsimony analyses, one for each of the Ford and Benson [7] and Simöes et al. [8] datasets, using only genera common to both and eliminating remaining taxa. If genera consisted of more than one species in either matrix, then all species were included. The strict consensus of the 206 shortest trees obtained after the re-analysis of the Ford and Benson [7] matrix (41 out of 71 taxa) is poorly resolved, with 15 taxa and clades collapsed in a large polytomy. In particular, we found no support for the monophyly of synapsids (S6A Fig). The strict consensus of the 27 shortest trees yielded by the re-analysis of the Simöes et al. [8] matrix (44 out of 126 taxa) is mostly well-resolved, except for some polytomies within synapsids (S6B Fig). In the strict consensus, a clade of *Protorothyris* plus araeoscelidians, captorhinids, and *Coelostegus* are increasingly more closely related, in that order, to neoreptiles.

A core objective of future investigations into amniote interrelationships will be the construction of new, large-scale phylogenies from cross-referenced, combined, and leveraged datasets, and with new data aiming at filling existing gaps (e.g., variation in skeletal structures that are under-represented in published data matrices). It is hoped that the present revision of *Coelostegus prothales* will offer researchers an opportunity to examine in greater detail character signal and noise near the roots of an important animal clade.

## Supporting information

S1 Appendix Ford and Benson’s [7] data matrix. PAUP-readable nexus format after inclusion of *Coelostegus* and addition of character 295 (see main text for details).
(TXT)

S2 Appendix Simöes et al.’s [8] data matrix. PAUP-readable nexus format after inclusion of *Coelostegus*.
(TXT)

S3 Appendix Ford and Benson’s [7] dataset for Bayesian analysis. MrBayes-readable script for running non-calibrated Bayesian analysis after inclusion of *Coelostegus*.
(TXT)

S4 Appendix Results of Bayesian analysis. Output of MrBayes analysis of Ford and Benson’s [7] dataset (see S3 Appendix).
(RTF)

S1 Fig Interrelationships of *Coelostegus prothales*. Bootstrap 50% majority-rule consensus from Ford and Benson’s [7] dataset, with bootstrap percentage support appended to branches.
(TIF)

S2 Fig Interrelationships of *Coelostegus prothales*. Jackknife 50% majority-rule consensus from Ford and Benson’s [7] dataset, with jackknife percentage support appended to branches.
(TIF)

S3 Fig Interrelationships of *Coelostegus prothales*. Bayesian 50% majority-rule consensus from Ford and Benson’s [7] dataset, including groups compatible with consensus, and with Bayesian posterior probabilities appended to branches.
(TIF)

S4 Fig Interrelationships of *Coelostegus prothales*. Bootstrap 50% majority-rule consensus from Simoes et al.’s [8] dataset, with bootstrap percentage support appended to branches.
(TIF)

S5 Fig Interrelationships of *Coelostegus prothales*. Jackknife 50% majority-rule consensus from Simoes et al.’s [8] dataset, with jackknife percentage support appended to branches.
(TIF)

S6 Fig Interrelationships of *Coelostegus prothales*. **A**. Strict consensus of the 206 shortest trees from a maximum parsimony analysis of the taxonomically pruned version of Ford and Benson’s [7] dataset, using only taxa in common with the Simoes et al.’s [8] dataset, and with all characters having equal unit weight. **B**. Strict consensus of the 27 shortest trees from a maximum parsimony analysis of the taxonomically pruned version of the Simoes et al.’s [8] dataset, using only taxa in common with the Ford and Benson’s [7] dataset, and with all characters having equal unit weight.
(ZIP)
