# Supplementary material for: A review of Coelostegus prothales Carroll and Baird, 1972 from the Upper Carboniferous of the Czech Republic and the interrelationships of basal eureptiles
Source: PLoS One. 2023 Sep 21;18(9):e0291687. doi: 10.1371/journal.pone.0291687 (PMC10513281; doi:10.1371/journal.pone.0291687)
Supplement: S4 Appendix — Output of MrBayes analysis of Ford and Benson’s [7] dataset (see S3 Appendix). (RTF) [file pone.0291687.s004.rtf]

Last login: Thu Jul 13 14:34:44 on ttys000Marcellos-MacBook-Pro:~ marcelloruta$ cd /Applications/MrBayesMarcellos-MacBook-Pro:MrBayes marcelloruta$ /Applications/MrBayes/mb                             MrBayes v3.2.6 x64                      (Bayesian Analysis of Phylogeny)              Distributed under the GNU General Public License               Type "help" or "help <command>" for information                     on the commands that are available.                   Type "about" for authorship and general                       information about the program.MrBayes > execute CoelostegusBayes.txt   Executing file "CoelostegusBayes.txt"   UNIX line termination   Longest line length = 351   Parsing file   Expecting NEXUS formatted file   Reading data block      Allocated taxon set      Allocated matrix      Defining new matrix with 71 taxa and 295 characters      Data is Standard      Gaps coded as -      Missing data coded as ?      WARNING: MrBayes does not support 'symbols' specification; default symbols assumed      Taxon  1 -> Gephyrostegus_bohemicus      Taxon  2 -> Seymouria_spp      Taxon  3 -> Tseajaia_campi      Taxon  4 -> Limnoscelis_paludis      Taxon  5 -> Oedaleops_campi      Taxon  6 -> Eothyris_parkeyi      Taxon  7 -> Vaughnictis_smithae      Taxon  8 -> Eocasea_martini      Taxon  9 -> Euromycter_rutena      Taxon 10 -> Casea_broilii      Taxon 11 -> Ennatosaurus_tecton      Taxon 12 -> Echinerpeton_intermedium      Taxon 13 -> Archaeothyris_florensis      Taxon 14 -> Varanosaurus_acutirostris      Taxon 15 -> Ophiacodon_spp      Taxon 16 -> Cutleria_wilmarthi      Taxon 17 -> Pantelosaurus_saxonicus      Taxon 18 -> Haptodus_garnettensis      Taxon 19 -> Secodontosaurus_obtusidens      Taxon 20 -> Dimetrodon_spp      Taxon 21 -> Ianthasaurus_hardestiorum      Taxon 22 -> Edaphosaurus_boanerges      Taxon 23 -> Hylonomus_lyelli      Taxon 24 -> Anthracodromeus_longipes      Taxon 25 -> Paleothyris_acadiana      Taxon 26 -> Protorothyris_archeri      Taxon 27 -> Petrolacosaurus_kansensis      Taxon 28 -> Araeoscelis_spp      Taxon 29 -> Orovenator_mayorum      Taxon 30 -> Archaeovenator_hamiltonensis      Taxon 31 -> Ascendonanus_nestleri      Taxon 32 -> Aerosaurus_wellesi      Taxon 33 -> Apsisaurus_witteri      Taxon 34 -> Heleosaurus_scholtzi      Taxon 35 -> Mesenosaurus_romeri      Taxon 36 -> Mycterosaurus_longiceps      Taxon 37 -> Elliotsmithia_longiceps      Taxon 38 -> Varanops_brevirostris      Taxon 39 -> Varanodon_agilis      Taxon 40 -> Watongia_meieri      Taxon 41 -> Youngina_capensis      Taxon 42 -> Acerosodontosaurus_piveteaui      Taxon 43 -> Lanthanolania_ivakhnenkoi      Taxon 44 -> Claudiosaurus_germaini      Taxon 45 -> Prolacerta_broomi      Taxon 46 -> Proterosuchus_spp      Taxon 47 -> Thuringothyris_mahlendorffae      Taxon 48 -> Captorhinus_aguti      Taxon 49 -> Captorhinus_laticeps      Taxon 50 -> Labidosaurikos_meachami      Taxon 51 -> Labidosaurus_hamatus      Taxon 52 -> Protocaptorhinus_pricei      Taxon 53 -> Romeria_spp      Taxon 54 -> Euconcordia_cunninghami      Taxon 55 -> Reiszorhinus_olsoni      Taxon 56 -> Eudibamus_cursoris      Taxon 57 -> Mesosaurus_tenuidens      Taxon 58 -> Stereosternum_tumidum      Taxon 59 -> Erpetonyx_arsenaultorum      Taxon 60 -> Belebey_vegrandis      Taxon 61 -> Macroleter_poezicus      Taxon 62 -> Milleretta_rubidgei      Taxon 63 -> Acleistorhinus_pteroticus      Taxon 64 -> Colobomycter_pholeter      Taxon 65 -> Nyctiphruretus_acudens      Taxon 66 -> Emeroleter_levis      Taxon 67 -> Deltavjatia_rossicus      Taxon 68 -> Procolophon_trigoniceps      Taxon 69 -> Candelaria_barbouri      Taxon 70 -> Owenetta_kitchingorum      Taxon 71 -> Coelostegus_prothales      Successfully read matrix      Matrix contains polymorphisms, interpreted as ambiguity      Setting default partition (does not divide up characters)      Setting model defaults      Seed (for generating default start values) = 1689256826      Setting output file names to "CoelostegusBayes.txt.run<i>.<p|t>"   Reading mrbayes block      Logging screen output to file "with_Coelostegus_Mkv.log"      Setting Rates to Gamma      Enabling Coding Variable      Successfully set likelihood model parameters      Setting outgroup to taxon "Gephyrostegus_bohemicus"      Defining constraint called 'ingroup1'      Defining constraint called 'ingroup2'      Setting Topologypr to Constraints      Successfully set prior model parameters      Setting number of runs to 2      Setting number of chains to 4      Setting heating parameter to 0.100000      Setting number of generations to 10000000      Setting sample frequency to 1000      Setting print frequency to 1000      Setting diagnosing frequency to 5000      Using relative burnin (a fraction of samples discarded).      Setting burnin fraction to 0.25      Setting chain output file names to "CoelostegusBayes.txt.run<i>.<p/t>"      Successfully set chain parameters      Running Markov chain      MCMC stamp = 7420410946      Seed = 18442760      Swapseed = 1689256826      Model settings:         Data not partitioned --            Datatype  = Standard            Coding    = Variable            # States  = Variable, up to 10                        State frequencies are fixed to be equal            Rates     = Gamma                        The distribution is approximated using 4 categories.                        Likelihood summarized over all rate categories in each generation.                        Shape parameter is exponentially                        distributed with parameter (1.00).      Active parameters:          Parameters         ---------------------         Statefreq           1         Shape               2         Ratemultiplier      3         Topology            4         Brlens              5         ---------------------         1 --  Parameter  = Alpha_symdir               Type       = Symmetric diricihlet/beta distribution alpha_i parameter               Prior      = Symmetric dirichlet with all parameters equal to infinity         2 --  Parameter  = Alpha               Type       = Shape of scaled gamma distribution of site rates               Prior      = Exponential(1.00)         3 --  Parameter  = Ratemultiplier               Type       = Partition-specific rate multiplier               Prior      = Fixed(1.0)         4 --  Parameter  = Tau               Type       = Topology               Prior      = Prior on topologies obeys constraints               Subparam.  = V         5 --  Parameter  = V               Type       = Branch lengths               Prior      = Unconstrained:GammaDir(1.0,0.1000,1.0,1.0)      The MCMC sampler will use the following moves:         With prob.  Chain will use move            1.96 %   Multiplier(Alpha)            9.80 %   ExtSPR(Tau,V)            9.80 %   ExtTBR(Tau,V)            9.80 %   NNI(Tau,V)            9.80 %   ParsSPR(Tau,V)           39.22 %   Multiplier(V)           13.73 %   Nodeslider(V)            5.88 %   TLMultiplier(V)      Division 1 has 297 unique site patterns      Initializing conditional likelihoods      Using standard non-SSE likelihood calculator for division 1 (single-precision)      Initial log likelihoods and log prior probs for run 1:         Chain 1 -- -9787.797764 -- 134.258872         Chain 2 -- -9922.017077 -- 134.258872         Chain 3 -- -9913.326494 -- 134.258872         Chain 4 -- -9982.955028 -- 134.258872      Initial log likelihoods and log prior probs for run 2:         Chain 1 -- -10087.167251 -- 134.258872         Chain 2 -- -9854.582582 -- 134.258872         Chain 3 -- -9946.022647 -- 134.258872         Chain 4 -- -9921.715989 -- 134.258872      Using a relative burnin of 25.0 % for diagnostics      Chain results (10000000 generations requested):          0 -- [-9787.798] (-9922.017) (-9913.326) (-9982.955) * [-10087.167] (-9854.583) (-9946.023) (-9921.716)        1000 -- (-7234.818) (-6991.727) [-6779.556] (-7195.031) * (-7116.660) (-6980.684) (-7436.913) [-6860.462] -- 11:06:36       2000 -- (-6799.584) (-6537.531) [-6370.015] (-6576.558) * (-6556.327) (-6581.138) (-6792.940) [-6395.190] -- 11:06:32       3000 -- (-6439.825) (-6314.113) [-6231.650] (-6237.976) * (-6276.042) [-6152.635] (-6471.818) (-6157.828) -- 11:06:28       4000 -- (-6241.955) (-6202.990) (-6152.986) [-6094.139] * (-6152.160) [-6016.731] (-6236.497) (-6051.539) -- 11:48:03       5000 -- (-6126.175) (-6010.650) (-6082.169) [-5978.954] * (-6091.156) [-5987.596] (-6169.033) (-6001.912) -- 11:39:39      Average standard deviation of split frequencies: 0.173139       6000 -- (-6058.196) (-5937.399) (-6036.740) [-5917.487] * (-5995.115) [-5941.976] (-6092.482) (-5990.603) -- 11:34:01       7000 -- (-6015.630) (-5897.993) (-5976.847) [-5897.048] * [-5866.637] (-5914.513) (-6055.163) (-5958.544) -- 11:29:59       8000 -- (-6007.229) (-5899.236) (-5928.277) [-5867.463] * [-5859.099] (-5901.232) (-6013.387) (-5968.785) -- 11:26:57       9000 -- (-5964.426) (-5898.991) (-5876.718) [-5856.505] * [-5839.746] (-5903.256) (-5976.415) (-5954.075) -- 11:24:34      10000 -- (-5909.545) (-5903.870) (-5857.864) [-5851.631] * [-5832.199] (-5894.609) (-5980.186) (-5932.474) -- 11:39:18      Average standard deviation of split frequencies: 0.117950      11000 -- (-5889.403) (-5900.447) (-5877.062) [-5878.643] * [-5822.217] (-5903.982) (-5981.226) (-5927.114) -- 11:36:12      12000 -- [-5848.502] (-5878.090) (-5867.511) (-5873.623) * [-5825.492] (-5880.680) (-5940.129) (-5957.221) -- 11:33:36      13000 -- (-5852.863) [-5842.711] (-5878.851) (-5878.213) * [-5823.060] (-5870.243) (-5915.847) (-5940.236) -- 11:44:12      14000 -- (-5852.096) (-5834.505) [-5856.567] (-5897.299) * [-5828.415] (-5876.152) (-5887.727) (-5951.348) -- 11:41:23      15000 -- (-5858.717) [-5827.846] (-5861.506) (-5889.557) * [-5827.162] (-5876.570) (-5862.218) (-5963.686) -- 11:38:57      Average standard deviation of split frequencies: 0.141494      16000 -- [-5837.752] (-5828.478) (-5862.982) (-5866.611) * [-5831.892] (-5867.712) (-5863.536) (-5959.402) -- 11:36:48      17000 -- (-5849.265) [-5829.037] (-5856.396) (-5888.505) * [-5838.988] (-5864.133) (-5857.140) (-5926.160) -- 11:44:40      18000 -- (-5863.365) [-5816.329] (-5842.656) (-5896.315) * [-5822.869] (-5876.385) (-5836.842) (-5937.897) -- 11:42:26      19000 -- (-5852.491) [-5807.073] (-5872.072) (-5864.577) * (-5828.176) (-5876.649) [-5838.012] (-5929.329) -- 11:40:25      20000 -- (-5862.739) [-5828.786] (-5872.902) (-5861.717) * (-5836.742) (-5881.883) [-5844.635] (-5905.602) -- 11:38:36      Average standard deviation of split frequencies: 0.111331      21000 -- (-5845.204) [-5820.722] (-5866.800) (-5862.118) * (-5838.241) (-5886.095) [-5837.197] (-5914.466) -- 11:36:56      22000 -- [-5853.140] (-5840.822) (-5875.053) (-5877.802) * (-5833.686) (-5866.880) [-5847.167] (-5896.847) -- 11:35:26      23000 -- (-5867.978) [-5845.082] (-5844.154) (-5854.256) * [-5827.420] (-5846.688) (-5847.217) (-5916.411) -- 11:34:03      24000 -- (-5853.691) [-5836.677] (-5851.629) (-5883.045) * [-5828.759] (-5841.846) (-5850.743) (-5888.388) -- 11:39:42      25000 -- (-5852.198) [-5846.447] (-5852.784) (-5859.835) * (-5845.060) [-5833.822] (-5832.558) (-5904.893) -- 11:38:15      Average standard deviation of split frequencies: 0.095118      26000 -- (-5841.835) [-5820.921] (-5873.408) (-5862.236) * (-5844.229) [-5842.999] (-5831.148) (-5914.001) -- 11:36:54      27000 -- (-5830.718) [-5830.165] (-5842.366) (-5883.810) * (-5851.752) (-5841.767) [-5815.873] (-5885.940) -- 11:35:38      28000 -- (-5833.158) [-5827.976] (-5873.112) (-5878.621) * (-5840.635) (-5851.747) [-5830.925] (-5911.221) -- 11:34:28      29000 -- (-5842.055) [-5817.094] (-5853.924) (-5857.653) * [-5825.586] (-5841.222) (-5845.443) (-5892.451) -- 11:33:23      30000 -- (-5850.548) [-5817.440] (-5864.909) (-5855.788) * [-5831.102] (-5842.295) (-5847.761) (-5896.258) -- 11:37:54      Average standard deviation of split frequencies: 0.095560      31000 -- (-5853.589) [-5825.239] (-5865.719) (-5856.023) * [-5821.311] (-5847.059) (-5857.069) (-5898.509) -- 11:36:45      32000 -- (-5865.381) [-5837.171] (-5857.334) (-5851.227) * (-5828.244) (-5863.199) [-5845.826] (-5890.107) -- 11:35:41      33000 -- (-5851.137) [-5827.599] (-5871.161) (-5858.694) * [-5836.431] (-5845.879) (-5843.355) (-5912.467) -- 11:34:40      34000 -- (-5849.485) [-5833.312] (-5852.468) (-5852.875) * [-5829.392] (-5841.614) (-5858.662) (-5909.077) -- 11:33:42      35000 -- (-5837.037) [-5843.474] (-5846.065) (-5875.577) * [-5820.030] (-5857.576) (-5852.254) (-5897.835) -- 11:32:48      Average standard deviation of split frequencies: 0.085664      36000 -- [-5831.927] (-5829.678) (-5839.151) (-5865.672) * [-5824.911] (-5849.996) (-5842.966) (-5894.394) -- 11:36:33      37000 -- [-5833.445] (-5833.310) (-5852.052) (-5859.066) * (-5827.190) (-5843.665) [-5831.394] (-5896.663) -- 11:35:36      38000 -- (-5847.000) [-5811.103] (-5860.155) (-5848.361) * (-5838.863) (-5839.269) [-5822.288] (-5937.020) -- 11:34:43      39000 -- (-5835.540) [-5823.010] (-5846.033) (-5859.040) * (-5824.084) (-5842.473) [-5822.200] (-5926.548) -- 11:33:51      40000 -- [-5838.357] (-5841.551) (-5885.107) (-5854.915) * (-5857.587) (-5837.548) [-5833.828] (-5894.372) -- 11:37:12      Average standard deviation of split frequencies: 0.083688      41000 -- [-5832.347] (-5853.206) (-5873.397) (-5881.380) * (-5848.143) (-5840.088) [-5843.333] (-5912.168) -- 11:36:19      42000 -- (-5842.930) [-5821.944] (-5866.302) (-5857.005) * (-5849.555) (-5857.066) [-5831.362] (-5913.138) -- 11:35:28      43000 -- (-5834.360) [-5831.756] (-5861.256) (-5878.652) * [-5850.605] (-5844.637) (-5847.492) (-5899.286) -- 11:34:40      44000 -- (-5862.738) [-5827.183] (-5885.795) (-5861.521) * (-5842.681) [-5834.501] (-5843.879) (-5904.411) -- 11:33:54      45000 -- (-5853.462) (-5830.747) (-5866.358) [-5838.775] * [-5838.063] (-5834.286) (-5831.845) (-5902.781) -- 11:33:09      Average standard deviation of split frequencies: 0.078188      46000 -- (-5851.267) [-5828.817] (-5865.623) (-5840.456) * [-5820.443] (-5841.601) (-5828.568) (-5910.631) -- 11:32:27      47000 -- [-5836.784] (-5861.200) (-5868.699) (-5838.857) * [-5839.341] (-5863.485) (-5819.229) (-5911.432) -- 11:35:17      48000 -- [-5837.955] (-5853.150) (-5865.890) (-5847.127) * (-5848.476) (-5879.737) [-5847.512] (-5901.028) -- 11:34:34      49000 -- (-5826.688) [-5826.373] (-5879.623) (-5856.397) * [-5831.209] (-5859.899) (-5831.426) (-5894.709) -- 11:33:51      50000 -- [-5828.440] (-5840.263) (-5877.494) (-5867.420) * (-5829.884) (-5861.092) [-5828.087] (-5917.348) -- 11:33:11      Average standard deviation of split frequencies: 0.069290      51000 -- [-5830.653] (-5840.116) (-5858.028) (-5866.168) * [-5837.548] (-5849.233) (-5845.903) (-5916.712) -- 11:32:31      52000 -- [-5825.040] (-5847.446) (-5861.203) (-5876.138) * (-5837.926) [-5846.211] (-5845.608) (-5897.923) -- 11:31:53      53000 -- [-5832.301] (-5848.468) (-5866.739) (-5868.150) * [-5829.867] (-5838.652) (-5843.956) (-5907.204) -- 11:34:24      54000 -- [-5837.063] (-5850.016) (-5864.572) (-5854.854) * [-5824.780] (-5849.280) (-5867.752) (-5895.319) -- 11:33:45      55000 -- (-5835.376) [-5844.336] (-5870.168) (-5849.556) * [-5829.457] (-5875.140) (-5856.015) (-5910.576) -- 11:33:08      Average standard deviation of split frequencies: 0.065858      56000 -- (-5825.338) [-5824.613] (-5846.170) (-5861.583) * [-5816.678] (-5859.348) (-5849.892) (-5901.999) -- 11:32:31      57000 -- [-5822.061] (-5854.728) (-5860.854) (-5855.874) * (-5838.834) (-5880.936) [-5839.301] (-5888.935) -- 11:34:50      58000 -- [-5828.359] (-5842.195) (-5873.913) (-5841.838) * (-5841.349) (-5877.360) [-5826.372] (-5894.439) -- 11:34:13      59000 -- (-5826.330) [-5841.215] (-5893.453) (-5865.163) * (-5848.418) [-5845.632] (-5830.894) (-5891.391) -- 11:33:37      60000 -- (-5842.128) [-5827.680] (-5888.081) (-5852.096) * [-5828.603] (-5871.100) (-5838.979) (-5891.224) -- 11:33:02      Average standard deviation of split frequencies: 0.061488      61000 -- (-5840.797) [-5817.349] (-5882.566) (-5876.984) * (-5831.985) (-5874.815) [-5833.140] (-5913.783) -- 11:32:28      62000 -- (-5842.844) [-5812.581] (-5859.542) (-5896.731) * (-5849.573) (-5876.009) [-5820.350] (-5918.518) -- 11:34:35      63000 -- (-5844.627) [-5811.860] (-5851.485) (-5883.284) * (-5833.082) (-5849.060) [-5836.288] (-5896.217) -- 11:34:00      64000 -- (-5839.702) [-5824.129] (-5860.151) (-5853.778) * (-5825.506) (-5867.007) [-5829.224] (-5903.701) -- 11:33:27      65000 -- (-5837.194) [-5822.630] (-5840.934) (-5855.243) * (-5843.428) (-5838.560) [-5824.598] (-5903.501) -- 11:32:54      Average standard deviation of split frequencies: 0.059174      66000 -- (-5847.376) [-5823.247] (-5858.149) (-5885.099) * (-5834.296) [-5842.307] (-5824.782) (-5907.508) -- 11:32:22      67000 -- [-5845.398] (-5840.217) (-5844.355) (-5870.145) * [-5825.513] (-5867.225) (-5838.204) (-5888.155) -- 11:34:19      68000 -- (-5846.729) [-5828.478] (-5850.807) (-5854.781) * (-5829.062) (-5830.769) [-5831.385] (-5883.113) -- 11:33:46      69000 -- (-5845.286) [-5833.206] (-5845.245) (-5865.574) * [-5830.072] (-5842.671) (-5835.031) (-5926.789) -- 11:33:15      70000 -- (-5836.037) [-5830.042] (-5862.471) (-5857.819) * [-5819.041] (-5867.267) (-5839.353) (-5914.808) -- 11:32:44      Average standard deviation of split frequencies: 0.055091      71000 -- [-5827.020] (-5829.942) (-5861.492) (-5857.140) * [-5829.525] (-5878.142) (-5821.861) (-5920.867) -- 11:32:13      72000 -- (-5827.914) [-5849.230] (-5856.922) (-5866.092) * [-5824.573] (-5863.824) (-5835.174) (-5892.246) -- 11:31:44      73000 -- [-5820.765] (-5845.719) (-5841.846) (-5862.060) * [-5830.641] (-5844.253) (-5840.935) (-5894.238) -- 11:33:31      74000 -- [-5833.084] (-5838.942) (-5858.376) (-5872.641) * [-5819.644] (-5849.560) (-5835.408) (-5910.137) -- 11:33:01      75000 -- [-5846.315] (-5843.886) (-5859.004) (-5859.716) * [-5826.549] (-5843.576) (-5829.210) (-5881.909) -- 11:32:32      Average standard deviation of split frequencies: 0.047910      76000 -- (-5827.655) (-5837.619) [-5830.240] (-5859.257) * [-5842.693] (-5846.456) (-5837.869) (-5874.529) -- 11:32:04      77000 -- [-5837.774] (-5837.263) (-5843.613) (-5851.346) * (-5823.628) (-5844.766) [-5831.981] (-5887.319) -- 11:33:45      78000 -- (-5845.273) [-5828.858] (-5855.887) (-5851.028) * [-5835.519] (-5852.044) (-5855.922) (-5896.226) -- 11:33:16      79000 -- (-5836.907) [-5815.234] (-5852.946) (-5872.276) * (-5855.035) [-5837.093] (-5853.231) (-5877.441) -- 11:32:47      80000 -- (-5839.487) [-5818.781] (-5854.119) (-5871.114) * [-5818.501] (-5821.027) (-5843.477) (-5867.950) -- 11:32:20      Average standard deviation of split frequencies: 0.046368      81000 -- (-5823.427) [-5837.879] (-5862.637) (-5883.202) * (-5839.491) [-5814.247] (-5842.678) (-5883.070) -- 11:33:55      82000 -- [-5807.687] (-5830.337) (-5879.943) (-5864.310) * (-5851.209) [-5830.144] (-5836.381) (-5872.992) -- 11:33:27      83000 -- [-5822.070] (-5840.452) (-5853.436) (-5865.683) * (-5832.898) [-5826.727] (-5828.014) (-5857.714) -- 11:32:59      84000 -- [-5819.998] (-5868.038) (-5859.054) (-5850.785) * [-5828.287] (-5827.171) (-5867.308) (-5866.251) -- 11:32:32      85000 -- [-5829.738] (-5853.120) (-5828.307) (-5853.052) * (-5839.214) [-5826.604] (-5861.304) (-5868.000) -- 11:34:03      Average standard deviation of split frequencies: 0.045987      86000 -- [-5832.474] (-5866.784) (-5826.262) (-5859.407) * [-5823.624] (-5839.194) (-5857.177) (-5870.456) -- 11:33:35      87000 -- [-5818.689] (-5868.171) (-5825.896) (-5874.185) * [-5830.442] (-5836.678) (-5860.966) (-5889.442) -- 11:33:09      88000 -- [-5821.928] (-5851.391) (-5823.078) (-5871.447) * (-5836.216) [-5825.617] (-5865.094) (-5868.076) -- 11:32:42      89000 -- (-5817.116) (-5859.954) [-5833.386] (-5862.514) * [-5822.251] (-5815.629) (-5877.372) (-5856.741) -- 11:32:17      90000 -- [-5816.905] (-5873.271) (-5841.699) (-5855.448) * [-5813.770] (-5836.064) (-5864.930) (-5876.738) -- 11:31:51      Average standard deviation of split frequencies: 0.048527      91000 -- (-5819.968) [-5834.903] (-5862.874) (-5874.723) * [-5815.480] (-5828.434) (-5848.880) (-5869.320) -- 11:31:27      92000 -- (-5861.344) [-5826.952] (-5849.557) (-5874.440) * [-5832.518] (-5841.772) (-5840.082) (-5862.392) -- 11:32:50      93000 -- [-5845.598] (-5852.741) (-5832.685) (-5854.905) * [-5824.683] (-5848.986) (-5845.333) (-5869.455) -- 11:32:25      94000 -- (-5852.667) (-5836.898) [-5830.960] (-5868.302) * [-5830.035] (-5850.705) (-5828.261) (-5870.152) -- 11:32:00      95000 -- (-5849.578) (-5834.066) [-5823.085] (-5874.003) * (-5816.661) (-5837.562) [-5823.523] (-5860.623) -- 11:31:36      Average standard deviation of split frequencies: 0.047713      96000 -- (-5848.278) [-5823.774] (-5822.947) (-5865.639) * (-5829.769) (-5844.953) [-5825.082] (-5859.970) -- 11:31:13      97000 -- (-5835.252) [-5838.309] (-5828.956) (-5875.611) * (-5824.940) (-5824.458) [-5837.009] (-5848.425) -- 11:30:49      98000 -- (-5851.832) (-5840.942) [-5830.956] (-5881.097) * (-5830.633) [-5820.238] (-5825.767) (-5865.256) -- 11:32:07      99000 -- [-5834.936] (-5827.902) (-5837.401) (-5862.321) * (-5843.097) (-5837.443) [-5821.217] (-5855.292) -- 11:31:44      100000 -- [-5829.898] (-5848.673) (-5843.271) (-5880.221) * (-5865.506) (-5821.369) [-5827.333] (-5856.049) -- 11:31:21      Average standard deviation of split frequencies: 0.047930      101000 -- (-5834.353) [-5817.467] (-5846.596) (-5876.544) * (-5854.537) (-5821.991) [-5825.407] (-5869.891) -- 11:30:58      102000 -- (-5828.155) [-5813.978] (-5835.354) (-5858.989) * (-5862.771) (-5826.542) [-5830.200] (-5863.519) -- 11:32:12      103000 -- (-5834.981) [-5812.139] (-5852.330) (-5863.362) * (-5842.592) (-5815.325) [-5832.693] (-5864.664) -- 11:31:49      104000 -- (-5838.910) [-5825.134] (-5843.620) (-5862.736) * (-5828.560) [-5822.883] (-5838.317) (-5851.950) -- 11:31:27      105000 -- (-5835.739) [-5820.793] (-5849.162) (-5866.892) * [-5829.934] (-5847.770) (-5853.032) (-5856.083) -- 11:31:04      Average standard deviation of split frequencies: 0.046729      106000 -- [-5817.660] (-5836.258) (-5841.478) (-5865.850) * (-5837.023) [-5833.061] (-5866.659) (-5845.633) -- 11:32:16      107000 -- [-5817.951] (-5840.215) (-5825.201) (-5860.137) * (-5839.209) [-5842.065] (-5850.443) (-5857.775) -- 11:31:53      108000 -- (-5823.331) (-5829.794) [-5820.632] (-5890.945) * (-5834.929) [-5825.363] (-5852.551) (-5852.068) -- 11:31:31      109000 -- (-5830.913) (-5834.630) [-5823.396] (-5883.883) * (-5846.268) [-5821.576] (-5851.483) (-5854.355) -- 11:31:09      110000 -- [-5813.293] (-5835.982) (-5836.377) (-5886.506) * (-5830.903) [-5813.650] (-5832.813) (-5863.981) -- 11:32:18      Average standard deviation of split frequencies: 0.049342      111000 -- (-5829.193) (-5843.637) [-5813.329] (-5875.263) * [-5820.778] (-5828.939) (-5846.493) (-5854.517) -- 11:31:55      112000 -- (-5822.365) [-5839.318] (-5826.936) (-5884.743) * [-5834.460] (-5832.622) (-5843.113) (-5864.696) -- 11:31:34      113000 -- (-5828.709) [-5828.070] (-5841.121) (-5874.423) * (-5831.617) (-5827.488) [-5812.937] (-5862.228) -- 11:32:40      114000 -- [-5835.097] (-5832.731) (-5823.682) (-5870.868) * (-5823.654) (-5840.561) [-5821.187] (-5856.169) -- 11:32:18      115000 -- (-5836.818) [-5822.684] (-5838.068) (-5875.812) * (-5830.999) (-5856.998) [-5806.830] (-5867.328) -- 11:31:57      Average standard deviation of split frequencies: 0.048520      116000 -- (-5846.539) [-5815.858] (-5830.489) (-5854.800) * [-5825.871] (-5836.829) (-5831.464) (-5863.549) -- 11:31:35      117000 -- (-5832.422) [-5821.282] (-5832.663) (-5851.030) * [-5821.720] (-5840.213) (-5819.744) (-5877.546) -- 11:32:39      118000 -- (-5837.516) [-5811.473] (-5817.370) (-5887.861) * [-5815.027] (-5850.144) (-5836.657) (-5879.609) -- 11:32:17      119000 -- (-5835.773) (-5846.342) [-5835.088] (-5889.466) * (-5818.827) (-5850.352) [-5828.053] (-5891.667) -- 11:31:56      120000 -- [-5818.940] (-5838.850) (-5819.137) (-5878.671) * [-5811.485] (-5832.436) (-5845.640) (-5841.017) -- 11:31:36      Average standard deviation of split frequencies: 0.045538      121000 -- (-5826.231) [-5827.469] (-5834.844) (-5871.686) * (-5827.121) (-5834.666) (-5849.628) [-5829.316] -- 11:32:37      122000 -- [-5814.284] (-5821.479) (-5846.442) (-5852.287) * [-5819.108] (-5835.244) (-5854.772) (-5841.038) -- 11:32:16      123000 -- [-5819.789] (-5846.286) (-5848.259) (-5871.501) * [-5812.853] (-5840.961) (-5845.899) (-5869.698) -- 11:31:55      124000 -- (-5823.955) [-5831.668] (-5844.531) (-5892.718) * (-5822.165) [-5823.700] (-5848.459) (-5847.779) -- 11:31:35      125000 -- [-5826.823] (-5835.390) (-5856.616) (-5872.158) * (-5833.717) (-5838.795) [-5829.498] (-5856.872) -- 11:31:15      Average standard deviation of split frequencies: 0.045352      126000 -- (-5835.603) (-5843.705) [-5819.536] (-5868.951) * (-5830.997) [-5835.181] (-5830.872) (-5865.478) -- 11:32:13      127000 -- (-5842.236) [-5831.578] (-5833.425) (-5866.347) * (-5837.203) (-5840.210) [-5824.773] (-5861.145) -- 11:31:53      128000 -- (-5851.846) (-5843.646) [-5830.576] (-5865.226) * (-5849.006) (-5839.281) [-5822.630] (-5894.039) -- 11:31:33      129000 -- (-5871.751) [-5838.213] (-5829.370) (-5870.761) * (-5833.561) (-5841.730) [-5812.446] (-5868.803) -- 11:32:30      130000 -- (-5853.231) (-5840.946) [-5827.146] (-5880.039) * (-5855.498) (-5827.043) [-5817.181] (-5890.875) -- 11:32:09      Average standard deviation of split frequencies: 0.042251      131000 -- (-5846.415) (-5851.111) [-5824.641] (-5857.405) * (-5836.558) (-5844.501) [-5825.104] (-5877.516) -- 11:31:50      132000 -- (-5819.056) (-5834.390) [-5826.622] (-5893.258) * (-5855.006) [-5830.749] (-5838.672) (-5852.531) -- 11:32:45      133000 -- [-5820.635] (-5861.236) (-5849.576) (-5875.541) * (-5847.065) (-5824.435) [-5830.962] (-5840.266) -- 11:32:25      134000 -- [-5824.585] (-5847.296) (-5838.020) (-5864.029) * (-5859.662) (-5839.288) [-5818.519] (-5856.889) -- 11:32:05      135000 -- (-5846.765) (-5857.300) [-5826.274] (-5877.275) * (-5871.219) (-5839.009) [-5819.376] (-5860.081) -- 11:31:46      Average standard deviation of split frequencies: 0.042741      136000 -- (-5831.386) (-5853.665) [-5833.422] (-5843.612) * (-5871.753) (-5843.182) [-5816.591] (-5856.543) -- 11:32:39      137000 -- (-5838.755) (-5833.519) [-5843.688] (-5868.527) * (-5855.832) (-5841.236) [-5802.173] (-5857.713) -- 11:32:19      138000 -- [-5837.661] (-5844.965) (-5850.766) (-5857.012) * (-5872.254) (-5835.485) [-5822.995] (-5837.972) -- 11:32:00      139000 -- (-5842.504) (-5843.779) [-5827.937] (-5884.390) * (-5870.599) (-5820.819) [-5817.483] (-5843.792) -- 11:31:41      140000 -- (-5862.357) [-5836.649] (-5832.965) (-5872.398) * (-5854.690) [-5813.548] (-5820.419) (-5846.515) -- 11:31:22      Average standard deviation of split frequencies: 0.041328      141000 -- (-5869.294) [-5825.980] (-5841.871) (-5875.547) * (-5867.456) (-5829.395) [-5831.233] (-5860.775) -- 11:32:13      142000 -- (-5852.365) (-5836.401) [-5823.017] (-5867.495) * (-5828.866) (-5817.352) [-5833.155] (-5865.261) -- 11:31:54      143000 -- (-5850.279) [-5839.923] (-5826.064) (-5852.024) * (-5821.109) [-5805.443] (-5820.247) (-5854.523) -- 11:31:35      144000 -- (-5862.212) (-5832.460) [-5819.063] (-5844.086) * (-5847.916) [-5821.111] (-5841.911) (-5843.671) -- 11:31:17      145000 -- (-5853.981) (-5845.826) [-5826.755] (-5856.636) * (-5838.735) [-5814.830] (-5842.931) (-5857.865) -- 11:32:06      Average standard deviation of split frequencies: 0.041289      146000 -- (-5844.217) (-5855.532) [-5832.434] (-5853.000) * (-5848.681) (-5824.386) [-5817.484] (-5852.715) -- 11:31:48      147000 -- [-5836.725] (-5860.479) (-5840.354) (-5872.638) * (-5856.365) [-5826.419] (-5819.376) (-5842.819) -- 11:31:29      148000 -- (-5834.870) (-5858.605) [-5846.513] (-5866.715) * (-5844.493) [-5829.316] (-5834.069) (-5867.841) -- 11:31:11      149000 -- [-5837.981] (-5848.820) (-5847.076) (-5859.226) * (-5841.858) (-5857.424) [-5825.470] (-5862.100) -- 11:30:53      150000 -- (-5838.966) (-5849.432) [-5819.635] (-5888.461) * (-5860.485) (-5837.889) [-5827.151] (-5867.286) -- 11:30:35      Average standard deviation of split frequencies: 0.039896      151000 -- (-5852.024) (-5827.900) [-5827.400] (-5868.627) * (-5850.003) [-5817.703] (-5832.607) (-5858.336) -- 11:31:23      152000 -- (-5846.218) [-5824.940] (-5819.709) (-5863.914) * (-5835.004) [-5847.002] (-5861.638) (-5834.821) -- 11:31:05      153000 -- (-5850.960) [-5837.272] (-5815.841) (-5866.039) * [-5845.375] (-5845.864) (-5830.167) (-5859.944) -- 11:30:47      154000 -- (-5835.696) (-5860.762) [-5818.402] (-5863.492) * (-5832.338) [-5826.131] (-5841.161) (-5852.412) -- 11:30:29      155000 -- (-5825.681) [-5837.402] (-5828.753) (-5860.322) * (-5849.127) [-5823.314] (-5828.927) (-5867.756) -- 11:31:16      Average standard deviation of split frequencies: 0.039545      156000 -- (-5834.007) [-5824.385] (-5826.210) (-5883.487) * (-5841.758) [-5821.590] (-5852.338) (-5871.478) -- 11:30:58      157000 -- (-5857.744) [-5820.931] (-5834.334) (-5869.773) * [-5840.530] (-5816.102) (-5847.125) (-5862.771) -- 11:30:40      158000 -- (-5850.944) [-5818.846] (-5827.881) (-5859.934) * (-5844.858) [-5831.911] (-5830.086) (-5859.094) -- 11:30:23      159000 -- [-5848.705] (-5815.087) (-5827.101) (-5872.665) * (-5846.083) (-5850.032) [-5833.959] (-5839.674) -- 11:31:08      160000 -- (-5848.614) (-5816.882) [-5829.472] (-5865.413) * [-5827.818] (-5834.953) (-5835.975) (-5843.907) -- 11:30:51      Average standard deviation of split frequencies: 0.040171      161000 -- (-5854.546) (-5829.945) [-5837.030] (-5861.087) * (-5834.514) (-5846.570) [-5831.660] (-5850.145) -- 11:30:33      162000 -- (-5886.656) [-5810.854] (-5832.147) (-5852.599) * [-5836.297] (-5860.179) (-5860.226) (-5858.245) -- 11:31:17      163000 -- (-5887.367) (-5816.176) [-5831.676] (-5843.680) * (-5821.507) (-5870.670) (-5840.749) [-5842.547] -- 11:31:00      164000 -- (-5868.738) [-5833.060] (-5835.738) (-5867.454) * (-5839.726) (-5861.154) (-5837.316) [-5838.885] -- 11:30:43      165000 -- (-5872.356) [-5822.916] (-5846.180) (-5877.567) * (-5835.366) (-5851.321) [-5831.847] (-5849.732) -- 11:30:26      Average standard deviation of split frequencies: 0.039466      166000 -- (-5860.893) (-5832.733) (-5851.369) [-5829.501] * [-5812.678] (-5830.078) (-5860.128) (-5840.285) -- 11:30:09      167000 -- (-5846.305) [-5818.830] (-5839.808) (-5856.392) * (-5836.997) (-5824.351) (-5857.032) [-5845.108] -- 11:30:51      168000 -- (-5830.804) [-5832.457] (-5835.849) (-5859.113) * (-5839.450) [-5825.792] (-5852.835) (-5845.390) -- 11:30:34      169000 -- (-5840.320) (-5816.518) [-5841.885] (-5874.973) * [-5823.589] (-5836.226) (-5832.785) (-5858.584) -- 11:30:18      170000 -- (-5842.797) [-5829.767] (-5849.720) (-5857.909) * (-5837.440) [-5836.956] (-5821.347) (-5854.968) -- 11:30:59      Average standard deviation of split frequencies: 0.037690      171000 -- [-5826.230] (-5831.084) (-5854.625) (-5850.615) * (-5855.414) (-5830.132) [-5808.952] (-5860.989) -- 11:31:40      172000 -- (-5842.169) [-5824.801] (-5854.658) (-5837.573) * (-5845.659) [-5821.680] (-5818.111) (-5844.628) -- 11:31:23      173000 -- (-5849.214) [-5814.334] (-5863.045) (-5857.288) * (-5861.996) [-5817.527] (-5829.219) (-5843.402) -- 11:31:06      174000 -- (-5858.442) [-5815.430] (-5840.999) (-5828.997) * [-5842.960] (-5839.139) (-5842.733) (-5855.936) -- 11:31:46      175000 -- (-5847.069) [-5839.907] (-5835.909) (-5842.688) * (-5848.924) [-5839.547] (-5848.373) (-5863.118) -- 11:32:25      Average standard deviation of split frequencies: 0.036684      176000 -- (-5846.890) (-5835.050) (-5846.679) [-5846.169] * (-5840.250) [-5827.781] (-5841.327) (-5847.297) -- 11:32:08      177000 -- (-5834.698) [-5813.229] (-5849.485) (-5860.200) * (-5842.881) [-5827.643] (-5846.206) (-5836.253) -- 11:31:51      178000 -- (-5841.774) [-5822.275] (-5840.152) (-5862.852) * (-5848.766) [-5821.298] (-5824.208) (-5839.045) -- 11:32:30      179000 -- (-5845.905) [-5833.444] (-5831.780) (-5852.665) * (-5857.721) [-5818.981] (-5817.339) (-5846.294) -- 11:32:13      180000 -- (-5845.694) [-5831.424] (-5835.450) (-5849.961) * (-5844.504) [-5826.135] (-5815.170) (-5870.253) -- 11:31:56      Average standard deviation of split frequencies: 0.035694      181000 -- (-5852.243) [-5827.773] (-5848.528) (-5835.112) * (-5843.814) (-5839.815) [-5813.183] (-5867.039) -- 11:32:34      182000 -- (-5852.828) (-5813.785) (-5840.194) [-5833.995] * (-5827.699) (-5836.100) [-5829.067] (-5860.910) -- 11:32:17      183000 -- (-5849.353) (-5830.439) (-5844.729) [-5828.534] * (-5830.945) [-5809.527] (-5847.067) (-5861.228) -- 11:32:01      184000 -- (-5855.149) (-5827.615) (-5849.111) [-5808.802] * (-5833.288) [-5816.237] (-5866.904) (-5876.761) -- 11:31:44      185000 -- (-5844.612) [-5817.167] (-5839.339) (-5820.156) * (-5829.430) [-5821.140] (-5853.185) (-5876.938) -- 11:31:28      Average standard deviation of split frequencies: 0.033685      186000 -- (-5831.710) [-5819.840] (-5865.865) (-5831.477) * (-5858.569) [-5825.840] (-5846.724) (-5867.808) -- 11:32:04      187000 -- (-5842.757) [-5809.583] (-5853.484) (-5849.577) * (-5842.200) [-5814.395] (-5843.383) (-5845.587) -- 11:31:48      188000 -- (-5848.239) [-5820.270] (-5830.874) (-5832.481) * (-5839.524) (-5821.811) [-5821.308] (-5849.969) -- 11:31:32      189000 -- (-5835.686) [-5819.226] (-5834.731) (-5859.790) * (-5835.570) [-5816.178] (-5830.769) (-5858.560) -- 11:31:16      190000 -- [-5813.494] (-5837.486) (-5852.417) (-5847.505) * (-5819.218) [-5812.482] (-5828.410) (-5868.262) -- 11:31:00      Average standard deviation of split frequencies: 0.032291      191000 -- (-5832.925) (-5833.880) [-5845.790] (-5869.091) * (-5832.423) [-5811.287] (-5822.433) (-5852.639) -- 11:30:44      192000 -- (-5853.021) [-5823.243] (-5839.116) (-5839.536) * (-5833.852) [-5821.747] (-5829.669) (-5853.325) -- 11:31:19      193000 -- (-5861.491) [-5815.580] (-5849.938) (-5844.559) * (-5829.497) [-5835.375] (-5839.888) (-5869.963) -- 11:31:03      194000 -- (-5836.430) [-5800.601] (-5833.855) (-5865.261) * (-5828.161) [-5815.643] (-5825.662) (-5869.251) -- 11:30:48      195000 -- (-5838.685) [-5816.585] (-5858.812) (-5843.698) * (-5870.831) (-5832.878) [-5812.621] (-5882.369) -- 11:30:32      Average standard deviation of split frequencies: 0.029599      196000 -- (-5852.225) [-5809.261] (-5844.796) (-5838.956) * (-5843.800) (-5839.305) [-5819.049] (-5874.371) -- 11:30:16      197000 -- [-5848.831] (-5819.151) (-5848.449) (-5851.179) * [-5831.318] (-5850.322) (-5833.025) (-5867.536) -- 11:30:01      198000 -- (-5834.908) (-5837.900) [-5831.397] (-5843.066) * (-5832.956) (-5849.718) [-5814.818] (-5871.686) -- 11:30:35      199000 -- (-5834.909) [-5821.125] (-5832.977) (-5828.530) * (-5837.923) [-5821.792] (-5821.999) (-5860.904) -- 11:30:20      200000 -- (-5843.784) (-5839.716) (-5847.176) [-5840.259] * (-5827.908) (-5830.746) [-5827.658] (-5866.053) -- 11:30:04      Average standard deviation of split frequencies: 0.026798      201000 -- (-5853.974) [-5830.125] (-5860.027) (-5842.490) * (-5829.234) (-5841.843) [-5806.763] (-5848.201) -- 11:29:49      202000 -- (-5847.943) [-5825.109] (-5865.770) (-5845.019) * (-5851.546) (-5837.818) [-5817.167] (-5865.468) -- 11:30:23      203000 -- [-5829.455] (-5838.100) (-5851.161) (-5850.781) * (-5858.518) (-5848.269) [-5824.825] (-5859.122) -- 11:30:08      204000 -- [-5820.953] (-5839.111) (-5858.406) (-5842.798) * (-5847.149) (-5890.420) [-5824.364] (-5842.622) -- 11:29:52      205000 -- [-5797.824] (-5834.893) (-5860.439) (-5854.387) * (-5852.250) (-5868.309) (-5821.707) [-5836.719] -- 11:30:25      Average standard deviation of split frequencies: 0.026497      206000 -- [-5811.235] (-5832.737) (-5837.908) (-5858.728) * [-5834.462] (-5863.785) (-5831.947) (-5843.015) -- 11:30:10      207000 -- [-5822.614] (-5845.436) (-5838.536) (-5855.655) * [-5819.650] (-5856.569) (-5822.072) (-5852.282) -- 11:29:55      208000 -- (-5837.608) (-5843.102) [-5837.919] (-5873.446) * [-5824.857] (-5845.540) (-5831.189) (-5862.561) -- 11:29:40      209000 -- [-5835.437] (-5830.743) (-5855.560) (-5860.505) * (-5840.381) (-5835.539) [-5821.040] (-5916.540) -- 11:29:25      210000 -- [-5825.460] (-5844.142) (-5850.549) (-5866.467) * [-5832.112] (-5827.139) (-5823.832) (-5882.322) -- 11:29:57      Average standard deviation of split frequencies: 0.027054      211000 -- (-5833.360) [-5846.929] (-5843.020) (-5845.402) * [-5818.410] (-5836.970) (-5865.524) (-5877.059) -- 11:29:42      212000 -- (-5818.859) (-5850.549) (-5854.119) [-5835.353] * (-5817.491) [-5835.525] (-5853.782) (-5858.032) -- 11:29:28      213000 -- [-5811.812] (-5836.240) (-5854.655) (-5824.485) * [-5826.653] (-5842.478) (-5848.381) (-5855.847) -- 11:29:13      214000 -- [-5810.118] (-5834.665) (-5853.333) (-5851.623) * [-5826.053] (-5845.221) (-5841.683) (-5854.466) -- 11:28:58      215000 -- (-5829.552) [-5834.112] (-5855.226) (-5860.909) * (-5845.492) (-5860.648) [-5818.693] (-5854.600) -- 11:29:30      Average standard deviation of split frequencies: 0.026880      216000 -- (-5816.090) [-5821.384] (-5846.088) (-5839.454) * [-5834.375] (-5846.728) (-5826.447) (-5858.910) -- 11:29:15      217000 -- [-5827.026] (-5832.109) (-5857.545) (-5851.878) * (-5848.799) (-5839.509) [-5820.548] (-5836.615) -- 11:29:01      218000 -- (-5838.657) (-5864.878) (-5830.372) [-5826.697] * (-5835.702) (-5826.925) [-5826.515] (-5853.523) -- 11:29:31      219000 -- (-5826.922) (-5852.137) (-5826.026) [-5834.215] * (-5835.098) [-5829.151] (-5827.435) (-5844.654) -- 11:29:17      220000 -- (-5838.164) (-5863.634) [-5821.049] (-5841.218) * (-5844.383) (-5844.797) [-5809.223] (-5865.101) -- 11:29:02      Average standard deviation of split frequencies: 0.025132      221000 -- (-5835.567) (-5832.428) (-5837.556) [-5841.178] * (-5835.965) [-5830.138] (-5831.146) (-5872.649) -- 11:28:48      222000 -- (-5840.783) (-5848.608) [-5828.618] (-5842.028) * (-5819.509) (-5836.335) [-5815.183] (-5873.054) -- 11:29:18      223000 -- (-5824.083) (-5851.318) [-5830.656] (-5836.715) * (-5871.838) (-5844.989) [-5823.094] (-5884.580) -- 11:29:03      224000 -- [-5825.260] (-5850.433) (-5843.684) (-5842.095) * [-5827.037] (-5834.446) (-5833.548) (-5867.879) -- 11:29:33      225000 -- [-5825.348] (-5856.463) (-5822.091) (-5853.471) * (-5860.616) [-5827.014] (-5843.915) (-5872.781) -- 11:29:19      Average standard deviation of split frequencies: 0.024714      226000 -- (-5837.992) (-5857.883) [-5819.744] (-5841.267) * (-5863.572) [-5824.451] (-5839.525) (-5861.874) -- 11:29:48      227000 -- (-5832.744) (-5855.003) (-5825.011) [-5820.879] * (-5853.970) (-5830.465) [-5836.586] (-5865.934) -- 11:29:33      228000 -- (-5857.711) (-5849.736) [-5823.001] (-5843.427) * [-5818.449] (-5816.655) (-5847.986) (-5862.701) -- 11:29:19      229000 -- (-5857.182) (-5854.761) (-5829.031) [-5826.087] * [-5824.068] (-5817.090) (-5848.006) (-5857.417) -- 11:29:05      230000 -- (-5859.234) (-5849.554) (-5848.398) [-5828.869] * [-5818.499] (-5824.255) (-5851.283) (-5847.303) -- 11:29:33      Average standard deviation of split frequencies: 0.024103      231000 -- (-5844.562) (-5864.099) [-5844.766] (-5835.777) * [-5814.078] (-5825.840) (-5842.672) (-5847.667) -- 11:29:19      232000 -- [-5852.474] (-5854.533) (-5837.377) (-5825.297) * [-5805.769] (-5835.827) (-5860.482) (-5838.713) -- 11:29:47      233000 -- [-5837.143] (-5849.103) (-5834.701) (-5818.283) * [-5817.222] (-5854.429) (-5833.960) (-5824.898) -- 11:29:33      234000 -- (-5829.876) (-5832.565) (-5825.502) [-5811.759] * [-5828.775] (-5840.518) (-5830.884) (-5836.017) -- 11:30:01      235000 -- (-5814.142) (-5842.161) (-5862.752) [-5832.694] * [-5821.400] (-5831.197) (-5836.069) (-5844.163) -- 11:29:46      Average standard deviation of split frequencies: 0.024624      236000 -- [-5823.724] (-5848.606) (-5861.640) (-5840.217) * (-5820.958) [-5823.987] (-5849.781) (-5837.532) -- 11:29:32      237000 -- (-5818.592) (-5858.967) (-5859.221) [-5828.781] * (-5820.649) [-5807.164] (-5865.266) (-5863.522) -- 11:29:18      238000 -- (-5827.616) (-5866.666) (-5865.224) [-5825.646] * [-5825.054] (-5820.629) (-5850.660) (-5849.676) -- 11:29:45      239000 -- (-5835.351) (-5861.167) (-5865.951) [-5804.082] * [-5813.946] (-5831.803) (-5838.468) (-5852.071) -- 11:29:31      240000 -- (-5845.794) (-5856.348) (-5849.490) [-5821.653] * [-5821.192] (-5839.412) (-5842.760) (-5852.092) -- 11:29:18      Average standard deviation of split frequencies: 0.024062      241000 -- (-5875.472) (-5865.641) (-5837.889) [-5826.512] * (-5816.863) [-5827.999] (-5840.098) (-5835.916) -- 11:29:44      242000 -- (-5868.952) (-5847.988) (-5856.938) [-5817.071] * (-5830.698) (-5857.672) [-5832.024] (-5843.879) -- 11:29:30      243000 -- (-5843.085) [-5829.977] (-5865.488) (-5834.468) * [-5828.841] (-5850.013) (-5828.538) (-5869.322) -- 11:29:16      244000 -- (-5850.143) (-5836.637) (-5873.184) [-5833.616] * [-5830.214] (-5836.732) (-5841.261) (-5857.640) -- 11:29:43      245000 -- (-5843.799) [-5828.095] (-5854.411) (-5825.072) * [-5833.440] (-5853.888) (-5838.364) (-5841.850) -- 11:29:29      Average standard deviation of split frequencies: 0.023399      246000 -- (-5861.384) (-5830.741) (-5847.318) [-5829.807] * (-5828.013) (-5856.823) (-5862.500) [-5844.624] -- 11:29:55      247000 -- (-5852.903) (-5824.732) (-5845.665) [-5828.981] * (-5848.064) (-5834.297) (-5840.399) [-5844.796] -- 11:29:41      248000 -- (-5843.730) [-5832.463] (-5854.099) (-5840.627) * (-5841.557) (-5848.650) (-5848.725) [-5845.043] -- 11:29:27      249000 -- [-5839.715] (-5837.621) (-5846.150) (-5842.766) * (-5851.007) [-5831.130] (-5834.318) (-5846.469) -- 11:29:52      250000 -- (-5875.010) [-5812.871] (-5838.849) (-5837.185) * (-5843.801) (-5854.225) (-5842.072) [-5824.762] -- 11:29:39      Average standard deviation of split frequencies: 0.023279      251000 -- (-5863.761) [-5824.208] (-5854.964) (-5852.865) * (-5837.921) (-5843.815) (-5846.522) [-5812.640] -- 11:29:25      252000 -- (-5842.806) (-5840.707) (-5851.953) [-5842.881] * (-5839.197) (-5822.374) (-5841.455) [-5815.388] -- 11:29:50      253000 -- (-5856.521) (-5832.758) (-5844.739) [-5826.271] * (-5827.284) (-5848.030) [-5825.381] (-5826.111) -- 11:29:36      254000 -- (-5854.245) [-5837.246] (-5849.680) (-5826.825) * [-5835.847] (-5850.286) (-5829.350) (-5836.782) -- 11:29:22      255000 -- (-5864.000) [-5835.959] (-5837.147) (-5830.877) * (-5865.142) (-5829.804) (-5850.582) [-5847.420] -- 11:29:47      Average standard deviation of split frequencies: 0.023939      256000 -- (-5825.593) (-5848.979) (-5845.368) [-5811.983] * (-5870.905) (-5840.608) [-5812.019] (-5826.482) -- 11:29:33      257000 -- (-5853.015) (-5839.235) (-5844.573) [-5833.725] * (-5862.194) (-5838.530) (-5855.913) [-5831.785] -- 11:29:20      258000 -- (-5869.495) (-5845.801) (-5843.207) [-5819.720] * (-5842.838) [-5828.473] (-5845.672) (-5817.307) -- 11:29:06      259000 -- (-5837.305) (-5875.482) (-5841.825) [-5817.491] * (-5841.964) (-5822.843) (-5862.732) [-5814.954] -- 11:29:31      260000 -- (-5835.928) (-5845.810) (-5837.701) [-5831.564] * (-5843.884) (-5837.808) (-5863.952) [-5823.317] -- 11:29:17      Average standard deviation of split frequencies: 0.023873      261000 -- (-5854.502) (-5860.154) [-5822.569] (-5849.017) * (-5846.215) [-5826.505] (-5886.958) (-5828.685) -- 11:29:41      262000 -- (-5831.687) (-5850.528) [-5811.156] (-5841.682) * (-5846.242) [-5822.738] (-5851.328) (-5826.074) -- 11:29:27      263000 -- [-5820.757] (-5843.909) (-5826.612) (-5838.067) * (-5844.714) [-5813.489] (-5837.328) (-5850.068) -- 11:29:14      264000 -- [-5813.640] (-5835.776) (-5824.341) (-5847.294) * (-5841.604) (-5833.893) (-5849.570) [-5825.101] -- 11:29:38      265000 -- (-5812.785) (-5821.348) [-5814.972] (-5850.526) * (-5861.834) (-5838.775) (-5847.125) [-5821.241] -- 11:29:24      Average standard deviation of split frequencies: 0.022950      266000 -- (-5829.867) [-5825.565] (-5829.033) (-5855.288) * (-5871.417) (-5829.145) (-5851.921) [-5822.377] -- 11:29:47      267000 -- (-5847.363) [-5821.947] (-5838.025) (-5839.487) * (-5864.131) [-5822.022] (-5850.209) (-5839.507) -- 11:29:34      268000 -- [-5832.522] (-5836.241) (-5841.222) (-5844.140) * (-5878.225) [-5832.368] (-5853.973) (-5825.065) -- 11:29:21      269000 -- [-5819.071] (-5847.082) (-5858.530) (-5841.230) * (-5849.725) [-5822.115] (-5852.943) (-5844.822) -- 11:29:43      270000 -- [-5820.475] (-5844.283) (-5854.422) (-5832.973) * (-5861.829) [-5825.064] (-5849.439) (-5831.180) -- 11:29:30      Average standard deviation of split frequencies: 0.021942      271000 -- (-5831.559) [-5828.745] (-5866.026) (-5839.830) * (-5861.715) (-5851.929) (-5835.357) [-5827.913] -- 11:29:17      272000 -- [-5816.761] (-5823.374) (-5876.123) (-5838.304) * (-5854.418) (-5839.334) (-5820.867) [-5828.452] -- 11:29:04      273000 -- (-5811.332) (-5845.196) (-5860.321) [-5833.375] * (-5840.403) [-5827.687] (-5826.419) (-5849.420) -- 11:29:26      274000 -- [-5822.218] (-5835.073) (-5856.379) (-5827.295) * (-5846.972) (-5824.671) (-5836.379) [-5823.046] -- 11:29:13      275000 -- [-5811.373] (-5835.463) (-5850.354) (-5841.628) * (-5829.114) [-5818.430] (-5864.950) (-5833.303) -- 11:29:00      Average standard deviation of split frequencies: 0.021221      276000 -- [-5816.391] (-5861.631) (-5834.969) (-5834.970) * (-5829.166) (-5833.526) (-5841.240) [-5831.648] -- 11:29:22      277000 -- (-5834.720) (-5855.566) [-5822.681] (-5825.011) * (-5848.001) (-5856.694) (-5828.613) [-5824.919] -- 11:29:09      278000 -- [-5833.826] (-5862.387) (-5838.110) (-5815.030) * (-5862.112) [-5821.054] (-5854.060) (-5835.583) -- 11:28:55      279000 -- (-5834.615) (-5853.952) [-5828.969] (-5805.146) * (-5862.263) (-5838.265) (-5856.433) [-5830.656] -- 11:29:17      280000 -- (-5830.202) (-5847.917) (-5831.094) [-5810.004] * (-5841.644) (-5840.985) (-5872.910) [-5815.524] -- 11:29:04      Average standard deviation of split frequencies: 0.020772      281000 -- (-5823.257) (-5857.321) [-5824.106] (-5816.764) * (-5863.179) (-5837.898) (-5849.781) [-5815.221] -- 11:28:51      282000 -- (-5841.452) (-5881.924) [-5819.876] (-5828.223) * (-5840.449) (-5829.414) (-5851.907) [-5819.175] -- 11:29:13      283000 -- (-5856.258) (-5850.952) [-5816.553] (-5828.021) * (-5847.769) (-5838.538) (-5852.213) [-5818.330] -- 11:29:00      284000 -- (-5850.565) (-5836.696) [-5818.820] (-5856.187) * (-5832.117) (-5823.133) (-5854.059) [-5828.571] -- 11:28:47      285000 -- (-5870.122) (-5842.779) [-5819.685] (-5836.128) * [-5820.602] (-5819.858) (-5842.058) (-5812.558) -- 11:28:34      Average standard deviation of split frequencies: 0.021363      286000 -- (-5862.435) (-5837.946) [-5815.804] (-5858.687) * (-5846.548) (-5837.591) (-5844.732) [-5819.567] -- 11:28:55      287000 -- (-5856.156) (-5855.045) [-5812.155] (-5844.013) * (-5837.214) [-5817.312] (-5855.116) (-5834.758) -- 11:28:42      288000 -- (-5830.658) (-5862.709) [-5815.689] (-5833.852) * [-5814.395] (-5826.496) (-5838.233) (-5849.753) -- 11:29:03      289000 -- [-5824.340] (-5853.482) (-5840.138) (-5839.994) * (-5825.657) [-5833.381] (-5868.015) (-5840.298) -- 11:28:50      290000 -- (-5821.219) (-5858.966) (-5844.222) [-5822.808] * (-5823.435) [-5816.374] (-5845.133) (-5846.523) -- 11:28:37      Average standard deviation of split frequencies: 0.021126      291000 -- [-5817.115] (-5848.379) (-5825.623) (-5826.978) * (-5832.885) [-5814.122] (-5841.854) (-5838.652) -- 11:28:58      292000 -- (-5845.749) (-5871.559) (-5838.027) [-5829.003] * (-5830.824) [-5825.327] (-5847.195) (-5853.928) -- 11:28:45      293000 -- (-5842.084) (-5855.284) (-5844.393) [-5816.334] * (-5835.096) [-5831.706] (-5834.401) (-5838.243) -- 11:28:32      294000 -- (-5852.722) (-5850.443) [-5828.954] (-5835.566) * (-5846.009) [-5832.823] (-5830.105) (-5839.127) -- 11:28:53      295000 -- [-5846.425] (-5855.465) (-5831.542) (-5829.769) * [-5840.362] (-5831.688) (-5827.487) (-5844.503) -- 11:28:40      Average standard deviation of split frequencies: 0.021358      296000 -- (-5834.385) (-5857.430) [-5827.219] (-5837.497) * [-5824.051] (-5815.746) (-5854.262) (-5838.132) -- 11:29:00      297000 -- (-5839.229) (-5843.958) [-5819.143] (-5842.995) * [-5809.577] (-5830.730) (-5862.364) (-5832.191) -- 11:28:47      298000 -- (-5827.939) (-5846.371) (-5825.367) [-5835.177] * (-5837.330) [-5824.626] (-5847.542) (-5845.006) -- 11:28:34      299000 -- (-5824.458) (-5857.343) (-5845.026) [-5843.953] * (-5850.565) (-5863.962) [-5831.410] (-5843.975) -- 11:28:54      300000 -- (-5839.026) (-5856.591) (-5831.107) [-5820.696] * (-5831.273) (-5858.520) [-5822.999] (-5835.725) -- 11:28:42      Average standard deviation of split frequencies: 0.021099      301000 -- (-5828.355) (-5867.028) (-5850.796) [-5834.734] * (-5827.413) (-5860.241) (-5828.328) [-5823.963] -- 11:28:29      302000 -- [-5850.603] (-5867.714) (-5839.376) (-5844.155) * (-5844.566) (-5854.966) [-5816.887] (-5819.880) -- 11:28:48      303000 -- (-5826.242) (-5861.799) (-5843.359) [-5825.079] * (-5868.586) (-5843.731) [-5833.694] (-5816.453) -- 11:28:36      304000 -- (-5855.621) (-5845.258) [-5837.482] (-5830.911) * (-5845.792) (-5838.651) [-5822.486] (-5817.430) -- 11:28:23      305000 -- [-5838.235] (-5849.045) (-5827.181) (-5837.070) * (-5852.508) (-5856.568) [-5814.013] (-5820.221) -- 11:28:42      Average standard deviation of split frequencies: 0.020620      306000 -- [-5829.854] (-5860.757) (-5840.274) (-5828.166) * (-5850.881) (-5858.506) (-5840.581) [-5809.398] -- 11:29:02      307000 -- (-5828.668) (-5848.529) [-5818.259] (-5820.198) * [-5822.102] (-5842.136) (-5819.293) (-5827.955) -- 11:29:21      308000 -- (-5842.299) (-5864.567) [-5817.218] (-5847.190) * (-5830.022) (-5850.173) (-5831.647) [-5829.422] -- 11:29:39      309000 -- (-5838.844) (-5858.803) [-5812.874] (-5850.518) * (-5846.130) (-5833.145) (-5875.202) [-5812.280] -- 11:29:58      310000 -- [-5831.431] (-5848.580) (-5833.146) (-5830.927) * (-5845.761) (-5833.542) (-5855.876) [-5815.853] -- 11:30:16      Average standard deviation of split frequencies: 0.019589      311000 -- (-5819.964) (-5836.227) [-5833.434] (-5843.594) * (-5843.055) [-5817.945] (-5865.567) (-5838.318) -- 11:30:04      312000 -- [-5843.892] (-5856.204) (-5841.328) (-5848.343) * (-5847.052) [-5818.169] (-5850.933) (-5827.088) -- 11:30:22      313000 -- (-5838.362) (-5857.863) [-5839.441] (-5841.175) * (-5851.631) [-5823.906] (-5844.503) (-5813.016) -- 11:30:09      314000 -- (-5847.543) (-5849.460) [-5842.773] (-5829.509) * (-5871.521) (-5848.845) (-5831.735) [-5816.680] -- 11:30:27      315000 -- (-5842.474) (-5825.027) (-5832.674) [-5836.260] * (-5883.421) (-5824.911) (-5847.188) [-5815.501] -- 11:30:45      Average standard deviation of split frequencies: 0.019536      316000 -- (-5826.639) [-5810.514] (-5843.461) (-5846.847) * (-5867.385) (-5825.896) (-5858.152) [-5816.407] -- 11:30:32      317000 -- [-5826.130] (-5818.446) (-5842.068) (-5843.740) * (-5875.670) [-5829.626] (-5826.314) (-5821.889) -- 11:30:50      318000 -- [-5836.588] (-5835.631) (-5841.195) (-5846.001) * (-5851.808) [-5830.139] (-5828.864) (-5820.084) -- 11:30:37      319000 -- (-5845.796) (-5841.630) [-5833.121] (-5852.096) * (-5856.353) (-5819.626) [-5817.660] (-5843.711) -- 11:30:55      320000 -- (-5829.657) (-5835.423) (-5834.352) [-5838.288] * (-5863.537) [-5821.878] (-5831.002) (-5826.457) -- 11:30:42      Average standard deviation of split frequencies: 0.019921      321000 -- [-5821.289] (-5863.515) (-5823.154) (-5841.774) * (-5846.319) (-5832.212) (-5854.893) [-5823.663] -- 11:30:59      322000 -- [-5824.113] (-5865.956) (-5818.466) (-5842.353) * (-5835.507) (-5829.476) (-5831.574) [-5818.766] -- 11:30:47      323000 -- [-5827.462] (-5851.546) (-5832.701) (-5838.552) * (-5835.904) (-5846.823) (-5823.809) [-5824.671] -- 11:31:04      324000 -- [-5816.083] (-5854.487) (-5816.110) (-5854.299) * (-5834.317) (-5837.595) [-5817.383] (-5830.872) -- 11:30:51      325000 -- [-5824.473] (-5825.105) (-5816.876) (-5865.077) * (-5851.578) (-5842.787) [-5833.267] (-5831.902) -- 11:31:08      Average standard deviation of split frequencies: 0.019450      326000 -- (-5841.479) [-5821.930] (-5816.211) (-5850.014) * (-5873.648) (-5820.191) (-5827.190) [-5814.972] -- 11:30:55      327000 -- (-5835.513) [-5819.438] (-5832.029) (-5842.075) * (-5855.625) (-5839.291) (-5824.313) [-5830.482] -- 11:30:43      328000 -- (-5828.824) [-5811.992] (-5832.351) (-5847.233) * (-5861.262) [-5829.218] (-5835.500) (-5845.187) -- 11:30:59      329000 -- [-5826.298] (-5833.953) (-5813.999) (-5847.408) * (-5846.947) [-5825.651] (-5838.783) (-5836.739) -- 11:30:47      330000 -- (-5841.543) (-5832.683) [-5822.148] (-5859.589) * (-5844.609) [-5824.761] (-5832.286) (-5855.227) -- 11:30:34      Average standard deviation of split frequencies: 0.020099      331000 -- (-5856.998) (-5829.072) [-5835.327] (-5870.101) * (-5871.164) [-5826.378] (-5826.348) (-5875.550) -- 11:30:51      332000 -- (-5841.154) (-5844.972) [-5829.550] (-5857.762) * (-5847.394) (-5842.497) [-5824.048] (-5886.493) -- 11:30:38      333000 -- (-5847.637) (-5844.983) [-5839.422] (-5855.729) * [-5832.866] (-5832.754) (-5813.595) (-5848.585) -- 11:30:25      334000 -- (-5858.445) (-5843.278) [-5820.720] (-5851.323) * (-5837.707) [-5826.824] (-5815.498) (-5865.194) -- 11:30:42      335000 -- (-5841.916) (-5825.784) [-5824.182] (-5861.598) * (-5827.616) (-5829.931) [-5822.525] (-5848.095) -- 11:30:29      Average standard deviation of split frequencies: 0.019837      336000 -- (-5853.533) (-5827.854) [-5841.296] (-5843.758) * (-5832.458) (-5832.341) [-5830.166] (-5849.934) -- 11:30:17      337000 -- (-5841.497) [-5827.276] (-5840.294) (-5852.646) * [-5811.472] (-5863.688) (-5834.255) (-5860.684) -- 11:30:33      338000 -- (-5840.303) [-5831.427] (-5829.719) (-5844.881) * [-5808.188] (-5845.569) (-5845.348) (-5862.699) -- 11:30:20      339000 -- (-5843.074) [-5812.247] (-5827.418) (-5848.536) * [-5817.504] (-5861.103) (-5850.525) (-5839.753) -- 11:30:08      340000 -- (-5843.603) [-5832.025] (-5838.783) (-5838.916) * [-5812.478] (-5862.275) (-5837.560) (-5847.806) -- 11:30:24      Average standard deviation of split frequencies: 0.020445      341000 -- (-5861.409) (-5836.177) [-5839.156] (-5831.743) * [-5807.867] (-5840.048) (-5846.968) (-5865.789) -- 11:30:11      342000 -- (-5862.284) [-5831.144] (-5830.049) (-5842.406) * (-5831.628) (-5857.210) [-5831.275] (-5848.890) -- 11:30:27      343000 -- (-5850.539) (-5829.444) [-5833.947] (-5851.844) * (-5817.877) (-5858.622) (-5839.091) [-5832.211] -- 11:30:15      344000 -- (-5847.663) [-5818.992] (-5834.629) (-5843.771) * (-5841.720) (-5868.869) (-5828.032) [-5833.497] -- 11:30:02      345000 -- (-5863.641) (-5811.317) [-5840.865] (-5845.296) * (-5838.261) (-5858.819) [-5825.891] (-5827.718) -- 11:29:50      Average standard deviation of split frequencies: 0.020605      346000 -- (-5864.599) [-5811.928] (-5850.020) (-5835.528) * (-5842.352) (-5841.670) [-5820.798] (-5828.042) -- 11:30:06      347000 -- (-5854.909) [-5821.076] (-5842.850) (-5859.713) * (-5842.787) (-5836.860) (-5834.056) [-5828.107] -- 11:29:53      348000 -- (-5850.254) [-5823.419] (-5853.478) (-5860.519) * (-5834.440) [-5838.308] (-5841.841) (-5849.744) -- 11:29:41      349000 -- (-5835.589) [-5813.970] (-5853.282) (-5864.818) * [-5842.871] (-5863.669) (-5850.010) (-5826.715) -- 11:29:29      350000 -- (-5825.383) [-5823.391] (-5844.998) (-5864.880) * (-5830.940) (-5836.917) (-5864.577) [-5830.328] -- 11:29:44      Average standard deviation of split frequencies: 0.020712      351000 -- (-5834.196) [-5827.069] (-5841.325) (-5864.080) * [-5838.590] (-5868.678) (-5854.068) (-5835.961) -- 11:29:32      352000 -- (-5826.569) [-5826.602] (-5828.690) (-5841.242) * [-5830.008] (-5842.220) (-5854.719) (-5825.644) -- 11:29:20      353000 -- (-5843.768) [-5845.840] (-5856.164) (-5848.106) * (-5848.527) [-5827.140] (-5888.380) (-5833.374) -- 11:29:35      354000 -- (-5841.444) (-5824.740) [-5838.230] (-5846.297) * [-5826.263] (-5834.951) (-5882.850) (-5818.096) -- 11:29:23      355000 -- (-5833.852) [-5837.005] (-5872.648) (-5846.394) * (-5836.856) [-5826.377] (-5889.118) (-5830.316) -- 11:29:11      Average standard deviation of split frequencies: 0.020473      356000 -- [-5834.399] (-5827.322) (-5850.164) (-5852.430) * (-5835.886) (-5842.220) (-5866.935) [-5830.361] -- 11:29:26      357000 -- (-5824.904) [-5843.971] (-5835.947) (-5832.206) * [-5814.001] (-5841.657) (-5856.946) (-5839.149) -- 11:29:14      358000 -- (-5828.691) [-5820.650] (-5859.993) (-5838.204) * (-5843.266) (-5832.805) (-5854.788) [-5834.124] -- 11:29:02      359000 -- [-5820.076] (-5827.549) (-5852.528) (-5843.950) * (-5870.072) (-5834.229) [-5841.739] (-5841.510) -- 11:29:16      360000 -- (-5829.426) [-5812.480] (-5855.238) (-5841.894) * (-5849.059) [-5832.094] (-5837.395) (-5828.465) -- 11:29:04      Average standard deviation of split frequencies: 0.020624      361000 -- [-5837.011] (-5832.328) (-5852.537) (-5844.235) * (-5826.012) (-5833.003) (-5855.017) [-5826.586] -- 11:29:19      362000 -- (-5829.759) [-5821.499] (-5838.761) (-5848.676) * (-5853.394) [-5821.761] (-5862.459) (-5836.961) -- 11:29:07      363000 -- (-5827.721) (-5843.807) [-5825.305] (-5857.342) * (-5845.714) (-5825.556) (-5848.742) [-5833.737] -- 11:28:55      364000 -- (-5831.538) (-5853.961) [-5817.193] (-5860.400) * (-5826.967) (-5828.137) (-5897.925) [-5832.577] -- 11:29:10      365000 -- (-5829.927) (-5863.186) [-5817.359] (-5853.738) * [-5818.693] (-5832.461) (-5856.144) (-5848.717) -- 11:28:58      Average standard deviation of split frequencies: 0.021080      366000 -- (-5832.003) (-5861.130) [-5815.798] (-5838.334) * [-5815.629] (-5839.265) (-5842.944) (-5852.409) -- 11:29:12      367000 -- (-5838.220) (-5846.799) [-5822.338] (-5843.513) * [-5819.781] (-5840.971) (-5860.076) (-5844.342) -- 11:29:00      368000 -- (-5832.896) (-5845.413) [-5813.917] (-5850.438) * (-5828.665) [-5846.671] (-5845.280) (-5857.487) -- 11:28:48      369000 -- (-5830.169) (-5820.972) (-5820.386) [-5826.132] * (-5823.305) [-5829.543] (-5853.239) (-5864.501) -- 11:29:02      370000 -- (-5839.601) (-5826.358) [-5823.190] (-5849.153) * (-5826.973) [-5836.075] (-5839.937) (-5861.664) -- 11:28:50      Average standard deviation of split frequencies: 0.020844      371000 -- (-5866.246) [-5832.831] (-5822.546) (-5839.529) * (-5826.459) [-5821.280] (-5851.109) (-5875.671) -- 11:28:39      372000 -- (-5837.065) (-5844.892) [-5817.988] (-5833.876) * [-5831.085] (-5829.074) (-5851.801) (-5860.678) -- 11:28:27      373000 -- (-5848.680) (-5849.707) [-5825.441] (-5850.953) * (-5843.340) (-5840.322) [-5824.615] (-5854.843) -- 11:28:41      374000 -- (-5849.888) [-5840.551] (-5836.171) (-5868.343) * (-5861.207) (-5835.812) [-5832.825] (-5839.459) -- 11:28:29      375000 -- (-5850.523) [-5815.697] (-5838.531) (-5845.631) * (-5865.939) [-5840.987] (-5842.296) (-5851.342) -- 11:28:17      Average standard deviation of split frequencies: 0.021876      376000 -- (-5837.885) [-5835.124] (-5836.647) (-5856.644) * [-5838.434] (-5827.632) (-5855.489) (-5838.235) -- 11:28:31      377000 -- (-5846.946) (-5827.890) [-5833.144] (-5827.376) * (-5837.637) [-5832.936] (-5850.043) (-5862.846) -- 11:28:19      378000 -- (-5840.670) (-5842.679) (-5832.193) [-5824.325] * (-5842.029) [-5837.106] (-5824.935) (-5866.171) -- 11:28:08      379000 -- (-5839.229) (-5839.456) (-5829.613) [-5821.103] * (-5842.508) [-5838.260] (-5834.039) (-5854.285) -- 11:27:56      380000 -- (-5860.573) (-5840.530) [-5821.764] (-5849.762) * (-5839.574) [-5828.213] (-5853.478) (-5838.040) -- 11:27:44      Average standard deviation of split frequencies: 0.022928      381000 -- [-5825.543] (-5854.401) (-5830.662) (-5861.134) * (-5856.963) [-5826.560] (-5825.148) (-5866.049) -- 11:27:58      382000 -- (-5828.585) [-5852.549] (-5826.904) (-5854.626) * (-5850.907) [-5827.059] (-5825.001) (-5827.246) -- 11:27:46      383000 -- (-5830.596) (-5864.686) [-5823.245] (-5854.234) * (-5856.041) (-5823.628) (-5846.216) [-5813.621] -- 11:27:35      384000 -- (-5834.535) (-5847.288) [-5828.318] (-5872.817) * (-5859.076) (-5846.631) (-5826.888) [-5814.236] -- 11:27:23      385000 -- (-5854.511) (-5853.268) [-5826.695] (-5872.457) * (-5845.468) (-5870.733) [-5823.710] (-5827.972) -- 11:27:37      Average standard deviation of split frequencies: 0.023195      386000 -- (-5837.179) (-5865.446) [-5816.240] (-5852.220) * (-5838.123) (-5865.829) [-5830.630] (-5839.791) -- 11:27:25      387000 -- (-5848.830) (-5853.309) [-5831.073] (-5829.216) * (-5840.249) (-5853.954) [-5834.965] (-5831.169) -- 11:27:14      388000 -- (-5843.652) (-5864.039) [-5823.366] (-5836.006) * (-5841.817) (-5857.123) (-5822.109) [-5833.393] -- 11:27:02      389000 -- (-5837.998) (-5868.076) (-5839.022) [-5826.889] * [-5825.145] (-5851.204) (-5841.621) (-5832.886) -- 11:27:15      390000 -- (-5838.553) (-5872.668) [-5833.717] (-5834.984) * (-5820.099) (-5866.104) [-5828.315] (-5825.799) -- 11:27:04      Average standard deviation of split frequencies: 0.023823      391000 -- (-5857.219) (-5843.162) (-5842.689) [-5831.353] * (-5834.722) (-5833.803) [-5836.361] (-5850.893) -- 11:26:53      392000 -- (-5834.469) (-5842.100) (-5853.905) [-5822.438] * (-5833.244) [-5830.988] (-5843.051) (-5851.437) -- 11:27:06      393000 -- (-5832.429) (-5846.360) (-5844.054) [-5823.353] * [-5827.181] (-5835.339) (-5852.553) (-5832.466) -- 11:26:54      394000 -- (-5843.200) (-5838.248) (-5840.081) [-5813.791] * [-5818.347] (-5862.064) (-5849.944) (-5836.911) -- 11:26:43      395000 -- (-5833.053) (-5841.674) (-5841.711) [-5815.731] * [-5834.778] (-5851.644) (-5840.187) (-5840.984) -- 11:26:32      Average standard deviation of split frequencies: 0.023584      396000 -- (-5828.010) [-5829.386] (-5839.891) (-5827.147) * [-5834.733] (-5865.449) (-5867.016) (-5839.037) -- 11:26:45      397000 -- (-5832.265) [-5825.993] (-5859.775) (-5812.399) * [-5812.625] (-5859.078) (-5847.913) (-5833.764) -- 11:26:33      398000 -- (-5846.917) (-5831.851) (-5875.981) [-5818.518] * [-5824.286] (-5853.348) (-5832.022) (-5850.479) -- 11:26:46      399000 -- (-5827.418) [-5817.409] (-5859.691) (-5838.155) * (-5826.422) (-5859.655) [-5836.826] (-5847.988) -- 11:26:59      400000 -- (-5824.911) [-5814.243] (-5849.980) (-5841.921) * [-5835.209] (-5839.734) (-5830.045) (-5849.508) -- 11:27:12      Average standard deviation of split frequencies: 0.023431      401000 -- (-5816.051) [-5806.412] (-5865.488) (-5849.017) * (-5838.379) (-5846.852) [-5823.844] (-5870.427) -- 11:27:24      402000 -- (-5842.297) [-5813.757] (-5865.070) (-5842.251) * (-5830.790) (-5826.300) (-5828.977) [-5847.390] -- 11:27:13      403000 -- (-5845.773) [-5831.580] (-5855.922) (-5844.897) * (-5839.223) (-5839.748) [-5822.562] (-5837.567) -- 11:27:01      404000 -- (-5847.896) (-5842.867) [-5838.667] (-5849.728) * (-5838.506) (-5846.125) (-5835.144) [-5843.159] -- 11:27:14      405000 -- (-5848.565) [-5838.998] (-5829.802) (-5831.534) * (-5851.023) [-5821.774] (-5832.655) (-5842.627) -- 11:27:02      Average standard deviation of split frequencies: 0.022624      406000 -- (-5836.992) (-5861.528) [-5837.372] (-5833.172) * (-5854.315) [-5810.985] (-5820.292) (-5858.823) -- 11:27:15      407000 -- (-5839.036) (-5864.996) [-5841.729] (-5847.364) * (-5846.181) (-5842.617) (-5852.233) [-5832.810] -- 11:27:27      408000 -- (-5831.050) (-5848.162) (-5843.769) [-5821.823] * [-5831.848] (-5857.012) (-5858.312) (-5836.648) -- 11:27:16      409000 -- [-5832.648] (-5847.489) (-5845.506) (-5836.535) * (-5835.406) (-5860.541) (-5857.696) [-5811.586] -- 11:27:28      410000 -- (-5836.650) (-5849.846) (-5852.466) [-5832.008] * (-5832.034) (-5848.335) (-5847.101) [-5824.849] -- 11:27:40      Average standard deviation of split frequencies: 0.021917      411000 -- (-5835.220) (-5858.939) [-5844.157] (-5819.070) * [-5820.019] (-5850.683) (-5836.878) (-5828.049) -- 11:27:29      412000 -- [-5818.449] (-5858.757) (-5844.307) (-5835.406) * (-5832.145) (-5812.327) (-5864.642) [-5820.931] -- 11:27:40      413000 -- [-5836.018] (-5847.794) (-5831.133) (-5851.976) * [-5815.106] (-5828.539) (-5871.748) (-5830.841) -- 11:27:29      414000 -- [-5838.385] (-5858.480) (-5838.460) (-5857.199) * (-5823.883) (-5826.329) (-5847.983) [-5829.796] -- 11:27:41      415000 -- (-5844.845) (-5856.217) (-5838.287) [-5828.175] * [-5805.139] (-5830.352) (-5853.536) (-5841.821) -- 11:27:53      Average standard deviation of split frequencies: 0.020278      416000 -- (-5831.911) (-5858.821) (-5851.724) [-5809.687] * [-5812.733] (-5855.911) (-5838.844) (-5827.772) -- 11:28:04      417000 -- [-5834.639] (-5861.327) (-5831.244) (-5838.344) * (-5822.248) (-5859.916) [-5836.069] (-5845.737) -- 11:27:53      418000 -- (-5840.842) (-5843.653) [-5819.801] (-5840.080) * [-5818.465] (-5847.525) (-5840.137) (-5864.667) -- 11:28:05      419000 -- (-5847.387) (-5850.781) [-5832.428] (-5862.916) * [-5827.946] (-5827.834) (-5825.685) (-5849.187) -- 11:27:53      420000 -- (-5848.821) (-5836.589) [-5815.278] (-5876.536) * [-5818.942] (-5846.882) (-5832.244) (-5866.400) -- 11:28:05      Average standard deviation of split frequencies: 0.018922      421000 -- (-5846.633) [-5825.009] (-5827.270) (-5862.357) * [-5819.179] (-5832.202) (-5844.798) (-5854.045) -- 11:27:53      422000 -- (-5861.347) [-5839.244] (-5844.471) (-5842.230) * [-5826.602] (-5838.744) (-5843.446) (-5850.448) -- 11:27:42      423000 -- (-5862.572) (-5827.754) [-5833.912] (-5852.141) * [-5833.140] (-5831.398) (-5853.556) (-5817.656) -- 11:27:53      424000 -- (-5859.326) (-5830.663) [-5828.969] (-5845.824) * [-5821.505] (-5841.884) (-5847.937) (-5832.268) -- 11:27:42      425000 -- (-5847.632) (-5827.029) [-5835.152] (-5838.998) * (-5829.726) [-5828.249] (-5853.498) (-5845.313) -- 11:27:53      Average standard deviation of split frequencies: 0.018802      426000 -- (-5848.398) [-5813.185] (-5833.649) (-5833.406) * (-5836.083) [-5836.170] (-5852.780) (-5850.194) -- 11:27:42      427000 -- [-5831.132] (-5828.370) (-5856.849) (-5841.521) * [-5815.598] (-5835.463) (-5842.061) (-5854.625) -- 11:27:53      428000 -- (-5830.353) [-5818.764] (-5839.000) (-5835.629) * [-5822.496] (-5831.906) (-5850.211) (-5870.050) -- 11:27:42      429000 -- (-5853.272) (-5834.339) (-5863.970) [-5839.844] * (-5824.532) [-5839.934] (-5862.710) (-5870.249) -- 11:27:31      430000 -- (-5855.555) (-5842.144) (-5857.204) [-5827.648] * (-5820.204) [-5820.173] (-5859.655) (-5855.613) -- 11:27:42      Average standard deviation of split frequencies: 0.018831      431000 -- (-5846.310) (-5828.267) (-5855.003) [-5827.486] * (-5824.324) [-5809.772] (-5851.532) (-5850.165) -- 11:27:31      432000 -- (-5843.155) [-5832.175] (-5851.151) (-5861.748) * (-5824.009) [-5808.092] (-5848.537) (-5866.178) -- 11:27:42      433000 -- [-5822.265] (-5817.158) (-5852.149) (-5850.493) * (-5814.008) [-5820.228] (-5842.163) (-5870.287) -- 11:27:52      434000 -- (-5835.006) (-5823.414) (-5852.344) [-5837.709] * (-5820.677) [-5817.166] (-5850.645) (-5834.464) -- 11:27:41      435000 -- [-5818.532] (-5837.872) (-5830.994) (-5846.251) * [-5830.364] (-5832.914) (-5855.623) (-5859.183) -- 11:27:30      Average standard deviation of split frequencies: 0.018677      436000 -- (-5832.702) (-5858.260) [-5824.302] (-5849.824) * [-5821.946] (-5847.621) (-5848.745) (-5825.600) -- 11:27:41      437000 -- [-5811.567] (-5854.423) (-5851.682) (-5838.000) * [-5819.487] (-5845.494) (-5837.249) (-5838.684) -- 11:27:30      438000 -- [-5812.502] (-5834.784) (-5842.941) (-5855.658) * [-5817.286] (-5848.620) (-5854.916) (-5834.898) -- 11:27:18      439000 -- [-5814.211] (-5833.067) (-5837.537) (-5854.710) * [-5815.683] (-5842.866) (-5850.442) (-5837.689) -- 11:27:29      440000 -- [-5811.238] (-5844.716) (-5854.142) (-5833.540) * [-5810.804] (-5843.948) (-5844.356) (-5832.933) -- 11:27:18      Average standard deviation of split frequencies: 0.019397      441000 -- [-5819.628] (-5861.567) (-5857.323) (-5830.293) * [-5823.598] (-5863.890) (-5842.980) (-5841.730) -- 11:27:07      442000 -- [-5817.126] (-5866.454) (-5828.615) (-5846.107) * [-5820.085] (-5862.124) (-5835.860) (-5838.914) -- 11:27:17      443000 -- [-5826.713] (-5864.398) (-5830.441) (-5824.147) * [-5819.726] (-5880.096) (-5836.047) (-5841.940) -- 11:27:06      444000 -- (-5848.132) (-5860.579) (-5833.269) [-5823.922] * (-5838.947) (-5886.158) [-5839.619] (-5826.278) -- 11:26:55      445000 -- (-5841.996) (-5847.426) [-5829.890] (-5827.067) * (-5841.557) (-5880.536) (-5860.747) [-5826.397] -- 11:26:44      Average standard deviation of split frequencies: 0.020052      446000 -- (-5852.111) (-5830.522) (-5860.817) [-5835.317] * [-5832.546] (-5878.206) (-5853.473) (-5824.364) -- 11:26:55      447000 -- (-5846.977) [-5824.920] (-5861.917) (-5833.068) * [-5819.679] (-5869.556) (-5859.703) (-5827.427) -- 11:26:43      448000 -- (-5840.169) (-5837.115) (-5863.758) [-5829.488] * (-5834.450) (-5870.311) (-5842.050) [-5823.429] -- 11:26:54      449000 -- (-5836.813) (-5847.074) (-5876.270) [-5828.157] * (-5832.422) (-5845.464) (-5852.268) [-5814.749] -- 11:26:43      450000 -- (-5839.306) (-5848.838) (-5852.096) [-5824.092] * [-5820.205] (-5854.421) (-5848.171) (-5827.562) -- 11:26:32      Average standard deviation of split frequencies: 0.021421      451000 -- (-5843.326) (-5845.164) [-5843.083] (-5850.226) * (-5842.684) (-5850.924) (-5858.902) [-5810.992] -- 11:26:42      452000 -- [-5832.241] (-5825.223) (-5869.622) (-5858.556) * (-5833.541) (-5856.610) (-5849.770) [-5825.873] -- 11:26:52      453000 -- (-5830.594) [-5841.941] (-5843.768) (-5855.325) * (-5828.416) (-5862.562) (-5844.202) [-5840.151] -- 11:26:41      454000 -- [-5836.469] (-5830.451) (-5859.612) (-5852.758) * [-5839.098] (-5873.658) (-5845.162) (-5827.816) -- 11:26:30      455000 -- (-5834.061) [-5831.760] (-5854.110) (-5876.074) * (-5842.879) (-5874.021) (-5845.561) [-5814.355] -- 11:26:19      Average standard deviation of split frequencies: 0.021934      456000 -- (-5836.448) [-5812.549] (-5836.573) (-5843.293) * (-5849.532) (-5888.104) (-5845.030) [-5828.142] -- 11:26:29      457000 -- (-5848.418) (-5827.470) (-5851.415) [-5837.299] * (-5864.617) (-5858.378) (-5838.798) [-5834.758] -- 11:26:18      458000 -- (-5858.427) (-5828.380) (-5848.453) [-5826.574] * (-5883.201) (-5849.359) (-5825.934) [-5825.358] -- 11:26:08      459000 -- (-5865.586) [-5839.312] (-5859.145) (-5840.504) * (-5849.063) (-5842.181) [-5829.107] (-5845.523) -- 11:26:18      460000 -- (-5842.456) [-5818.338] (-5852.916) (-5832.990) * [-5833.815] (-5838.261) (-5866.333) (-5835.997) -- 11:26:07      Average standard deviation of split frequencies: 0.022125      461000 -- (-5837.766) [-5817.229] (-5861.896) (-5839.579) * (-5831.060) (-5840.313) (-5848.874) [-5835.426] -- 11:26:17      462000 -- (-5844.112) [-5825.436] (-5856.575) (-5848.038) * (-5829.484) (-5849.106) (-5848.119) [-5822.686] -- 11:26:06      463000 -- (-5856.079) [-5824.291] (-5857.816) (-5843.253) * (-5870.890) (-5840.155) (-5850.154) [-5841.810] -- 11:25:55      464000 -- [-5831.795] (-5824.219) (-5857.245) (-5841.529) * (-5844.449) [-5818.876] (-5842.591) (-5840.736) -- 11:26:05      465000 -- (-5831.632) [-5821.119] (-5856.282) (-5828.640) * (-5846.621) (-5834.094) (-5863.841) [-5835.812] -- 11:25:54      Average standard deviation of split frequencies: 0.022540      466000 -- (-5842.947) (-5820.768) (-5843.861) [-5819.239] * (-5841.145) [-5820.222] (-5850.342) (-5845.663) -- 11:26:03      467000 -- (-5838.042) [-5822.287] (-5874.399) (-5829.323) * (-5870.330) (-5825.586) (-5838.393) [-5827.981] -- 11:25:53      468000 -- (-5840.318) [-5827.290] (-5838.931) (-5839.151) * (-5865.826) (-5850.229) (-5828.608) [-5812.159] -- 11:25:42      469000 -- (-5833.995) [-5829.969] (-5852.082) (-5845.448) * (-5854.498) (-5824.108) (-5843.092) [-5817.236] -- 11:25:51      470000 -- [-5815.815] (-5858.297) (-5838.087) (-5831.175) * (-5875.149) (-5847.945) (-5834.290) [-5821.928] -- 11:26:01      Average standard deviation of split frequencies: 0.022337      471000 -- (-5840.238) [-5845.040] (-5852.016) (-5838.633) * (-5839.406) (-5843.687) (-5843.114) [-5829.278] -- 11:26:10      472000 -- (-5843.646) (-5836.742) [-5833.095] (-5839.993) * (-5839.159) [-5831.926] (-5847.122) (-5856.569) -- 11:26:00      473000 -- [-5827.417] (-5825.703) (-5831.510) (-5848.036) * [-5827.857] (-5828.511) (-5863.505) (-5854.264) -- 11:26:09      474000 -- (-5827.329) [-5828.831] (-5835.759) (-5838.110) * [-5829.853] (-5845.041) (-5851.267) (-5849.946) -- 11:26:18      475000 -- [-5813.600] (-5840.569) (-5870.780) (-5835.425) * (-5836.746) (-5849.664) (-5850.587) [-5831.795] -- 11:26:08      Average standard deviation of split frequencies: 0.022838      476000 -- [-5820.144] (-5850.726) (-5879.083) (-5837.292) * (-5852.937) [-5835.289] (-5863.755) (-5833.581) -- 11:25:57      477000 -- [-5832.305] (-5847.490) (-5847.200) (-5855.583) * (-5839.776) (-5830.840) (-5869.398) [-5832.811] -- 11:26:06      478000 -- [-5833.962] (-5865.328) (-5840.283) (-5852.298) * (-5839.858) (-5839.563) (-5882.032) [-5826.663] -- 11:25:55      479000 -- [-5823.722] (-5857.328) (-5851.029) (-5844.440) * [-5832.460] (-5842.683) (-5876.377) (-5848.434) -- 11:26:04      480000 -- (-5832.570) (-5850.574) [-5835.613] (-5848.804) * (-5842.450) (-5846.001) [-5831.765] (-5834.546) -- 11:25:54      Average standard deviation of split frequencies: 0.022457      481000 -- [-5828.787] (-5853.071) (-5845.462) (-5852.236) * [-5819.698] (-5852.603) (-5848.876) (-5835.975) -- 11:25:43      482000 -- (-5832.016) (-5861.299) [-5831.942] (-5848.727) * (-5848.007) (-5862.286) (-5828.848) [-5820.000] -- 11:25:52      483000 -- (-5827.036) (-5865.056) [-5819.841] (-5862.125) * (-5817.477) (-5856.927) (-5824.615) [-5822.569] -- 11:25:41      484000 -- (-5833.739) (-5830.186) [-5820.445] (-5858.628) * [-5811.770] (-5872.384) (-5836.887) (-5848.442) -- 11:25:31      485000 -- (-5843.396) (-5843.658) [-5807.909] (-5835.699) * [-5821.117] (-5843.171) (-5841.709) (-5870.351) -- 11:25:40      Average standard deviation of split frequencies: 0.021548      486000 -- (-5854.015) (-5845.060) [-5819.197] (-5847.838) * [-5823.062] (-5849.922) (-5858.490) (-5863.495) -- 11:25:29      487000 -- (-5836.818) [-5822.788] (-5827.722) (-5831.436) * [-5814.949] (-5825.485) (-5842.585) (-5873.404) -- 11:25:38      488000 -- (-5840.465) (-5849.070) (-5834.011) [-5825.911] * [-5808.096] (-5847.921) (-5853.363) (-5861.890) -- 11:25:27      489000 -- [-5830.907] (-5841.170) (-5849.210) (-5830.104) * [-5830.223] (-5852.243) (-5842.849) (-5845.753) -- 11:25:36      490000 -- [-5824.159] (-5853.342) (-5838.952) (-5853.599) * [-5826.079] (-5848.160) (-5838.040) (-5843.224) -- 11:25:25      Average standard deviation of split frequencies: 0.020979      491000 -- (-5822.598) [-5841.764] (-5842.100) (-5841.003) * (-5832.605) (-5861.134) (-5836.056) [-5828.099] -- 11:25:34      492000 -- (-5832.710) [-5824.521] (-5840.950) (-5840.304) * (-5836.311) (-5842.743) [-5816.476] (-5833.789) -- 11:25:24      493000 -- (-5865.448) (-5840.991) (-5836.835) [-5832.127] * (-5839.156) (-5858.412) [-5827.469] (-5835.222) -- 11:25:13      494000 -- (-5839.622) (-5871.401) [-5823.882] (-5844.979) * [-5837.505] (-5851.729) (-5834.526) (-5846.170) -- 11:25:22      495000 -- (-5828.284) (-5822.716) (-5825.896) [-5821.198] * [-5833.110] (-5842.242) (-5832.031) (-5834.078) -- 11:25:11      Average standard deviation of split frequencies: 0.020587      496000 -- [-5833.063] (-5815.283) (-5858.867) (-5841.881) * [-5831.580] (-5839.943) (-5834.336) (-5846.689) -- 11:25:20      497000 -- (-5847.312) [-5816.046] (-5871.700) (-5821.823) * (-5835.250) [-5815.877] (-5848.644) (-5841.311) -- 11:25:09      498000 -- (-5855.739) [-5821.200] (-5856.224) (-5820.271) * (-5832.876) [-5808.264] (-5843.470) (-5854.410) -- 11:24:59      499000 -- (-5846.980) (-5833.740) (-5862.131) [-5819.888] * (-5847.646) (-5828.610) (-5846.663) [-5851.894] -- 11:25:07      500000 -- [-5819.831] (-5854.299) (-5838.682) (-5843.108) * (-5835.050) [-5835.769] (-5835.144) (-5844.973) -- 11:24:57      Average standard deviation of split frequencies: 0.020836      501000 -- [-5829.808] (-5842.538) (-5832.184) (-5854.075) * (-5835.728) (-5846.360) [-5831.237] (-5837.010) -- 11:24:46      502000 -- (-5819.137) [-5810.193] (-5837.351) (-5840.463) * (-5842.405) (-5844.964) [-5834.619] (-5831.023) -- 11:24:54      503000 -- (-5827.356) [-5811.193] (-5863.949) (-5845.154) * (-5844.364) (-5845.836) [-5828.007] (-5824.893) -- 11:25:03      504000 -- (-5824.667) [-5823.112] (-5848.774) (-5850.320) * (-5842.474) (-5861.693) [-5815.968] (-5818.698) -- 11:24:52      505000 -- [-5817.034] (-5823.211) (-5856.759) (-5840.971) * (-5854.155) (-5830.911) (-5825.461) [-5828.162] -- 11:24:42      Average standard deviation of split frequencies: 0.020617      506000 -- (-5837.546) [-5814.708] (-5842.033) (-5847.442) * (-5854.250) (-5816.205) [-5838.633] (-5831.774) -- 11:24:50      507000 -- (-5850.381) (-5824.925) [-5839.477] (-5843.839) * [-5829.335] (-5829.727) (-5849.300) (-5841.372) -- 11:24:40      508000 -- (-5852.177) (-5830.009) [-5829.150] (-5834.868) * (-5841.805) [-5818.472] (-5840.235) (-5845.596) -- 11:24:48      509000 -- (-5864.874) [-5821.643] (-5844.536) (-5824.323) * (-5849.018) (-5817.465) (-5836.115) [-5834.168] -- 11:24:37      510000 -- (-5857.122) [-5820.966] (-5852.071) (-5841.448) * (-5844.963) (-5824.908) [-5820.109] (-5845.265) -- 11:24:27      Average standard deviation of split frequencies: 0.020554      511000 -- (-5850.645) [-5823.151] (-5846.507) (-5838.434) * (-5852.985) [-5819.094] (-5824.649) (-5828.174) -- 11:24:35      512000 -- (-5846.840) (-5838.397) (-5841.381) [-5816.889] * (-5863.519) (-5838.907) (-5838.751) [-5821.676] -- 11:24:25      513000 -- (-5850.577) (-5844.541) (-5834.978) [-5812.956] * (-5850.100) (-5823.734) (-5853.413) [-5812.816] -- 11:24:14      514000 -- (-5841.609) (-5852.207) [-5822.333] (-5817.814) * (-5853.041) (-5847.351) (-5837.264) [-5820.325] -- 11:24:22      515000 -- (-5856.757) (-5859.122) (-5824.548) [-5829.638] * (-5843.558) (-5847.326) (-5860.245) [-5828.559] -- 11:24:30      Average standard deviation of split frequencies: 0.020849      516000 -- (-5832.121) (-5845.473) [-5813.780] (-5847.042) * (-5836.087) (-5841.357) (-5864.288) [-5827.203] -- 11:24:20      517000 -- (-5840.568) (-5841.275) [-5808.788] (-5870.329) * (-5862.197) (-5843.397) (-5846.886) [-5814.863] -- 11:24:10      518000 -- (-5843.277) (-5840.669) (-5839.031) [-5827.100] * (-5853.992) (-5859.687) (-5868.580) [-5804.865] -- 11:24:18      519000 -- (-5822.698) (-5843.340) (-5848.418) [-5823.516] * (-5861.171) (-5834.384) (-5855.164) [-5823.999] -- 11:24:07      520000 -- [-5826.601] (-5878.928) (-5855.477) (-5825.937) * (-5852.094) (-5823.370) (-5865.868) [-5839.837] -- 11:24:15      Average standard deviation of split frequencies: 0.020724      521000 -- [-5818.453] (-5867.143) (-5853.635) (-5841.107) * (-5855.730) [-5828.715] (-5863.507) (-5821.825) -- 11:24:23      522000 -- [-5817.173] (-5847.511) (-5867.430) (-5855.211) * (-5883.978) (-5826.743) (-5859.085) [-5822.853] -- 11:24:13      523000 -- (-5812.158) (-5850.349) [-5833.534] (-5872.058) * (-5869.314) (-5838.321) (-5845.825) [-5820.706] -- 11:24:20      524000 -- [-5812.794] (-5861.009) (-5864.153) (-5830.541) * (-5828.159) (-5843.384) (-5861.494) [-5812.458] -- 11:24:10      525000 -- [-5818.482] (-5837.209) (-5854.976) (-5832.191) * (-5834.381) (-5823.748) (-5865.647) [-5822.438] -- 11:24:00      Average standard deviation of split frequencies: 0.020046      526000 -- (-5807.574) (-5861.804) (-5833.887) [-5832.895] * (-5847.525) (-5874.498) (-5850.514) [-5815.646] -- 11:23:49      527000 -- [-5821.496] (-5858.882) (-5830.288) (-5844.814) * (-5848.129) (-5874.249) (-5841.993) [-5825.609] -- 11:23:57      528000 -- (-5831.269) (-5833.670) [-5817.507] (-5870.481) * (-5845.971) (-5864.720) [-5838.274] (-5835.884) -- 11:23:47      529000 -- [-5830.433] (-5825.856) (-5823.172) (-5851.518) * (-5830.749) (-5870.288) [-5834.416] (-5830.262) -- 11:23:37      530000 -- [-5831.774] (-5828.839) (-5842.317) (-5857.431) * (-5852.421) (-5870.631) (-5831.349) [-5834.844] -- 11:23:26      Average standard deviation of split frequencies: 0.019792      531000 -- [-5831.432] (-5830.458) (-5835.211) (-5846.066) * (-5855.625) (-5870.164) (-5843.233) [-5830.137] -- 11:23:34      532000 -- [-5826.294] (-5837.118) (-5831.470) (-5843.409) * (-5846.852) (-5846.324) (-5851.564) [-5826.021] -- 11:23:24      533000 -- (-5828.090) (-5840.496) [-5806.821] (-5851.921) * [-5821.821] (-5828.474) (-5858.416) (-5844.161) -- 11:23:14      534000 -- (-5841.203) (-5829.999) [-5820.291] (-5854.233) * (-5835.207) [-5811.479] (-5855.023) (-5840.995) -- 11:23:21      535000 -- (-5836.361) (-5841.685) [-5813.132] (-5846.495) * (-5837.610) [-5809.631] (-5866.660) (-5846.681) -- 11:23:11      Average standard deviation of split frequencies: 0.020484      536000 -- (-5836.635) (-5837.556) [-5820.109] (-5850.584) * (-5829.922) [-5833.038] (-5841.519) (-5850.740) -- 11:23:01      537000 -- (-5846.027) [-5815.919] (-5822.595) (-5859.236) * (-5849.733) [-5835.184] (-5838.919) (-5871.388) -- 11:22:51      538000 -- (-5855.915) (-5845.135) [-5813.993] (-5856.725) * (-5871.744) [-5827.214] (-5834.836) (-5840.870) -- 11:22:58      539000 -- (-5852.938) (-5844.828) [-5822.173] (-5846.528) * (-5839.023) (-5853.957) [-5833.191] (-5829.610) -- 11:22:48      540000 -- (-5851.799) (-5856.070) [-5823.069] (-5863.586) * (-5825.841) (-5859.227) (-5833.707) [-5840.601] -- 11:22:38      Average standard deviation of split frequencies: 0.019660      541000 -- (-5850.983) (-5843.164) [-5832.530] (-5848.640) * [-5832.269] (-5855.872) (-5844.455) (-5857.212) -- 11:22:28      542000 -- (-5856.076) (-5834.854) [-5818.150] (-5838.034) * [-5822.775] (-5850.861) (-5856.030) (-5857.068) -- 11:22:35      543000 -- (-5854.152) (-5849.073) [-5825.146] (-5824.855) * [-5836.911] (-5844.332) (-5852.322) (-5864.520) -- 11:22:42      544000 -- (-5841.268) (-5856.236) [-5829.805] (-5830.431) * (-5846.950) [-5838.476] (-5849.053) (-5821.137) -- 11:22:50      545000 -- (-5850.532) (-5856.030) (-5837.880) [-5825.287] * (-5848.358) (-5833.969) (-5852.610) [-5827.342] -- 11:22:40      Average standard deviation of split frequencies: 0.019941      546000 -- [-5829.068] (-5872.390) (-5826.241) (-5853.394) * [-5833.038] (-5839.256) (-5843.994) (-5831.029) -- 11:22:47      547000 -- [-5843.215] (-5851.083) (-5822.124) (-5847.311) * [-5824.917] (-5867.085) (-5831.470) (-5831.789) -- 11:22:37      548000 -- (-5842.839) (-5844.440) [-5829.814] (-5848.997) * [-5831.586] (-5873.780) (-5841.764) (-5832.896) -- 11:22:27      549000 -- (-5859.362) (-5835.270) (-5851.270) [-5852.933] * [-5840.631] (-5850.221) (-5853.000) (-5838.591) -- 11:22:34      550000 -- (-5840.139) [-5835.667] (-5833.186) (-5852.471) * (-5845.316) (-5837.356) [-5834.553] (-5830.914) -- 11:22:24      Average standard deviation of split frequencies: 0.020188      551000 -- (-5860.426) (-5830.898) [-5819.348] (-5848.621) * (-5834.606) [-5832.283] (-5837.850) (-5834.443) -- 11:22:14      552000 -- (-5847.424) (-5834.071) [-5822.475] (-5818.510) * [-5817.705] (-5827.407) (-5834.504) (-5882.619) -- 11:22:21      553000 -- (-5840.160) (-5817.360) [-5827.945] (-5834.814) * [-5816.616] (-5834.226) (-5830.075) (-5865.342) -- 11:22:11      554000 -- (-5864.661) [-5830.239] (-5848.719) (-5842.767) * (-5827.073) (-5843.416) [-5839.520] (-5850.321) -- 11:22:18      555000 -- [-5857.592] (-5833.099) (-5848.805) (-5842.155) * [-5814.144] (-5824.786) (-5861.274) (-5832.066) -- 11:22:25      Average standard deviation of split frequencies: 0.020288      556000 -- (-5865.060) (-5839.263) [-5824.900] (-5826.250) * [-5832.102] (-5818.792) (-5854.824) (-5841.050) -- 11:22:15      557000 -- (-5850.982) (-5843.905) [-5831.759] (-5846.643) * [-5836.849] (-5847.353) (-5831.928) (-5861.052) -- 11:22:22      558000 -- (-5845.536) [-5839.252] (-5838.497) (-5848.704) * [-5821.102] (-5847.341) (-5848.554) (-5839.272) -- 11:22:12      559000 -- (-5834.855) (-5851.999) [-5833.263] (-5875.370) * (-5847.920) [-5832.244] (-5843.354) (-5845.891) -- 11:22:02      560000 -- (-5833.454) (-5862.720) [-5831.686] (-5849.682) * (-5843.104) [-5833.829] (-5829.320) (-5866.312) -- 11:22:09      Average standard deviation of split frequencies: 0.020306      561000 -- [-5818.830] (-5860.406) (-5833.814) (-5835.355) * (-5824.992) [-5816.071] (-5847.082) (-5855.159) -- 11:21:59      562000 -- (-5844.900) [-5826.347] (-5854.339) (-5841.710) * (-5828.320) [-5825.987] (-5849.878) (-5861.538) -- 11:21:49      563000 -- (-5866.843) (-5829.632) [-5826.966] (-5852.967) * (-5823.521) [-5822.883] (-5850.799) (-5854.106) -- 11:21:56      564000 -- (-5859.578) (-5818.447) (-5832.730) [-5830.377] * [-5823.610] (-5827.770) (-5851.115) (-5840.991) -- 11:21:46      565000 -- (-5820.143) (-5843.543) (-5841.272) [-5816.061] * [-5823.210] (-5848.571) (-5843.729) (-5841.672) -- 11:21:36      Average standard deviation of split frequencies: 0.020715      566000 -- (-5839.516) (-5830.549) [-5816.928] (-5835.874) * (-5833.735) (-5835.117) [-5824.248] (-5841.571) -- 11:21:42      567000 -- (-5836.763) (-5830.649) [-5832.488] (-5829.553) * (-5822.459) (-5842.092) (-5842.049) [-5825.086] -- 11:22:39      568000 -- [-5831.487] (-5856.557) (-5839.278) (-5834.421) * [-5810.264] (-5839.523) (-5832.836) (-5853.070) -- 11:22:46      569000 -- (-5834.755) (-5855.613) [-5839.909] (-5816.756) * (-5829.127) (-5852.945) [-5832.439] (-5834.993) -- 11:22:52      570000 -- [-5835.726] (-5861.823) (-5830.300) (-5827.219) * (-5829.060) (-5842.281) [-5815.793] (-5852.864) -- 11:22:59      Average standard deviation of split frequencies: 0.020990      571000 -- [-5829.313] (-5855.369) (-5841.340) (-5837.065) * (-5823.677) (-5859.877) [-5823.816] (-5843.038) -- 11:23:05      572000 -- [-5827.924] (-5862.868) (-5848.430) (-5826.355) * (-5835.996) (-5866.810) [-5807.663] (-5852.739) -- 11:22:55      573000 -- [-5828.099] (-5867.052) (-5846.001) (-5824.913) * (-5831.629) (-5869.425) [-5819.735] (-5838.488) -- 11:23:01      574000 -- [-5820.400] (-5859.100) (-5852.779) (-5836.924) * (-5825.167) [-5836.308] (-5846.018) (-5849.815) -- 11:22:51      575000 -- [-5813.960] (-5868.798) (-5836.659) (-5826.283) * [-5819.845] (-5835.836) (-5840.567) (-5839.850) -- 11:22:41      Average standard deviation of split frequencies: 0.021220      576000 -- [-5819.946] (-5871.603) (-5838.171) (-5829.829) * (-5825.454) (-5833.833) (-5840.123) [-5822.119] -- 11:22:48      577000 -- (-5824.616) (-5865.254) (-5830.253) [-5819.284] * [-5828.175] (-5836.208) (-5853.008) (-5830.480) -- 11:22:38      578000 -- (-5838.603) (-5866.598) (-5840.661) [-5815.032] * [-5809.947] (-5842.619) (-5835.728) (-5839.375) -- 11:22:44      579000 -- [-5827.276] (-5859.579) (-5829.429) (-5806.210) * (-5829.708) (-5844.786) (-5845.856) [-5823.050] -- 11:22:34      580000 -- (-5827.044) (-5861.937) (-5838.819) [-5811.738] * (-5852.028) (-5830.402) (-5853.486) [-5825.587] -- 11:22:24      Average standard deviation of split frequencies: 0.020939      581000 -- [-5811.614] (-5859.351) (-5843.977) (-5832.067) * [-5823.789] (-5832.397) (-5845.591) (-5850.038) -- 11:22:14      582000 -- [-5825.785] (-5852.433) (-5866.529) (-5826.310) * [-5819.786] (-5833.952) (-5849.505) (-5838.634) -- 11:22:20      583000 -- (-5827.436) (-5859.446) (-5853.662) [-5829.917] * (-5843.523) [-5824.771] (-5847.776) (-5824.399) -- 11:22:10      584000 -- [-5832.836] (-5857.990) (-5869.248) (-5835.723) * (-5841.645) [-5827.714] (-5846.404) (-5828.475) -- 11:22:00      585000 -- (-5854.002) (-5842.671) [-5841.357] (-5826.387) * (-5847.588) [-5814.899] (-5871.301) (-5833.702) -- 11:22:07      Average standard deviation of split frequencies: 0.020705      586000 -- (-5848.403) (-5845.464) [-5832.541] (-5849.492) * (-5850.873) [-5823.093] (-5858.775) (-5834.816) -- 11:21:57      587000 -- (-5861.532) [-5845.016] (-5832.744) (-5878.131) * (-5861.279) [-5824.878] (-5836.823) (-5840.639) -- 11:22:03      588000 -- (-5851.097) (-5829.043) [-5840.474] (-5862.101) * (-5857.377) [-5825.750] (-5825.972) (-5854.136) -- 11:21:53      589000 -- (-5834.892) [-5825.256] (-5822.389) (-5874.624) * (-5855.079) (-5830.460) [-5824.401] (-5834.188) -- 11:21:43      590000 -- [-5828.652] (-5844.517) (-5830.826) (-5884.159) * (-5859.833) (-5831.888) [-5813.666] (-5839.124) -- 11:21:49      Average standard deviation of split frequencies: 0.020845      591000 -- [-5818.636] (-5850.561) (-5836.755) (-5863.554) * (-5862.522) (-5830.465) [-5818.429] (-5827.992) -- 11:21:39      592000 -- [-5817.748] (-5851.823) (-5851.229) (-5854.391) * (-5859.170) (-5840.925) [-5821.483] (-5838.050) -- 11:21:45      593000 -- (-5856.739) (-5854.875) [-5824.635] (-5843.600) * (-5845.430) [-5823.162] (-5819.888) (-5847.659) -- 11:21:35      594000 -- (-5847.600) (-5873.131) [-5835.267] (-5853.719) * (-5851.840) (-5819.578) [-5820.959] (-5840.289) -- 11:21:41      595000 -- (-5843.528) (-5831.946) [-5825.350] (-5839.604) * [-5817.132] (-5827.976) (-5836.664) (-5852.399) -- 11:21:31      Average standard deviation of split frequencies: 0.021120      596000 -- (-5848.963) (-5836.752) [-5833.394] (-5836.439) * [-5824.419] (-5828.586) (-5853.451) (-5840.752) -- 11:21:37      597000 -- (-5841.147) (-5848.281) [-5828.508] (-5845.320) * (-5834.400) (-5854.590) (-5848.473) [-5823.080] -- 11:21:28      598000 -- (-5873.748) [-5835.093] (-5834.269) (-5822.912) * [-5827.738] (-5842.487) (-5863.621) (-5832.727) -- 11:21:33      599000 -- (-5851.996) (-5839.425) [-5830.921] (-5829.252) * [-5820.330] (-5851.243) (-5849.600) (-5861.004) -- 11:21:24      600000 -- (-5853.102) (-5823.809) (-5825.446) [-5820.016] * [-5814.377] (-5845.019) (-5831.880) (-5859.106) -- 11:21:14      Average standard deviation of split frequencies: 0.020882      601000 -- (-5839.465) (-5826.139) [-5832.035] (-5827.288) * [-5801.520] (-5846.459) (-5849.650) (-5834.790) -- 11:21:20      602000 -- (-5845.532) [-5831.352] (-5831.826) (-5830.759) * [-5807.550] (-5857.220) (-5851.901) (-5829.329) -- 11:21:10      603000 -- (-5846.700) (-5853.423) (-5846.343) [-5839.632] * [-5809.443] (-5850.632) (-5855.308) (-5836.921) -- 11:21:00      604000 -- [-5840.330] (-5851.162) (-5829.161) (-5849.334) * [-5810.425] (-5841.752) (-5860.196) (-5857.423) -- 11:20:50      605000 -- (-5875.768) [-5826.524] (-5842.421) (-5855.660) * (-5859.184) (-5837.317) (-5842.760) [-5837.554] -- 11:20:56      Average standard deviation of split frequencies: 0.020781      606000 -- (-5872.026) [-5823.245] (-5848.177) (-5840.856) * (-5846.614) (-5849.710) (-5848.671) [-5833.984] -- 11:20:46      607000 -- (-5857.142) [-5829.273] (-5837.251) (-5843.395) * (-5838.591) (-5837.006) [-5833.252] (-5845.696) -- 11:20:37      608000 -- (-5841.683) [-5837.420] (-5856.067) (-5851.202) * (-5821.516) [-5824.723] (-5838.768) (-5830.892) -- 11:20:42      609000 -- [-5831.064] (-5844.746) (-5843.103) (-5832.956) * (-5832.812) (-5842.608) (-5826.582) [-5826.246] -- 11:20:33      610000 -- (-5843.456) (-5841.342) (-5841.364) [-5830.956] * (-5817.772) (-5832.207) [-5827.654] (-5852.807) -- 11:20:38      Average standard deviation of split frequencies: 0.020272      611000 -- (-5873.295) [-5825.438] (-5832.000) (-5845.941) * (-5819.695) [-5826.972] (-5838.122) (-5871.267) -- 11:20:29      612000 -- [-5841.602] (-5833.354) (-5839.517) (-5855.652) * [-5826.959] (-5822.246) (-5835.795) (-5869.420) -- 11:20:19      613000 -- (-5842.567) (-5829.865) [-5820.360] (-5838.971) * (-5863.364) (-5833.032) [-5826.499] (-5850.465) -- 11:20:25      614000 -- [-5822.196] (-5811.966) (-5857.425) (-5832.419) * (-5862.551) (-5829.282) [-5819.987] (-5844.177) -- 11:20:15      615000 -- [-5813.362] (-5833.972) (-5858.823) (-5836.640) * (-5849.836) (-5835.865) [-5829.359] (-5851.912) -- 11:20:05      Average standard deviation of split frequencies: 0.019815      616000 -- (-5824.398) [-5843.933] (-5849.366) (-5856.254) * [-5842.410] (-5849.411) (-5832.365) (-5863.259) -- 11:20:11      617000 -- [-5819.942] (-5856.831) (-5831.710) (-5873.975) * [-5828.929] (-5836.182) (-5833.605) (-5864.255) -- 11:20:01      618000 -- [-5830.437] (-5856.553) (-5864.243) (-5870.180) * [-5810.652] (-5826.751) (-5850.634) (-5868.664) -- 11:20:07      619000 -- [-5827.330] (-5848.217) (-5834.830) (-5863.186) * [-5817.673] (-5834.970) (-5850.935) (-5850.316) -- 11:19:57      620000 -- [-5831.003] (-5829.341) (-5831.120) (-5891.021) * [-5829.710] (-5832.504) (-5850.101) (-5845.598) -- 11:19:47      Average standard deviation of split frequencies: 0.019306      621000 -- [-5829.347] (-5840.374) (-5846.965) (-5869.300) * [-5822.672] (-5840.272) (-5874.965) (-5854.221) -- 11:19:38      622000 -- (-5831.088) (-5852.220) [-5821.273] (-5877.342) * [-5819.741] (-5824.166) (-5835.933) (-5860.354) -- 11:19:43      623000 -- [-5829.915] (-5856.145) (-5835.403) (-5860.698) * [-5829.353] (-5836.171) (-5828.525) (-5848.215) -- 11:19:34      624000 -- (-5833.657) [-5821.616] (-5835.098) (-5849.425) * [-5830.709] (-5840.100) (-5819.328) (-5853.108) -- 11:19:24      625000 -- (-5854.531) (-5831.395) [-5828.571] (-5846.290) * [-5826.116] (-5850.461) (-5824.074) (-5849.814) -- 11:19:30      Average standard deviation of split frequencies: 0.019303      626000 -- (-5830.602) (-5823.785) [-5819.298] (-5861.664) * (-5843.462) (-5834.261) (-5836.469) [-5827.582] -- 11:19:20      627000 -- (-5826.698) [-5825.632] (-5840.736) (-5849.779) * [-5846.095] (-5837.021) (-5842.143) (-5829.517) -- 11:19:10      628000 -- (-5828.380) (-5820.676) [-5833.650] (-5845.886) * (-5836.250) (-5838.633) (-5869.662) [-5824.061] -- 11:19:16      629000 -- (-5838.195) [-5826.846] (-5829.093) (-5877.623) * (-5841.539) [-5832.783] (-5859.111) (-5817.732) -- 11:19:06      630000 -- (-5833.597) (-5839.130) [-5821.823] (-5867.606) * (-5857.745) (-5859.097) (-5829.792) [-5820.203] -- 11:18:57      Average standard deviation of split frequencies: 0.019233      631000 -- (-5836.818) [-5836.778] (-5824.189) (-5870.397) * (-5837.102) (-5853.360) [-5821.493] (-5818.387) -- 11:19:02      632000 -- (-5848.313) (-5835.371) [-5832.099] (-5870.574) * (-5844.829) (-5843.065) [-5817.015] (-5830.633) -- 11:18:53      633000 -- (-5871.532) (-5834.722) [-5838.644] (-5853.538) * (-5853.045) (-5856.332) (-5840.711) [-5817.992] -- 11:18:43      634000 -- (-5849.050) (-5837.728) [-5828.793] (-5857.400) * (-5857.442) [-5829.678] (-5847.104) (-5834.269) -- 11:18:48      635000 -- (-5839.841) [-5818.560] (-5842.455) (-5858.156) * (-5834.117) [-5810.836] (-5867.941) (-5821.551) -- 11:18:39      Average standard deviation of split frequencies: 0.019139      636000 -- (-5827.888) [-5835.574] (-5854.888) (-5862.692) * (-5849.791) (-5817.216) (-5850.909) [-5825.591] -- 11:18:29      637000 -- [-5822.352] (-5841.064) (-5841.210) (-5846.761) * (-5867.823) (-5827.906) (-5840.649) [-5827.176] -- 11:18:20      638000 -- (-5847.704) (-5819.559) (-5840.088) [-5831.606] * (-5853.473) (-5854.399) (-5841.467) [-5816.805] -- 11:18:25      639000 -- (-5839.793) [-5819.583] (-5834.386) (-5852.786) * [-5825.820] (-5853.017) (-5828.676) (-5832.871) -- 11:18:16      640000 -- (-5840.040) (-5837.887) [-5830.095] (-5844.014) * (-5814.753) (-5843.021) (-5844.789) [-5833.168] -- 11:18:06      Average standard deviation of split frequencies: 0.018131      641000 -- [-5817.701] (-5830.220) (-5839.995) (-5847.351) * [-5814.292] (-5832.352) (-5842.231) (-5821.050) -- 11:18:11      642000 -- [-5814.584] (-5843.567) (-5827.442) (-5835.945) * (-5824.468) [-5823.954] (-5861.335) (-5837.361) -- 11:18:02      643000 -- (-5811.834) (-5854.765) [-5819.985] (-5844.901) * [-5829.372] (-5824.309) (-5848.150) (-5857.132) -- 11:17:53      644000 -- [-5840.916] (-5840.442) (-5837.901) (-5844.875) * [-5843.906] (-5820.453) (-5878.807) (-5846.984) -- 11:17:58      645000 -- (-5844.851) (-5848.998) (-5826.446) [-5815.458] * (-5825.393) (-5839.006) (-5846.698) [-5810.481] -- 11:17:48      Average standard deviation of split frequencies: 0.018507      646000 -- (-5840.395) (-5863.306) (-5816.708) [-5815.815] * [-5831.972] (-5839.214) (-5852.967) (-5820.565) -- 11:17:39      647000 -- (-5834.883) (-5851.314) (-5821.868) [-5821.238] * (-5823.006) (-5850.448) (-5866.253) [-5833.844] -- 11:17:30      648000 -- (-5843.951) (-5851.005) [-5818.014] (-5819.828) * [-5819.123] (-5854.417) (-5842.040) (-5847.062) -- 11:17:35      649000 -- (-5836.350) (-5870.403) (-5823.464) [-5827.350] * [-5813.503] (-5846.353) (-5853.812) (-5853.760) -- 11:17:25      650000 -- (-5832.023) (-5865.791) [-5828.030] (-5836.265) * [-5823.023] (-5851.079) (-5850.092) (-5832.903) -- 11:17:16      Average standard deviation of split frequencies: 0.018251      651000 -- [-5838.840] (-5887.450) (-5825.813) (-5835.605) * (-5837.651) (-5818.601) (-5857.995) [-5828.973] -- 11:17:07      652000 -- (-5842.291) (-5856.970) (-5826.672) [-5828.039] * (-5833.972) [-5815.500] (-5860.022) (-5833.313) -- 11:16:57      653000 -- (-5843.610) (-5858.310) [-5811.225] (-5826.807) * (-5834.309) [-5827.180] (-5856.512) (-5836.345) -- 11:17:02      654000 -- (-5860.904) (-5864.060) [-5815.028] (-5819.240) * (-5840.258) (-5825.890) (-5848.786) [-5821.071] -- 11:16:53      655000 -- (-5841.219) (-5864.857) [-5821.654] (-5818.983) * (-5841.804) [-5817.987] (-5859.065) (-5830.101) -- 11:16:44      Average standard deviation of split frequencies: 0.017829      656000 -- (-5832.762) (-5864.878) (-5827.529) [-5817.777] * (-5845.072) (-5822.053) (-5845.076) [-5824.438] -- 11:16:35      657000 -- (-5828.859) (-5857.961) [-5817.908] (-5809.728) * (-5824.065) [-5811.373] (-5839.202) (-5843.455) -- 11:16:40      658000 -- (-5830.390) (-5862.464) (-5836.835) [-5811.129] * (-5840.871) [-5806.254] (-5866.339) (-5848.693) -- 11:16:30      659000 -- (-5822.421) (-5865.699) (-5827.192) [-5811.299] * (-5836.890) [-5812.261] (-5854.632) (-5835.056) -- 11:16:21      660000 -- (-5854.828) (-5841.138) [-5832.547] (-5812.772) * (-5833.769) [-5817.465] (-5860.736) (-5857.343) -- 11:16:26      Average standard deviation of split frequencies: 0.018054      661000 -- (-5839.065) (-5866.138) [-5824.099] (-5829.643) * (-5846.755) [-5827.828] (-5840.755) (-5850.322) -- 11:16:17      662000 -- (-5850.117) (-5849.374) [-5813.456] (-5840.569) * (-5849.408) [-5821.880] (-5883.341) (-5837.992) -- 11:16:08      663000 -- (-5829.792) (-5842.025) [-5811.417] (-5834.548) * (-5864.731) [-5817.494] (-5859.118) (-5844.455) -- 11:15:58      664000 -- (-5816.880) (-5835.019) [-5819.496] (-5843.515) * (-5877.327) [-5821.391] (-5835.598) (-5832.332) -- 11:16:03      665000 -- [-5833.875] (-5842.588) (-5838.410) (-5857.353) * (-5901.298) [-5826.728] (-5835.863) (-5848.080) -- 11:15:54      Average standard deviation of split frequencies: 0.018247      666000 -- [-5827.172] (-5843.110) (-5840.178) (-5850.439) * (-5875.231) (-5832.585) [-5840.430] (-5851.544) -- 11:15:45      667000 -- [-5826.728] (-5836.120) (-5856.477) (-5830.043) * (-5861.283) (-5819.468) [-5824.815] (-5834.695) -- 11:15:36      668000 -- (-5838.460) [-5844.295] (-5876.983) (-5853.605) * (-5871.785) [-5818.712] (-5829.490) (-5842.177) -- 11:15:41      669000 -- (-5829.853) (-5843.036) (-5835.785) [-5821.199] * (-5866.860) [-5819.885] (-5837.471) (-5853.950) -- 11:15:31      670000 -- (-5829.093) (-5862.861) (-5842.355) [-5825.225] * (-5851.805) [-5812.431] (-5829.077) (-5838.091) -- 11:15:22      Average standard deviation of split frequencies: 0.017991      671000 -- [-5821.856] (-5862.599) (-5871.214) (-5827.160) * (-5856.055) (-5818.469) [-5833.120] (-5852.238) -- 11:15:27      672000 -- [-5831.580] (-5849.903) (-5860.695) (-5841.858) * (-5848.644) [-5820.345] (-5836.412) (-5849.694) -- 11:15:18      673000 -- (-5849.664) (-5856.989) (-5860.033) [-5818.638] * (-5847.170) (-5816.299) (-5842.523) [-5826.576] -- 11:15:09      674000 -- (-5845.627) (-5842.878) (-5842.126) [-5814.879] * (-5844.948) [-5817.503] (-5840.878) (-5840.139) -- 11:15:00      675000 -- (-5842.124) (-5845.467) (-5841.624) [-5826.140] * (-5831.205) (-5816.544) [-5831.402] (-5873.648) -- 11:15:05      Average standard deviation of split frequencies: 0.018205      676000 -- [-5829.870] (-5851.046) (-5849.469) (-5831.093) * (-5840.222) (-5838.574) (-5828.645) [-5835.808] -- 11:14:55      677000 -- (-5859.016) [-5827.907] (-5849.820) (-5847.102) * (-5823.820) (-5850.511) (-5827.477) [-5826.148] -- 11:14:46      678000 -- (-5857.332) [-5827.232] (-5848.093) (-5852.185) * (-5835.223) (-5857.174) [-5832.839] (-5831.496) -- 11:14:37      679000 -- (-5843.856) [-5814.938] (-5834.612) (-5863.019) * (-5849.612) (-5830.286) (-5835.627) [-5810.947] -- 11:14:42      680000 -- (-5830.174) [-5833.613] (-5840.776) (-5859.710) * (-5867.664) (-5868.406) (-5836.250) [-5813.583] -- 11:15:00      Average standard deviation of split frequencies: 0.018185      681000 -- (-5846.733) [-5828.029] (-5838.569) (-5868.116) * (-5850.324) (-5845.164) [-5844.102] (-5826.832) -- 11:14:51      682000 -- [-5833.715] (-5839.686) (-5853.203) (-5838.737) * (-5832.308) (-5869.067) (-5854.977) [-5815.807] -- 11:14:42      683000 -- [-5817.033] (-5834.250) (-5860.593) (-5851.267) * (-5836.896) (-5872.536) (-5834.661) [-5828.587] -- 11:15:00      684000 -- (-5842.022) [-5826.097] (-5873.969) (-5834.346) * (-5835.202) (-5860.640) [-5818.522] (-5859.278) -- 11:15:05      685000 -- (-5837.181) (-5837.900) (-5853.092) [-5824.983] * (-5842.381) (-5861.195) (-5836.461) [-5828.092] -- 11:14:56      Average standard deviation of split frequencies: 0.018603      686000 -- (-5847.820) (-5858.090) (-5845.644) [-5817.637] * (-5876.035) (-5850.244) (-5858.165) [-5832.355] -- 11:15:00      687000 -- (-5853.363) (-5860.184) (-5855.333) [-5823.475] * (-5836.630) (-5849.204) (-5854.214) [-5830.354] -- 11:14:51      688000 -- (-5848.693) (-5852.839) [-5815.441] (-5819.416) * [-5829.838] (-5883.323) (-5832.552) (-5836.344) -- 11:14:56      689000 -- (-5862.392) (-5853.787) [-5816.677] (-5833.259) * (-5826.338) (-5855.892) (-5844.937) [-5841.595] -- 11:14:47      690000 -- (-5854.658) (-5846.757) [-5809.977] (-5842.881) * (-5826.272) (-5873.335) (-5852.091) [-5838.930] -- 11:14:51      Average standard deviation of split frequencies: 0.018399      691000 -- (-5844.719) (-5829.996) [-5808.793] (-5844.328) * (-5855.351) (-5845.097) [-5837.117] (-5856.191) -- 11:14:42      692000 -- (-5850.828) (-5844.851) (-5824.127) [-5823.877] * (-5841.532) (-5862.737) [-5834.520] (-5831.379) -- 11:14:33      693000 -- [-5838.601] (-5841.795) (-5834.089) (-5837.873) * (-5840.496) (-5862.551) [-5838.131] (-5856.498) -- 11:14:38      694000 -- (-5836.167) [-5835.131] (-5840.680) (-5833.175) * (-5838.294) (-5872.874) (-5848.324) [-5826.407] -- 11:14:29      695000 -- (-5843.471) (-5869.409) [-5828.291] (-5837.244) * (-5842.564) (-5865.095) (-5824.868) [-5843.774] -- 11:14:20      Average standard deviation of split frequencies: 0.019204      696000 -- (-5837.849) (-5863.895) (-5843.488) [-5821.480] * (-5834.072) (-5855.237) [-5828.013] (-5839.208) -- 11:14:24      697000 -- (-5844.996) (-5864.208) (-5852.504) [-5830.516] * (-5837.903) (-5863.870) (-5832.621) [-5838.876] -- 11:14:15      698000 -- (-5865.560) (-5845.066) [-5828.415] (-5856.071) * (-5838.347) (-5861.694) [-5841.648] (-5840.691) -- 11:14:06      699000 -- (-5855.637) (-5841.400) [-5819.550] (-5857.281) * (-5839.449) (-5838.079) [-5841.225] (-5842.600) -- 11:14:10      700000 -- (-5864.635) (-5849.095) [-5816.357] (-5847.278) * [-5829.560] (-5843.729) (-5856.631) (-5835.125) -- 11:14:01      Average standard deviation of split frequencies: 0.019641      701000 -- (-5866.613) (-5835.872) (-5827.037) [-5827.080] * [-5829.430] (-5857.617) (-5850.380) (-5821.203) -- 11:13:52      702000 -- (-5894.038) (-5826.705) (-5824.321) [-5818.168] * [-5806.960] (-5872.656) (-5833.692) (-5844.141) -- 11:13:43      703000 -- (-5860.881) (-5832.694) (-5829.855) [-5810.076] * [-5815.079] (-5864.199) (-5833.457) (-5846.592) -- 11:13:48      704000 -- (-5851.995) [-5847.262] (-5837.722) (-5836.091) * [-5818.362] (-5864.867) (-5838.216) (-5866.573) -- 11:13:39      705000 -- (-5857.030) (-5819.291) [-5823.767] (-5818.685) * [-5820.777] (-5852.181) (-5828.884) (-5849.278) -- 11:13:30      Average standard deviation of split frequencies: 0.020036      706000 -- (-5837.773) (-5824.607) (-5834.839) [-5811.203] * (-5832.182) (-5821.965) [-5818.748] (-5861.983) -- 11:13:34      707000 -- (-5857.003) (-5838.947) (-5843.898) [-5811.228] * (-5846.337) [-5828.069] (-5834.797) (-5867.213) -- 11:13:25      708000 -- (-5853.178) (-5829.333) (-5846.879) [-5805.012] * (-5842.622) [-5822.950] (-5858.300) (-5844.836) -- 11:13:16      709000 -- (-5828.913) (-5841.268) [-5832.481] (-5814.589) * (-5832.909) [-5823.991] (-5840.857) (-5865.659) -- 11:13:20      710000 -- (-5833.693) (-5840.782) (-5840.324) [-5816.163] * [-5828.353] (-5838.838) (-5828.534) (-5866.245) -- 11:13:11      Average standard deviation of split frequencies: 0.019991      711000 -- [-5805.511] (-5846.283) (-5848.017) (-5817.073) * [-5833.241] (-5820.412) (-5849.922) (-5838.976) -- 11:13:02      712000 -- [-5822.175] (-5854.805) (-5829.086) (-5833.039) * (-5836.648) [-5842.389] (-5857.807) (-5842.832) -- 11:13:07      713000 -- (-5829.170) (-5856.107) (-5817.870) [-5823.517] * (-5841.304) [-5827.579] (-5848.012) (-5856.289) -- 11:12:58      714000 -- (-5828.577) (-5843.903) [-5814.603] (-5830.521) * [-5822.870] (-5830.250) (-5861.470) (-5857.450) -- 11:12:49      715000 -- (-5850.446) (-5859.424) [-5813.553] (-5823.012) * (-5817.349) [-5811.202] (-5853.125) (-5870.535) -- 11:12:53      Average standard deviation of split frequencies: 0.020241      716000 -- (-5858.292) (-5845.301) [-5827.244] (-5837.555) * [-5822.067] (-5813.283) (-5847.696) (-5861.701) -- 11:12:44      717000 -- (-5846.629) [-5831.110] (-5824.998) (-5851.533) * (-5829.677) [-5824.033] (-5844.912) (-5860.397) -- 11:12:35      718000 -- (-5852.081) [-5828.679] (-5846.045) (-5852.223) * (-5832.712) [-5819.093] (-5850.855) (-5864.302) -- 11:12:26      719000 -- (-5841.130) (-5841.887) (-5835.238) [-5818.209] * (-5839.665) [-5823.471] (-5844.052) (-5839.448) -- 11:12:31      720000 -- (-5849.636) [-5818.354] (-5836.993) (-5822.144) * (-5867.103) (-5825.872) (-5848.759) [-5826.486] -- 11:12:22      Average standard deviation of split frequencies: 0.020773      721000 -- (-5856.376) [-5820.091] (-5824.339) (-5841.625) * (-5846.925) (-5820.278) [-5817.843] (-5847.343) -- 11:12:13      722000 -- (-5842.986) (-5827.537) [-5827.516] (-5840.216) * (-5850.711) [-5819.796] (-5847.341) (-5843.149) -- 11:12:04      723000 -- [-5820.692] (-5847.158) (-5852.845) (-5829.923) * (-5852.311) [-5824.892] (-5834.034) (-5842.126) -- 11:12:08      724000 -- (-5829.306) [-5847.880] (-5834.943) (-5826.571) * (-5838.840) [-5817.487] (-5831.652) (-5844.697) -- 11:11:59      725000 -- [-5816.890] (-5836.675) (-5835.553) (-5828.973) * (-5835.834) [-5822.682] (-5833.628) (-5846.009) -- 11:11:51      Average standard deviation of split frequencies: 0.020574      726000 -- [-5839.619] (-5820.429) (-5843.428) (-5855.019) * (-5832.968) [-5823.000] (-5854.524) (-5854.652) -- 11:11:55      727000 -- (-5831.859) [-5833.996] (-5857.304) (-5842.358) * (-5840.324) [-5827.024] (-5833.167) (-5863.950) -- 11:11:46      728000 -- (-5821.464) [-5814.663] (-5845.251) (-5820.614) * (-5845.558) (-5827.634) [-5822.231] (-5857.902) -- 11:11:50      729000 -- (-5832.870) [-5826.840] (-5827.368) (-5852.586) * (-5827.292) (-5827.086) [-5808.538] (-5846.784) -- 11:11:41      730000 -- (-5830.827) [-5826.739] (-5844.047) (-5844.712) * (-5845.079) (-5840.868) [-5831.205] (-5858.069) -- 11:11:32      Average standard deviation of split frequencies: 0.020780      731000 -- (-5836.353) [-5828.893] (-5839.793) (-5856.759) * (-5842.019) (-5831.799) [-5807.937] (-5856.299) -- 11:11:24      732000 -- [-5808.585] (-5845.550) (-5839.044) (-5851.161) * (-5820.984) (-5841.678) [-5831.462] (-5841.660) -- 11:11:15      733000 -- [-5819.339] (-5847.188) (-5829.677) (-5828.096) * (-5823.288) [-5821.822] (-5845.032) (-5875.613) -- 11:11:19      734000 -- (-5825.971) (-5849.232) [-5819.906] (-5829.015) * (-5838.866) (-5831.493) (-5851.543) [-5829.529] -- 11:11:10      735000 -- [-5825.736] (-5840.363) (-5847.381) (-5828.848) * (-5830.745) (-5851.273) (-5854.119) [-5825.362] -- 11:11:01      Average standard deviation of split frequencies: 0.021171      736000 -- (-5825.174) (-5851.410) (-5845.232) [-5832.284] * [-5828.723] (-5846.849) (-5852.521) (-5831.882) -- 11:10:53      737000 -- [-5825.491] (-5860.605) (-5860.870) (-5848.143) * (-5838.884) [-5825.602] (-5860.405) (-5835.118) -- 11:10:44      738000 -- (-5848.887) (-5864.570) (-5851.071) [-5832.508] * (-5829.345) [-5821.502] (-5844.068) (-5836.190) -- 11:10:48      739000 -- (-5852.877) (-5835.763) [-5824.743] (-5831.209) * [-5821.110] (-5822.922) (-5857.439) (-5833.241) -- 11:10:39      740000 -- (-5837.205) [-5818.511] (-5816.120) (-5847.161) * [-5823.896] (-5836.574) (-5851.447) (-5827.689) -- 11:10:30      Average standard deviation of split frequencies: 0.021173      741000 -- [-5822.477] (-5829.751) (-5840.858) (-5848.586) * (-5849.732) [-5829.793] (-5859.406) (-5834.560) -- 11:10:22      742000 -- (-5835.488) [-5832.648] (-5843.977) (-5857.546) * (-5854.788) (-5842.091) (-5857.389) [-5836.064] -- 11:10:26      743000 -- (-5830.515) (-5825.676) [-5823.934] (-5832.881) * (-5844.728) (-5853.693) (-5861.707) [-5822.795] -- 11:10:17      744000 -- [-5828.059] (-5816.650) (-5843.419) (-5870.491) * (-5828.823) (-5843.096) (-5871.733) [-5829.513] -- 11:10:08      745000 -- (-5827.363) (-5814.231) [-5812.344] (-5858.908) * [-5818.516] (-5830.501) (-5880.992) (-5842.838) -- 11:10:12      Average standard deviation of split frequencies: 0.021045      746000 -- (-5826.507) (-5835.936) [-5815.426] (-5844.151) * (-5838.797) [-5822.742] (-5896.331) (-5839.049) -- 11:10:04      747000 -- [-5828.412] (-5843.611) (-5832.954) (-5870.878) * [-5831.560] (-5831.800) (-5877.118) (-5839.658) -- 11:09:55      748000 -- (-5829.082) (-5837.444) [-5814.992] (-5865.348) * (-5840.018) [-5827.006] (-5853.156) (-5841.152) -- 11:09:46      749000 -- (-5831.340) (-5822.326) [-5814.468] (-5865.937) * (-5849.687) [-5818.221] (-5844.412) (-5822.840) -- 11:09:50      750000 -- [-5830.993] (-5828.208) (-5819.405) (-5866.712) * [-5830.195] (-5830.244) (-5824.613) (-5857.484) -- 11:09:42      Average standard deviation of split frequencies: 0.020709      751000 -- (-5840.188) (-5845.770) [-5825.502] (-5848.018) * (-5827.483) (-5835.767) [-5823.929] (-5872.595) -- 11:09:45      752000 -- (-5818.365) (-5858.474) [-5820.802] (-5843.581) * [-5817.460] (-5849.878) (-5827.154) (-5864.687) -- 11:09:37      753000 -- [-5818.009] (-5832.211) (-5840.616) (-5850.389) * (-5814.743) (-5836.636) [-5828.282] (-5861.067) -- 11:09:28      754000 -- [-5829.552] (-5828.287) (-5835.030) (-5842.729) * (-5825.665) (-5842.473) [-5830.393] (-5851.151) -- 11:09:32      755000 -- (-5841.535) (-5832.566) (-5828.950) [-5823.298] * (-5856.272) [-5832.244] (-5845.178) (-5854.501) -- 11:09:23      Average standard deviation of split frequencies: 0.020339      756000 -- (-5828.508) (-5841.405) (-5831.609) [-5829.216] * (-5843.428) [-5838.768] (-5860.524) (-5859.416) -- 11:09:27      757000 -- [-5820.377] (-5846.042) (-5851.824) (-5825.089) * (-5846.535) [-5825.238] (-5835.033) (-5858.244) -- 11:09:31      758000 -- (-5838.268) (-5855.360) (-5840.015) [-5814.182] * (-5843.333) (-5844.836) [-5819.001] (-5841.861) -- 11:09:22      759000 -- (-5833.496) [-5827.851] (-5845.746) (-5850.464) * (-5846.924) (-5840.075) (-5835.988) [-5836.765] -- 11:09:26      760000 -- (-5861.266) [-5831.232] (-5851.084) (-5836.000) * (-5863.025) (-5842.712) [-5824.946] (-5855.676) -- 11:09:17      Average standard deviation of split frequencies: 0.020689      761000 -- (-5830.414) [-5823.212] (-5867.988) (-5842.762) * (-5848.576) (-5838.810) [-5822.150] (-5856.123) -- 11:09:08      762000 -- [-5814.648] (-5829.011) (-5844.495) (-5815.634) * (-5845.668) (-5862.698) [-5823.718] (-5843.654) -- 11:09:00      763000 -- (-5826.226) (-5836.185) (-5858.638) [-5826.327] * (-5881.054) [-5844.635] (-5817.917) (-5845.269) -- 11:09:04      764000 -- (-5841.162) (-5845.265) (-5855.061) [-5815.561] * (-5863.352) (-5840.105) [-5823.368] (-5870.085) -- 11:08:55      765000 -- (-5833.689) (-5835.284) (-5839.660) [-5810.343] * (-5856.894) (-5862.952) [-5826.191] (-5843.897) -- 11:08:46      Average standard deviation of split frequencies: 0.021152      766000 -- (-5831.881) (-5841.123) (-5843.626) [-5834.052] * (-5865.476) (-5851.023) (-5819.782) [-5834.062] -- 11:08:50      767000 -- [-5824.951] (-5854.606) (-5830.281) (-5829.836) * (-5852.144) (-5846.652) (-5842.862) [-5830.094] -- 11:08:42      768000 -- (-5831.410) (-5856.448) (-5848.481) [-5821.970] * (-5844.386) (-5854.684) (-5833.636) [-5823.215] -- 11:08:33      769000 -- [-5828.533] (-5859.320) (-5842.361) (-5834.081) * (-5830.151) (-5850.712) (-5836.293) [-5836.231] -- 11:08:37      770000 -- (-5841.884) (-5873.400) [-5832.597] (-5839.092) * (-5839.830) (-5858.398) [-5836.263] (-5828.635) -- 11:08:28      Average standard deviation of split frequencies: 0.021708      771000 -- (-5868.152) (-5862.725) [-5832.704] (-5824.003) * (-5828.888) (-5867.508) (-5843.221) [-5826.803] -- 11:08:20      772000 -- (-5851.161) (-5852.145) (-5842.775) [-5822.587] * (-5832.553) (-5827.124) [-5830.405] (-5859.671) -- 11:08:23      773000 -- (-5858.576) (-5845.950) (-5849.457) [-5829.586] * (-5855.476) [-5823.251] (-5844.674) (-5858.908) -- 11:08:15      774000 -- (-5852.466) (-5841.552) (-5837.156) [-5820.967] * (-5846.911) [-5820.113] (-5855.754) (-5854.449) -- 11:08:18      775000 -- (-5846.147) (-5842.564) (-5854.296) [-5808.542] * (-5832.374) [-5817.418] (-5839.117) (-5852.094) -- 11:08:21      Average standard deviation of split frequencies: 0.021913      776000 -- (-5834.871) (-5842.067) (-5865.336) [-5818.232] * (-5863.113) [-5815.268] (-5845.191) (-5839.010) -- 11:08:25      777000 -- (-5845.026) (-5847.329) (-5850.010) [-5815.449] * (-5860.301) [-5818.302] (-5849.585) (-5841.456) -- 11:08:16      778000 -- (-5841.021) (-5860.229) (-5856.965) [-5815.949] * (-5833.967) [-5823.364] (-5840.935) (-5843.822) -- 11:08:20      779000 -- (-5842.196) (-5862.574) (-5859.197) [-5823.766] * (-5834.274) (-5847.026) [-5816.874] (-5840.394) -- 11:08:11      780000 -- (-5840.351) (-5851.868) (-5845.804) [-5834.341] * (-5834.278) (-5852.033) [-5839.264] (-5846.225) -- 11:08:15      Average standard deviation of split frequencies: 0.021939      781000 -- (-5853.913) [-5826.140] (-5839.141) (-5828.641) * (-5840.069) [-5829.607] (-5836.246) (-5835.771) -- 11:08:18      782000 -- (-5837.713) [-5820.786] (-5829.681) (-5830.322) * [-5824.407] (-5855.798) (-5828.905) (-5824.056) -- 11:08:21      783000 -- (-5835.548) (-5850.086) [-5819.239] (-5823.216) * [-5821.085] (-5850.038) (-5863.600) (-5838.090) -- 11:08:25      784000 -- [-5823.724] (-5855.416) (-5850.665) (-5843.076) * [-5820.343] (-5862.834) (-5855.102) (-5844.850) -- 11:08:16      785000 -- (-5826.565) (-5871.252) (-5841.236) [-5826.969] * [-5813.565] (-5864.372) (-5854.682) (-5859.021) -- 11:08:08      Average standard deviation of split frequencies: 0.021856      786000 -- (-5827.740) (-5850.253) (-5845.519) [-5826.665] * [-5825.428] (-5856.509) (-5823.265) (-5856.959) -- 11:08:11      787000 -- (-5833.686) (-5835.655) (-5848.329) [-5833.486] * (-5826.118) (-5889.432) [-5830.084] (-5851.122) -- 11:08:02      788000 -- (-5827.698) [-5840.376] (-5849.948) (-5830.978) * [-5826.480] (-5876.156) (-5827.686) (-5840.685) -- 11:07:54      789000 -- (-5832.717) [-5834.233] (-5826.676) (-5851.442) * (-5820.085) (-5860.214) (-5832.303) [-5816.628] -- 11:07:57      790000 -- (-5841.060) (-5837.974) [-5832.008] (-5842.316) * [-5826.766] (-5859.851) (-5840.010) (-5828.150) -- 11:07:49      Average standard deviation of split frequencies: 0.021676      791000 -- (-5841.099) (-5854.246) [-5831.341] (-5855.527) * [-5829.276] (-5857.163) (-5844.521) (-5821.628) -- 11:07:40      792000 -- (-5828.638) (-5830.419) [-5829.659] (-5864.211) * (-5843.090) [-5830.664] (-5863.212) (-5833.255) -- 11:07:32      793000 -- (-5852.023) (-5830.637) [-5821.024] (-5847.540) * [-5832.259] (-5836.898) (-5841.324) (-5840.833) -- 11:07:35      794000 -- (-5829.985) [-5829.641] (-5826.027) (-5842.363) * (-5856.280) (-5843.630) (-5857.681) [-5833.740] -- 11:07:27      795000 -- (-5850.197) [-5816.281] (-5821.795) (-5848.317) * (-5851.569) (-5855.565) (-5842.796) [-5827.921] -- 11:07:18      Average standard deviation of split frequencies: 0.021514      796000 -- (-5843.532) (-5822.362) (-5827.386) [-5827.495] * (-5849.669) (-5862.403) (-5845.616) [-5823.547] -- 11:07:10      797000 -- (-5846.558) (-5842.100) [-5827.845] (-5828.185) * [-5834.209] (-5872.048) (-5842.451) (-5842.668) -- 11:07:13      798000 -- (-5839.060) (-5843.753) (-5814.692) [-5813.981] * (-5836.954) (-5856.827) (-5849.245) [-5832.503] -- 11:07:05      799000 -- (-5831.821) (-5845.578) (-5835.003) [-5822.764] * (-5829.238) (-5864.005) (-5851.791) [-5828.787] -- 11:06:56      800000 -- [-5850.271] (-5836.021) (-5836.716) (-5831.094) * [-5825.097] (-5869.223) (-5869.427) (-5823.592) -- 11:06:48      Average standard deviation of split frequencies: 0.021210      801000 -- (-5846.301) [-5848.499] (-5843.041) (-5824.232) * [-5829.145] (-5847.861) (-5842.319) (-5832.724) -- 11:06:51      802000 -- (-5874.666) (-5846.446) (-5832.854) [-5822.787] * [-5830.574] (-5839.132) (-5852.288) (-5863.599) -- 11:06:43      803000 -- (-5834.308) (-5867.540) [-5823.611] (-5823.312) * (-5853.150) (-5844.174) (-5845.867) [-5834.901] -- 11:06:34      804000 -- (-5837.984) (-5846.915) [-5829.504] (-5825.660) * [-5825.870] (-5855.971) (-5831.785) (-5843.395) -- 11:06:26      805000 -- (-5842.745) (-5838.218) (-5836.708) [-5820.694] * [-5832.872] (-5855.197) (-5828.258) (-5837.505) -- 11:06:29      Average standard deviation of split frequencies: 0.021410      806000 -- [-5836.366] (-5850.029) (-5840.219) (-5843.570) * (-5829.769) (-5849.792) (-5846.925) [-5823.875] -- 11:06:21      807000 -- [-5836.295] (-5841.368) (-5842.095) (-5843.041) * (-5826.745) (-5857.117) (-5839.154) [-5834.068] -- 11:06:13      808000 -- (-5851.737) (-5834.097) (-5835.826) [-5840.919] * (-5837.316) (-5847.672) [-5825.705] (-5848.106) -- 11:06:04      809000 -- (-5842.935) (-5828.184) [-5834.635] (-5857.717) * (-5838.283) (-5833.019) [-5827.196] (-5846.606) -- 11:06:07      810000 -- (-5847.660) [-5839.351] (-5816.834) (-5841.291) * (-5833.468) [-5833.437] (-5842.046) (-5835.366) -- 11:05:59      Average standard deviation of split frequencies: 0.021174      811000 -- (-5861.595) [-5840.032] (-5826.954) (-5845.497) * [-5822.837] (-5833.808) (-5827.794) (-5860.283) -- 11:05:51      812000 -- (-5883.148) (-5856.147) (-5848.790) [-5835.882] * [-5822.552] (-5845.588) (-5840.416) (-5868.049) -- 11:05:54      813000 -- (-5863.318) (-5842.694) (-5837.811) [-5819.311] * [-5831.848] (-5837.261) (-5833.346) (-5826.334) -- 11:05:45      814000 -- (-5862.828) [-5832.037] (-5830.181) (-5835.708) * (-5835.744) (-5851.303) [-5835.614] (-5849.140) -- 11:05:37      815000 -- (-5860.213) [-5835.710] (-5829.050) (-5832.080) * (-5840.096) [-5823.080] (-5842.811) (-5852.307) -- 11:05:40      Average standard deviation of split frequencies: 0.020934      816000 -- (-5858.764) (-5847.917) (-5841.562) [-5821.820] * [-5833.173] (-5835.084) (-5849.805) (-5861.169) -- 11:05:32      817000 -- (-5863.597) [-5837.562] (-5854.353) (-5833.087) * (-5840.485) (-5835.898) [-5833.700] (-5863.699) -- 11:05:24      818000 -- (-5842.572) [-5827.130] (-5849.938) (-5833.245) * (-5846.967) [-5823.850] (-5833.163) (-5854.446) -- 11:05:27      819000 -- (-5841.759) [-5814.809] (-5845.688) (-5842.512) * (-5840.093) [-5816.525] (-5854.071) (-5843.482) -- 11:05:18      820000 -- [-5826.565] (-5823.751) (-5866.996) (-5854.695) * (-5856.703) [-5815.239] (-5846.097) (-5833.274) -- 11:05:10      Average standard deviation of split frequencies: 0.020057      821000 -- (-5848.886) [-5828.217] (-5841.661) (-5842.973) * (-5852.328) [-5807.793] (-5837.840) (-5833.833) -- 11:05:02      822000 -- (-5844.303) (-5825.173) [-5831.657] (-5827.348) * (-5832.632) [-5816.208] (-5845.578) (-5862.235) -- 11:05:05      823000 -- (-5861.047) (-5836.109) [-5825.612] (-5844.919) * (-5853.746) [-5810.547] (-5830.861) (-5847.718) -- 11:04:57      824000 -- (-5836.261) (-5841.247) [-5813.039] (-5840.333) * (-5856.422) [-5820.172] (-5839.770) (-5875.822) -- 11:04:48      825000 -- [-5838.535] (-5842.027) (-5830.751) (-5862.378) * (-5848.792) (-5829.192) [-5837.861] (-5857.180) -- 11:04:51      Average standard deviation of split frequencies: 0.019814      826000 -- [-5832.973] (-5837.602) (-5854.036) (-5863.533) * (-5841.023) [-5825.041] (-5845.162) (-5833.460) -- 11:04:43      827000 -- [-5825.478] (-5826.815) (-5849.108) (-5872.659) * (-5818.381) [-5847.517] (-5850.810) (-5859.155) -- 11:04:35      828000 -- (-5841.581) (-5848.489) [-5836.090] (-5857.054) * (-5838.395) (-5837.715) [-5826.239] (-5832.419) -- 11:04:27      829000 -- (-5858.713) [-5841.865] (-5847.233) (-5865.073) * (-5853.818) [-5822.042] (-5832.133) (-5848.945) -- 11:04:30      830000 -- (-5873.475) [-5831.014] (-5849.245) (-5830.783) * (-5837.682) [-5834.453] (-5844.368) (-5861.609) -- 11:04:21      Average standard deviation of split frequencies: 0.019728      831000 -- [-5829.701] (-5835.278) (-5863.427) (-5833.372) * (-5859.272) (-5820.409) [-5829.089] (-5853.905) -- 11:04:13      832000 -- (-5840.280) (-5820.068) (-5864.309) [-5815.892] * (-5836.829) (-5839.969) [-5834.818] (-5872.622) -- 11:04:16      833000 -- (-5839.586) [-5822.105] (-5850.220) (-5837.653) * [-5835.242] (-5827.942) (-5832.254) (-5851.265) -- 11:04:08      834000 -- (-5832.426) (-5826.444) (-5857.519) [-5814.205] * (-5850.501) [-5817.415] (-5839.012) (-5853.496) -- 11:04:00      835000 -- (-5838.852) [-5831.001] (-5853.167) (-5813.518) * [-5832.704] (-5830.791) (-5862.703) (-5847.404) -- 11:03:52      Average standard deviation of split frequencies: 0.019972      836000 -- (-5835.641) [-5836.320] (-5858.251) (-5836.444) * (-5857.693) [-5818.516] (-5840.894) (-5822.548) -- 11:03:54      837000 -- (-5854.185) (-5864.473) (-5849.406) [-5832.327] * (-5862.971) [-5843.909] (-5839.585) (-5848.307) -- 11:03:46      838000 -- (-5847.753) (-5835.689) (-5841.011) [-5822.338] * (-5844.932) [-5847.627] (-5846.867) (-5833.032) -- 11:03:38      839000 -- (-5851.423) (-5839.609) [-5835.334] (-5832.649) * [-5824.291] (-5842.770) (-5839.144) (-5825.807) -- 11:03:30      840000 -- (-5856.081) (-5851.186) (-5855.075) [-5832.359] * (-5827.750) (-5864.651) (-5849.039) [-5816.111] -- 11:03:33      Average standard deviation of split frequencies: 0.020426      841000 -- (-5859.642) (-5855.495) (-5858.539) [-5828.841] * (-5840.575) (-5840.469) (-5829.121) [-5816.791] -- 11:03:25      842000 -- (-5844.289) (-5845.320) (-5864.440) [-5824.546] * (-5856.558) (-5831.830) (-5828.622) [-5831.570] -- 11:03:17      843000 -- (-5838.658) [-5822.034] (-5840.069) (-5841.567) * (-5827.718) (-5848.527) [-5820.732] (-5852.263) -- 11:03:08      844000 -- (-5860.139) (-5838.000) (-5841.006) [-5835.835] * (-5837.750) (-5859.360) [-5819.473] (-5866.346) -- 11:03:11      845000 -- (-5858.875) (-5857.517) (-5847.732) [-5839.995] * [-5814.331] (-5845.760) (-5813.879) (-5851.941) -- 11:03:03      Average standard deviation of split frequencies: 0.020601      846000 -- (-5845.222) (-5866.770) (-5852.869) [-5827.765] * [-5829.929] (-5850.469) (-5833.953) (-5840.467) -- 11:02:55      847000 -- [-5829.932] (-5854.968) (-5867.246) (-5825.389) * (-5828.325) (-5846.153) [-5831.084] (-5838.648) -- 11:02:58      848000 -- [-5816.263] (-5861.929) (-5865.920) (-5821.748) * [-5828.660] (-5852.133) (-5829.513) (-5848.654) -- 11:02:50      849000 -- [-5817.311] (-5848.998) (-5832.464) (-5841.296) * [-5812.396] (-5853.319) (-5850.293) (-5842.813) -- 11:02:42      850000 -- (-5822.926) (-5860.784) [-5832.686] (-5845.947) * [-5821.877] (-5856.484) (-5869.920) (-5842.193) -- 11:02:34      Average standard deviation of split frequencies: 0.020774      851000 -- [-5816.774] (-5859.684) (-5822.548) (-5846.857) * [-5817.200] (-5872.518) (-5844.518) (-5826.070) -- 11:02:36      852000 -- [-5838.601] (-5855.829) (-5826.575) (-5847.578) * [-5838.493] (-5852.756) (-5847.376) (-5837.195) -- 11:02:28      853000 -- (-5831.439) (-5860.632) (-5832.813) [-5833.833] * (-5830.428) (-5857.718) (-5840.825) [-5833.366] -- 11:02:20      854000 -- (-5839.518) (-5855.201) [-5809.488] (-5834.309) * (-5822.096) (-5848.355) (-5839.944) [-5827.550] -- 11:02:12      855000 -- (-5835.588) (-5828.377) [-5814.686] (-5848.802) * [-5812.748] (-5854.011) (-5862.156) (-5829.195) -- 11:02:04      Average standard deviation of split frequencies: 0.021047      856000 -- (-5839.333) [-5830.439] (-5844.171) (-5835.608) * [-5823.589] (-5838.231) (-5839.865) (-5824.770) -- 11:02:07      857000 -- (-5844.191) [-5819.906] (-5875.864) (-5858.564) * [-5828.709] (-5853.761) (-5821.236) (-5812.836) -- 11:01:59      858000 -- (-5847.297) [-5824.754] (-5847.295) (-5859.303) * (-5853.124) (-5834.968) (-5835.161) [-5831.732] -- 11:01:51      859000 -- [-5843.809] (-5818.504) (-5858.909) (-5845.466) * (-5840.567) (-5852.191) (-5834.472) [-5829.744] -- 11:01:43      860000 -- [-5820.815] (-5819.652) (-5860.759) (-5851.859) * (-5826.321) (-5843.411) (-5856.465) [-5832.145] -- 11:01:35      Average standard deviation of split frequencies: 0.020648      861000 -- (-5823.250) (-5839.226) (-5840.604) [-5843.071] * (-5827.416) (-5851.825) (-5829.861) [-5826.909] -- 11:01:37      862000 -- [-5827.629] (-5841.106) (-5845.869) (-5834.410) * [-5831.979] (-5871.685) (-5833.879) (-5826.939) -- 11:01:29      863000 -- [-5835.539] (-5815.921) (-5857.405) (-5843.346) * [-5823.809] (-5862.079) (-5838.856) (-5824.444) -- 11:01:21      864000 -- [-5821.384] (-5820.832) (-5868.177) (-5848.623) * (-5833.434) (-5842.763) (-5868.513) [-5816.856] -- 11:01:13      865000 -- [-5820.960] (-5844.812) (-5855.466) (-5859.950) * (-5843.530) (-5827.952) (-5846.195) [-5825.268] -- 11:01:16      Average standard deviation of split frequencies: 0.020541      866000 -- (-5847.237) (-5824.527) [-5826.952] (-5869.721) * (-5846.667) (-5831.680) [-5844.449] (-5844.930) -- 11:01:08      867000 -- (-5846.732) [-5819.398] (-5840.986) (-5852.540) * (-5857.229) (-5841.557) (-5842.503) [-5830.258] -- 11:01:00      868000 -- (-5836.498) [-5831.719] (-5854.466) (-5855.523) * (-5872.620) [-5826.263] (-5828.589) (-5839.434) -- 11:00:52      869000 -- (-5827.245) (-5841.557) [-5829.842] (-5851.874) * (-5848.861) (-5840.971) (-5846.366) [-5832.125] -- 11:00:55      870000 -- (-5824.425) [-5827.916] (-5876.945) (-5864.779) * (-5846.159) (-5838.871) (-5862.680) [-5837.086] -- 11:00:47      Average standard deviation of split frequencies: 0.020258      871000 -- (-5825.519) [-5840.069] (-5855.312) (-5859.975) * (-5863.603) (-5830.312) (-5838.242) [-5816.523] -- 11:00:39      872000 -- [-5824.116] (-5843.319) (-5856.347) (-5830.628) * (-5864.380) [-5824.632] (-5842.602) (-5839.857) -- 11:00:31      873000 -- (-5810.585) [-5832.231] (-5839.020) (-5833.492) * (-5863.628) (-5842.769) [-5840.568] (-5831.670) -- 11:00:33      874000 -- [-5826.380] (-5850.573) (-5828.889) (-5826.479) * (-5828.356) (-5856.424) (-5841.339) [-5834.453] -- 11:00:26      875000 -- [-5826.070] (-5850.714) (-5848.844) (-5830.509) * [-5819.775] (-5848.318) (-5829.544) (-5847.296) -- 11:00:18      Average standard deviation of split frequencies: 0.019960      876000 -- (-5821.933) [-5831.304] (-5848.504) (-5860.545) * (-5841.063) [-5831.997] (-5847.069) (-5854.681) -- 11:00:20      877000 -- [-5821.355] (-5833.518) (-5816.676) (-5850.819) * (-5851.869) [-5821.204] (-5845.038) (-5847.348) -- 11:00:12      878000 -- (-5831.472) (-5836.158) [-5815.795] (-5850.196) * (-5848.213) [-5828.511] (-5853.881) (-5847.377) -- 11:00:04      879000 -- (-5820.929) (-5832.016) [-5836.692] (-5849.796) * (-5848.999) (-5841.813) (-5854.761) [-5838.239] -- 11:00:07      880000 -- (-5823.751) (-5835.228) [-5820.508] (-5864.628) * (-5836.095) (-5849.755) (-5843.617) [-5841.946] -- 10:59:59      Average standard deviation of split frequencies: 0.020139      881000 -- [-5821.046] (-5834.752) (-5836.458) (-5844.494) * (-5837.165) [-5831.773] (-5839.931) (-5852.862) -- 10:59:51      882000 -- [-5811.406] (-5845.468) (-5823.441) (-5853.100) * (-5834.565) [-5816.142] (-5825.949) (-5855.731) -- 10:59:43      883000 -- (-5830.243) (-5856.496) [-5822.945] (-5840.257) * (-5843.504) [-5817.249] (-5816.766) (-5853.717) -- 10:59:35      884000 -- (-5836.546) (-5834.067) [-5829.559] (-5837.364) * (-5847.640) (-5831.705) [-5810.625] (-5832.673) -- 10:59:38      885000 -- [-5834.391] (-5867.324) (-5836.161) (-5843.019) * (-5859.121) (-5825.019) [-5833.204] (-5833.237) -- 10:59:30      Average standard deviation of split frequencies: 0.020106      886000 -- [-5839.115] (-5842.501) (-5843.748) (-5850.566) * (-5862.896) (-5833.582) [-5825.704] (-5829.048) -- 10:59:22      887000 -- (-5839.363) [-5827.715] (-5842.705) (-5842.832) * (-5849.492) (-5826.332) [-5821.689] (-5840.645) -- 10:59:14      888000 -- [-5821.851] (-5832.370) (-5850.214) (-5867.096) * (-5859.526) (-5851.642) [-5828.084] (-5833.823) -- 10:59:06      889000 -- [-5811.081] (-5843.823) (-5840.952) (-5858.944) * (-5859.037) (-5846.807) (-5840.977) [-5816.104] -- 10:59:09      890000 -- [-5832.023] (-5839.827) (-5847.457) (-5855.082) * (-5859.375) [-5831.553] (-5848.164) (-5830.277) -- 10:59:01      Average standard deviation of split frequencies: 0.020188      891000 -- (-5830.242) [-5830.339] (-5846.913) (-5832.629) * (-5869.129) [-5835.911] (-5835.835) (-5837.414) -- 10:59:14      892000 -- [-5825.229] (-5856.944) (-5845.510) (-5848.353) * (-5834.308) [-5833.788] (-5845.125) (-5839.927) -- 10:59:06      893000 -- (-5838.068) [-5840.421] (-5840.740) (-5858.066) * (-5824.625) (-5827.793) (-5843.829) [-5820.856] -- 10:59:08      894000 -- (-5861.360) (-5843.476) [-5845.546] (-5862.156) * (-5857.964) [-5826.962] (-5850.235) (-5835.832) -- 10:59:00      895000 -- (-5862.632) (-5847.968) [-5832.744] (-5843.231) * (-5850.346) (-5834.333) (-5844.518) [-5820.489] -- 10:59:03      Average standard deviation of split frequencies: 0.019780      896000 -- (-5846.249) [-5827.093] (-5847.032) (-5819.364) * (-5852.146) [-5826.671] (-5853.577) (-5837.745) -- 10:58:55      897000 -- [-5832.445] (-5844.801) (-5838.998) (-5834.025) * (-5837.793) (-5842.287) (-5861.547) [-5828.752] -- 10:58:47      898000 -- (-5841.511) (-5847.492) [-5851.480] (-5829.112) * [-5829.153] (-5843.980) (-5845.436) (-5825.358) -- 10:58:49      899000 -- [-5831.280] (-5853.828) (-5836.621) (-5824.134) * (-5822.425) (-5841.188) (-5847.738) [-5830.834] -- 10:58:42      900000 -- (-5823.708) (-5869.167) [-5838.752] (-5841.462) * (-5830.300) [-5828.232] (-5834.931) (-5868.525) -- 10:58:34      Average standard deviation of split frequencies: 0.019570      901000 -- [-5829.332] (-5880.476) (-5844.733) (-5833.658) * [-5832.293] (-5829.596) (-5865.209) (-5859.649) -- 10:58:26      902000 -- (-5822.843) (-5873.637) [-5836.260] (-5837.461) * (-5830.790) [-5815.177] (-5840.887) (-5873.686) -- 10:58:28      903000 -- [-5848.051] (-5854.017) (-5840.630) (-5845.640) * (-5826.061) [-5814.388] (-5853.043) (-5850.595) -- 10:58:20      904000 -- (-5835.324) (-5863.413) [-5847.154] (-5842.406) * (-5838.367) [-5816.212] (-5856.102) (-5850.280) -- 10:58:13      905000 -- [-5823.335] (-5870.950) (-5824.862) (-5830.363) * [-5814.233] (-5823.386) (-5861.589) (-5858.014) -- 10:58:05      Average standard deviation of split frequencies: 0.019336      906000 -- (-5831.364) [-5841.272] (-5835.977) (-5848.698) * [-5805.619] (-5851.569) (-5835.702) (-5867.861) -- 10:58:07      907000 -- (-5837.575) (-5864.831) [-5814.134] (-5848.361) * [-5810.752] (-5852.293) (-5842.166) (-5833.250) -- 10:58:09      908000 -- (-5846.468) (-5884.684) [-5831.284] (-5848.150) * [-5819.284] (-5825.332) (-5849.187) (-5844.777) -- 10:58:02      909000 -- (-5828.086) (-5883.681) (-5833.753) [-5817.781] * [-5821.904] (-5849.183) (-5837.836) (-5833.354) -- 10:58:04      910000 -- (-5834.354) (-5871.060) [-5830.130] (-5819.638) * (-5824.792) (-5851.786) (-5865.290) [-5818.715] -- 10:57:56      Average standard deviation of split frequencies: 0.019266      911000 -- (-5843.281) (-5861.168) (-5836.651) [-5814.039] * [-5821.563] (-5848.676) (-5857.466) (-5826.532) -- 10:57:48      912000 -- [-5823.444] (-5844.464) (-5826.532) (-5831.953) * (-5831.591) (-5843.787) (-5858.099) [-5831.278] -- 10:57:41      913000 -- (-5823.288) (-5840.206) [-5808.424] (-5819.004) * (-5839.503) (-5865.081) (-5864.846) [-5823.841] -- 10:57:43      914000 -- (-5824.188) (-5835.589) (-5811.432) [-5833.376] * [-5831.795] (-5837.125) (-5854.027) (-5825.911) -- 10:57:35      915000 -- (-5828.784) (-5840.059) [-5827.616] (-5831.309) * (-5837.751) (-5845.152) (-5843.395) [-5809.252] -- 10:57:27      Average standard deviation of split frequencies: 0.019041      916000 -- [-5826.559] (-5839.258) (-5827.361) (-5857.975) * (-5850.339) (-5837.118) (-5831.060) [-5812.795] -- 10:57:20      917000 -- (-5827.940) [-5840.192] (-5809.532) (-5875.991) * (-5856.941) (-5838.661) [-5828.997] (-5825.104) -- 10:57:22      918000 -- (-5838.549) [-5820.974] (-5836.440) (-5870.449) * (-5880.236) (-5853.380) [-5814.021] (-5825.829) -- 10:57:14      919000 -- [-5818.962] (-5822.459) (-5849.348) (-5854.918) * (-5853.515) (-5844.629) [-5829.383] (-5855.532) -- 10:57:06      920000 -- (-5817.039) [-5816.473] (-5842.161) (-5841.379) * [-5827.763] (-5833.040) (-5835.491) (-5833.521) -- 10:57:08      Average standard deviation of split frequencies: 0.019229      921000 -- (-5814.415) [-5824.557] (-5840.192) (-5844.476) * [-5818.548] (-5849.734) (-5849.504) (-5841.812) -- 10:57:01      922000 -- [-5831.025] (-5839.158) (-5847.214) (-5845.923) * [-5820.127] (-5850.306) (-5846.985) (-5841.058) -- 10:56:53      923000 -- (-5828.490) [-5829.633] (-5826.469) (-5823.008) * (-5825.824) (-5864.624) (-5842.041) [-5834.443] -- 10:56:55      924000 -- (-5829.630) [-5827.692] (-5840.016) (-5848.085) * (-5850.487) (-5844.582) (-5850.116) [-5836.751] -- 10:56:47      925000 -- (-5836.465) [-5833.350] (-5840.704) (-5852.486) * (-5862.838) (-5831.537) (-5843.587) [-5834.863] -- 10:56:40      Average standard deviation of split frequencies: 0.018517      926000 -- (-5828.324) (-5843.467) [-5826.365] (-5831.156) * (-5869.411) (-5856.120) (-5836.766) [-5819.243] -- 10:56:42      927000 -- [-5813.657] (-5843.427) (-5837.733) (-5843.011) * (-5846.814) (-5834.825) [-5814.562] (-5825.636) -- 10:56:34      928000 -- [-5810.752] (-5835.056) (-5831.559) (-5851.107) * (-5866.378) [-5841.049] (-5851.418) (-5841.069) -- 10:56:36      929000 -- (-5825.240) [-5829.270] (-5820.850) (-5842.967) * (-5850.097) (-5853.011) (-5847.652) [-5830.678] -- 10:56:38      930000 -- [-5812.926] (-5822.667) (-5831.791) (-5837.830) * [-5842.893] (-5859.098) (-5842.558) (-5828.929) -- 10:56:31      Average standard deviation of split frequencies: 0.018521      931000 -- [-5809.093] (-5825.537) (-5846.032) (-5844.260) * (-5842.507) (-5847.626) (-5836.289) [-5828.399] -- 10:56:23      932000 -- [-5813.626] (-5840.407) (-5841.379) (-5849.682) * (-5838.639) (-5832.671) (-5851.089) [-5818.693] -- 10:56:15      933000 -- (-5829.708) (-5827.010) [-5831.589] (-5847.450) * (-5829.257) (-5830.673) (-5845.250) [-5832.364] -- 10:56:17      934000 -- [-5835.628] (-5831.335) (-5856.716) (-5836.226) * (-5831.657) (-5846.708) [-5839.928] (-5826.060) -- 10:56:10      935000 -- (-5840.201) [-5817.449] (-5856.811) (-5826.805) * (-5839.401) [-5833.237] (-5838.977) (-5830.610) -- 10:56:02      Average standard deviation of split frequencies: 0.018589      936000 -- (-5836.369) [-5836.162] (-5846.569) (-5832.326) * (-5831.258) [-5823.283] (-5846.063) (-5821.165) -- 10:56:14      937000 -- [-5843.492] (-5838.044) (-5858.682) (-5857.926) * (-5824.371) (-5839.190) (-5839.099) [-5825.891] -- 10:56:06      938000 -- (-5840.042) [-5840.449] (-5845.330) (-5852.391) * (-5834.358) (-5841.045) (-5842.798) [-5826.490] -- 10:55:58      939000 -- [-5825.540] (-5841.845) (-5840.395) (-5853.569) * (-5854.042) (-5835.011) [-5816.556] (-5841.934) -- 10:56:00      940000 -- [-5823.201] (-5847.459) (-5838.366) (-5841.268) * (-5847.726) (-5851.309) [-5831.148] (-5833.678) -- 10:55:53      Average standard deviation of split frequencies: 0.018702      941000 -- (-5832.955) (-5835.614) (-5833.458) [-5834.256] * (-5860.468) [-5822.573] (-5835.435) (-5814.770) -- 10:55:45      942000 -- [-5824.279] (-5845.828) (-5861.589) (-5834.318) * (-5855.087) (-5837.917) (-5824.254) [-5818.660] -- 10:55:47      943000 -- [-5820.078] (-5830.785) (-5847.753) (-5830.134) * (-5848.481) (-5851.461) [-5824.079] (-5816.420) -- 10:55:39      944000 -- [-5822.274] (-5842.800) (-5836.087) (-5835.909) * (-5841.836) (-5830.741) [-5815.374] (-5856.636) -- 10:55:41      945000 -- [-5817.920] (-5824.269) (-5842.346) (-5849.705) * (-5843.385) (-5857.949) [-5818.937] (-5859.911) -- 10:55:34      Average standard deviation of split frequencies: 0.018615      946000 -- [-5825.600] (-5834.327) (-5859.122) (-5848.345) * (-5831.201) (-5868.782) [-5836.621] (-5837.967) -- 10:55:36      947000 -- [-5835.777] (-5836.401) (-5856.476) (-5846.835) * (-5840.327) (-5869.697) [-5831.593] (-5834.096) -- 10:55:28      948000 -- (-5845.551) [-5823.740] (-5836.954) (-5840.902) * (-5829.245) (-5861.049) [-5825.047] (-5837.959) -- 10:55:20      949000 -- (-5854.828) (-5840.327) [-5852.521] (-5851.265) * (-5842.294) (-5862.125) [-5818.762] (-5820.622) -- 10:55:13      950000 -- [-5828.540] (-5845.775) (-5868.278) (-5835.990) * [-5826.402] (-5874.737) (-5820.483) (-5831.930) -- 10:55:15      Average standard deviation of split frequencies: 0.018753      951000 -- (-5844.122) (-5843.245) [-5861.421] (-5839.101) * [-5827.314] (-5855.491) (-5833.097) (-5822.787) -- 10:55:07      952000 -- (-5870.001) [-5824.867] (-5850.019) (-5847.622) * (-5839.289) (-5852.847) (-5825.972) [-5835.287] -- 10:54:59      953000 -- (-5857.518) [-5821.422] (-5851.142) (-5816.570) * [-5828.101] (-5851.552) (-5852.710) (-5837.089) -- 10:55:01      954000 -- (-5844.932) (-5830.696) (-5836.391) [-5822.496] * [-5838.680] (-5852.201) (-5837.785) (-5825.110) -- 10:54:54      955000 -- (-5845.662) [-5830.654] (-5829.267) (-5834.878) * (-5822.122) (-5847.843) [-5835.240] (-5831.956) -- 10:54:46      Average standard deviation of split frequencies: 0.018837      956000 -- (-5868.136) (-5839.176) (-5833.434) [-5837.385] * [-5814.604] (-5855.497) (-5840.183) (-5856.792) -- 10:54:48      957000 -- (-5868.895) (-5830.479) (-5841.635) [-5815.875] * (-5808.574) (-5854.890) (-5838.604) [-5827.673] -- 10:54:40      958000 -- (-5866.880) [-5832.698] (-5852.206) (-5828.510) * [-5814.137] (-5829.460) (-5865.442) (-5843.664) -- 10:54:33      959000 -- (-5847.655) (-5859.118) (-5843.631) [-5823.135] * (-5820.544) (-5848.274) (-5883.551) [-5817.490] -- 10:54:25      960000 -- (-5866.415) (-5836.192) (-5827.982) [-5826.596] * (-5839.221) [-5837.598] (-5858.328) (-5846.442) -- 10:54:27      Average standard deviation of split frequencies: 0.018823      961000 -- (-5852.656) (-5848.020) (-5836.792) [-5820.247] * (-5843.493) (-5828.411) (-5878.178) [-5831.629] -- 10:54:19      962000 -- (-5863.056) (-5848.267) (-5840.738) [-5826.572] * (-5848.987) [-5833.493] (-5857.568) (-5839.261) -- 10:54:12      963000 -- (-5850.705) (-5836.864) [-5820.640] (-5828.048) * (-5844.430) (-5824.604) (-5838.602) [-5834.719] -- 10:54:04      964000 -- (-5826.713) [-5815.557] (-5834.941) (-5831.956) * (-5830.675) (-5836.995) [-5833.544] (-5839.559) -- 10:54:06      965000 -- [-5816.941] (-5829.298) (-5843.804) (-5841.670) * (-5874.260) [-5819.730] (-5827.782) (-5838.856) -- 10:53:59      Average standard deviation of split frequencies: 0.018389      966000 -- (-5834.961) [-5821.955] (-5860.211) (-5828.762) * (-5875.425) (-5835.051) [-5832.288] (-5840.331) -- 10:53:51      967000 -- (-5842.621) [-5840.055] (-5862.667) (-5828.467) * (-5877.557) (-5848.126) (-5828.744) [-5827.914] -- 10:53:53      968000 -- (-5856.618) (-5831.601) (-5854.923) [-5822.128] * (-5858.762) (-5839.913) [-5829.166] (-5834.331) -- 10:53:55      969000 -- (-5858.052) (-5837.789) (-5838.412) [-5819.368] * (-5842.710) (-5821.485) [-5833.859] (-5837.873) -- 10:53:47      970000 -- (-5839.604) (-5871.255) (-5830.392) [-5811.243] * (-5851.650) [-5831.437] (-5849.077) (-5835.792) -- 10:53:49      Average standard deviation of split frequencies: 0.018447      971000 -- (-5836.043) (-5861.201) (-5823.676) [-5821.157] * (-5838.558) [-5814.515] (-5865.268) (-5839.802) -- 10:53:41      972000 -- (-5839.478) (-5864.040) (-5831.162) [-5822.818] * (-5831.267) [-5832.815] (-5849.302) (-5853.306) -- 10:53:43      973000 -- (-5820.364) (-5864.813) [-5828.613] (-5841.408) * (-5836.393) [-5824.337] (-5835.094) (-5844.692) -- 10:53:35      974000 -- (-5820.627) (-5858.377) [-5826.586] (-5842.864) * (-5838.868) [-5808.467] (-5857.917) (-5851.634) -- 10:53:28      975000 -- [-5825.987] (-5860.790) (-5823.647) (-5841.069) * [-5837.981] (-5836.059) (-5851.349) (-5861.787) -- 10:53:30      Average standard deviation of split frequencies: 0.018522      976000 -- [-5826.795] (-5878.332) (-5834.104) (-5835.596) * (-5842.752) [-5813.787] (-5854.530) (-5830.165) -- 10:53:22      977000 -- [-5818.846] (-5849.139) (-5859.456) (-5829.701) * (-5861.574) (-5818.798) (-5847.602) [-5827.874] -- 10:53:15      978000 -- [-5819.986] (-5864.898) (-5833.939) (-5831.459) * (-5846.268) [-5831.910] (-5865.391) (-5844.605) -- 10:53:16      979000 -- [-5819.156] (-5845.447) (-5824.513) (-5846.345) * (-5834.952) [-5822.885] (-5855.081) (-5863.179) -- 10:53:09      980000 -- [-5813.260] (-5841.929) (-5849.268) (-5855.230) * (-5829.480) [-5820.340] (-5863.249) (-5840.598) -- 10:53:10      Average standard deviation of split frequencies: 0.018633      981000 -- (-5822.249) (-5835.788) (-5832.001) [-5832.060] * [-5833.682] (-5853.106) (-5864.702) (-5868.648) -- 10:53:03      982000 -- [-5833.438] (-5853.563) (-5844.941) (-5828.978) * [-5821.729] (-5855.813) (-5836.184) (-5845.368) -- 10:52:55      983000 -- (-5827.953) (-5847.007) (-5847.304) [-5825.750] * [-5814.601] (-5847.050) (-5844.587) (-5867.421) -- 10:52:57      984000 -- (-5839.195) (-5858.646) (-5847.446) [-5832.990] * (-5825.032) (-5845.652) (-5844.049) [-5839.910] -- 10:52:59      985000 -- [-5816.093] (-5862.690) (-5840.587) (-5837.174) * [-5823.797] (-5856.181) (-5864.571) (-5842.427) -- 10:52:51      Average standard deviation of split frequencies: 0.018734      986000 -- [-5821.347] (-5858.860) (-5858.602) (-5824.100) * [-5826.800] (-5840.561) (-5876.619) (-5834.492) -- 10:52:53      987000 -- [-5813.548] (-5858.486) (-5849.134) (-5828.198) * (-5833.052) (-5844.620) (-5842.891) [-5832.940] -- 10:52:45      988000 -- [-5818.355] (-5841.755) (-5868.628) (-5840.844) * (-5840.992) (-5864.422) [-5834.900] (-5844.409) -- 10:52:47      989000 -- [-5821.671] (-5839.624) (-5852.368) (-5838.139) * (-5841.239) (-5870.482) [-5832.202] (-5843.414) -- 10:52:40      990000 -- (-5811.108) [-5816.859] (-5845.555) (-5853.870) * [-5822.152] (-5861.719) (-5831.472) (-5830.628) -- 10:52:32      Average standard deviation of split frequencies: 0.018494      991000 -- (-5838.865) [-5817.642] (-5834.119) (-5854.403) * (-5818.280) (-5838.617) (-5846.824) [-5825.339] -- 10:52:34      992000 -- (-5865.913) (-5816.945) [-5835.842] (-5837.595) * [-5828.286] (-5840.804) (-5856.394) (-5836.208) -- 10:52:26      993000 -- (-5844.620) (-5836.086) [-5824.270] (-5821.570) * [-5814.348] (-5847.079) (-5854.299) (-5846.659) -- 10:52:19      994000 -- [-5835.571] (-5844.734) (-5823.470) (-5850.175) * [-5829.583] (-5834.875) (-5869.436) (-5848.318) -- 10:52:20      995000 -- (-5854.734) (-5839.840) (-5813.809) [-5825.729] * [-5827.311] (-5831.466) (-5845.722) (-5870.851) -- 10:52:22      Average standard deviation of split frequencies: 0.018515      996000 -- [-5823.592] (-5840.523) (-5822.869) (-5849.128) * (-5853.032) [-5823.549] (-5840.047) (-5838.186) -- 10:52:14      997000 -- (-5832.209) [-5813.789] (-5835.654) (-5848.749) * (-5867.181) [-5818.618] (-5848.495) (-5830.760) -- 10:52:16      998000 -- (-5818.541) (-5822.739) [-5820.795] (-5827.734) * (-5850.393) (-5836.020) (-5851.489) [-5832.347] -- 10:52:08      999000 -- [-5827.582] (-5827.863) (-5818.449) (-5851.126) * (-5853.627) [-5838.511] (-5851.336) (-5851.716) -- 10:52:01      1000000 -- [-5813.071] (-5842.676) (-5821.114) (-5836.030) * (-5840.795) (-5834.263) (-5853.646) [-5836.328] -- 10:52:03      Average standard deviation of split frequencies: 0.018529      1001000 -- (-5817.544) (-5840.182) [-5823.312] (-5857.668) * (-5825.346) (-5828.337) (-5852.756) [-5844.160] -- 10:51:55      1002000 -- (-5837.239) [-5823.465] (-5853.566) (-5867.323) * (-5845.843) [-5833.120] (-5838.134) (-5839.604) -- 10:51:48      1003000 -- (-5855.851) [-5821.456] (-5828.552) (-5876.189) * (-5861.000) (-5832.734) (-5848.580) [-5816.644] -- 10:51:40      1004000 -- (-5855.655) [-5826.611] (-5840.818) (-5849.342) * (-5856.163) (-5843.671) (-5846.113) [-5817.604] -- 10:51:42      1005000 -- (-5850.865) [-5822.241] (-5848.388) (-5839.455) * (-5848.394) [-5825.853] (-5842.300) (-5830.451) -- 10:51:34      Average standard deviation of split frequencies: 0.018306      1006000 -- (-5844.388) [-5833.298] (-5837.078) (-5839.329) * (-5845.325) [-5823.630] (-5840.545) (-5818.149) -- 10:51:27      1007000 -- (-5852.797) (-5823.809) (-5826.006) [-5828.298] * (-5844.989) (-5834.501) [-5829.375] (-5839.451) -- 10:51:28      1008000 -- (-5856.687) (-5837.865) (-5820.871) [-5822.080] * (-5837.286) (-5826.346) [-5840.749] (-5849.668) -- 10:51:30      1009000 -- (-5839.168) (-5836.904) (-5830.406) [-5803.358] * (-5842.384) (-5854.349) (-5831.008) [-5831.616] -- 10:51:22      1010000 -- [-5830.949] (-5859.172) (-5837.303) (-5821.567) * [-5828.806] (-5845.174) (-5831.466) (-5840.034) -- 10:51:24      Average standard deviation of split frequencies: 0.018328      1011000 -- (-5856.038) (-5828.590) (-5842.820) [-5804.779] * [-5833.319] (-5845.982) (-5849.922) (-5819.413) -- 10:51:16      1012000 -- (-5863.441) (-5846.743) (-5830.291) [-5818.508] * (-5837.813) (-5851.953) (-5829.343) [-5825.492] -- 10:51:09      1013000 -- (-5880.949) (-5837.848) (-5823.405) [-5809.906] * (-5840.262) (-5842.971) (-5845.635) [-5817.556] -- 10:51:10      1014000 -- (-5858.111) (-5839.709) [-5827.792] (-5825.382) * (-5837.428) (-5857.603) [-5833.832] (-5819.947) -- 10:51:03      1015000 -- (-5850.186) (-5838.491) [-5818.025] (-5860.895) * (-5838.337) (-5854.186) [-5832.862] (-5837.713) -- 10:51:04      Average standard deviation of split frequencies: 0.018391      1016000 -- (-5858.856) [-5830.113] (-5820.240) (-5858.242) * (-5831.614) (-5844.291) (-5850.066) [-5831.496] -- 10:50:57      1017000 -- (-5865.622) (-5822.358) [-5816.194] (-5865.185) * (-5839.345) (-5824.667) (-5843.346) [-5816.116] -- 10:50:58      1018000 -- (-5853.578) (-5827.937) [-5820.119] (-5832.418) * (-5850.307) (-5812.356) [-5821.072] (-5833.412) -- 10:50:51      1019000 -- (-5844.275) (-5831.100) [-5820.806] (-5835.493) * (-5853.577) (-5820.597) [-5818.554] (-5828.809) -- 10:50:43      1020000 -- (-5846.424) (-5848.175) [-5827.185] (-5823.628) * (-5834.571) (-5836.950) [-5816.569] (-5830.507) -- 10:50:45      Average standard deviation of split frequencies: 0.018538      1021000 -- (-5837.402) (-5865.463) (-5837.771) [-5813.554] * (-5853.570) (-5852.575) (-5833.858) [-5823.096] -- 10:50:37      1022000 -- [-5832.396] (-5863.772) (-5855.697) (-5824.364) * (-5834.123) (-5835.860) (-5831.957) [-5821.725] -- 10:50:39      1023000 -- (-5832.300) (-5845.656) [-5835.553] (-5848.327) * (-5857.483) (-5846.528) [-5823.927] (-5826.891) -- 10:50:31      1024000 -- (-5858.460) (-5845.150) [-5818.616] (-5841.500) * (-5860.152) (-5858.607) [-5816.111] (-5845.239) -- 10:50:33      1025000 -- (-5846.830) (-5845.017) [-5845.258] (-5823.282) * [-5843.373] (-5869.854) (-5829.558) (-5835.758) -- 10:50:25      Average standard deviation of split frequencies: 0.018350      1026000 -- (-5858.387) (-5819.846) (-5835.983) [-5823.379] * (-5846.017) [-5828.070] (-5832.790) (-5847.374) -- 10:50:27      1027000 -- (-5859.584) (-5848.272) (-5839.932) [-5829.459] * (-5840.853) [-5819.464] (-5838.511) (-5861.547) -- 10:50:19      1028000 -- (-5855.110) (-5844.713) [-5822.293] (-5836.583) * (-5834.703) [-5827.576] (-5844.119) (-5838.683) -- 10:50:12      1029000 -- (-5835.331) (-5844.063) [-5834.876] (-5837.615) * (-5861.888) [-5819.453] (-5833.580) (-5856.931) -- 10:50:13      1030000 -- (-5825.469) (-5866.472) [-5840.967] (-5820.499) * (-5860.239) (-5823.923) [-5825.148] (-5843.591) -- 10:50:06      Average standard deviation of split frequencies: 0.018238      1031000 -- (-5835.389) (-5849.698) [-5833.004] (-5829.171) * (-5851.958) [-5824.086] (-5841.260) (-5828.814) -- 10:49:59      1032000 -- (-5837.284) (-5856.325) (-5836.366) [-5829.975] * (-5880.307) [-5823.567] (-5845.987) (-5843.249) -- 10:50:00      1033000 -- (-5857.763) (-5838.909) (-5834.038) [-5819.326] * (-5867.004) [-5823.567] (-5845.953) (-5831.348) -- 10:49:52      1034000 -- (-5841.753) [-5825.109] (-5836.417) (-5846.881) * (-5843.017) (-5824.361) (-5864.664) [-5836.241] -- 10:49:54      1035000 -- [-5827.769] (-5835.395) (-5822.422) (-5864.243) * [-5834.500] (-5826.423) (-5865.052) (-5849.015) -- 10:49:46      Average standard deviation of split frequencies: 0.018093      1036000 -- (-5824.287) (-5848.496) (-5827.929) [-5828.706] * (-5835.479) (-5837.563) (-5858.386) [-5838.158] -- 10:49:48      1037000 -- [-5833.304] (-5846.663) (-5836.305) (-5873.303) * (-5841.146) [-5830.755] (-5857.354) (-5833.435) -- 10:49:40      1038000 -- (-5846.588) (-5833.838) [-5820.962] (-5870.267) * (-5841.215) [-5813.779] (-5864.470) (-5837.073) -- 10:49:42      1039000 -- [-5832.637] (-5845.591) (-5830.380) (-5866.405) * (-5850.030) [-5814.461] (-5868.373) (-5821.378) -- 10:49:43      1040000 -- (-5830.296) (-5831.230) [-5819.459] (-5874.988) * (-5865.917) [-5831.862] (-5856.763) (-5824.733) -- 10:49:53      Average standard deviation of split frequencies: 0.018198      1041000 -- (-5828.414) (-5837.512) [-5820.678] (-5870.790) * [-5826.930] (-5830.478) (-5855.690) (-5831.767) -- 10:50:03      1042000 -- [-5822.341] (-5845.811) (-5818.329) (-5867.983) * (-5856.007) [-5825.863] (-5844.435) (-5843.365) -- 10:50:04      1043000 -- (-5833.644) (-5832.057) [-5810.894] (-5864.757) * (-5827.704) (-5838.647) [-5828.285] (-5861.136) -- 10:49:56      1044000 -- [-5832.416] (-5835.787) (-5825.281) (-5874.956) * [-5811.667] (-5839.334) (-5851.492) (-5847.773) -- 10:49:58      1045000 -- [-5831.831] (-5847.588) (-5826.013) (-5868.862) * [-5822.651] (-5829.594) (-5850.757) (-5831.774) -- 10:49:50      Average standard deviation of split frequencies: 0.018302      1046000 -- (-5824.141) (-5855.180) [-5817.405] (-5852.806) * [-5824.635] (-5830.887) (-5874.208) (-5842.875) -- 10:49:43      1047000 -- (-5845.453) (-5832.323) [-5823.390] (-5848.550) * [-5835.932] (-5859.933) (-5848.639) (-5832.054) -- 10:49:44      1048000 -- (-5858.936) [-5836.003] (-5832.155) (-5852.786) * (-5840.031) [-5820.242] (-5858.637) (-5835.973) -- 10:49:37      1049000 -- (-5856.230) [-5829.682] (-5829.955) (-5863.316) * [-5832.077] (-5843.059) (-5846.440) (-5833.750) -- 10:49:29      1050000 -- [-5838.417] (-5816.795) (-5843.208) (-5852.724) * (-5848.688) [-5819.234] (-5846.392) (-5831.707) -- 10:49:30      Average standard deviation of split frequencies: 0.018022      1051000 -- [-5821.340] (-5818.321) (-5837.995) (-5831.638) * [-5827.621] (-5828.058) (-5840.104) (-5822.634) -- 10:49:23      1052000 -- (-5815.716) [-5833.478] (-5834.051) (-5827.120) * (-5828.087) [-5827.872] (-5846.764) (-5829.251) -- 10:49:16      1053000 -- (-5838.404) [-5823.649] (-5824.852) (-5835.357) * [-5820.561] (-5831.180) (-5842.226) (-5846.482) -- 10:49:08      1054000 -- (-5851.502) [-5824.591] (-5825.174) (-5835.783) * (-5818.149) [-5823.145] (-5844.717) (-5854.851) -- 10:49:09      1055000 -- (-5855.906) [-5823.020] (-5829.519) (-5821.904) * [-5810.068] (-5833.244) (-5831.643) (-5843.102) -- 10:49:02      Average standard deviation of split frequencies: 0.017994      1056000 -- (-5852.213) [-5827.055] (-5829.067) (-5820.953) * (-5832.967) (-5823.585) [-5817.300] (-5852.538) -- 10:48:55      1057000 -- [-5834.578] (-5830.343) (-5842.209) (-5836.662) * (-5846.342) (-5835.276) [-5816.986] (-5862.019) -- 10:48:47      1058000 -- [-5828.899] (-5855.434) (-5833.830) (-5845.628) * (-5844.679) [-5826.233] (-5820.777) (-5842.416) -- 10:48:48      1059000 -- (-5841.469) [-5832.846] (-5831.815) (-5837.987) * (-5841.227) (-5818.741) [-5823.341] (-5835.011) -- 10:48:41      1060000 -- (-5837.619) (-5844.099) [-5815.410] (-5851.491) * (-5860.214) [-5829.365] (-5842.457) (-5830.561) -- 10:48:34      Average standard deviation of split frequencies: 0.017880      1061000 -- (-5854.509) (-5842.159) (-5837.886) [-5832.091] * (-5833.023) (-5830.718) [-5834.667] (-5837.182) -- 10:48:35      1062000 -- (-5837.687) (-5860.734) [-5820.007] (-5854.899) * (-5836.138) [-5815.491] (-5832.568) (-5835.730) -- 10:48:28      1063000 -- (-5837.315) (-5876.046) [-5825.942] (-5827.183) * (-5846.945) [-5824.371] (-5868.295) (-5849.977) -- 10:48:20      1064000 -- (-5839.714) (-5859.967) (-5843.429) [-5830.726] * (-5828.122) [-5823.024] (-5829.984) (-5838.325) -- 10:48:21      1065000 -- [-5825.932] (-5874.899) (-5835.691) (-5826.728) * (-5831.190) [-5816.271] (-5852.504) (-5875.635) -- 10:48:14      Average standard deviation of split frequencies: 0.017967      1066000 -- (-5841.955) (-5863.028) (-5854.123) [-5824.108] * (-5853.328) [-5809.622] (-5842.104) (-5845.775) -- 10:48:07      1067000 -- (-5864.658) (-5863.013) (-5848.085) [-5831.158] * (-5863.687) [-5820.026] (-5859.924) (-5844.176) -- 10:48:08      1068000 -- (-5848.649) [-5849.418] (-5859.214) (-5830.796) * (-5846.825) [-5826.748] (-5854.598) (-5828.170) -- 10:48:00      1069000 -- (-5863.294) [-5840.259] (-5879.789) (-5821.765) * (-5863.436) (-5841.644) (-5845.787) [-5827.745] -- 10:47:53      1070000 -- (-5824.806) (-5838.944) (-5838.723) [-5816.768] * (-5840.862) (-5818.903) (-5840.700) [-5829.137] -- 10:47:46      Average standard deviation of split frequencies: 0.017872      1071000 -- (-5843.105) (-5861.512) (-5846.941) [-5823.304] * (-5855.398) [-5833.109] (-5836.415) (-5830.920) -- 10:47:39      1072000 -- (-5872.274) [-5819.992] (-5846.464) (-5831.652) * (-5822.139) (-5849.377) (-5866.585) [-5825.224] -- 10:47:40      1073000 -- [-5840.050] (-5832.566) (-5855.065) (-5844.790) * (-5832.139) (-5831.008) (-5861.687) [-5828.404] -- 10:47:32      1074000 -- (-5846.843) [-5826.025] (-5831.070) (-5835.086) * [-5833.053] (-5841.582) (-5831.900) (-5850.240) -- 10:47:25      1075000 -- (-5835.497) (-5825.437) [-5831.227] (-5830.211) * [-5837.764] (-5856.018) (-5850.101) (-5831.388) -- 10:47:18      Average standard deviation of split frequencies: 0.017739      1076000 -- (-5837.395) (-5829.604) (-5834.655) [-5832.098] * (-5838.549) (-5863.511) (-5834.036) [-5827.406] -- 10:47:19      1077000 -- [-5824.426] (-5836.331) (-5844.306) (-5839.188) * (-5847.587) (-5856.606) (-5822.444) [-5830.831] -- 10:47:12      1078000 -- [-5822.694] (-5854.375) (-5832.800) (-5839.806) * (-5854.774) (-5859.409) (-5830.501) [-5831.949] -- 10:47:04      1079000 -- (-5850.523) (-5834.151) [-5819.376] (-5836.404) * (-5839.494) (-5847.674) (-5839.324) [-5820.370] -- 10:46:57      1080000 -- (-5841.038) (-5826.849) (-5843.185) [-5829.028] * (-5838.018) [-5816.910] (-5841.399) (-5826.157) -- 10:46:50      Average standard deviation of split frequencies: 0.017734      1081000 -- (-5853.756) [-5825.582] (-5841.258) (-5830.513) * (-5866.058) [-5819.628] (-5832.815) (-5824.979) -- 10:46:51      1082000 -- (-5853.716) (-5825.297) (-5841.200) [-5825.517] * (-5862.857) (-5844.858) (-5840.066) [-5823.676] -- 10:46:44      1083000 -- (-5863.868) (-5833.830) (-5859.958) [-5827.061] * (-5841.385) (-5831.690) (-5837.185) [-5814.416] -- 10:46:36      1084000 -- (-5852.618) [-5830.480] (-5867.364) (-5856.776) * (-5862.023) (-5841.968) (-5827.635) [-5815.510] -- 10:46:29      1085000 -- (-5852.578) (-5842.206) (-5843.469) [-5828.478] * [-5854.554] (-5844.743) (-5830.688) (-5834.246) -- 10:46:30      Average standard deviation of split frequencies: 0.017676      1086000 -- (-5834.553) [-5834.623] (-5877.621) (-5847.240) * (-5852.541) (-5843.361) (-5839.383) [-5828.333] -- 10:46:23      1087000 -- (-5850.376) (-5844.438) (-5857.126) [-5830.170] * (-5842.058) (-5848.555) [-5837.931] (-5860.156) -- 10:46:16      1088000 -- (-5839.486) (-5842.803) (-5855.218) [-5839.252] * (-5835.407) [-5830.597] (-5825.416) (-5859.461) -- 10:46:17      1089000 -- (-5830.608) [-5822.105] (-5859.665) (-5851.058) * (-5848.055) (-5832.602) [-5820.104] (-5845.728) -- 10:46:09      1090000 -- [-5821.222] (-5840.022) (-5870.745) (-5833.273) * (-5831.797) (-5822.775) [-5821.305] (-5855.756) -- 10:46:02      Average standard deviation of split frequencies: 0.017519      1091000 -- (-5835.730) [-5832.420] (-5878.297) (-5822.532) * [-5829.015] (-5828.904) (-5832.171) (-5846.866) -- 10:45:55      1092000 -- (-5825.029) (-5872.576) (-5849.341) [-5818.376] * [-5825.477] (-5825.582) (-5831.237) (-5834.602) -- 10:45:56      1093000 -- (-5833.371) (-5850.754) (-5859.140) [-5829.372] * (-5827.158) (-5840.688) (-5850.183) [-5828.606] -- 10:45:49      1094000 -- (-5838.913) (-5838.375) (-5872.172) [-5827.243] * (-5830.582) (-5837.896) [-5838.509] (-5864.180) -- 10:45:41      1095000 -- (-5843.461) [-5838.751] (-5857.999) (-5831.513) * (-5837.324) [-5818.460] (-5839.964) (-5835.010) -- 10:45:42      Average standard deviation of split frequencies: 0.017642      1096000 -- (-5830.731) (-5851.004) (-5834.408) [-5818.806] * (-5832.071) [-5813.532] (-5827.201) (-5853.737) -- 10:45:35      1097000 -- (-5833.750) (-5861.170) (-5854.118) [-5819.110] * (-5846.172) (-5828.307) [-5836.318] (-5850.166) -- 10:45:28      1098000 -- (-5853.834) (-5851.766) (-5881.098) [-5816.985] * (-5848.377) [-5845.170] (-5826.387) (-5846.122) -- 10:45:29      1099000 -- (-5837.995) (-5851.555) (-5860.653) [-5817.953] * (-5852.262) [-5831.605] (-5832.579) (-5848.947) -- 10:45:22      1100000 -- (-5832.331) [-5836.826] (-5840.873) (-5834.798) * (-5827.884) (-5828.515) [-5836.636] (-5858.798) -- 10:45:15      Average standard deviation of split frequencies: 0.017222      1101000 -- (-5851.763) (-5836.128) (-5861.361) [-5830.904] * (-5829.626) [-5848.847] (-5840.457) (-5837.508) -- 10:45:15      1102000 -- (-5843.749) [-5818.695] (-5865.132) (-5832.991) * [-5826.518] (-5846.000) (-5853.054) (-5840.206) -- 10:45:08      1103000 -- (-5825.804) (-5831.983) (-5858.587) [-5835.160] * (-5825.218) (-5855.047) (-5847.176) [-5832.184] -- 10:45:01      1104000 -- (-5831.518) (-5843.838) [-5845.510] (-5860.896) * (-5833.563) (-5846.332) (-5840.109) [-5825.665] -- 10:44:54      1105000 -- (-5833.617) [-5843.707] (-5844.638) (-5842.115) * (-5824.441) [-5832.237] (-5845.669) (-5827.099) -- 10:44:47      Average standard deviation of split frequencies: 0.017139      1106000 -- (-5840.668) (-5858.516) [-5820.004] (-5837.639) * [-5821.792] (-5831.736) (-5835.728) (-5834.717) -- 10:44:40      1107000 -- (-5843.564) (-5861.580) [-5829.473] (-5828.728) * (-5843.956) (-5853.388) [-5830.337] (-5830.432) -- 10:44:40      1108000 -- (-5838.972) (-5842.291) (-5841.115) [-5815.894] * (-5848.727) (-5831.355) [-5828.363] (-5845.024) -- 10:44:33      1109000 -- (-5829.666) (-5865.124) (-5839.730) [-5812.495] * (-5862.797) [-5821.442] (-5849.009) (-5843.093) -- 10:44:26      1110000 -- [-5820.863] (-5869.666) (-5848.777) (-5819.472) * (-5872.039) (-5830.432) [-5826.911] (-5856.754) -- 10:44:19      Average standard deviation of split frequencies: 0.016803      1111000 -- [-5821.070] (-5864.862) (-5854.870) (-5823.308) * (-5874.269) (-5835.082) [-5839.737] (-5851.624) -- 10:44:12      1112000 -- (-5817.750) (-5844.015) (-5839.472) [-5823.027] * (-5853.691) (-5835.963) (-5835.756) [-5841.384] -- 10:44:05      1113000 -- [-5825.967] (-5859.276) (-5855.568) (-5829.471) * (-5842.226) [-5838.140] (-5841.335) (-5849.495) -- 10:43:58      1114000 -- [-5826.835] (-5853.098) (-5852.818) (-5825.126) * (-5873.598) (-5832.277) (-5830.418) [-5832.673] -- 10:43:58      1115000 -- (-5827.793) (-5854.753) (-5871.730) [-5832.222] * (-5873.605) [-5837.045] (-5827.016) (-5816.486) -- 10:43:51      Average standard deviation of split frequencies: 0.016758      1116000 -- [-5835.591] (-5840.393) (-5862.627) (-5834.230) * (-5832.294) (-5858.080) (-5828.751) [-5815.793] -- 10:43:44      1117000 -- (-5841.560) [-5820.937] (-5850.023) (-5843.832) * [-5821.850] (-5863.216) (-5840.991) (-5819.401) -- 10:43:37      1118000 -- (-5854.233) (-5817.570) (-5830.848) [-5823.888] * (-5831.607) (-5846.550) [-5823.833] (-5841.085) -- 10:43:30      1119000 -- (-5870.517) (-5831.850) [-5826.754] (-5820.038) * [-5827.639] (-5855.476) (-5840.447) (-5833.923) -- 10:43:23      1120000 -- (-5859.729) (-5831.760) [-5811.894] (-5826.929) * [-5835.421] (-5857.885) (-5846.444) (-5847.474) -- 10:43:24      Average standard deviation of split frequencies: 0.016609      1121000 -- (-5846.385) [-5847.502] (-5832.803) (-5843.559) * (-5850.669) [-5831.605] (-5858.711) (-5851.775) -- 10:43:17      1122000 -- (-5848.612) (-5840.899) [-5835.523] (-5855.148) * (-5838.744) (-5836.571) (-5860.684) [-5828.780] -- 10:43:10      1123000 -- (-5829.950) [-5823.512] (-5828.997) (-5857.100) * (-5838.862) (-5845.605) (-5863.777) [-5838.633] -- 10:43:02      1124000 -- [-5827.436] (-5837.727) (-5829.674) (-5874.714) * (-5835.550) (-5853.885) (-5847.953) [-5833.226] -- 10:43:03      1125000 -- [-5828.662] (-5845.989) (-5844.331) (-5851.654) * (-5821.597) (-5852.029) (-5861.265) [-5837.637] -- 10:42:56      Average standard deviation of split frequencies: 0.016668      1126000 -- (-5822.372) [-5828.932] (-5856.402) (-5852.910) * [-5832.814] (-5864.618) (-5856.851) (-5844.015) -- 10:42:49      1127000 -- (-5837.819) [-5817.166] (-5846.842) (-5842.243) * [-5828.095] (-5846.698) (-5835.963) (-5843.947) -- 10:42:50      1128000 -- [-5819.417] (-5820.589) (-5848.142) (-5848.278) * (-5825.154) [-5830.228] (-5833.929) (-5851.335) -- 10:42:43      1129000 -- (-5839.845) [-5811.012] (-5838.030) (-5856.420) * (-5852.732) (-5837.445) (-5861.438) [-5831.555] -- 10:42:36      1130000 -- (-5830.726) [-5820.010] (-5867.902) (-5860.605) * (-5833.407) [-5824.885] (-5829.793) (-5837.243) -- 10:42:29      Average standard deviation of split frequencies: 0.016529      1131000 -- [-5830.327] (-5835.281) (-5853.399) (-5857.318) * [-5814.280] (-5821.210) (-5856.640) (-5851.719) -- 10:42:29      1132000 -- (-5836.598) [-5834.209] (-5851.316) (-5858.003) * (-5848.621) (-5846.475) (-5858.034) [-5825.821] -- 10:42:22      1133000 -- [-5826.137] (-5837.955) (-5852.725) (-5832.737) * (-5839.451) (-5827.514) (-5861.468) [-5829.142] -- 10:42:15      1134000 -- [-5817.300] (-5846.200) (-5866.113) (-5826.035) * (-5824.706) [-5820.844] (-5851.019) (-5853.897) -- 10:42:16      1135000 -- (-5818.008) (-5855.368) (-5848.473) [-5818.222] * (-5826.221) (-5850.496) [-5838.015] (-5841.069) -- 10:42:09      Average standard deviation of split frequencies: 0.016214      1136000 -- [-5821.199] (-5853.276) (-5848.081) (-5815.610) * (-5827.410) (-5865.241) (-5840.824) [-5825.502] -- 10:42:02      1137000 -- [-5814.380] (-5858.852) (-5854.692) (-5831.740) * [-5826.871] (-5833.022) (-5849.003) (-5826.990) -- 10:42:03      1138000 -- (-5810.280) (-5859.940) (-5878.096) [-5817.305] * (-5837.764) [-5825.558] (-5844.004) (-5826.605) -- 10:41:56      1139000 -- [-5812.615] (-5844.796) (-5850.526) (-5814.051) * (-5843.781) (-5850.207) (-5841.841) [-5831.473] -- 10:41:49      1140000 -- [-5820.300] (-5862.282) (-5840.619) (-5822.850) * (-5841.796) (-5846.320) (-5837.545) [-5814.728] -- 10:41:49      Average standard deviation of split frequencies: 0.015898      1141000 -- (-5827.845) (-5836.195) (-5838.610) [-5813.276] * (-5845.984) (-5834.711) [-5820.117] (-5831.279) -- 10:41:42      1142000 -- (-5832.769) (-5873.158) (-5831.552) [-5812.720] * (-5840.086) (-5823.908) [-5832.145] (-5828.084) -- 10:41:35      1143000 -- (-5847.463) (-5868.176) (-5840.888) [-5824.506] * (-5847.095) [-5827.346] (-5829.037) (-5832.749) -- 10:41:36      1144000 -- (-5838.524) (-5845.575) (-5858.138) [-5826.479] * (-5845.707) [-5817.704] (-5839.518) (-5841.658) -- 10:41:29      1145000 -- (-5858.744) (-5844.719) (-5824.963) [-5829.047] * (-5847.422) (-5849.155) [-5839.285] (-5825.913) -- 10:41:22      Average standard deviation of split frequencies: 0.015869      1146000 -- (-5845.246) (-5841.185) [-5818.989] (-5844.209) * (-5865.389) (-5836.345) (-5835.083) [-5820.273] -- 10:41:15      1147000 -- (-5855.736) (-5847.810) [-5823.002] (-5833.908) * (-5838.204) (-5836.201) [-5844.878] (-5818.525) -- 10:41:16      1148000 -- (-5840.076) (-5848.408) [-5832.367] (-5854.255) * (-5846.023) (-5853.135) (-5830.035) [-5831.767] -- 10:41:09      1149000 -- [-5835.558] (-5850.904) (-5833.697) (-5864.243) * (-5875.737) (-5854.942) (-5857.222) [-5828.252] -- 10:41:02      1150000 -- [-5842.942] (-5850.262) (-5827.795) (-5864.193) * (-5832.671) (-5848.298) (-5854.866) [-5826.882] -- 10:41:02      Average standard deviation of split frequencies: 0.015654      1151000 -- (-5831.694) (-5859.467) [-5819.708] (-5847.483) * (-5826.248) (-5858.212) (-5851.774) [-5837.267] -- 10:40:55      1152000 -- [-5824.788] (-5856.904) (-5830.348) (-5843.130) * [-5828.800] (-5854.831) (-5864.519) (-5820.938) -- 10:40:48      1153000 -- (-5839.911) (-5859.117) [-5824.619] (-5841.299) * [-5825.656] (-5846.011) (-5850.632) (-5823.346) -- 10:40:41      1154000 -- (-5815.213) (-5852.175) [-5820.111] (-5841.214) * [-5815.084] (-5817.737) (-5857.091) (-5853.817) -- 10:40:42      1155000 -- [-5837.143] (-5863.035) (-5840.645) (-5838.255) * (-5829.579) [-5818.836] (-5844.208) (-5861.897) -- 10:40:35      Average standard deviation of split frequencies: 0.015540      1156000 -- [-5828.511] (-5864.770) (-5819.027) (-5872.119) * (-5838.939) [-5806.050] (-5859.429) (-5824.409) -- 10:40:28      1157000 -- [-5845.236] (-5845.834) (-5838.818) (-5878.952) * [-5814.475] (-5814.726) (-5854.671) (-5824.483) -- 10:40:29      1158000 -- [-5837.488] (-5850.488) (-5853.699) (-5850.238) * [-5807.856] (-5847.056) (-5836.036) (-5832.361) -- 10:40:22      1159000 -- (-5836.078) [-5852.111] (-5846.876) (-5841.533) * [-5819.407] (-5848.125) (-5857.292) (-5843.767) -- 10:40:15      1160000 -- [-5823.832] (-5847.554) (-5848.839) (-5834.451) * (-5817.953) [-5826.309] (-5838.812) (-5862.010) -- 10:40:08      Average standard deviation of split frequencies: 0.015226      1161000 -- [-5821.037] (-5832.961) (-5871.205) (-5841.893) * [-5820.189] (-5819.708) (-5838.081) (-5853.725) -- 10:40:08      1162000 -- [-5828.655] (-5832.581) (-5889.666) (-5839.650) * (-5840.394) [-5829.380] (-5854.094) (-5842.749) -- 10:40:01      1163000 -- [-5831.803] (-5822.876) (-5854.190) (-5838.483) * (-5860.230) [-5826.686] (-5831.216) (-5845.911) -- 10:39:54      1164000 -- (-5824.322) [-5822.094] (-5849.402) (-5825.479) * (-5853.840) (-5841.298) [-5819.860] (-5831.956) -- 10:39:48      1165000 -- (-5831.992) [-5829.479] (-5853.864) (-5837.137) * (-5840.194) [-5817.617] (-5814.128) (-5851.667) -- 10:39:48      Average standard deviation of split frequencies: 0.015105      1166000 -- [-5825.377] (-5838.316) (-5839.325) (-5842.099) * (-5846.598) [-5835.902] (-5832.267) (-5858.431) -- 10:39:41      1167000 -- (-5853.539) (-5830.064) [-5842.954] (-5848.411) * [-5841.151] (-5840.453) (-5850.165) (-5867.433) -- 10:39:34      1168000 -- (-5838.121) [-5831.405] (-5834.984) (-5837.609) * (-5825.776) [-5833.842] (-5857.838) (-5878.607) -- 10:39:35      1169000 -- (-5858.240) (-5819.241) [-5819.785] (-5848.400) * (-5836.335) [-5832.955] (-5851.144) (-5860.102) -- 10:39:28      1170000 -- (-5855.248) [-5823.024] (-5828.054) (-5843.406) * [-5821.705] (-5842.625) (-5857.333) (-5853.922) -- 10:39:21      Average standard deviation of split frequencies: 0.015116      1171000 -- (-5850.663) (-5846.163) [-5831.663] (-5837.714) * [-5813.176] (-5841.068) (-5846.924) (-5844.320) -- 10:39:14      1172000 -- (-5875.482) (-5840.413) (-5840.563) [-5839.188] * (-5831.202) [-5819.216] (-5852.876) (-5844.215) -- 10:39:07      1173000 -- (-5859.400) [-5828.822] (-5834.747) (-5850.021) * (-5826.671) [-5825.593] (-5847.856) (-5841.203) -- 10:39:08      1174000 -- (-5853.964) (-5836.930) [-5832.863] (-5854.949) * [-5820.155] (-5839.401) (-5864.989) (-5874.337) -- 10:39:01      1175000 -- (-5846.465) (-5847.266) [-5821.073] (-5860.440) * [-5810.882] (-5846.048) (-5849.962) (-5833.586) -- 10:38:54      Average standard deviation of split frequencies: 0.014919      1176000 -- (-5829.252) (-5841.665) [-5827.835] (-5874.641) * (-5830.075) (-5829.113) (-5842.834) [-5837.938] -- 10:38:47      1177000 -- (-5847.307) (-5833.496) [-5830.829] (-5878.541) * [-5824.492] (-5833.561) (-5830.748) (-5833.731) -- 10:38:47      1178000 -- (-5837.929) [-5817.720] (-5848.748) (-5883.702) * (-5848.791) [-5834.620] (-5855.667) (-5849.883) -- 10:38:41      1179000 -- (-5840.952) [-5834.874] (-5849.910) (-5876.918) * (-5839.354) [-5815.718] (-5858.389) (-5840.116) -- 10:38:34      1180000 -- (-5823.924) (-5843.892) [-5828.623] (-5866.662) * (-5844.381) [-5823.486] (-5844.445) (-5859.176) -- 10:38:27      Average standard deviation of split frequencies: 0.014627      1181000 -- [-5831.857] (-5845.639) (-5830.593) (-5859.657) * (-5832.826) [-5830.586] (-5857.901) (-5832.419) -- 10:38:20      1182000 -- (-5837.919) (-5841.061) (-5838.295) [-5836.270] * (-5836.617) [-5817.561] (-5875.819) (-5840.871) -- 10:38:13      1183000 -- [-5817.910] (-5878.666) (-5832.300) (-5834.597) * (-5842.193) [-5831.368] (-5878.032) (-5838.327) -- 10:38:13      1184000 -- [-5814.608] (-5849.273) (-5818.895) (-5844.256) * (-5843.548) [-5822.473] (-5846.876) (-5839.520) -- 10:38:07      1185000 -- [-5824.860] (-5866.050) (-5819.604) (-5836.478) * (-5841.528) [-5815.372] (-5843.381) (-5839.173) -- 10:38:00      Average standard deviation of split frequencies: 0.014836      1186000 -- (-5841.391) (-5855.800) [-5821.400] (-5851.206) * (-5846.240) [-5827.767] (-5837.044) (-5841.281) -- 10:37:53      1187000 -- (-5824.219) (-5852.456) [-5814.980] (-5844.764) * (-5834.303) [-5833.753] (-5835.089) (-5836.591) -- 10:37:53      1188000 -- [-5826.645] (-5869.800) (-5842.701) (-5835.367) * (-5861.610) (-5841.187) (-5835.440) [-5824.361] -- 10:37:46      1189000 -- [-5819.531] (-5863.429) (-5838.416) (-5835.978) * (-5850.163) [-5833.412] (-5844.283) (-5834.412) -- 10:37:40      1190000 -- (-5825.543) (-5843.573) (-5834.576) [-5831.243] * (-5841.731) (-5826.984) [-5839.787] (-5824.427) -- 10:37:40      Average standard deviation of split frequencies: 0.014776      1191000 -- (-5826.323) (-5844.432) (-5844.935) [-5826.784] * (-5829.967) (-5845.054) (-5850.225) [-5814.097] -- 10:37:33      1192000 -- (-5832.101) (-5835.880) (-5851.106) [-5817.273] * (-5839.522) (-5864.269) (-5838.985) [-5810.546] -- 10:37:26      1193000 -- (-5845.030) (-5843.010) [-5837.106] (-5820.665) * (-5833.335) [-5849.413] (-5850.865) (-5830.505) -- 10:37:27      1194000 -- (-5838.919) [-5838.549] (-5838.109) (-5855.164) * (-5818.249) (-5849.896) (-5845.233) [-5827.633] -- 10:37:20      1195000 -- (-5824.862) [-5841.862] (-5859.454) (-5839.750) * [-5823.223] (-5855.987) (-5830.217) (-5838.637) -- 10:37:13      Average standard deviation of split frequencies: 0.014741      1196000 -- [-5827.656] (-5836.374) (-5833.041) (-5849.850) * [-5820.360] (-5841.609) (-5851.320) (-5846.280) -- 10:37:06      1197000 -- [-5819.508] (-5849.561) (-5836.314) (-5849.469) * (-5841.345) (-5825.713) [-5829.267] (-5830.686) -- 10:37:07      1198000 -- (-5827.815) (-5837.317) [-5823.125] (-5852.981) * (-5835.917) (-5856.123) (-5832.489) [-5833.637] -- 10:37:00      1199000 -- (-5830.123) (-5841.794) [-5807.097] (-5851.809) * [-5833.036] (-5843.475) (-5840.192) (-5848.814) -- 10:36:53      1200000 -- (-5821.305) (-5831.181) [-5811.879] (-5840.191) * [-5812.577] (-5863.342) (-5851.258) (-5833.632) -- 10:36:54      Average standard deviation of split frequencies: 0.014833      1201000 -- (-5841.753) (-5825.347) [-5814.050] (-5847.420) * [-5802.130] (-5852.771) (-5859.838) (-5849.577) -- 10:36:47      1202000 -- (-5859.968) (-5826.276) [-5824.772] (-5845.295) * [-5817.025] (-5857.527) (-5841.991) (-5834.005) -- 10:36:40      1203000 -- (-5856.360) (-5831.195) (-5833.177) [-5829.751] * [-5814.377] (-5865.388) (-5838.206) (-5838.995) -- 10:36:40      1204000 -- (-5842.672) [-5814.083] (-5837.600) (-5830.481) * [-5809.517] (-5856.366) (-5832.563) (-5830.288) -- 10:36:33      1205000 -- (-5824.995) [-5824.174] (-5847.995) (-5826.268) * [-5807.138] (-5846.504) (-5825.116) (-5832.089) -- 10:36:27      Average standard deviation of split frequencies: 0.014923      1206000 -- (-5824.565) (-5848.481) (-5865.216) [-5819.394] * [-5815.060] (-5867.210) (-5828.794) (-5832.012) -- 10:36:20      1207000 -- (-5845.743) (-5822.182) (-5856.970) [-5834.578] * (-5825.348) (-5851.824) (-5841.152) [-5832.230] -- 10:36:20      1208000 -- (-5828.889) [-5832.773] (-5875.682) (-5829.018) * (-5839.189) (-5847.170) [-5817.523] (-5830.326) -- 10:36:13      1209000 -- (-5858.924) (-5846.565) (-5881.570) [-5821.828] * (-5843.366) (-5857.301) [-5830.016] (-5827.462) -- 10:36:07      1210000 -- [-5835.867] (-5831.616) (-5878.365) (-5831.618) * [-5834.695] (-5861.394) (-5818.274) (-5847.223) -- 10:36:00      Average standard deviation of split frequencies: 0.015197      1211000 -- (-5847.650) (-5828.839) (-5863.073) [-5842.202] * (-5854.276) (-5845.669) [-5820.563] (-5857.022) -- 10:36:00      1212000 -- (-5847.824) [-5826.933] (-5839.767) (-5844.749) * (-5841.908) (-5835.976) [-5823.048] (-5865.772) -- 10:35:53      1213000 -- (-5855.277) (-5833.830) [-5823.261] (-5838.618) * [-5834.081] (-5855.278) (-5840.046) (-5844.938) -- 10:35:47      1214000 -- [-5830.319] (-5828.237) (-5852.612) (-5837.943) * (-5850.459) (-5836.615) (-5845.727) [-5840.740] -- 10:35:40      1215000 -- (-5849.144) (-5828.784) [-5824.358] (-5825.891) * (-5846.064) (-5833.857) [-5831.248] (-5859.682) -- 10:35:40      Average standard deviation of split frequencies: 0.015246      1216000 -- (-5879.997) (-5844.596) (-5832.073) [-5825.624] * [-5836.534] (-5850.624) (-5843.031) (-5847.003) -- 10:35:33      1217000 -- (-5845.865) [-5828.214] (-5824.801) (-5842.368) * (-5845.963) (-5857.690) [-5832.834] (-5836.278) -- 10:35:27      1218000 -- (-5849.426) (-5852.139) [-5827.545] (-5830.276) * (-5849.868) (-5874.394) (-5853.104) [-5839.165] -- 10:35:20      1219000 -- (-5849.893) [-5830.953] (-5844.179) (-5839.961) * (-5851.406) (-5868.440) [-5830.806] (-5829.896) -- 10:35:20      1220000 -- (-5855.641) [-5835.220] (-5855.563) (-5840.358) * (-5842.714) (-5837.706) (-5822.998) [-5821.732] -- 10:35:13      Average standard deviation of split frequencies: 0.015292      1221000 -- (-5835.351) (-5820.558) [-5834.191] (-5842.950) * (-5849.267) (-5841.252) (-5843.225) [-5821.195] -- 10:35:07      1222000 -- (-5834.969) (-5821.709) [-5835.927] (-5859.176) * (-5844.171) (-5849.766) [-5820.822] (-5845.488) -- 10:35:00      1223000 -- (-5853.882) [-5833.047] (-5832.618) (-5845.061) * [-5836.530] (-5838.852) (-5839.765) (-5858.281) -- 10:34:53      1224000 -- (-5846.239) [-5841.736] (-5846.751) (-5840.306) * (-5844.432) (-5829.126) [-5823.374] (-5860.110) -- 10:34:53      1225000 -- (-5852.533) [-5829.747] (-5846.725) (-5856.686) * (-5865.755) [-5836.724] (-5820.134) (-5852.196) -- 10:34:47      Average standard deviation of split frequencies: 0.015274      1226000 -- (-5851.839) [-5830.483] (-5834.512) (-5852.596) * (-5863.247) [-5824.691] (-5830.292) (-5849.073) -- 10:34:47      1227000 -- (-5847.695) [-5812.975] (-5833.804) (-5841.330) * (-5863.806) [-5821.156] (-5833.741) (-5861.479) -- 10:34:40      1228000 -- (-5847.507) [-5816.060] (-5851.477) (-5825.580) * (-5834.647) [-5833.591] (-5843.505) (-5859.156) -- 10:34:33      1229000 -- (-5840.961) [-5823.933] (-5852.538) (-5825.849) * [-5825.518] (-5851.182) (-5834.419) (-5867.707) -- 10:34:34      1230000 -- [-5812.754] (-5830.590) (-5851.567) (-5834.009) * [-5815.173] (-5844.145) (-5834.021) (-5852.734) -- 10:34:27      Average standard deviation of split frequencies: 0.015716      1231000 -- [-5813.991] (-5828.353) (-5858.571) (-5817.744) * (-5823.287) [-5826.676] (-5851.771) (-5834.504) -- 10:34:20      1232000 -- [-5822.797] (-5837.593) (-5845.672) (-5835.212) * (-5841.551) [-5816.638] (-5843.058) (-5830.876) -- 10:34:13      1233000 -- [-5821.784] (-5834.072) (-5848.318) (-5836.160) * [-5825.167] (-5831.081) (-5868.695) (-5849.183) -- 10:34:14      1234000 -- (-5824.198) (-5857.694) (-5841.941) [-5816.444] * [-5836.699] (-5837.890) (-5858.670) (-5850.601) -- 10:34:07      1235000 -- [-5823.332] (-5857.030) (-5849.913) (-5829.542) * [-5832.055] (-5848.179) (-5847.677) (-5845.807) -- 10:34:00      Average standard deviation of split frequencies: 0.016386      1236000 -- [-5815.810] (-5857.807) (-5862.303) (-5848.924) * (-5857.794) (-5840.997) [-5809.923] (-5854.114) -- 10:34:01      1237000 -- [-5816.279] (-5871.883) (-5853.636) (-5844.724) * (-5861.422) (-5838.332) [-5821.291] (-5853.684) -- 10:33:54      1238000 -- [-5834.613] (-5858.305) (-5844.572) (-5866.365) * (-5865.805) (-5848.185) (-5831.541) [-5829.741] -- 10:33:47      1239000 -- [-5835.572] (-5866.986) (-5844.315) (-5848.054) * (-5828.264) [-5825.459] (-5831.273) (-5859.669) -- 10:33:40      1240000 -- [-5826.065] (-5886.713) (-5860.225) (-5833.690) * (-5853.064) [-5823.226] (-5834.361) (-5844.685) -- 10:33:34      Average standard deviation of split frequencies: 0.016613      1241000 -- (-5836.846) (-5864.551) [-5829.955] (-5840.966) * (-5875.267) (-5828.754) [-5811.328] (-5834.510) -- 10:33:34      1242000 -- [-5825.326] (-5843.571) (-5835.208) (-5852.647) * (-5869.521) (-5830.268) (-5837.322) [-5833.069] -- 10:33:27      1243000 -- [-5835.213] (-5831.222) (-5818.136) (-5867.706) * (-5857.750) (-5819.062) (-5823.246) [-5829.577] -- 10:33:21      1244000 -- (-5830.175) (-5827.119) [-5825.822] (-5862.506) * (-5841.082) [-5826.275] (-5849.442) (-5829.997) -- 10:33:21      1245000 -- (-5828.836) [-5824.810] (-5856.099) (-5858.307) * (-5844.707) [-5832.654] (-5852.029) (-5851.305) -- 10:33:14      Average standard deviation of split frequencies: 0.016672      1246000 -- [-5826.871] (-5825.497) (-5842.306) (-5852.900) * (-5834.958) [-5837.703] (-5845.425) (-5833.724) -- 10:33:07      1247000 -- (-5841.499) (-5839.689) [-5823.295] (-5829.281) * (-5862.644) (-5838.593) (-5838.519) [-5820.256] -- 10:33:08      1248000 -- (-5836.019) [-5839.774] (-5834.620) (-5841.674) * (-5868.025) (-5830.237) (-5862.258) [-5834.369] -- 10:33:01      1249000 -- (-5848.912) (-5839.965) [-5825.011] (-5838.697) * (-5876.666) (-5827.375) (-5870.900) [-5832.400] -- 10:32:54      1250000 -- [-5834.464] (-5867.024) (-5821.943) (-5850.637) * (-5848.910) (-5824.154) (-5873.037) [-5827.171] -- 10:32:55      Average standard deviation of split frequencies: 0.016567      1251000 -- (-5846.606) (-5852.671) (-5822.245) [-5820.856] * (-5837.046) [-5815.140] (-5860.565) (-5832.397) -- 10:32:48      1252000 -- (-5847.992) (-5829.460) [-5838.541] (-5832.067) * (-5834.576) [-5823.085] (-5844.301) (-5837.851) -- 10:32:41      1253000 -- [-5831.006] (-5833.437) (-5850.183) (-5826.518) * (-5841.369) (-5819.564) (-5855.214) [-5831.159] -- 10:32:41      1254000 -- (-5841.053) [-5840.987] (-5847.071) (-5828.654) * (-5848.387) [-5808.345] (-5866.988) (-5828.275) -- 10:32:35      1255000 -- (-5830.154) (-5847.567) (-5846.594) [-5821.782] * (-5848.949) [-5806.699] (-5864.075) (-5820.790) -- 10:32:28      Average standard deviation of split frequencies: 0.016576      1256000 -- (-5835.869) (-5851.225) (-5847.864) [-5829.977] * (-5828.061) [-5810.641] (-5858.292) (-5828.740) -- 10:32:28      1257000 -- [-5829.768] (-5845.080) (-5840.966) (-5835.189) * (-5856.510) [-5832.313] (-5835.661) (-5832.185) -- 10:32:21      1258000 -- (-5848.949) [-5816.945] (-5839.949) (-5844.323) * (-5830.206) (-5829.143) (-5832.452) [-5834.410] -- 10:32:15      1259000 -- (-5836.551) (-5827.162) (-5841.388) [-5832.013] * (-5842.528) [-5846.631] (-5830.962) (-5849.415) -- 10:32:15      1260000 -- (-5839.107) (-5836.858) [-5838.771] (-5834.412) * (-5833.915) [-5825.268] (-5829.224) (-5861.429) -- 10:32:08      Average standard deviation of split frequencies: 0.016712      1261000 -- [-5830.812] (-5835.758) (-5831.986) (-5837.399) * (-5842.498) [-5812.806] (-5838.575) (-5869.811) -- 10:32:02      1262000 -- (-5832.463) (-5852.491) [-5817.570] (-5837.882) * (-5853.467) [-5812.627] (-5832.130) (-5854.416) -- 10:31:55      1263000 -- (-5841.065) (-5847.022) [-5818.990] (-5832.965) * (-5867.925) [-5821.522] (-5845.468) (-5833.218) -- 10:31:55      1264000 -- (-5840.623) (-5849.610) (-5841.001) [-5824.586] * (-5872.171) [-5834.721] (-5842.832) (-5834.820) -- 10:31:48      1265000 -- (-5852.918) (-5871.013) (-5834.738) [-5818.294] * (-5879.312) [-5828.615] (-5836.556) (-5849.845) -- 10:31:42      Average standard deviation of split frequencies: 0.016964      1266000 -- (-5841.593) (-5869.484) [-5819.852] (-5825.247) * (-5860.778) (-5843.545) (-5846.868) [-5823.372] -- 10:31:42      1267000 -- (-5844.191) (-5850.573) [-5823.716] (-5824.161) * (-5853.783) (-5827.461) (-5849.889) [-5817.253] -- 10:31:35      1268000 -- (-5852.957) (-5834.617) [-5836.073] (-5829.345) * (-5848.320) (-5831.855) (-5858.947) [-5818.503] -- 10:31:29      1269000 -- (-5881.222) [-5830.609] (-5828.116) (-5839.078) * (-5842.731) (-5838.551) (-5840.779) [-5812.519] -- 10:31:22      1270000 -- (-5882.988) (-5841.841) (-5826.412) [-5838.085] * (-5837.346) (-5865.505) [-5815.957] (-5820.780) -- 10:31:15      Average standard deviation of split frequencies: 0.016872      1271000 -- (-5871.380) (-5835.660) (-5825.570) [-5828.676] * (-5817.696) (-5852.177) [-5818.627] (-5835.309) -- 10:31:16      1272000 -- (-5877.246) (-5848.271) [-5820.701] (-5832.705) * (-5844.583) (-5846.607) [-5820.433] (-5829.651) -- 10:31:09      1273000 -- (-5859.037) (-5854.807) (-5845.054) [-5826.782] * (-5846.791) (-5837.706) [-5825.707] (-5833.339) -- 10:31:02      1274000 -- (-5876.407) [-5828.660] (-5824.903) (-5846.095) * (-5863.817) (-5827.499) (-5835.593) [-5826.772] -- 10:30:56      1275000 -- (-5846.387) (-5840.654) (-5830.176) [-5840.874] * (-5853.846) (-5850.438) (-5834.851) [-5821.561] -- 10:30:56      Average standard deviation of split frequencies: 0.016766      1276000 -- (-5859.008) [-5824.352] (-5839.278) (-5831.720) * (-5856.199) (-5864.597) [-5828.305] (-5827.013) -- 10:30:49      1277000 -- (-5862.036) [-5821.967] (-5842.125) (-5834.122) * (-5836.227) (-5846.940) [-5814.821] (-5816.545) -- 10:30:42      1278000 -- (-5826.808) [-5822.842] (-5839.855) (-5836.011) * (-5842.106) (-5864.542) (-5841.987) [-5826.095] -- 10:30:36      1279000 -- [-5813.724] (-5830.132) (-5842.139) (-5851.165) * (-5845.410) (-5855.294) (-5855.563) [-5820.027] -- 10:30:36      1280000 -- [-5822.781] (-5839.166) (-5841.418) (-5839.537) * (-5859.453) (-5846.601) (-5856.291) [-5817.133] -- 10:30:29      Average standard deviation of split frequencies: 0.016736      1281000 -- [-5820.364] (-5850.080) (-5841.996) (-5838.359) * (-5862.334) (-5831.723) (-5857.929) [-5818.626] -- 10:30:23      1282000 -- (-5859.801) (-5851.968) [-5848.080] (-5823.599) * (-5854.267) [-5810.683] (-5856.149) (-5840.178) -- 10:30:16      1283000 -- (-5855.265) (-5863.046) (-5850.058) [-5813.974] * (-5857.328) (-5827.279) (-5863.934) [-5832.798] -- 10:30:16      1284000 -- (-5856.056) (-5829.022) (-5856.143) [-5818.663] * [-5820.723] (-5830.058) (-5857.439) (-5828.504) -- 10:30:10      1285000 -- (-5848.627) (-5835.870) (-5869.872) [-5830.670] * [-5842.169] (-5837.207) (-5856.336) (-5837.840) -- 10:30:03      Average standard deviation of split frequencies: 0.016760      1286000 -- (-5853.530) [-5836.496] (-5869.469) (-5846.255) * [-5840.056] (-5820.691) (-5866.271) (-5841.673) -- 10:29:56      1287000 -- (-5852.414) [-5822.822] (-5847.617) (-5826.202) * (-5837.858) (-5832.745) (-5851.032) [-5829.753] -- 10:29:56      1288000 -- (-5845.016) (-5830.656) (-5853.678) [-5816.606] * (-5843.641) [-5828.946] (-5837.018) (-5835.556) -- 10:29:50      1289000 -- [-5831.316] (-5822.534) (-5837.928) (-5836.501) * (-5837.118) [-5821.522] (-5841.806) (-5824.890) -- 10:29:43      1290000 -- [-5829.261] (-5830.315) (-5844.902) (-5836.306) * (-5839.404) [-5815.222] (-5849.576) (-5836.009) -- 10:29:43      Average standard deviation of split frequencies: 0.016598      1291000 -- (-5836.789) [-5823.050] (-5861.137) (-5827.559) * (-5867.442) [-5829.480] (-5838.206) (-5834.731) -- 10:29:37      1292000 -- (-5851.142) (-5834.202) [-5817.635] (-5848.678) * (-5843.806) (-5840.826) (-5835.974) [-5835.899] -- 10:29:30      1293000 -- (-5840.751) (-5836.535) [-5827.972] (-5842.404) * (-5853.255) [-5835.959] (-5841.232) (-5840.130) -- 10:29:24      1294000 -- [-5826.078] (-5859.489) (-5826.560) (-5840.032) * (-5831.806) (-5853.309) [-5832.593] (-5826.965) -- 10:29:24      1295000 -- (-5863.692) (-5852.229) [-5830.141] (-5836.465) * [-5830.841] (-5865.000) (-5842.031) (-5833.061) -- 10:29:17      Average standard deviation of split frequencies: 0.016534      1296000 -- (-5852.015) (-5851.283) [-5834.208] (-5837.522) * [-5827.196] (-5863.195) (-5831.299) (-5846.688) -- 10:29:10      1297000 -- [-5833.215] (-5844.667) (-5852.538) (-5845.740) * (-5844.030) (-5886.139) [-5845.462] (-5843.983) -- 10:29:04      1298000 -- [-5827.377] (-5849.732) (-5841.174) (-5847.734) * (-5835.174) (-5868.748) (-5843.310) [-5825.732] -- 10:29:04      1299000 -- (-5823.413) (-5848.638) (-5855.668) [-5835.271] * (-5826.715) (-5880.722) (-5839.999) [-5828.872] -- 10:28:57      1300000 -- [-5817.182] (-5851.017) (-5840.852) (-5830.459) * (-5834.588) (-5856.257) (-5841.348) [-5825.950] -- 10:28:51      Average standard deviation of split frequencies: 0.016598      1301000 -- [-5814.161] (-5836.433) (-5862.070) (-5832.523) * [-5826.161] (-5871.378) (-5849.517) (-5822.174) -- 10:28:44      1302000 -- [-5820.439] (-5846.678) (-5859.238) (-5819.845) * (-5829.909) (-5860.571) (-5854.930) [-5813.878] -- 10:28:44      1303000 -- [-5836.543] (-5860.029) (-5852.262) (-5829.440) * (-5843.973) (-5850.500) (-5837.788) [-5823.423] -- 10:28:38      1304000 -- [-5834.254] (-5843.617) (-5852.745) (-5838.033) * (-5845.504) (-5840.870) [-5842.066] (-5823.350) -- 10:28:31      1305000 -- (-5847.625) [-5843.575] (-5862.139) (-5833.649) * (-5839.558) (-5856.302) (-5854.056) [-5818.791] -- 10:28:24      Average standard deviation of split frequencies: 0.016602      1306000 -- [-5838.274] (-5856.925) (-5849.152) (-5832.036) * (-5825.961) (-5859.953) (-5849.056) [-5816.931] -- 10:28:25      1307000 -- [-5828.453] (-5874.826) (-5837.093) (-5843.891) * (-5845.907) (-5852.227) [-5837.242] (-5827.353) -- 10:28:18      1308000 -- [-5850.199] (-5845.552) (-5833.858) (-5845.109) * (-5843.715) (-5844.699) (-5842.956) [-5830.021] -- 10:28:11      1309000 -- (-5844.253) (-5860.308) [-5834.280] (-5858.730) * (-5849.933) (-5835.022) (-5850.921) [-5816.935] -- 10:28:05      1310000 -- (-5865.963) (-5861.472) [-5833.884] (-5842.480) * (-5865.471) (-5836.727) (-5856.625) [-5814.505] -- 10:27:58      Average standard deviation of split frequencies: 0.016757      1311000 -- (-5853.851) (-5861.592) [-5839.378] (-5830.427) * (-5854.581) [-5843.092] (-5863.670) (-5822.205) -- 10:27:58      1312000 -- (-5842.314) (-5831.598) (-5841.673) [-5809.280] * [-5840.521] (-5844.914) (-5853.110) (-5829.663) -- 10:27:52      1313000 -- (-5869.090) (-5836.669) (-5842.965) [-5806.065] * (-5828.342) [-5835.185] (-5851.273) (-5835.083) -- 10:27:45      1314000 -- (-5863.247) (-5858.742) (-5836.039) [-5822.793] * (-5822.774) (-5836.187) (-5872.579) [-5831.802] -- 10:27:39      1315000 -- (-5854.082) (-5868.029) (-5842.008) [-5823.973] * [-5825.856] (-5830.382) (-5852.939) (-5820.904) -- 10:27:39      Average standard deviation of split frequencies: 0.016588      1316000 -- (-5861.684) (-5845.378) [-5824.350] (-5836.517) * [-5817.783] (-5855.997) (-5856.287) (-5834.569) -- 10:27:32      1317000 -- (-5864.598) (-5875.925) (-5821.318) [-5830.290] * (-5829.135) (-5857.812) (-5852.605) [-5834.155] -- 10:27:26      1318000 -- (-5843.487) (-5873.863) [-5827.615] (-5829.439) * [-5806.676] (-5863.797) (-5855.223) (-5830.900) -- 10:27:19      1319000 -- [-5833.986] (-5861.836) (-5833.991) (-5823.703) * [-5823.666] (-5848.128) (-5856.179) (-5834.340) -- 10:27:19      1320000 -- (-5834.283) (-5863.338) (-5843.138) [-5829.647] * (-5831.252) (-5842.158) (-5850.920) [-5819.254] -- 10:27:13      Average standard deviation of split frequencies: 0.016223      1321000 -- (-5830.154) (-5852.476) [-5835.087] (-5847.241) * [-5804.038] (-5844.274) (-5833.302) (-5839.411) -- 10:27:06      1322000 -- (-5825.640) (-5856.970) [-5823.953] (-5844.276) * [-5798.604] (-5827.878) (-5832.971) (-5845.043) -- 10:26:59      1323000 -- (-5839.973) (-5847.406) [-5825.910] (-5863.616) * [-5817.724] (-5838.626) (-5827.911) (-5840.512) -- 10:27:00      1324000 -- (-5830.389) (-5838.887) [-5835.354] (-5876.205) * [-5814.604] (-5853.205) (-5838.165) (-5857.227) -- 10:26:53      1325000 -- [-5827.730] (-5848.042) (-5845.366) (-5872.443) * (-5827.603) [-5826.887] (-5850.237) (-5825.860) -- 10:26:46      Average standard deviation of split frequencies: 0.015996      1326000 -- (-5838.338) (-5870.386) [-5832.587] (-5857.800) * (-5843.306) (-5877.000) (-5844.858) [-5824.139] -- 10:26:40      1327000 -- (-5834.458) (-5857.418) [-5824.611] (-5851.261) * (-5833.182) (-5855.329) [-5849.026] (-5823.934) -- 10:26:40      1328000 -- (-5838.617) (-5856.242) [-5826.426] (-5854.127) * [-5831.027] (-5841.159) (-5878.522) (-5820.593) -- 10:26:33      1329000 -- (-5834.377) (-5861.464) [-5825.021] (-5847.094) * [-5817.549] (-5859.473) (-5865.968) (-5829.708) -- 10:26:27      1330000 -- [-5823.612] (-5850.989) (-5825.623) (-5852.532) * [-5810.824] (-5845.292) (-5856.846) (-5818.724) -- 10:26:20      Average standard deviation of split frequencies: 0.015701      1331000 -- [-5837.327] (-5855.562) (-5837.399) (-5821.738) * [-5831.054] (-5854.553) (-5840.153) (-5838.019) -- 10:26:20      1332000 -- (-5855.010) (-5875.219) [-5821.632] (-5817.142) * [-5820.564] (-5856.158) (-5830.943) (-5832.409) -- 10:26:14      1333000 -- (-5829.886) (-5841.498) (-5841.020) [-5828.415] * [-5818.719] (-5865.793) (-5844.922) (-5826.961) -- 10:26:07      1334000 -- (-5831.728) [-5828.257] (-5839.246) (-5826.489) * [-5831.081] (-5865.891) (-5843.865) (-5850.115) -- 10:26:01      1335000 -- (-5856.223) (-5840.521) (-5854.750) [-5816.907] * [-5829.540] (-5877.258) (-5850.139) (-5853.201) -- 10:26:01      Average standard deviation of split frequencies: 0.015385      1336000 -- (-5840.450) (-5840.913) (-5855.740) [-5821.896] * (-5834.744) (-5851.506) [-5834.196] (-5832.565) -- 10:25:54      1337000 -- (-5835.404) [-5840.788] (-5861.187) (-5839.512) * (-5846.298) (-5839.268) [-5824.466] (-5844.053) -- 10:25:48      1338000 -- (-5851.851) [-5850.441] (-5848.989) (-5839.950) * (-5844.864) (-5827.496) [-5825.378] (-5848.212) -- 10:25:41      1339000 -- (-5847.161) (-5858.132) (-5844.787) [-5839.370] * (-5860.324) [-5823.931] (-5843.646) (-5852.210) -- 10:25:41      1340000 -- [-5828.675] (-5838.180) (-5849.027) (-5845.490) * (-5846.252) [-5814.582] (-5844.361) (-5846.713) -- 10:25:35      Average standard deviation of split frequencies: 0.015445      1341000 -- (-5852.634) (-5830.160) [-5843.297] (-5841.910) * (-5872.767) (-5837.584) [-5837.997] (-5834.538) -- 10:25:28      1342000 -- (-5848.991) [-5829.522] (-5845.914) (-5849.386) * (-5866.457) [-5826.935] (-5848.018) (-5836.766) -- 10:25:22      1343000 -- [-5831.950] (-5831.545) (-5846.525) (-5862.441) * (-5860.699) (-5823.964) [-5836.339] (-5824.416) -- 10:25:15      1344000 -- (-5832.612) [-5835.056] (-5839.886) (-5863.727) * (-5850.652) (-5829.825) (-5830.844) [-5822.032] -- 10:25:15      1345000 -- (-5845.763) [-5825.638] (-5861.086) (-5844.742) * [-5833.490] (-5839.900) (-5826.549) (-5835.235) -- 10:25:09      Average standard deviation of split frequencies: 0.015122      1346000 -- (-5827.491) [-5844.725] (-5850.226) (-5848.689) * (-5837.988) (-5841.253) (-5836.101) [-5819.902] -- 10:25:02      1347000 -- [-5822.206] (-5835.881) (-5851.339) (-5831.328) * (-5825.318) (-5841.106) (-5839.404) [-5826.619] -- 10:24:56      1348000 -- (-5830.423) [-5832.033] (-5862.779) (-5844.110) * (-5813.786) (-5851.240) (-5833.322) [-5826.483] -- 10:24:49      1349000 -- (-5822.164) (-5840.648) (-5876.119) [-5842.804] * [-5824.711] (-5861.582) (-5837.358) (-5834.373) -- 10:24:43      1350000 -- (-5831.402) [-5839.075] (-5872.769) (-5864.273) * (-5825.886) (-5881.385) (-5837.974) [-5828.337] -- 10:24:43      Average standard deviation of split frequencies: 0.014980      1351000 -- [-5822.050] (-5859.723) (-5860.700) (-5841.042) * (-5832.355) (-5853.706) (-5829.410) [-5833.888] -- 10:24:36      1352000 -- [-5821.385] (-5873.367) (-5848.239) (-5832.562) * [-5827.815] (-5852.926) (-5832.373) (-5821.395) -- 10:24:30      1353000 -- [-5821.246] (-5863.813) (-5843.715) (-5831.910) * [-5828.909] (-5840.915) (-5831.818) (-5824.056) -- 10:24:23      1354000 -- (-5840.442) (-5843.047) (-5860.088) [-5831.536] * (-5847.944) (-5867.992) (-5836.756) [-5823.612] -- 10:24:17      1355000 -- (-5823.129) (-5831.124) (-5849.811) [-5830.197] * (-5833.851) [-5822.005] (-5840.946) (-5838.746) -- 10:24:17      Average standard deviation of split frequencies: 0.014987      1356000 -- (-5837.305) [-5821.965] (-5859.089) (-5834.523) * [-5828.513] (-5834.556) (-5845.562) (-5848.179) -- 10:24:10      1357000 -- (-5843.290) (-5826.797) (-5864.455) [-5835.873] * (-5834.790) [-5850.470] (-5827.587) (-5858.213) -- 10:24:04      1358000 -- (-5839.933) [-5826.324] (-5840.157) (-5836.640) * (-5835.728) (-5853.223) [-5837.285] (-5851.135) -- 10:24:04      1359000 -- (-5836.330) [-5820.296] (-5835.452) (-5849.643) * [-5827.892] (-5833.645) (-5839.739) (-5848.928) -- 10:23:57      1360000 -- (-5862.657) (-5834.059) [-5831.129] (-5862.457) * (-5846.676) (-5842.237) (-5825.147) [-5816.657] -- 10:23:51      Average standard deviation of split frequencies: 0.014676      1361000 -- (-5853.265) (-5832.312) [-5820.686] (-5877.006) * (-5833.459) (-5871.919) (-5830.512) [-5818.458] -- 10:23:45      1362000 -- [-5834.615] (-5824.965) (-5849.793) (-5890.325) * [-5830.435] (-5852.457) (-5828.598) (-5825.769) -- 10:23:44      1363000 -- (-5841.290) [-5835.774] (-5847.366) (-5847.791) * (-5840.461) (-5857.695) [-5828.395] (-5821.968) -- 10:23:38      1364000 -- (-5849.525) (-5838.888) (-5839.439) [-5824.647] * (-5833.630) (-5853.354) (-5831.548) [-5824.765] -- 10:23:32      1365000 -- (-5856.228) [-5837.171] (-5828.129) (-5838.275) * (-5827.081) (-5861.052) [-5843.107] (-5838.000) -- 10:23:25      Average standard deviation of split frequencies: 0.014355      1366000 -- (-5860.930) (-5843.639) [-5823.115] (-5843.292) * (-5840.294) (-5856.355) (-5835.088) [-5835.670] -- 10:23:25      1367000 -- (-5860.321) (-5847.700) [-5817.792] (-5847.165) * (-5824.395) (-5832.847) [-5826.485] (-5835.406) -- 10:23:19      1368000 -- (-5844.476) (-5832.240) [-5822.741] (-5846.549) * (-5814.713) (-5852.049) [-5822.871] (-5826.884) -- 10:23:12      1369000 -- (-5854.008) [-5832.982] (-5824.355) (-5859.261) * [-5818.121] (-5844.733) (-5835.454) (-5826.025) -- 10:23:12      1370000 -- (-5855.189) (-5837.230) [-5826.844] (-5851.542) * (-5828.747) (-5850.263) [-5827.370] (-5833.355) -- 10:23:06      Average standard deviation of split frequencies: 0.014187      1371000 -- (-5858.930) (-5821.078) [-5826.614] (-5827.408) * (-5836.934) (-5847.388) [-5816.130] (-5834.177) -- 10:22:59      1372000 -- (-5864.906) [-5830.730] (-5829.047) (-5833.136) * (-5842.159) (-5867.727) [-5817.908] (-5817.308) -- 10:22:59      1373000 -- (-5860.809) [-5820.078] (-5851.859) (-5833.229) * (-5840.340) (-5852.890) (-5840.411) [-5820.307] -- 10:22:53      1374000 -- (-5849.928) (-5842.764) (-5862.480) [-5827.854] * (-5842.938) (-5830.246) (-5855.631) [-5818.562] -- 10:22:46      1375000 -- (-5864.805) (-5842.186) (-5850.843) [-5833.687] * (-5835.003) (-5825.812) (-5856.447) [-5820.544] -- 10:22:46      Average standard deviation of split frequencies: 0.014028      1376000 -- (-5865.392) [-5819.852] (-5843.313) (-5854.684) * (-5842.085) (-5838.896) (-5855.837) [-5816.251] -- 10:22:40      1377000 -- (-5851.871) [-5822.070] (-5833.337) (-5858.251) * (-5845.872) (-5834.157) (-5870.959) [-5823.610] -- 10:22:40      1378000 -- (-5853.436) (-5844.641) [-5823.933] (-5829.734) * (-5867.920) (-5821.898) (-5880.872) [-5821.554] -- 10:22:33      1379000 -- (-5838.964) (-5847.099) [-5812.890] (-5841.703) * (-5858.846) (-5825.613) (-5880.051) [-5826.242] -- 10:22:27      1380000 -- (-5841.407) (-5873.655) [-5819.133] (-5850.288) * (-5842.674) [-5836.252] (-5880.523) (-5816.544) -- 10:22:20      Average standard deviation of split frequencies: 0.013738      1381000 -- (-5835.720) (-5847.518) [-5822.581] (-5845.171) * (-5865.647) (-5838.598) (-5877.554) [-5835.660] -- 10:22:20      1382000 -- (-5845.153) (-5834.399) [-5817.807] (-5866.906) * (-5853.285) [-5839.589] (-5849.273) (-5846.340) -- 10:22:14      1383000 -- (-5841.183) (-5845.098) [-5825.394] (-5869.418) * [-5843.908] (-5852.244) (-5862.950) (-5847.216) -- 10:22:07      1384000 -- (-5844.428) (-5845.911) (-5830.746) [-5840.358] * (-5858.044) (-5839.470) (-5853.699) [-5826.814] -- 10:22:01      1385000 -- (-5857.356) [-5832.776] (-5822.668) (-5873.948) * (-5863.169) [-5834.922] (-5860.627) (-5830.898) -- 10:22:01      Average standard deviation of split frequencies: 0.013487      1386000 -- (-5871.275) (-5843.857) [-5829.348] (-5838.420) * (-5868.076) [-5823.480] (-5844.374) (-5841.681) -- 10:21:54      1387000 -- (-5870.184) (-5832.944) [-5828.138] (-5848.235) * (-5851.741) (-5846.283) [-5841.636] (-5839.998) -- 10:21:48      1388000 -- (-5858.862) (-5833.201) (-5837.320) [-5827.983] * (-5840.615) [-5821.766] (-5851.175) (-5831.193) -- 10:21:48      1389000 -- (-5877.873) (-5828.707) (-5886.534) [-5816.488] * (-5838.286) [-5818.106] (-5845.094) (-5830.046) -- 10:21:41      1390000 -- (-5857.484) (-5823.700) (-5866.969) [-5824.108] * [-5842.940] (-5830.068) (-5839.819) (-5825.643) -- 10:21:35      Average standard deviation of split frequencies: 0.013380      1391000 -- (-5853.751) [-5830.290] (-5881.014) (-5829.571) * (-5826.144) (-5842.846) (-5844.093) [-5823.931] -- 10:21:29      1392000 -- (-5858.855) (-5831.960) (-5846.549) [-5821.750] * (-5819.252) (-5847.147) [-5823.773] (-5825.422) -- 10:21:28      1393000 -- (-5835.098) (-5835.984) (-5840.326) [-5829.530] * (-5850.968) (-5849.066) (-5829.845) [-5820.934] -- 10:21:22      1394000 -- (-5851.968) (-5807.007) (-5839.313) [-5821.785] * (-5833.965) (-5864.302) (-5833.158) [-5807.844] -- 10:21:16      1395000 -- (-5850.812) [-5826.975] (-5821.527) (-5838.920) * (-5843.128) [-5840.335] (-5856.071) (-5814.255) -- 10:21:15      Average standard deviation of split frequencies: 0.013433      1396000 -- (-5862.423) (-5816.622) (-5840.450) [-5829.397] * (-5843.670) (-5847.709) (-5868.759) [-5829.804] -- 10:21:09      1397000 -- (-5841.929) [-5802.936] (-5835.404) (-5832.710) * (-5834.909) (-5815.859) (-5856.555) [-5815.439] -- 10:21:03      1398000 -- (-5838.784) [-5807.528] (-5835.533) (-5828.371) * (-5824.281) [-5826.780] (-5846.461) (-5829.882) -- 10:21:03      1399000 -- (-5840.271) (-5811.058) [-5834.616] (-5841.947) * (-5847.551) [-5825.672] (-5845.538) (-5833.391) -- 10:20:56      1400000 -- (-5842.553) (-5841.808) (-5841.421) [-5821.358] * (-5853.192) [-5832.717] (-5836.353) (-5836.501) -- 10:20:50      Average standard deviation of split frequencies: 0.013573      1401000 -- (-5860.004) (-5838.367) (-5831.557) [-5836.154] * (-5856.154) (-5821.107) [-5820.960] (-5844.673) -- 10:20:50      1402000 -- (-5845.575) [-5843.921] (-5855.379) (-5840.092) * (-5850.412) (-5824.925) [-5827.411] (-5840.460) -- 10:20:43      1403000 -- (-5840.378) (-5855.100) [-5850.373] (-5836.088) * (-5853.546) (-5827.730) [-5815.661] (-5849.824) -- 10:20:37      1404000 -- [-5832.439] (-5864.996) (-5824.755) (-5827.695) * (-5855.158) [-5823.674] (-5822.175) (-5843.232) -- 10:20:37      1405000 -- (-5850.470) (-5837.651) (-5832.512) [-5836.403] * [-5831.864] (-5840.077) (-5821.171) (-5848.227) -- 10:20:30      Average standard deviation of split frequencies: 0.013559      1406000 -- (-5856.216) (-5847.329) [-5823.025] (-5852.201) * [-5831.707] (-5833.943) (-5854.104) (-5837.103) -- 10:20:24      1407000 -- (-5858.959) (-5854.531) [-5829.644] (-5834.630) * (-5839.898) (-5816.549) [-5838.506] (-5844.488) -- 10:20:18      1408000 -- [-5820.500] (-5834.631) (-5829.490) (-5837.233) * (-5835.432) [-5818.193] (-5866.812) (-5847.911) -- 10:20:17      1409000 -- (-5846.174) [-5836.245] (-5831.205) (-5842.321) * (-5842.446) [-5838.991] (-5851.275) (-5843.812) -- 10:20:11      1410000 -- (-5844.533) (-5833.663) (-5826.772) [-5822.000] * (-5851.376) (-5840.996) [-5831.479] (-5830.287) -- 10:20:05      Average standard deviation of split frequencies: 0.013360      1411000 -- (-5835.444) (-5828.390) [-5824.376] (-5849.593) * [-5825.518] (-5847.891) (-5824.419) (-5848.931) -- 10:20:04      1412000 -- [-5835.690] (-5828.051) (-5826.033) (-5842.920) * (-5835.095) (-5824.526) [-5814.582] (-5851.088) -- 10:19:58      1413000 -- (-5846.355) [-5828.965] (-5833.598) (-5843.393) * (-5829.615) [-5825.104] (-5810.693) (-5839.163) -- 10:19:52      1414000 -- [-5831.190] (-5836.047) (-5840.875) (-5862.503) * (-5857.681) (-5831.513) [-5824.538] (-5838.762) -- 10:19:51      1415000 -- [-5833.906] (-5840.825) (-5827.479) (-5863.448) * (-5845.939) [-5833.085] (-5839.318) (-5832.034) -- 10:19:45      Average standard deviation of split frequencies: 0.013310      1416000 -- (-5829.896) [-5818.096] (-5832.661) (-5855.633) * (-5842.924) (-5847.278) (-5829.090) [-5822.139] -- 10:19:39      1417000 -- (-5827.906) [-5818.867] (-5847.041) (-5864.155) * (-5849.636) (-5828.242) [-5833.610] (-5833.157) -- 10:19:32      1418000 -- (-5836.651) [-5824.504] (-5842.458) (-5843.631) * (-5838.269) [-5821.282] (-5864.735) (-5834.466) -- 10:19:32      1419000 -- (-5837.331) [-5834.838] (-5839.469) (-5835.805) * (-5852.856) (-5830.412) (-5863.749) [-5825.591] -- 10:19:26      1420000 -- (-5833.353) (-5834.588) [-5839.931] (-5850.186) * (-5857.073) (-5833.246) (-5847.965) [-5820.609] -- 10:19:19      Average standard deviation of split frequencies: 0.013200      1421000 -- (-5837.306) (-5840.925) [-5828.236] (-5852.155) * (-5865.902) (-5855.632) [-5825.577] (-5833.324) -- 10:19:19      1422000 -- (-5856.868) [-5826.640] (-5845.638) (-5848.615) * (-5862.544) (-5838.099) [-5823.569] (-5853.971) -- 10:19:13      1423000 -- (-5840.541) (-5819.358) [-5833.251] (-5842.005) * (-5851.365) (-5843.402) [-5838.354] (-5853.061) -- 10:19:06      1424000 -- (-5843.067) [-5818.052] (-5858.431) (-5835.035) * (-5855.757) [-5825.576] (-5837.672) (-5844.721) -- 10:19:06      1425000 -- (-5856.018) (-5841.805) (-5855.125) [-5831.801] * (-5863.829) (-5826.348) [-5830.089] (-5840.796) -- 10:19:00      Average standard deviation of split frequencies: 0.013211      1426000 -- (-5880.324) (-5821.538) (-5871.757) [-5827.108] * (-5868.068) (-5828.921) (-5840.187) [-5830.143] -- 10:18:53      1427000 -- (-5847.215) [-5840.401] (-5846.423) (-5843.365) * (-5858.139) [-5828.686] (-5846.440) (-5844.694) -- 10:18:53      1428000 -- (-5866.163) [-5824.779] (-5841.612) (-5838.659) * (-5845.102) [-5839.589] (-5840.486) (-5855.284) -- 10:18:47      1429000 -- (-5843.875) [-5828.065] (-5848.857) (-5852.451) * (-5844.320) [-5829.470] (-5843.268) (-5872.337) -- 10:18:41      1430000 -- (-5822.136) [-5838.962] (-5822.958) (-5857.916) * (-5847.789) [-5815.484] (-5849.957) (-5833.724) -- 10:18:40      Average standard deviation of split frequencies: 0.013018      1431000 -- (-5857.175) (-5841.777) [-5811.994] (-5852.019) * (-5852.404) (-5835.105) (-5844.942) [-5818.698] -- 10:18:34      1432000 -- (-5867.727) (-5845.570) [-5811.282] (-5836.395) * (-5831.780) (-5853.732) (-5833.190) [-5806.730] -- 10:18:28      1433000 -- (-5854.376) (-5851.352) [-5823.208] (-5840.916) * (-5833.286) (-5887.143) (-5844.259) [-5804.970] -- 10:18:27      1434000 -- (-5861.734) (-5857.983) [-5825.842] (-5839.117) * (-5843.113) (-5868.525) (-5836.157) [-5812.697] -- 10:18:21      1435000 -- (-5876.138) (-5845.577) [-5820.569] (-5849.111) * (-5837.458) (-5847.781) (-5844.101) [-5824.764] -- 10:18:15      Average standard deviation of split frequencies: 0.012797      1436000 -- (-5868.555) (-5850.809) [-5833.123] (-5851.106) * (-5843.722) [-5825.184] (-5847.738) (-5826.496) -- 10:18:14      1437000 -- (-5849.429) (-5835.370) [-5826.152] (-5853.929) * (-5849.702) (-5829.558) [-5840.615] (-5828.602) -- 10:18:08      1438000 -- (-5838.625) (-5853.164) [-5829.309] (-5864.971) * [-5835.777] (-5857.186) (-5846.006) (-5838.306) -- 10:18:02      1439000 -- [-5838.869] (-5851.024) (-5847.428) (-5851.859) * (-5845.424) (-5840.728) (-5854.021) [-5821.172] -- 10:17:55      1440000 -- [-5825.194] (-5840.256) (-5847.286) (-5855.061) * (-5847.536) [-5835.538] (-5862.524) (-5839.538) -- 10:17:55      Average standard deviation of split frequencies: 0.012791      1441000 -- (-5817.979) [-5826.800] (-5852.974) (-5843.788) * (-5845.221) (-5838.171) (-5836.835) [-5836.001] -- 10:17:49      1442000 -- [-5813.519] (-5822.311) (-5853.735) (-5844.319) * (-5856.240) (-5854.886) (-5849.631) [-5829.680] -- 10:17:42      1443000 -- [-5818.210] (-5826.537) (-5845.064) (-5842.200) * (-5845.419) (-5857.871) (-5826.217) [-5820.440] -- 10:17:42      1444000 -- (-5834.962) [-5823.216] (-5843.041) (-5863.100) * (-5847.284) (-5845.178) [-5826.442] (-5824.538) -- 10:17:36      1445000 -- [-5817.390] (-5836.401) (-5834.026) (-5853.933) * (-5843.453) (-5845.898) [-5813.790] (-5823.818) -- 10:17:29      Average standard deviation of split frequencies: 0.012922      1446000 -- [-5818.188] (-5839.413) (-5837.891) (-5867.402) * (-5835.128) (-5873.269) [-5831.231] (-5836.933) -- 10:17:23      1447000 -- (-5838.746) (-5826.439) [-5821.587] (-5870.525) * (-5832.163) (-5861.222) [-5824.331] (-5833.810) -- 10:17:17      1448000 -- (-5829.055) (-5852.281) [-5843.290] (-5857.897) * (-5841.936) (-5869.938) [-5824.332] (-5842.158) -- 10:17:17      1449000 -- [-5818.093] (-5840.471) (-5855.476) (-5860.854) * (-5844.888) (-5854.186) [-5826.259] (-5856.764) -- 10:17:10      1450000 -- [-5811.954] (-5830.048) (-5839.526) (-5858.907) * (-5824.239) (-5850.271) [-5821.982] (-5857.823) -- 10:17:04      Average standard deviation of split frequencies: 0.012703      1451000 -- [-5814.519] (-5852.407) (-5830.891) (-5832.723) * (-5845.336) (-5846.880) [-5820.450] (-5850.567) -- 10:16:58      1452000 -- [-5823.004] (-5837.451) (-5837.324) (-5849.182) * (-5871.528) (-5826.179) [-5820.296] (-5835.450) -- 10:16:57      1453000 -- (-5863.420) [-5835.862] (-5826.864) (-5856.711) * (-5859.932) (-5837.673) [-5817.010] (-5847.600) -- 10:16:51      1454000 -- (-5849.152) [-5837.898] (-5831.183) (-5863.035) * (-5837.667) (-5850.479) [-5817.855] (-5847.065) -- 10:16:45      1455000 -- [-5820.145] (-5819.405) (-5860.604) (-5853.626) * (-5858.042) (-5837.268) [-5821.384] (-5852.860) -- 10:16:44      Average standard deviation of split frequencies: 0.012792      1456000 -- (-5817.767) [-5831.992] (-5854.485) (-5853.048) * (-5848.438) (-5842.524) (-5836.779) [-5832.613] -- 10:16:38      1457000 -- (-5829.015) [-5824.914] (-5875.651) (-5850.376) * (-5843.954) (-5841.413) (-5832.615) [-5823.376] -- 10:16:32      1458000 -- (-5846.848) (-5839.482) [-5835.593] (-5839.142) * (-5852.023) (-5848.366) [-5834.247] (-5836.695) -- 10:16:26      1459000 -- (-5824.693) (-5852.432) [-5841.477] (-5847.657) * (-5849.389) (-5843.453) [-5830.732] (-5836.573) -- 10:16:25      1460000 -- [-5825.328] (-5861.909) (-5824.039) (-5836.913) * (-5864.002) (-5837.816) [-5838.717] (-5837.181) -- 10:16:19      Average standard deviation of split frequencies: 0.012639      1461000 -- (-5820.585) [-5839.233] (-5848.923) (-5855.239) * (-5871.512) [-5826.071] (-5850.925) (-5839.659) -- 10:16:13      1462000 -- [-5818.177] (-5836.989) (-5832.262) (-5833.065) * (-5852.875) (-5825.004) (-5847.841) [-5829.265] -- 10:16:12      1463000 -- [-5827.585] (-5838.713) (-5836.463) (-5852.551) * [-5832.313] (-5846.484) (-5846.008) (-5832.334) -- 10:16:06      1464000 -- (-5840.443) (-5837.261) [-5836.407] (-5848.442) * (-5844.939) (-5844.340) (-5836.308) [-5835.230] -- 10:16:00      1465000 -- (-5842.963) [-5815.708] (-5840.286) (-5852.351) * [-5826.356] (-5850.709) (-5835.947) (-5835.930) -- 10:15:53      Average standard deviation of split frequencies: 0.012553      1466000 -- (-5826.959) [-5816.025] (-5836.771) (-5869.257) * (-5816.749) (-5852.630) (-5832.319) [-5841.186] -- 10:15:47      1467000 -- (-5820.945) [-5817.032] (-5839.280) (-5878.168) * (-5851.459) (-5850.193) (-5860.313) [-5825.317] -- 10:15:47      1468000 -- (-5830.648) [-5822.270] (-5848.139) (-5866.173) * (-5840.323) (-5856.877) (-5842.197) [-5810.549] -- 10:15:41      1469000 -- (-5821.847) [-5808.950] (-5836.340) (-5866.975) * (-5834.966) (-5866.743) (-5853.393) [-5815.923] -- 10:15:34      1470000 -- (-5839.583) [-5827.751] (-5836.469) (-5853.835) * (-5831.570) (-5843.954) (-5863.137) [-5814.703] -- 10:15:28      Average standard deviation of split frequencies: 0.012560      1471000 -- (-5853.197) [-5819.176] (-5839.661) (-5858.341) * (-5855.967) [-5828.303] (-5853.476) (-5818.915) -- 10:15:28      1472000 -- [-5844.636] (-5826.139) (-5871.165) (-5859.263) * (-5851.544) (-5846.652) (-5854.465) [-5820.641] -- 10:15:21      1473000 -- (-5829.498) [-5827.255] (-5858.259) (-5832.575) * (-5842.355) (-5847.303) (-5852.072) [-5820.261] -- 10:15:15      1474000 -- (-5835.090) [-5814.309] (-5859.097) (-5831.673) * (-5852.015) (-5830.085) (-5840.178) [-5816.584] -- 10:15:09      1475000 -- (-5835.514) [-5819.331] (-5866.381) (-5840.972) * (-5864.092) [-5835.804] (-5847.169) (-5838.098) -- 10:15:08      Average standard deviation of split frequencies: 0.012607      1476000 -- (-5844.543) [-5818.833] (-5867.100) (-5842.607) * [-5842.989] (-5841.413) (-5841.516) (-5831.697) -- 10:15:02      1477000 -- (-5837.605) [-5822.718] (-5840.699) (-5850.202) * (-5853.016) (-5851.248) (-5833.065) [-5825.056] -- 10:14:56      1478000 -- (-5831.705) [-5817.136] (-5853.196) (-5842.811) * [-5838.954] (-5839.263) (-5852.227) (-5823.007) -- 10:14:50      1479000 -- (-5826.653) [-5818.839] (-5864.575) (-5840.281) * (-5880.753) (-5844.842) (-5860.571) [-5816.244] -- 10:14:49      1480000 -- (-5811.204) (-5841.624) (-5875.958) [-5823.314] * (-5825.009) (-5853.256) (-5843.505) [-5811.211] -- 10:14:43      Average standard deviation of split frequencies: 0.012758      1481000 -- (-5829.858) [-5825.723] (-5846.313) (-5830.908) * (-5838.293) (-5845.971) (-5830.219) [-5813.425] -- 10:14:37      1482000 -- [-5824.787] (-5841.388) (-5833.455) (-5831.278) * (-5827.071) (-5841.054) (-5841.234) [-5832.240] -- 10:14:36      1483000 -- [-5822.400] (-5826.983) (-5843.463) (-5838.275) * [-5824.764] (-5844.118) (-5819.645) (-5861.444) -- 10:14:30      1484000 -- (-5835.497) (-5844.680) (-5858.843) [-5837.959] * (-5833.624) (-5847.817) [-5808.706] (-5853.900) -- 10:14:24      1485000 -- (-5829.582) (-5826.844) (-5865.936) [-5826.339] * (-5829.231) (-5856.718) [-5815.434] (-5838.195) -- 10:14:23      Average standard deviation of split frequencies: 0.012805      1486000 -- [-5812.053] (-5831.337) (-5854.597) (-5825.614) * (-5825.653) (-5860.305) [-5806.317] (-5811.621) -- 10:14:17      1487000 -- (-5827.305) (-5841.597) (-5870.290) [-5822.542] * (-5827.115) (-5880.340) [-5811.320] (-5831.647) -- 10:14:11      1488000 -- (-5839.641) (-5832.187) (-5866.475) [-5819.429] * (-5836.625) (-5857.258) [-5817.974] (-5837.693) -- 10:14:11      1489000 -- (-5855.232) [-5820.179] (-5879.047) (-5823.981) * (-5835.375) (-5851.236) [-5820.307] (-5831.239) -- 10:14:04      1490000 -- (-5859.700) [-5833.026] (-5889.148) (-5831.284) * (-5856.050) (-5844.216) (-5829.290) [-5822.383] -- 10:13:58      Average standard deviation of split frequencies: 0.012598      1491000 -- (-5856.672) (-5837.937) (-5863.074) [-5816.874] * (-5838.866) [-5809.916] (-5850.995) (-5830.381) -- 10:13:52      1492000 -- (-5853.350) (-5846.467) (-5877.233) [-5840.252] * (-5869.746) (-5814.125) [-5820.776] (-5842.086) -- 10:13:51      1493000 -- (-5850.087) [-5826.231] (-5871.063) (-5827.697) * (-5838.676) [-5812.505] (-5829.623) (-5854.346) -- 10:13:45      1494000 -- (-5829.647) (-5827.786) (-5871.478) [-5825.545] * (-5849.986) (-5836.906) [-5820.376] (-5870.944) -- 10:13:39      1495000 -- (-5830.169) (-5832.703) (-5864.469) [-5828.035] * [-5834.899] (-5845.887) (-5815.380) (-5836.307) -- 10:13:38      Average standard deviation of split frequencies: 0.012536      1496000 -- (-5846.203) [-5830.141] (-5867.388) (-5839.946) * (-5834.199) (-5844.059) [-5842.065] (-5830.218) -- 10:13:32      1497000 -- (-5836.162) (-5839.675) (-5863.477) [-5814.810] * (-5844.035) [-5838.472] (-5845.538) (-5824.793) -- 10:13:26      1498000 -- (-5828.819) (-5810.253) (-5855.779) [-5833.583] * (-5832.238) (-5847.156) (-5834.315) [-5831.022] -- 10:13:20      1499000 -- (-5828.604) [-5811.829] (-5849.782) (-5859.386) * (-5840.327) (-5842.930) (-5843.290) [-5841.214] -- 10:13:19      1500000 -- (-5847.900) [-5811.675] (-5862.715) (-5850.156) * (-5845.555) (-5849.763) (-5856.429) [-5825.189] -- 10:13:13      Average standard deviation of split frequencies: 0.012554      1501000 -- (-5837.535) [-5813.604] (-5862.801) (-5852.655) * [-5830.577] (-5863.606) (-5858.899) (-5838.100) -- 10:13:07      1502000 -- (-5845.406) [-5818.397] (-5858.679) (-5857.867) * [-5818.969] (-5834.285) (-5851.089) (-5850.044) -- 10:13:06      1503000 -- (-5838.345) [-5817.473] (-5858.434) (-5884.076) * (-5824.731) (-5840.341) (-5852.216) [-5827.830] -- 10:13:00      1504000 -- (-5831.894) [-5826.299] (-5852.554) (-5870.749) * [-5835.467] (-5841.596) (-5847.527) (-5840.116) -- 10:12:54      1505000 -- [-5827.318] (-5841.313) (-5866.645) (-5880.905) * [-5832.919] (-5844.430) (-5855.172) (-5853.869) -- 10:12:48      Average standard deviation of split frequencies: 0.012475      1506000 -- [-5833.746] (-5840.003) (-5856.334) (-5861.089) * (-5827.689) (-5846.713) (-5838.380) [-5834.985] -- 10:12:47      1507000 -- (-5822.509) [-5834.345] (-5842.947) (-5852.230) * (-5828.687) (-5864.714) (-5836.719) [-5821.218] -- 10:12:41      1508000 -- (-5810.702) [-5838.527] (-5839.069) (-5846.864) * [-5823.699] (-5849.811) (-5838.698) (-5831.506) -- 10:12:35      1509000 -- [-5824.966] (-5852.607) (-5824.523) (-5848.027) * (-5832.000) (-5841.052) (-5859.803) [-5811.880] -- 10:12:34      1510000 -- (-5817.326) (-5840.673) [-5819.693] (-5854.430) * (-5835.678) (-5853.566) (-5860.071) [-5823.493] -- 10:12:28      Average standard deviation of split frequencies: 0.012692      1511000 -- (-5857.356) (-5828.676) [-5830.273] (-5847.543) * (-5850.765) [-5840.841] (-5857.956) (-5834.716) -- 10:12:22      1512000 -- [-5845.453] (-5846.486) (-5826.443) (-5846.551) * [-5820.864] (-5832.476) (-5830.401) (-5855.282) -- 10:12:22      1513000 -- (-5842.327) (-5854.989) [-5824.186] (-5855.907) * (-5838.422) (-5844.744) [-5822.579] (-5868.186) -- 10:12:15      1514000 -- [-5822.120] (-5847.491) (-5843.301) (-5865.029) * [-5831.602] (-5849.983) (-5821.522) (-5848.997) -- 10:12:09      1515000 -- (-5831.139) [-5821.201] (-5851.149) (-5845.377) * (-5835.810) (-5822.207) [-5828.241] (-5852.961) -- 10:12:09      Average standard deviation of split frequencies: 0.012353      1516000 -- (-5828.079) [-5818.281] (-5854.677) (-5849.478) * (-5855.205) (-5827.326) [-5818.018] (-5852.336) -- 10:12:02      1517000 -- (-5836.000) [-5826.440] (-5850.571) (-5833.783) * (-5844.334) (-5828.051) [-5811.690] (-5856.910) -- 10:11:56      1518000 -- (-5843.006) (-5816.719) (-5858.590) [-5844.456] * (-5848.242) [-5829.773] (-5834.413) (-5851.865) -- 10:11:56      1519000 -- (-5857.904) [-5812.665] (-5860.198) (-5830.259) * (-5828.319) (-5825.818) [-5812.572] (-5867.426) -- 10:11:50      1520000 -- (-5837.573) [-5822.610] (-5873.479) (-5849.626) * (-5862.634) (-5832.985) [-5822.199] (-5837.952) -- 10:11:43      Average standard deviation of split frequencies: 0.012090      1521000 -- (-5825.717) [-5810.892] (-5880.276) (-5864.231) * (-5864.867) (-5838.289) (-5835.064) [-5829.532] -- 10:11:37      1522000 -- (-5843.923) (-5826.504) (-5887.777) [-5844.963] * (-5857.635) [-5835.417] (-5856.539) (-5828.745) -- 10:11:37      1523000 -- (-5856.145) [-5813.758] (-5878.876) (-5824.135) * (-5857.007) [-5835.922] (-5859.055) (-5824.250) -- 10:11:30      1524000 -- (-5830.357) [-5817.273] (-5870.055) (-5832.917) * (-5842.484) (-5832.479) (-5858.813) [-5820.434] -- 10:11:24      1525000 -- [-5833.130] (-5820.886) (-5868.243) (-5851.914) * (-5840.959) (-5824.548) (-5840.469) [-5825.037] -- 10:11:18      Average standard deviation of split frequencies: 0.011818      1526000 -- (-5848.059) (-5851.267) (-5849.073) [-5836.211] * (-5847.410) (-5841.838) (-5836.580) [-5814.106] -- 10:11:18      1527000 -- (-5850.859) (-5842.552) (-5857.145) [-5834.075] * (-5847.403) (-5839.870) [-5822.564] (-5844.854) -- 10:11:11      1528000 -- (-5866.657) (-5830.824) [-5845.721] (-5837.627) * (-5856.309) (-5830.578) [-5824.571] (-5830.705) -- 10:11:05      1529000 -- (-5864.795) [-5830.532] (-5862.288) (-5835.857) * (-5871.179) (-5850.799) (-5840.599) [-5828.790] -- 10:11:05      1530000 -- (-5841.199) [-5818.044] (-5849.507) (-5841.073) * (-5848.661) [-5832.046] (-5839.787) (-5837.753) -- 10:10:59      Average standard deviation of split frequencies: 0.011710      1531000 -- (-5848.662) [-5830.172] (-5861.757) (-5834.550) * (-5855.720) (-5842.505) [-5826.043] (-5831.290) -- 10:10:52      1532000 -- (-5866.463) (-5829.557) [-5817.682] (-5840.059) * (-5869.118) (-5840.187) [-5826.582] (-5839.145) -- 10:10:52      1533000 -- (-5859.633) (-5825.361) [-5824.351] (-5865.102) * (-5875.822) (-5838.389) [-5827.279] (-5848.526) -- 10:10:46      1534000 -- (-5845.564) (-5841.676) [-5812.483] (-5848.903) * (-5866.222) (-5842.335) (-5825.236) [-5830.695] -- 10:10:40      1535000 -- [-5823.390] (-5847.199) (-5827.036) (-5847.442) * (-5862.490) (-5842.045) [-5823.784] (-5828.890) -- 10:10:39      Average standard deviation of split frequencies: 0.011501      1536000 -- (-5816.403) (-5840.124) [-5815.202] (-5822.051) * (-5861.916) (-5836.024) [-5830.989] (-5833.583) -- 10:10:33      1537000 -- (-5823.501) (-5849.357) (-5822.591) [-5804.666] * (-5845.009) (-5851.458) [-5832.056] (-5834.867) -- 10:10:27      1538000 -- [-5824.622] (-5849.164) (-5831.382) (-5808.516) * (-5846.991) (-5854.713) [-5811.310] (-5825.166) -- 10:10:20      1539000 -- (-5839.833) (-5856.991) (-5828.193) [-5829.691] * (-5838.363) (-5828.654) (-5826.210) [-5822.813] -- 10:10:20      1540000 -- (-5826.249) (-5857.838) [-5820.518] (-5842.874) * (-5860.512) (-5810.860) (-5836.142) [-5817.095] -- 10:10:14      Average standard deviation of split frequencies: 0.011311      1541000 -- (-5839.633) (-5863.810) (-5844.301) [-5832.424] * (-5846.645) [-5805.629] (-5835.350) (-5833.698) -- 10:10:08      1542000 -- (-5833.790) (-5850.070) (-5851.612) [-5824.997] * (-5844.482) [-5819.749] (-5834.354) (-5832.578) -- 10:10:07      1543000 -- (-5840.544) (-5838.992) (-5841.383) [-5815.460] * [-5837.479] (-5834.537) (-5839.114) (-5835.064) -- 10:10:01      1544000 -- (-5855.371) (-5827.640) (-5843.663) [-5826.107] * [-5827.679] (-5871.326) (-5826.696) (-5833.099) -- 10:09:55      1545000 -- (-5840.029) (-5837.425) [-5829.650] (-5836.033) * [-5823.751] (-5866.136) (-5834.261) (-5829.854) -- 10:09:49      Average standard deviation of split frequencies: 0.011233      1546000 -- (-5833.407) (-5869.929) [-5829.471] (-5842.924) * [-5806.795] (-5861.539) (-5842.070) (-5826.141) -- 10:09:42      1547000 -- [-5823.589] (-5843.071) (-5814.186) (-5846.910) * (-5833.108) [-5832.330] (-5862.190) (-5823.369) -- 10:09:42      1548000 -- (-5835.308) (-5838.600) [-5808.209] (-5881.283) * (-5853.299) (-5835.632) (-5860.866) [-5825.200] -- 10:09:36      1549000 -- (-5827.987) (-5836.536) [-5806.036] (-5890.120) * (-5862.317) (-5830.774) (-5855.730) [-5824.137] -- 10:09:30      1550000 -- (-5832.698) (-5829.739) [-5825.123] (-5850.498) * (-5858.696) [-5837.272] (-5867.017) (-5831.417) -- 10:09:29      Average standard deviation of split frequencies: 0.011029      1551000 -- (-5845.678) (-5835.565) [-5828.603] (-5840.834) * (-5855.941) [-5832.131] (-5840.848) (-5847.023) -- 10:09:23      1552000 -- (-5826.881) (-5850.115) (-5837.701) [-5852.687] * (-5851.816) (-5837.389) (-5862.657) [-5840.870] -- 10:09:17      1553000 -- (-5836.426) [-5844.767] (-5843.105) (-5867.538) * (-5841.953) (-5848.182) (-5842.759) [-5844.450] -- 10:09:16      1554000 -- (-5851.594) (-5830.784) [-5838.506] (-5859.254) * (-5853.511) [-5841.717] (-5861.854) (-5840.892) -- 10:09:10      1555000 -- (-5855.766) [-5819.115] (-5832.095) (-5851.647) * (-5848.735) (-5835.002) (-5875.052) [-5834.523] -- 10:09:04      Average standard deviation of split frequencies: 0.010978      1556000 -- (-5855.346) [-5824.578] (-5837.013) (-5849.054) * (-5843.886) [-5825.035] (-5843.800) (-5827.029) -- 10:08:58      1557000 -- (-5827.998) [-5817.788] (-5861.415) (-5833.637) * (-5844.849) [-5820.425] (-5839.041) (-5824.066) -- 10:08:57      1558000 -- [-5817.153] (-5838.706) (-5852.448) (-5820.358) * (-5851.119) [-5808.700] (-5832.556) (-5828.631) -- 10:08:51      1559000 -- (-5836.141) (-5838.549) (-5881.047) [-5834.766] * (-5854.904) [-5819.374] (-5844.377) (-5827.976) -- 10:08:45      1560000 -- [-5818.655] (-5835.531) (-5843.617) (-5837.703) * (-5863.135) [-5826.506] (-5840.586) (-5822.683) -- 10:08:44      Average standard deviation of split frequencies: 0.011061      1561000 -- [-5824.584] (-5847.272) (-5857.399) (-5837.524) * (-5828.827) (-5835.060) (-5846.678) [-5821.868] -- 10:08:38      1562000 -- [-5833.719] (-5861.433) (-5877.156) (-5832.904) * (-5820.652) (-5842.547) (-5845.536) [-5817.299] -- 10:08:32      1563000 -- [-5834.631] (-5860.071) (-5858.840) (-5830.086) * (-5837.554) [-5820.376] (-5856.180) (-5813.673) -- 10:08:26      1564000 -- [-5825.996] (-5848.731) (-5863.998) (-5853.649) * (-5848.577) (-5817.880) (-5845.862) [-5808.444] -- 10:08:25      1565000 -- [-5816.772] (-5846.361) (-5850.982) (-5852.494) * (-5837.025) (-5820.316) (-5855.290) [-5806.349] -- 10:08:19      Average standard deviation of split frequencies: 0.010903      1566000 -- (-5814.797) (-5839.960) [-5832.350] (-5857.774) * (-5836.095) (-5810.691) (-5848.021) [-5833.214] -- 10:08:13      1567000 -- (-5811.776) [-5835.565] (-5853.368) (-5841.782) * [-5827.254] (-5825.546) (-5844.006) (-5856.387) -- 10:08:07      1568000 -- [-5817.816] (-5845.251) (-5840.139) (-5836.382) * (-5851.238) (-5834.152) [-5847.505] (-5847.513) -- 10:08:06      1569000 -- [-5815.713] (-5823.641) (-5849.881) (-5855.038) * (-5835.305) [-5824.682] (-5836.481) (-5851.327) -- 10:08:00      1570000 -- [-5831.620] (-5829.841) (-5847.804) (-5829.622) * (-5823.901) [-5827.854] (-5861.371) (-5848.943) -- 10:07:54      Average standard deviation of split frequencies: 0.010959      1571000 -- (-5836.880) [-5813.051] (-5851.874) (-5831.836) * [-5836.353] (-5834.251) (-5856.021) (-5841.485) -- 10:07:53      1572000 -- [-5833.336] (-5828.204) (-5870.235) (-5830.918) * (-5837.792) [-5822.953] (-5839.861) (-5838.970) -- 10:07:47      1573000 -- (-5829.053) (-5839.788) (-5853.009) [-5833.068] * [-5841.469] (-5841.828) (-5830.401) (-5863.487) -- 10:07:41      1574000 -- (-5843.459) (-5832.663) (-5850.175) [-5822.037] * (-5847.131) (-5821.012) [-5818.459] (-5849.555) -- 10:07:40      1575000 -- (-5851.060) (-5838.589) [-5826.153] (-5837.068) * (-5861.062) (-5835.084) [-5813.486] (-5862.403) -- 10:07:34      Average standard deviation of split frequencies: 0.011073      1576000 -- (-5851.373) [-5829.495] (-5850.185) (-5846.160) * (-5860.629) [-5825.796] (-5823.739) (-5851.770) -- 10:07:28      1577000 -- (-5879.109) (-5829.978) (-5869.948) [-5822.330] * (-5849.077) (-5820.359) [-5822.711] (-5851.566) -- 10:07:28      1578000 -- (-5859.886) (-5843.821) [-5864.256] (-5837.158) * (-5860.375) [-5810.531] (-5819.715) (-5839.276) -- 10:07:21      1579000 -- [-5839.184] (-5862.642) (-5841.122) (-5825.991) * (-5830.570) [-5821.125] (-5843.501) (-5855.534) -- 10:07:15      1580000 -- (-5841.027) (-5840.668) (-5862.678) [-5828.976] * (-5827.010) (-5830.626) [-5828.924] (-5852.297) -- 10:07:09      Average standard deviation of split frequencies: 0.011008      1581000 -- (-5841.788) [-5834.313] (-5843.427) (-5857.627) * (-5844.384) [-5836.371] (-5831.875) (-5837.046) -- 10:07:09      1582000 -- (-5855.555) (-5845.301) (-5841.459) [-5833.338] * [-5830.886] (-5860.362) (-5851.405) (-5833.422) -- 10:07:03      1583000 -- (-5877.289) (-5849.061) (-5845.653) [-5822.920] * (-5836.489) (-5844.769) (-5853.812) [-5819.196] -- 10:06:56      1584000 -- (-5866.833) (-5852.112) (-5845.644) [-5823.740] * (-5839.485) [-5829.033] (-5849.574) (-5825.836) -- 10:06:56      1585000 -- (-5876.153) [-5831.637] (-5841.447) (-5826.403) * (-5836.775) (-5844.554) (-5850.064) [-5829.670] -- 10:06:50      Average standard deviation of split frequencies: 0.010782      1586000 -- (-5846.745) (-5861.099) (-5827.660) [-5825.179] * (-5846.922) (-5850.854) (-5848.711) [-5815.670] -- 10:06:44      1587000 -- (-5868.863) (-5859.670) (-5840.308) [-5811.493] * [-5825.737] (-5856.704) (-5834.712) (-5828.354) -- 10:06:43      1588000 -- (-5843.308) (-5847.465) (-5849.841) [-5813.496] * (-5842.195) (-5858.021) (-5835.126) [-5826.176] -- 10:06:37      1589000 -- (-5837.686) (-5838.030) (-5860.226) [-5810.399] * (-5847.999) (-5873.332) (-5851.857) [-5829.114] -- 10:06:31      1590000 -- (-5837.238) (-5813.580) (-5849.816) [-5824.708] * (-5834.051) (-5866.250) (-5834.252) [-5830.810] -- 10:06:25      Average standard deviation of split frequencies: 0.010859      1591000 -- (-5859.862) [-5825.106] (-5842.541) (-5815.698) * (-5838.571) [-5831.578] (-5850.537) (-5820.873) -- 10:06:24      1592000 -- (-5830.619) (-5832.063) (-5870.339) [-5820.241] * [-5821.103] (-5857.551) (-5844.964) (-5839.385) -- 10:06:18      1593000 -- (-5849.340) (-5841.087) (-5852.712) [-5815.385] * (-5826.127) (-5842.128) (-5870.080) [-5826.627] -- 10:06:12      1594000 -- (-5841.033) (-5868.629) (-5839.818) [-5826.638] * (-5825.546) (-5861.916) (-5861.288) [-5826.436] -- 10:06:11      1595000 -- (-5838.076) (-5869.018) (-5851.184) [-5824.328] * (-5832.752) (-5845.167) (-5833.664) [-5827.714] -- 10:06:05      Average standard deviation of split frequencies: 0.010794      1596000 -- (-5831.028) (-5848.005) [-5825.075] (-5835.112) * (-5837.467) (-5852.518) [-5837.179] (-5843.901) -- 10:05:59      1597000 -- [-5831.247] (-5862.553) (-5835.642) (-5829.275) * (-5844.703) (-5855.842) [-5835.005] (-5851.769) -- 10:05:53      1598000 -- (-5835.225) (-5848.960) (-5840.841) [-5821.914] * (-5852.016) (-5858.793) [-5835.904] (-5840.442) -- 10:05:47      1599000 -- (-5817.943) (-5836.121) (-5836.124) [-5814.248] * (-5842.062) (-5861.220) [-5815.378] (-5843.876) -- 10:05:46      1600000 -- (-5826.158) (-5852.672) (-5861.834) [-5826.626] * [-5825.781] (-5866.760) (-5825.481) (-5838.310) -- 10:05:40      Average standard deviation of split frequencies: 0.010812      1601000 -- [-5822.134] (-5842.056) (-5844.990) (-5842.923) * (-5849.744) (-5857.661) (-5817.357) [-5823.454] -- 10:05:34      1602000 -- [-5815.737] (-5837.030) (-5845.281) (-5850.320) * (-5861.265) (-5855.827) [-5825.569] (-5823.976) -- 10:05:28      1603000 -- [-5834.135] (-5836.852) (-5850.927) (-5849.873) * (-5861.262) (-5841.762) (-5821.930) [-5831.728] -- 10:05:27      1604000 -- [-5831.432] (-5827.334) (-5869.638) (-5857.375) * (-5848.568) (-5850.717) (-5830.762) [-5835.055] -- 10:05:21      1605000 -- (-5825.127) [-5826.509] (-5862.262) (-5852.406) * (-5835.359) (-5854.766) (-5836.055) [-5822.289] -- 10:05:15      Average standard deviation of split frequencies: 0.010584      1606000 -- (-5827.612) [-5813.414] (-5853.679) (-5845.118) * [-5835.316] (-5849.332) (-5851.611) (-5829.260) -- 10:05:09      1607000 -- [-5824.661] (-5823.903) (-5856.158) (-5846.739) * (-5853.008) (-5864.721) [-5833.185] (-5835.323) -- 10:05:03      1608000 -- [-5825.827] (-5832.865) (-5835.129) (-5855.512) * [-5822.861] (-5854.444) (-5856.005) (-5837.687) -- 10:05:02      1609000 -- [-5832.522] (-5831.844) (-5843.339) (-5855.679) * [-5807.425] (-5835.010) (-5845.464) (-5873.608) -- 10:04:56      1610000 -- [-5824.994] (-5835.547) (-5846.759) (-5864.657) * [-5818.601] (-5847.865) (-5841.426) (-5876.114) -- 10:04:50      Average standard deviation of split frequencies: 0.010528      1611000 -- [-5830.443] (-5854.262) (-5841.920) (-5843.170) * [-5818.175] (-5852.995) (-5830.593) (-5865.934) -- 10:04:44      1612000 -- (-5839.974) (-5854.320) [-5839.551] (-5832.394) * [-5829.393] (-5889.533) (-5837.604) (-5837.136) -- 10:04:43      1613000 -- (-5839.322) (-5860.378) (-5835.277) [-5829.484] * (-5830.251) (-5873.253) [-5839.953] (-5846.258) -- 10:04:37      1614000 -- [-5826.696] (-5856.624) (-5837.518) (-5844.092) * (-5839.284) (-5854.980) [-5838.752] (-5846.900) -- 10:04:31      1615000 -- (-5828.388) (-5849.960) (-5848.216) [-5837.684] * (-5842.266) (-5872.981) (-5883.501) [-5839.329] -- 10:04:25      Average standard deviation of split frequencies: 0.010639      1616000 -- [-5816.705] (-5835.005) (-5837.074) (-5840.536) * (-5838.528) (-5871.833) (-5869.580) [-5820.180] -- 10:04:24      1617000 -- (-5832.360) (-5819.579) [-5826.004] (-5849.224) * (-5850.544) [-5836.619] (-5867.054) (-5818.353) -- 10:04:18      1618000 -- (-5837.531) [-5820.617] (-5830.238) (-5844.737) * (-5854.606) (-5854.426) (-5846.928) [-5833.375] -- 10:04:12      1619000 -- (-5834.553) (-5841.899) [-5822.209] (-5835.298) * (-5853.688) (-5863.225) (-5845.016) [-5817.132] -- 10:04:06      1620000 -- [-5827.017] (-5843.807) (-5827.997) (-5839.002) * (-5856.504) [-5842.317] (-5846.586) (-5837.539) -- 10:04:00      Average standard deviation of split frequencies: 0.010753      1621000 -- (-5852.661) (-5827.859) (-5819.712) [-5841.621] * (-5821.648) (-5843.021) (-5851.750) [-5834.110] -- 10:04:00      1622000 -- (-5862.596) [-5822.141] (-5833.794) (-5844.555) * (-5830.827) (-5845.559) (-5849.827) [-5831.032] -- 10:03:54      1623000 -- (-5830.692) [-5827.089] (-5835.241) (-5879.092) * (-5840.426) (-5834.534) (-5859.200) [-5822.632] -- 10:03:48      1624000 -- [-5835.286] (-5854.639) (-5837.476) (-5900.151) * (-5856.652) [-5818.311] (-5863.464) (-5820.704) -- 10:03:42      1625000 -- (-5828.826) [-5829.892] (-5844.405) (-5855.633) * (-5848.212) [-5819.333] (-5866.858) (-5821.854) -- 10:03:41      Average standard deviation of split frequencies: 0.010981      1626000 -- (-5843.674) [-5812.282] (-5832.170) (-5850.258) * (-5856.678) (-5824.228) [-5831.961] (-5834.254) -- 10:03:35      1627000 -- (-5848.624) [-5822.380] (-5844.653) (-5840.799) * (-5858.264) [-5838.312] (-5836.375) (-5838.946) -- 10:03:29      1628000 -- (-5846.355) [-5827.181] (-5856.796) (-5834.755) * [-5830.730] (-5826.461) (-5852.978) (-5847.969) -- 10:03:28      1629000 -- (-5827.389) [-5822.234] (-5846.751) (-5848.650) * (-5849.569) [-5840.952] (-5842.371) (-5855.913) -- 10:03:22      1630000 -- (-5842.379) (-5848.186) (-5866.139) [-5837.869] * (-5852.647) (-5825.204) [-5835.619] (-5865.156) -- 10:03:16      Average standard deviation of split frequencies: 0.011309      1631000 -- (-5826.085) [-5833.965] (-5856.307) (-5847.450) * (-5850.630) [-5819.429] (-5823.708) (-5866.123) -- 10:03:15      1632000 -- (-5856.854) (-5825.946) (-5873.744) [-5837.096] * (-5834.746) (-5827.355) [-5819.674] (-5859.312) -- 10:03:09      1633000 -- [-5841.958] (-5841.492) (-5855.482) (-5831.186) * (-5832.434) (-5848.463) [-5820.076] (-5839.808) -- 10:03:03      1634000 -- (-5835.096) (-5837.901) (-5849.575) [-5821.508] * (-5839.186) (-5838.659) [-5810.962] (-5861.302) -- 10:03:02      1635000 -- (-5851.145) (-5835.829) [-5828.304] (-5840.231) * (-5844.229) (-5833.553) [-5813.989] (-5874.975) -- 10:02:56      Average standard deviation of split frequencies: 0.011194      1636000 -- (-5846.512) (-5839.459) (-5840.188) [-5830.580] * (-5828.445) (-5830.550) [-5807.041] (-5860.150) -- 10:02:50      1637000 -- (-5851.674) (-5842.952) (-5865.033) [-5852.252] * (-5849.121) (-5825.844) [-5799.978] (-5857.329) -- 10:02:44      1638000 -- (-5878.218) [-5838.085] (-5832.858) (-5851.261) * (-5841.008) (-5852.548) [-5826.635] (-5846.585) -- 10:02:43      1639000 -- (-5872.969) (-5835.638) (-5833.741) [-5825.076] * (-5829.677) [-5828.931] (-5820.492) (-5840.901) -- 10:02:37      1640000 -- (-5848.616) [-5836.748] (-5848.240) (-5843.790) * [-5818.902] (-5828.131) (-5824.400) (-5848.466) -- 10:02:31      Average standard deviation of split frequencies: 0.011250      1641000 -- (-5854.096) [-5829.456] (-5861.553) (-5835.948) * (-5843.436) [-5827.258] (-5847.446) (-5853.431) -- 10:02:25      1642000 -- [-5836.820] (-5848.448) (-5837.803) (-5856.316) * (-5844.388) [-5825.142] (-5844.733) (-5838.613) -- 10:02:25      1643000 -- (-5826.617) (-5843.605) [-5826.117] (-5840.552) * (-5838.357) [-5806.732] (-5848.465) (-5842.130) -- 10:02:19      1644000 -- (-5842.035) (-5835.363) [-5827.572] (-5847.215) * (-5839.504) (-5829.524) (-5848.044) [-5827.441] -- 10:02:13      1645000 -- (-5832.291) [-5821.254] (-5843.972) (-5846.298) * [-5832.322] (-5844.013) (-5837.628) (-5854.975) -- 10:02:07      Average standard deviation of split frequencies: 0.011343      1646000 -- (-5846.880) (-5873.692) [-5817.202] (-5834.562) * (-5837.665) [-5831.202] (-5838.282) (-5850.188) -- 10:02:06      1647000 -- (-5868.680) (-5852.077) [-5836.243] (-5832.821) * (-5854.800) [-5841.564] (-5829.173) (-5855.226) -- 10:02:00      1648000 -- (-5842.931) (-5861.748) [-5821.399] (-5855.061) * (-5856.870) (-5824.450) (-5851.418) [-5839.923] -- 10:01:54      1649000 -- (-5869.432) (-5869.189) [-5819.615] (-5825.637) * (-5860.053) [-5833.742] (-5836.480) (-5823.916) -- 10:01:48      1650000 -- (-5861.001) (-5859.002) [-5832.084] (-5847.473) * (-5835.995) [-5819.982] (-5854.544) (-5845.841) -- 10:01:42      Average standard deviation of split frequencies: 0.011316      1651000 -- [-5837.912] (-5853.753) (-5829.236) (-5828.897) * (-5835.521) [-5838.070] (-5849.125) (-5851.290) -- 10:01:41      1652000 -- (-5865.508) (-5834.426) (-5838.953) [-5829.734] * [-5837.415] (-5856.341) (-5835.456) (-5837.729) -- 10:01:35      1653000 -- (-5877.111) [-5839.125] (-5851.794) (-5835.408) * (-5844.727) (-5833.451) [-5824.482] (-5851.202) -- 10:01:29      1654000 -- (-5844.875) (-5846.381) [-5823.902] (-5827.199) * (-5840.892) [-5813.050] (-5833.211) (-5845.697) -- 10:01:23      1655000 -- (-5867.027) (-5840.673) (-5840.560) [-5837.230] * (-5850.908) (-5817.302) (-5846.277) [-5820.155] -- 10:01:22      Average standard deviation of split frequencies: 0.011489      1656000 -- (-5852.814) (-5827.358) (-5827.787) [-5821.928] * (-5842.864) (-5814.093) (-5834.752) [-5831.853] -- 10:01:16      1657000 -- (-5832.304) [-5840.093] (-5830.415) (-5817.623) * (-5848.091) [-5823.649] (-5835.217) (-5838.031) -- 10:01:10      1658000 -- (-5822.963) (-5834.751) (-5850.506) [-5842.527] * (-5842.636) [-5826.272] (-5842.092) (-5830.881) -- 10:01:09      1659000 -- [-5832.764] (-5837.052) (-5855.237) (-5851.571) * (-5861.740) (-5835.474) [-5837.703] (-5847.461) -- 10:01:03      1660000 -- [-5824.857] (-5841.853) (-5847.373) (-5836.476) * (-5838.431) (-5848.554) (-5835.990) [-5840.067] -- 10:00:57      Average standard deviation of split frequencies: 0.011437      1661000 -- [-5826.445] (-5857.561) (-5835.010) (-5822.130) * (-5845.827) [-5825.295] (-5831.623) (-5828.913) -- 10:00:51      1662000 -- [-5832.127] (-5835.087) (-5856.378) (-5826.549) * (-5837.428) (-5847.051) [-5825.666] (-5857.552) -- 10:00:51      1663000 -- (-5817.288) (-5851.707) (-5856.032) [-5830.018] * [-5825.708] (-5830.310) (-5828.632) (-5871.636) -- 10:00:45      1664000 -- (-5819.124) (-5858.038) (-5852.521) [-5813.230] * (-5828.023) (-5821.346) [-5825.335] (-5863.078) -- 10:00:39      1665000 -- [-5821.636] (-5843.728) (-5845.419) (-5853.142) * (-5824.316) (-5836.927) [-5809.144] (-5858.475) -- 10:00:33      Average standard deviation of split frequencies: 0.011370      1666000 -- [-5809.770] (-5845.307) (-5829.023) (-5839.614) * [-5821.587] (-5841.454) (-5823.286) (-5857.559) -- 10:00:27      1667000 -- [-5811.491] (-5842.167) (-5825.650) (-5838.585) * (-5837.155) (-5847.026) [-5829.790] (-5849.783) -- 10:00:26      1668000 -- (-5818.184) (-5845.250) [-5827.430] (-5864.812) * [-5830.868] (-5848.216) (-5841.306) (-5835.810) -- 10:00:20      1669000 -- [-5816.157] (-5847.860) (-5839.219) (-5839.932) * [-5832.514] (-5832.043) (-5837.860) (-5854.388) -- 10:00:14      1670000 -- (-5826.912) (-5853.128) (-5852.798) [-5825.754] * [-5823.945] (-5826.292) (-5855.833) (-5872.665) -- 10:00:13      Average standard deviation of split frequencies: 0.011598      1671000 -- (-5839.611) (-5877.161) (-5837.299) [-5811.677] * [-5827.699] (-5838.009) (-5837.195) (-5854.831) -- 10:00:07      1672000 -- (-5840.777) (-5857.797) (-5842.572) [-5823.387] * [-5823.347] (-5837.168) (-5831.894) (-5853.862) -- 10:00:01      1673000 -- (-5843.711) (-5834.976) (-5837.864) [-5828.919] * [-5807.684] (-5851.012) (-5844.153) (-5839.514) -- 10:00:00      1674000 -- (-5834.299) [-5832.718] (-5856.780) (-5852.186) * [-5821.265] (-5812.463) (-5858.470) (-5824.988) -- 9:59:54      1675000 -- (-5820.874) [-5837.991] (-5846.825) (-5847.029) * (-5824.599) [-5822.951] (-5853.501) (-5822.541) -- 9:59:48      Average standard deviation of split frequencies: 0.011494      1676000 -- (-5847.905) [-5811.820] (-5847.951) (-5837.308) * [-5829.153] (-5826.562) (-5862.724) (-5841.564) -- 9:59:47      1677000 -- (-5833.555) (-5818.283) (-5856.697) [-5817.684] * (-5825.934) (-5855.607) [-5836.727] (-5853.087) -- 9:59:41      1678000 -- [-5823.724] (-5833.041) (-5859.877) (-5821.283) * [-5830.773] (-5837.819) (-5848.585) (-5864.135) -- 9:59:36      1679000 -- [-5826.024] (-5831.604) (-5863.046) (-5833.524) * [-5831.112] (-5831.282) (-5869.921) (-5847.556) -- 9:59:35      1680000 -- [-5824.894] (-5842.984) (-5870.157) (-5844.710) * (-5851.652) [-5828.303] (-5860.571) (-5841.751) -- 9:59:29      Average standard deviation of split frequencies: 0.011559      1681000 -- (-5848.085) [-5831.901] (-5855.382) (-5828.603) * (-5831.635) (-5858.665) (-5855.114) [-5813.464] -- 9:59:23      1682000 -- (-5868.643) [-5834.286] (-5845.086) (-5842.570) * (-5835.194) (-5851.152) (-5840.331) [-5814.683] -- 9:59:22      1683000 -- (-5843.885) [-5819.546] (-5849.126) (-5835.204) * (-5863.277) (-5878.605) [-5826.943] (-5835.272) -- 9:59:16      1684000 -- [-5821.307] (-5838.428) (-5847.871) (-5828.280) * [-5841.837] (-5836.223) (-5836.210) (-5844.941) -- 9:59:10      1685000 -- [-5826.143] (-5833.467) (-5852.805) (-5844.589) * (-5849.620) (-5822.924) (-5846.433) [-5822.224] -- 9:59:04      Average standard deviation of split frequencies: 0.011663      1686000 -- [-5830.307] (-5845.080) (-5849.365) (-5839.862) * (-5854.018) [-5813.665] (-5854.461) (-5847.814) -- 9:59:03      1687000 -- (-5855.435) (-5832.243) (-5825.483) [-5821.929] * (-5860.297) [-5810.743] (-5853.872) (-5836.691) -- 9:58:57      1688000 -- (-5854.395) [-5837.576] (-5840.739) (-5827.093) * (-5847.954) [-5830.356] (-5841.015) (-5842.360) -- 9:58:51      1689000 -- (-5858.867) [-5841.003] (-5834.222) (-5823.282) * (-5843.389) (-5830.339) (-5844.604) [-5828.639] -- 9:58:50      1690000 -- (-5881.634) (-5846.660) (-5825.632) [-5813.721] * (-5824.794) [-5817.186] (-5835.443) (-5837.017) -- 9:58:44      Average standard deviation of split frequencies: 0.011536      1691000 -- (-5873.749) (-5847.282) [-5828.856] (-5824.862) * (-5845.592) [-5823.902] (-5834.348) (-5835.029) -- 9:58:38      1692000 -- (-5857.901) (-5829.059) [-5831.915] (-5824.299) * (-5849.920) (-5850.095) [-5841.712] (-5840.477) -- 9:58:37      1693000 -- (-5866.621) (-5846.714) (-5824.290) [-5822.111] * (-5853.526) (-5852.323) (-5834.552) [-5842.086] -- 9:58:31      1694000 -- (-5884.436) (-5861.129) (-5831.970) [-5822.901] * (-5875.169) (-5835.306) [-5831.067] (-5830.264) -- 9:58:26      1695000 -- (-5838.667) (-5872.628) (-5836.273) [-5824.605] * (-5860.704) [-5832.277] (-5863.494) (-5827.784) -- 9:58:25      Average standard deviation of split frequencies: 0.011544      1696000 -- (-5820.364) (-5850.379) (-5847.102) [-5816.364] * (-5853.688) (-5822.415) (-5856.089) [-5831.632] -- 9:58:19      1697000 -- (-5845.478) (-5849.084) (-5853.352) [-5811.606] * (-5868.762) (-5830.929) (-5833.594) [-5825.001] -- 9:58:13      1698000 -- (-5851.449) (-5842.856) (-5828.305) [-5814.393] * (-5869.533) (-5847.874) [-5827.109] (-5831.935) -- 9:58:12      1699000 -- (-5855.462) (-5837.145) (-5821.984) [-5811.980] * (-5859.248) (-5814.869) [-5826.622] (-5820.518) -- 9:58:06      1700000 -- (-5851.765) (-5829.346) (-5832.787) [-5825.220] * (-5866.475) (-5817.330) (-5834.678) [-5832.099] -- 9:58:00      Average standard deviation of split frequencies: 0.011528      1701000 -- (-5847.504) (-5854.808) (-5817.709) [-5814.988] * (-5865.697) (-5832.795) (-5838.274) [-5819.384] -- 9:57:54      1702000 -- (-5858.877) (-5834.866) [-5804.649] (-5816.994) * (-5867.621) (-5844.722) (-5838.847) [-5817.522] -- 9:57:53      1703000 -- (-5867.898) [-5820.128] (-5814.791) (-5842.429) * (-5863.074) (-5828.182) (-5845.392) [-5820.544] -- 9:57:47      1704000 -- (-5853.788) [-5830.495] (-5820.456) (-5829.373) * (-5824.333) (-5829.218) (-5850.276) [-5821.055] -- 9:57:41      1705000 -- (-5851.589) (-5846.181) [-5818.086] (-5836.312) * (-5833.043) (-5845.711) (-5862.290) [-5826.119] -- 9:57:35      Average standard deviation of split frequencies: 0.011681      1706000 -- (-5859.382) (-5823.013) [-5838.817] (-5833.149) * [-5823.860] (-5852.729) (-5862.059) (-5845.112) -- 9:57:34      1707000 -- (-5836.327) (-5837.685) [-5843.480] (-5831.533) * [-5825.061] (-5849.009) (-5860.408) (-5835.442) -- 9:57:28      1708000 -- (-5846.524) (-5836.313) (-5858.490) [-5831.100] * [-5814.687] (-5849.120) (-5847.504) (-5841.684) -- 9:57:22      1709000 -- [-5816.902] (-5840.931) (-5843.387) (-5843.563) * [-5825.434] (-5842.337) (-5881.278) (-5845.349) -- 9:57:17      1710000 -- [-5833.101] (-5839.159) (-5857.312) (-5831.438) * (-5833.746) [-5837.877] (-5854.643) (-5824.054) -- 9:57:11      Average standard deviation of split frequencies: 0.011545      1711000 -- (-5834.955) (-5862.583) (-5859.084) [-5826.068] * (-5845.280) (-5842.353) (-5868.674) [-5826.467] -- 9:57:10      1712000 -- [-5821.972] (-5841.265) (-5842.130) (-5823.658) * (-5847.862) (-5833.257) [-5838.822] (-5837.032) -- 9:57:04      1713000 -- [-5817.250] (-5840.420) (-5843.464) (-5824.490) * [-5831.388] (-5839.369) (-5848.617) (-5826.286) -- 9:56:58      1714000 -- [-5827.600] (-5823.962) (-5837.380) (-5843.537) * [-5842.383] (-5842.219) (-5831.421) (-5831.577) -- 9:56:57      1715000 -- (-5827.912) [-5816.601] (-5845.157) (-5856.579) * (-5840.931) [-5835.552] (-5840.571) (-5852.706) -- 9:56:51      Average standard deviation of split frequencies: 0.011543      1716000 -- [-5823.612] (-5824.365) (-5850.457) (-5833.812) * (-5842.460) (-5837.155) [-5837.377] (-5852.134) -- 9:56:45      1717000 -- (-5841.500) (-5822.169) (-5831.499) [-5832.089] * (-5848.371) (-5849.937) [-5827.331] (-5851.284) -- 9:56:44      1718000 -- [-5815.262] (-5830.423) (-5855.737) (-5823.604) * (-5863.624) [-5828.462] (-5837.645) (-5839.559) -- 9:56:38      1719000 -- (-5815.473) [-5833.762] (-5875.627) (-5839.415) * (-5853.489) (-5838.501) [-5835.753] (-5837.037) -- 9:56:32      1720000 -- [-5819.638] (-5821.223) (-5873.129) (-5826.723) * (-5860.656) (-5861.351) [-5813.964] (-5822.368) -- 9:56:26      Average standard deviation of split frequencies: 0.011408      1721000 -- [-5821.276] (-5828.259) (-5861.350) (-5842.935) * (-5859.934) (-5869.570) [-5825.075] (-5816.229) -- 9:56:25      1722000 -- (-5825.581) [-5817.019] (-5855.052) (-5874.924) * (-5856.082) (-5865.142) (-5818.165) [-5815.480] -- 9:56:19      1723000 -- (-5837.006) [-5828.188] (-5830.336) (-5862.353) * (-5843.334) (-5844.283) (-5834.918) [-5817.975] -- 9:56:14      1724000 -- (-5844.908) (-5831.755) [-5821.859] (-5861.773) * (-5854.975) (-5854.162) [-5832.502] (-5828.630) -- 9:56:13      1725000 -- (-5866.176) [-5817.356] (-5834.459) (-5839.351) * (-5871.599) (-5851.797) (-5835.284) [-5838.345] -- 9:56:07      Average standard deviation of split frequencies: 0.011393      1726000 -- (-5849.956) [-5818.360] (-5855.446) (-5845.285) * (-5839.657) (-5858.631) [-5814.422] (-5835.272) -- 9:56:01      1727000 -- (-5855.731) (-5826.410) [-5833.807] (-5829.508) * (-5840.352) (-5838.994) [-5820.031] (-5844.012) -- 9:55:55      1728000 -- (-5849.886) (-5828.356) (-5842.629) [-5826.585] * [-5830.851] (-5845.504) (-5814.765) (-5852.392) -- 9:55:54      1729000 -- (-5853.652) (-5826.785) (-5837.848) [-5804.800] * (-5841.241) (-5847.190) (-5828.663) [-5836.206] -- 9:55:48      1730000 -- (-5864.477) (-5820.761) (-5842.595) [-5801.645] * (-5836.055) (-5842.785) [-5830.876] (-5833.976) -- 9:55:42      Average standard deviation of split frequencies: 0.011314      1731000 -- (-5840.162) (-5813.145) (-5849.555) [-5810.571] * (-5870.766) [-5827.724] (-5840.058) (-5825.471) -- 9:55:41      1732000 -- (-5876.766) (-5815.792) (-5839.363) [-5812.538] * (-5850.313) (-5845.960) [-5826.081] (-5845.161) -- 9:55:35      1733000 -- (-5853.638) [-5822.211] (-5856.801) (-5830.238) * (-5859.878) (-5837.761) (-5843.641) [-5816.032] -- 9:55:29      1734000 -- (-5853.793) [-5816.490] (-5856.096) (-5836.821) * (-5848.739) (-5839.955) (-5837.147) [-5828.889] -- 9:55:23      1735000 -- (-5867.507) [-5814.656] (-5836.945) (-5849.768) * (-5841.359) [-5821.284] (-5869.583) (-5831.333) -- 9:55:22      Average standard deviation of split frequencies: 0.011361      1736000 -- (-5875.038) [-5817.580] (-5837.378) (-5846.601) * [-5823.224] (-5829.520) (-5854.245) (-5848.760) -- 9:55:17      1737000 -- (-5858.196) (-5831.180) [-5834.923] (-5862.964) * [-5834.124] (-5825.674) (-5865.336) (-5821.992) -- 9:55:11      1738000 -- (-5854.474) [-5818.091] (-5840.171) (-5864.815) * (-5832.939) [-5828.759] (-5829.398) (-5838.411) -- 9:55:05      1739000 -- (-5847.450) [-5806.691] (-5841.423) (-5857.161) * (-5830.693) [-5828.693] (-5848.948) (-5845.483) -- 9:54:59      1740000 -- (-5829.477) [-5827.595] (-5867.216) (-5839.723) * (-5837.423) [-5826.383] (-5849.256) (-5853.811) -- 9:54:58      Average standard deviation of split frequencies: 0.011518      1741000 -- (-5828.701) (-5832.708) (-5831.335) [-5840.468] * (-5853.883) [-5836.098] (-5842.972) (-5858.886) -- 9:54:52      1742000 -- (-5848.043) [-5826.316] (-5843.036) (-5834.077) * (-5869.913) (-5823.617) [-5830.599] (-5834.877) -- 9:54:46      1743000 -- [-5836.603] (-5827.810) (-5835.789) (-5846.380) * (-5854.354) (-5830.321) [-5833.193] (-5831.290) -- 9:54:45      1744000 -- (-5849.821) (-5834.160) [-5843.301] (-5825.902) * (-5853.870) (-5845.210) [-5830.438] (-5832.690) -- 9:54:39      1745000 -- [-5825.481] (-5832.241) (-5856.090) (-5836.707) * (-5854.215) (-5830.931) [-5813.755] (-5865.300) -- 9:54:33      Average standard deviation of split frequencies: 0.011718      1746000 -- [-5830.821] (-5835.321) (-5854.202) (-5868.373) * (-5848.066) (-5854.185) [-5829.435] (-5869.189) -- 9:54:28      1747000 -- (-5833.573) [-5836.006] (-5853.620) (-5850.563) * (-5860.585) (-5855.420) [-5828.082] (-5870.157) -- 9:54:26      1748000 -- (-5841.678) (-5833.695) (-5853.407) [-5837.777] * [-5838.939] (-5852.295) (-5825.162) (-5864.221) -- 9:54:21      1749000 -- (-5832.831) (-5843.976) (-5838.193) [-5825.838] * (-5847.504) (-5847.669) [-5830.847] (-5855.888) -- 9:54:15      1750000 -- (-5823.322) (-5857.231) (-5859.971) [-5832.880] * (-5856.087) (-5846.898) (-5837.782) [-5822.959] -- 9:54:09      Average standard deviation of split frequencies: 0.011770      1751000 -- (-5832.517) (-5851.608) (-5857.711) [-5811.808] * (-5857.701) (-5838.859) (-5838.464) [-5813.826] -- 9:54:08      1752000 -- (-5823.238) (-5850.420) (-5872.911) [-5819.577] * (-5860.499) (-5851.242) (-5839.140) [-5824.515] -- 9:54:02      1753000 -- (-5842.865) (-5836.261) (-5849.332) [-5817.336] * (-5846.444) (-5844.862) (-5835.411) [-5816.283] -- 9:53:56      1754000 -- (-5833.902) (-5851.832) (-5853.872) [-5826.785] * (-5832.941) (-5862.655) (-5838.185) [-5814.065] -- 9:53:50      1755000 -- (-5852.685) (-5850.396) (-5868.535) [-5814.838] * (-5847.823) (-5862.097) (-5834.692) [-5822.652] -- 9:53:49      Average standard deviation of split frequencies: 0.012022      1756000 -- (-5853.622) (-5843.156) (-5835.997) [-5809.088] * (-5836.755) (-5873.570) (-5842.237) [-5823.589] -- 9:53:43      1757000 -- (-5850.052) (-5843.955) (-5832.326) [-5837.701] * (-5837.771) (-5861.969) (-5851.241) [-5825.160] -- 9:53:38      1758000 -- (-5845.605) (-5836.702) [-5838.499] (-5846.824) * [-5836.084] (-5871.777) (-5845.595) (-5819.727) -- 9:53:32      1759000 -- (-5846.637) (-5837.051) [-5848.407] (-5851.496) * (-5830.750) (-5852.583) (-5843.745) [-5817.326] -- 9:53:31      1760000 -- (-5843.726) [-5822.363] (-5844.102) (-5853.333) * (-5845.341) (-5862.295) (-5827.021) [-5817.445] -- 9:53:25      Average standard deviation of split frequencies: 0.012170      1761000 -- (-5840.530) [-5827.760] (-5849.334) (-5867.018) * [-5825.257] (-5867.888) (-5844.092) (-5824.153) -- 9:53:19      1762000 -- (-5828.292) (-5843.466) (-5839.994) [-5844.513] * (-5840.547) (-5869.772) [-5807.981] (-5831.623) -- 9:53:18      1763000 -- [-5828.397] (-5840.431) (-5857.331) (-5851.843) * (-5819.577) (-5864.776) (-5823.730) [-5820.918] -- 9:53:12      1764000 -- [-5824.886] (-5847.850) (-5841.977) (-5876.077) * (-5833.527) (-5884.680) (-5830.859) [-5825.476] -- 9:53:06      1765000 -- (-5827.631) (-5841.510) (-5840.216) [-5843.569] * [-5830.172] (-5842.569) (-5854.500) (-5833.262) -- 9:53:05      Average standard deviation of split frequencies: 0.012400      1766000 -- (-5823.916) (-5851.767) [-5837.322] (-5856.885) * (-5846.152) (-5843.962) (-5850.031) [-5819.432] -- 9:52:59      1767000 -- [-5817.732] (-5839.363) (-5835.480) (-5862.946) * (-5858.126) (-5835.471) (-5858.176) [-5827.965] -- 9:52:53      1768000 -- [-5811.239] (-5859.873) (-5815.810) (-5869.055) * (-5848.843) (-5829.768) [-5831.967] (-5842.160) -- 9:52:48      1769000 -- [-5812.685] (-5850.283) (-5825.637) (-5864.957) * (-5839.808) (-5853.762) [-5829.999] (-5835.524) -- 9:52:46      1770000 -- [-5814.738] (-5875.317) (-5828.016) (-5868.954) * (-5851.360) [-5818.556] (-5850.742) (-5833.154) -- 9:52:41      Average standard deviation of split frequencies: 0.012363      1771000 -- [-5803.670] (-5861.187) (-5827.376) (-5852.725) * (-5861.790) (-5821.976) (-5845.817) [-5830.836] -- 9:52:35      1772000 -- [-5824.427] (-5858.031) (-5824.856) (-5840.413) * (-5882.094) (-5828.137) (-5840.760) [-5827.594] -- 9:52:29      1773000 -- (-5843.616) (-5866.499) [-5804.549] (-5828.193) * (-5846.506) (-5821.135) (-5838.301) [-5820.434] -- 9:52:23      1774000 -- (-5845.036) (-5847.346) [-5818.646] (-5818.678) * (-5849.095) (-5823.629) (-5826.524) [-5830.366] -- 9:52:22      1775000 -- (-5837.095) (-5846.614) (-5824.816) [-5821.383] * (-5830.386) (-5826.649) (-5829.277) [-5821.297] -- 9:52:16      Average standard deviation of split frequencies: 0.012253      1776000 -- (-5832.751) (-5837.049) (-5856.698) [-5818.666] * (-5825.518) (-5839.380) (-5853.107) [-5835.169] -- 9:52:10      1777000 -- (-5843.714) (-5851.698) (-5861.474) [-5815.857] * [-5834.548] (-5828.421) (-5851.690) (-5840.049) -- 9:52:09      1778000 -- [-5821.540] (-5839.902) (-5847.459) (-5811.209) * (-5833.635) (-5842.512) [-5828.273] (-5878.732) -- 9:52:03      1779000 -- (-5837.953) (-5837.725) (-5848.923) [-5809.204] * [-5830.766] (-5822.003) (-5831.477) (-5848.078) -- 9:51:58      1780000 -- (-5856.395) (-5841.833) (-5839.833) [-5809.842] * [-5823.161] (-5846.621) (-5854.620) (-5859.392) -- 9:51:52      Average standard deviation of split frequencies: 0.012116      1781000 -- (-5849.264) [-5846.876] (-5840.635) (-5826.326) * [-5817.262] (-5838.036) (-5846.641) (-5857.462) -- 9:51:46      1782000 -- (-5849.367) (-5821.817) [-5849.988] (-5836.989) * (-5823.866) [-5816.780] (-5843.421) (-5862.650) -- 9:51:45      1783000 -- (-5853.318) (-5846.243) (-5835.340) [-5836.436] * (-5834.910) [-5818.761] (-5832.892) (-5855.248) -- 9:51:39      1784000 -- (-5850.339) [-5822.510] (-5841.253) (-5820.378) * [-5832.378] (-5830.855) (-5826.141) (-5853.907) -- 9:51:33      1785000 -- (-5819.929) (-5826.158) (-5855.757) [-5826.879] * [-5841.088] (-5834.477) (-5828.091) (-5869.951) -- 9:51:27      Average standard deviation of split frequencies: 0.011969      1786000 -- [-5817.783] (-5825.002) (-5880.089) (-5823.018) * (-5852.385) (-5824.640) [-5839.786] (-5850.017) -- 9:51:26      1787000 -- [-5819.503] (-5829.913) (-5870.001) (-5835.681) * (-5861.527) [-5823.290] (-5841.704) (-5866.121) -- 9:51:20      1788000 -- (-5835.501) [-5829.510] (-5857.438) (-5830.382) * [-5846.944] (-5833.754) (-5851.336) (-5865.129) -- 9:51:15      1789000 -- (-5848.080) [-5816.448] (-5888.323) (-5835.220) * (-5834.660) [-5822.014] (-5851.836) (-5836.385) -- 9:51:13      1790000 -- (-5864.408) [-5816.348] (-5864.724) (-5857.739) * (-5831.582) [-5819.971] (-5842.101) (-5867.822) -- 9:51:08      Average standard deviation of split frequencies: 0.011809      1791000 -- [-5827.160] (-5813.980) (-5849.541) (-5847.637) * (-5843.484) [-5833.423] (-5877.979) (-5842.414) -- 9:51:02      1792000 -- (-5830.991) [-5809.520] (-5839.675) (-5846.912) * (-5840.753) [-5821.022] (-5880.351) (-5837.448) -- 9:51:01      1793000 -- (-5855.535) [-5814.814] (-5875.812) (-5839.902) * (-5841.015) [-5812.980] (-5876.931) (-5828.694) -- 9:50:55      1794000 -- (-5822.339) [-5826.406] (-5878.366) (-5847.665) * (-5849.259) [-5829.385] (-5853.523) (-5828.792) -- 9:50:49      1795000 -- [-5839.511] (-5830.871) (-5862.242) (-5845.924) * (-5865.559) (-5838.176) (-5859.760) [-5818.552] -- 9:50:48      Average standard deviation of split frequencies: 0.011811      1796000 -- (-5831.252) [-5823.605] (-5876.695) (-5849.758) * (-5846.535) (-5844.872) (-5856.833) [-5815.592] -- 9:50:42      1797000 -- (-5837.510) [-5814.516] (-5860.327) (-5818.327) * (-5822.433) (-5855.899) (-5842.894) [-5826.511] -- 9:50:36      1798000 -- (-5845.228) [-5828.652] (-5852.783) (-5843.126) * [-5827.621] (-5859.722) (-5852.623) (-5821.183) -- 9:50:35      1799000 -- (-5845.710) [-5819.925] (-5846.251) (-5837.782) * [-5814.310] (-5846.576) (-5856.417) (-5842.255) -- 9:50:29      1800000 -- (-5856.139) (-5814.451) (-5861.091) [-5819.350] * [-5825.711] (-5847.849) (-5854.097) (-5828.313) -- 9:50:24      Average standard deviation of split frequencies: 0.011914      1801000 -- (-5839.495) [-5814.266] (-5876.213) (-5842.815) * (-5844.567) (-5837.524) (-5839.762) [-5827.653] -- 9:50:18      1802000 -- (-5846.964) [-5811.518] (-5852.304) (-5834.553) * (-5838.881) [-5826.374] (-5852.481) (-5832.718) -- 9:50:16      1803000 -- (-5843.727) [-5816.230] (-5845.917) (-5831.162) * (-5833.109) (-5821.010) (-5822.287) [-5823.632] -- 9:50:11      1804000 -- [-5824.274] (-5859.735) (-5845.851) (-5833.160) * [-5833.473] (-5844.164) (-5829.004) (-5823.663) -- 9:50:05      1805000 -- (-5865.397) (-5849.724) (-5852.280) [-5839.009] * (-5835.857) (-5861.790) (-5841.580) [-5821.128] -- 9:50:04      Average standard deviation of split frequencies: 0.011908      1806000 -- (-5867.157) (-5832.775) (-5843.886) [-5828.396] * (-5850.522) (-5832.230) [-5821.545] (-5835.612) -- 9:49:58      1807000 -- (-5833.989) (-5845.070) [-5831.504] (-5823.187) * (-5834.797) [-5830.008] (-5834.561) (-5836.285) -- 9:49:52      1808000 -- (-5824.598) [-5838.800] (-5837.612) (-5842.250) * (-5843.698) [-5834.743] (-5828.459) (-5854.008) -- 9:49:46      1809000 -- (-5844.654) (-5820.827) [-5840.990] (-5845.578) * (-5852.939) [-5834.554] (-5848.246) (-5852.654) -- 9:49:45      1810000 -- (-5847.213) (-5851.222) (-5830.222) [-5845.915] * (-5835.908) [-5836.235] (-5837.196) (-5862.863) -- 9:49:39      Average standard deviation of split frequencies: 0.011883      1811000 -- [-5844.405] (-5861.434) (-5838.936) (-5837.206) * (-5834.830) [-5826.362] (-5826.619) (-5857.970) -- 9:49:38      1812000 -- (-5861.294) (-5860.714) [-5815.662] (-5835.919) * (-5850.415) [-5834.770] (-5828.852) (-5849.865) -- 9:49:32      1813000 -- (-5872.172) (-5857.898) [-5828.590] (-5832.413) * (-5839.243) [-5837.114] (-5831.186) (-5855.387) -- 9:49:27      1814000 -- (-5856.536) (-5839.241) [-5811.008] (-5849.974) * (-5845.626) [-5832.348] (-5822.444) (-5864.256) -- 9:49:21      1815000 -- (-5849.935) (-5848.516) [-5828.576] (-5847.256) * (-5833.606) (-5828.508) [-5817.742] (-5841.881) -- 9:49:15      Average standard deviation of split frequencies: 0.011384      1816000 -- [-5835.096] (-5834.455) (-5823.137) (-5862.906) * (-5839.288) (-5841.291) [-5825.056] (-5834.780) -- 9:49:14      1817000 -- (-5832.658) [-5823.301] (-5826.433) (-5863.967) * (-5841.531) (-5819.821) [-5834.967] (-5834.307) -- 9:49:08      1818000 -- (-5822.574) (-5841.746) [-5821.809] (-5853.468) * (-5874.710) (-5827.040) [-5827.379] (-5850.290) -- 9:49:02      1819000 -- (-5835.462) [-5828.072] (-5820.402) (-5860.930) * (-5850.228) (-5854.858) [-5832.056] (-5846.820) -- 9:48:57      1820000 -- [-5826.190] (-5837.274) (-5828.273) (-5858.074) * (-5849.564) (-5849.496) [-5828.274] (-5868.692) -- 9:48:51      Average standard deviation of split frequencies: 0.011355      1821000 -- [-5818.918] (-5824.780) (-5826.522) (-5838.829) * (-5864.501) (-5841.677) [-5826.004] (-5851.104) -- 9:48:50      1822000 -- [-5815.856] (-5833.986) (-5826.381) (-5844.712) * (-5845.249) (-5844.460) [-5819.136] (-5839.735) -- 9:48:44      1823000 -- (-5838.407) [-5832.847] (-5847.478) (-5854.500) * [-5819.696] (-5841.808) (-5837.802) (-5856.495) -- 9:48:38      1824000 -- (-5828.881) (-5848.942) [-5823.757] (-5853.400) * [-5815.905] (-5840.264) (-5867.190) (-5838.211) -- 9:48:32      1825000 -- (-5845.210) (-5835.747) [-5821.524] (-5856.118) * [-5812.083] (-5832.087) (-5840.408) (-5851.257) -- 9:48:31      Average standard deviation of split frequencies: 0.011200      1826000 -- (-5868.335) [-5844.158] (-5826.632) (-5844.617) * (-5823.343) (-5840.788) [-5824.460] (-5854.323) -- 9:48:25      1827000 -- (-5845.557) (-5856.435) [-5825.458] (-5853.623) * (-5839.650) (-5832.548) [-5822.162] (-5856.008) -- 9:48:20      1828000 -- (-5856.128) (-5845.200) [-5832.035] (-5842.894) * [-5831.541] (-5840.821) (-5830.751) (-5859.905) -- 9:48:14      1829000 -- [-5836.758] (-5860.986) (-5836.752) (-5838.953) * (-5827.479) (-5830.348) [-5829.163] (-5859.837) -- 9:48:08      1830000 -- (-5838.400) (-5834.258) [-5834.046] (-5847.587) * [-5829.991] (-5825.675) (-5843.117) (-5851.636) -- 9:48:02      Average standard deviation of split frequencies: 0.011139      1831000 -- (-5830.241) [-5849.130] (-5839.718) (-5860.675) * (-5835.779) (-5828.427) [-5830.192] (-5842.416) -- 9:48:01      1832000 -- (-5828.508) (-5835.279) [-5826.880] (-5852.239) * [-5831.367] (-5843.862) (-5829.077) (-5837.134) -- 9:47:55      1833000 -- [-5822.278] (-5841.748) (-5829.472) (-5855.373) * (-5855.635) [-5842.977] (-5822.278) (-5845.775) -- 9:47:50      1834000 -- (-5842.100) (-5870.605) (-5834.066) [-5829.278] * (-5846.977) (-5854.717) [-5818.922] (-5849.742) -- 9:47:44      1835000 -- [-5835.205] (-5855.098) (-5863.712) (-5857.122) * (-5844.166) (-5831.915) [-5819.761] (-5841.462) -- 9:47:38      Average standard deviation of split frequencies: 0.011171      1836000 -- [-5809.470] (-5844.970) (-5834.927) (-5857.836) * (-5851.794) (-5833.872) [-5811.390] (-5836.812) -- 9:47:37      1837000 -- [-5811.589] (-5841.188) (-5818.890) (-5850.613) * (-5846.029) [-5834.636] (-5839.232) (-5852.744) -- 9:47:31      1838000 -- (-5808.896) (-5836.556) [-5815.115] (-5858.135) * (-5850.192) [-5820.840] (-5843.918) (-5838.561) -- 9:47:25      1839000 -- (-5817.940) [-5819.317] (-5828.735) (-5861.683) * (-5861.494) (-5838.094) (-5858.831) [-5855.044] -- 9:47:20      1840000 -- [-5831.485] (-5834.992) (-5821.595) (-5862.647) * [-5846.114] (-5833.936) (-5861.476) (-5846.409) -- 9:47:14      Average standard deviation of split frequencies: 0.011148      1841000 -- (-5826.194) (-5839.797) [-5825.299] (-5854.351) * (-5827.793) [-5832.990] (-5875.573) (-5845.597) -- 9:47:13      1842000 -- [-5816.787] (-5845.614) (-5825.573) (-5859.274) * (-5833.012) [-5825.393] (-5872.021) (-5831.989) -- 9:47:07      1843000 -- (-5813.627) (-5884.221) [-5820.729] (-5876.668) * (-5829.058) (-5828.300) (-5866.137) [-5826.763] -- 9:47:01      1844000 -- [-5822.991] (-5846.330) (-5837.305) (-5865.449) * [-5828.269] (-5839.673) (-5867.778) (-5838.353) -- 9:46:55      1845000 -- (-5835.988) (-5836.968) [-5816.297] (-5866.545) * [-5812.003] (-5833.825) (-5869.917) (-5835.717) -- 9:46:54      Average standard deviation of split frequencies: 0.011181      1846000 -- (-5824.660) (-5827.223) [-5814.363] (-5846.750) * (-5847.049) (-5838.080) [-5838.265] (-5840.050) -- 9:46:48      1847000 -- (-5839.850) (-5837.106) [-5805.848] (-5845.231) * (-5848.634) [-5841.731] (-5854.879) (-5866.907) -- 9:46:43      1848000 -- (-5827.075) [-5822.402] (-5822.265) (-5855.142) * (-5840.080) [-5839.767] (-5859.284) (-5860.464) -- 9:46:37      1849000 -- [-5829.167] (-5831.681) (-5839.384) (-5848.463) * [-5832.573] (-5843.954) (-5843.247) (-5864.330) -- 9:46:36      1850000 -- (-5835.993) [-5828.314] (-5860.844) (-5846.398) * [-5822.376] (-5827.273) (-5853.942) (-5837.487) -- 9:46:30      Average standard deviation of split frequencies: 0.011297      1851000 -- [-5811.261] (-5825.315) (-5842.709) (-5849.765) * (-5842.908) [-5828.303] (-5871.997) (-5853.109) -- 9:46:24      1852000 -- [-5823.049] (-5833.118) (-5847.031) (-5836.340) * (-5829.944) (-5847.348) (-5844.290) [-5843.958] -- 9:46:18      1853000 -- [-5819.125] (-5844.665) (-5827.507) (-5851.560) * (-5841.941) [-5839.187] (-5851.437) (-5828.760) -- 9:46:13      1854000 -- [-5824.101] (-5838.254) (-5858.044) (-5817.886) * [-5831.057] (-5829.949) (-5841.016) (-5847.540) -- 9:46:07      1855000 -- [-5827.415] (-5857.189) (-5872.115) (-5824.748) * (-5831.060) [-5821.768] (-5839.261) (-5853.712) -- 9:46:06      Average standard deviation of split frequencies: 0.011263      1856000 -- (-5856.171) (-5855.234) (-5863.704) [-5811.694] * (-5837.383) [-5820.695] (-5849.010) (-5849.815) -- 9:46:00      1857000 -- (-5833.473) (-5868.423) (-5844.118) [-5826.103] * (-5843.751) [-5823.862] (-5850.722) (-5858.250) -- 9:45:54      1858000 -- [-5823.177] (-5847.725) (-5830.950) (-5838.903) * (-5850.428) [-5830.417] (-5848.037) (-5864.963) -- 9:45:49      1859000 -- (-5823.672) [-5834.000] (-5818.367) (-5856.672) * [-5838.263] (-5836.568) (-5833.022) (-5854.131) -- 9:45:47      1860000 -- (-5847.442) (-5848.939) [-5824.653] (-5845.760) * (-5838.336) (-5853.040) [-5826.162] (-5869.231) -- 9:45:42      Average standard deviation of split frequencies: 0.011411      1861000 -- (-5850.298) (-5841.460) [-5822.879] (-5820.593) * [-5815.584] (-5859.319) (-5854.091) (-5856.306) -- 9:45:36      1862000 -- (-5851.048) (-5864.273) [-5840.992] (-5827.508) * (-5818.095) (-5847.417) [-5842.317] (-5852.855) -- 9:45:30      1863000 -- (-5840.381) (-5849.856) (-5846.023) [-5826.215] * [-5818.373] (-5840.233) (-5842.326) (-5829.316) -- 9:45:29      1864000 -- (-5835.800) (-5856.577) (-5847.395) [-5854.335] * [-5809.046] (-5834.892) (-5841.183) (-5838.892) -- 9:45:23      1865000 -- [-5826.484] (-5848.027) (-5838.135) (-5835.748) * (-5809.004) (-5843.653) (-5850.281) [-5821.333] -- 9:45:17      Average standard deviation of split frequencies: 0.011277      1866000 -- [-5818.231] (-5852.174) (-5816.068) (-5835.535) * (-5832.155) (-5823.459) (-5857.340) [-5820.048] -- 9:45:12      1867000 -- (-5835.750) (-5837.030) [-5844.210] (-5844.865) * [-5819.213] (-5847.304) (-5840.489) (-5839.669) -- 9:45:06      1868000 -- (-5821.264) (-5853.726) [-5829.314] (-5831.818) * (-5833.160) (-5848.138) [-5839.437] (-5842.677) -- 9:45:05      1869000 -- [-5821.138] (-5853.977) (-5824.802) (-5840.907) * (-5842.858) (-5831.707) (-5854.623) [-5827.606] -- 9:44:59      1870000 -- [-5820.896] (-5858.262) (-5828.063) (-5831.736) * (-5843.680) (-5851.692) (-5836.258) [-5848.944] -- 9:44:53      Average standard deviation of split frequencies: 0.011135      1871000 -- (-5839.340) (-5848.056) (-5840.627) [-5837.717] * (-5817.014) (-5827.392) [-5836.126] (-5840.628) -- 9:44:52      1872000 -- (-5835.744) (-5842.142) [-5830.437] (-5846.935) * [-5822.202] (-5844.894) (-5836.356) (-5829.928) -- 9:44:46      1873000 -- (-5845.678) (-5865.871) (-5843.823) [-5838.183] * [-5810.223] (-5829.719) (-5842.564) (-5845.607) -- 9:44:41      1874000 -- (-5852.731) (-5876.488) (-5832.091) [-5825.526] * [-5836.224] (-5827.623) (-5851.221) (-5849.984) -- 9:44:35      1875000 -- (-5830.157) (-5841.714) (-5836.295) [-5815.690] * (-5846.370) (-5841.049) (-5840.543) [-5832.669] -- 9:44:34      Average standard deviation of split frequencies: 0.011198      1876000 -- [-5808.959] (-5853.676) (-5851.783) (-5829.767) * [-5828.543] (-5846.371) (-5840.784) (-5837.175) -- 9:44:28      1877000 -- [-5830.323] (-5854.091) (-5831.635) (-5843.634) * (-5839.977) (-5833.480) (-5860.457) [-5832.237] -- 9:44:22      1878000 -- (-5829.469) (-5845.673) (-5848.367) [-5829.178] * [-5830.970] (-5854.335) (-5849.467) (-5824.028) -- 9:44:16      1879000 -- (-5840.391) (-5840.016) (-5865.029) [-5827.528] * (-5832.530) (-5845.397) (-5879.619) [-5831.750] -- 9:44:11      1880000 -- (-5841.366) [-5826.562] (-5858.267) (-5828.781) * [-5833.537] (-5869.820) (-5874.699) (-5826.021) -- 9:44:09      Average standard deviation of split frequencies: 0.011177      1881000 -- (-5838.786) (-5841.865) (-5854.134) [-5822.531] * (-5834.311) (-5851.673) (-5854.996) [-5833.021] -- 9:44:04      1882000 -- (-5827.121) (-5864.578) (-5849.937) [-5822.935] * (-5831.166) (-5854.343) (-5864.958) [-5821.746] -- 9:43:58      1883000 -- [-5826.009] (-5862.184) (-5846.990) (-5829.157) * [-5831.088] (-5845.669) (-5867.890) (-5845.363) -- 9:43:52      1884000 -- [-5813.628] (-5836.994) (-5845.681) (-5852.217) * (-5844.619) (-5843.766) [-5839.307] (-5832.975) -- 9:43:47      1885000 -- [-5818.082] (-5859.526) (-5862.973) (-5860.459) * (-5841.710) (-5862.631) [-5834.011] (-5844.797) -- 9:43:45      Average standard deviation of split frequencies: 0.011191      1886000 -- (-5811.804) (-5864.799) (-5833.939) [-5824.367] * (-5854.114) (-5850.348) [-5842.778] (-5828.093) -- 9:43:40      1887000 -- (-5804.799) [-5840.872] (-5839.225) (-5846.656) * (-5846.890) [-5832.784] (-5855.236) (-5837.943) -- 9:43:34      1888000 -- [-5808.499] (-5844.615) (-5826.097) (-5841.390) * (-5867.157) [-5822.553] (-5850.667) (-5844.543) -- 9:43:33      1889000 -- [-5808.064] (-5855.201) (-5835.924) (-5854.127) * (-5860.718) [-5818.057] (-5841.143) (-5825.083) -- 9:43:27      1890000 -- [-5815.630] (-5857.069) (-5846.724) (-5841.106) * [-5824.422] (-5823.546) (-5845.713) (-5822.354) -- 9:43:21      Average standard deviation of split frequencies: 0.011159      1891000 -- [-5807.747] (-5841.747) (-5860.355) (-5832.486) * (-5823.975) [-5824.526] (-5838.252) (-5838.644) -- 9:43:20      1892000 -- [-5823.979] (-5832.735) (-5856.615) (-5844.411) * (-5851.422) [-5812.790] (-5848.695) (-5843.216) -- 9:43:14      1893000 -- (-5832.593) [-5844.071] (-5837.435) (-5841.007) * (-5828.473) (-5826.065) (-5844.381) [-5829.604] -- 9:43:13      1894000 -- (-5829.358) (-5856.213) (-5848.103) [-5821.683] * (-5838.354) [-5833.784] (-5877.327) (-5836.716) -- 9:43:11      1895000 -- (-5829.788) (-5855.581) (-5863.590) [-5820.568] * (-5846.829) [-5825.458] (-5871.329) (-5833.763) -- 9:43:19      Average standard deviation of split frequencies: 0.011036      1896000 -- (-5843.998) [-5841.118] (-5840.667) (-5832.020) * (-5838.712) (-5835.816) (-5879.166) [-5828.564] -- 9:43:17      1897000 -- (-5834.025) (-5844.228) (-5843.749) [-5822.797] * (-5845.961) [-5816.432] (-5875.800) (-5851.420) -- 9:43:16      1898000 -- (-5831.865) (-5846.586) [-5850.582] (-5822.046) * (-5835.567) [-5823.769] (-5857.588) (-5841.939) -- 9:43:14      1899000 -- (-5858.383) (-5846.288) (-5840.276) [-5821.242] * (-5828.596) [-5838.595] (-5858.375) (-5818.282) -- 9:43:09      1900000 -- [-5833.582] (-5840.038) (-5833.955) (-5844.177) * [-5827.616] (-5845.077) (-5843.264) (-5826.202) -- 9:43:07      Average standard deviation of split frequencies: 0.010927      1901000 -- [-5844.179] (-5844.130) (-5848.391) (-5831.614) * (-5824.630) (-5850.908) (-5862.416) [-5827.702] -- 9:43:02      1902000 -- (-5835.200) (-5838.828) [-5815.775] (-5824.246) * [-5819.772] (-5850.211) (-5846.508) (-5830.469) -- 9:43:00      1903000 -- [-5827.764] (-5822.416) (-5824.177) (-5849.978) * [-5817.848] (-5850.884) (-5829.948) (-5838.328) -- 9:42:54      1904000 -- (-5858.867) (-5842.718) [-5825.697] (-5864.048) * [-5814.409] (-5832.182) (-5849.454) (-5854.523) -- 9:42:53      1905000 -- (-5852.795) (-5842.081) [-5819.197] (-5839.682) * (-5820.046) (-5863.383) (-5837.561) [-5831.734] -- 9:42:47      Average standard deviation of split frequencies: 0.010620      1906000 -- (-5823.845) (-5834.928) [-5817.915] (-5850.204) * (-5825.656) (-5845.593) [-5823.221] (-5870.689) -- 9:42:42      1907000 -- (-5823.497) (-5847.791) [-5811.923] (-5852.484) * [-5813.989] (-5828.675) (-5852.141) (-5865.553) -- 9:42:40      1908000 -- [-5813.325] (-5851.811) (-5833.241) (-5853.817) * (-5838.238) [-5835.228] (-5852.365) (-5869.200) -- 9:42:35      1909000 -- (-5831.824) (-5840.798) [-5821.774] (-5854.599) * [-5830.879] (-5854.338) (-5847.727) (-5858.825) -- 9:42:33      1910000 -- (-5859.337) (-5832.497) (-5839.392) [-5833.537] * [-5831.872] (-5843.407) (-5878.483) (-5862.700) -- 9:42:27      Average standard deviation of split frequencies: 0.010523      1911000 -- (-5862.240) (-5836.653) (-5838.568) [-5821.898] * [-5820.878] (-5847.219) (-5855.547) (-5859.484) -- 9:42:22      1912000 -- (-5843.353) (-5821.286) [-5824.149] (-5841.874) * [-5827.380] (-5831.168) (-5860.287) (-5857.757) -- 9:42:20      1913000 -- (-5842.833) [-5829.525] (-5825.626) (-5837.067) * (-5843.168) [-5828.088] (-5876.373) (-5851.151) -- 9:42:15      1914000 -- [-5819.875] (-5832.754) (-5833.928) (-5840.111) * (-5833.986) [-5833.593] (-5872.380) (-5863.981) -- 9:42:09      1915000 -- (-5845.214) [-5830.310] (-5846.174) (-5847.066) * (-5834.729) [-5822.592] (-5873.922) (-5842.886) -- 9:42:08      Average standard deviation of split frequencies: 0.010501      1916000 -- (-5857.258) [-5835.300] (-5855.589) (-5829.292) * (-5828.825) [-5831.262] (-5863.730) (-5830.035) -- 9:42:02      1917000 -- (-5843.253) [-5832.611] (-5846.880) (-5837.067) * [-5834.939] (-5847.705) (-5854.173) (-5838.568) -- 9:41:56      1918000 -- (-5862.131) (-5840.215) [-5828.996] (-5832.212) * (-5828.714) [-5826.364] (-5859.142) (-5840.063) -- 9:41:55      1919000 -- (-5863.856) (-5831.166) (-5831.614) [-5825.578] * (-5842.744) [-5817.885] (-5852.185) (-5853.813) -- 9:41:49      1920000 -- (-5860.839) (-5837.002) [-5822.547] (-5845.373) * (-5842.355) (-5840.406) (-5858.500) [-5829.315] -- 9:41:48      Average standard deviation of split frequencies: 0.010557      1921000 -- (-5856.179) (-5840.444) (-5829.423) [-5844.159] * [-5825.145] (-5853.945) (-5861.182) (-5841.097) -- 9:41:42      1922000 -- (-5853.516) (-5834.351) (-5833.171) [-5830.880] * [-5831.974] (-5838.792) (-5853.992) (-5865.085) -- 9:41:36      1923000 -- (-5855.944) (-5834.008) (-5823.115) [-5826.944] * (-5836.042) [-5840.315] (-5843.407) (-5847.951) -- 9:41:35      1924000 -- (-5851.671) (-5829.041) [-5830.164] (-5846.976) * [-5843.189] (-5863.203) (-5845.197) (-5855.865) -- 9:41:29      1925000 -- (-5845.969) (-5831.084) [-5831.475] (-5838.822) * [-5841.233] (-5854.933) (-5841.043) (-5864.801) -- 9:41:24      Average standard deviation of split frequencies: 0.010564      1926000 -- (-5845.573) (-5846.329) [-5833.520] (-5866.274) * [-5826.233] (-5858.535) (-5830.957) (-5845.954) -- 9:41:22      1927000 -- (-5839.999) (-5839.356) (-5841.733) [-5836.315] * [-5825.506] (-5855.550) (-5835.854) (-5846.387) -- 9:41:16      1928000 -- (-5857.917) (-5830.090) [-5831.346] (-5828.389) * (-5837.737) [-5834.093] (-5830.337) (-5836.679) -- 9:41:11      1929000 -- (-5833.679) (-5852.792) [-5827.850] (-5858.003) * [-5833.210] (-5832.383) (-5839.516) (-5847.293) -- 9:41:09      1930000 -- (-5833.254) (-5837.818) (-5834.533) [-5834.839] * (-5844.741) [-5833.018] (-5857.485) (-5837.405) -- 9:41:04      Average standard deviation of split frequencies: 0.010655      1931000 -- (-5835.872) [-5831.950] (-5835.798) (-5829.122) * (-5852.517) (-5857.817) (-5845.452) [-5841.599] -- 9:40:58      1932000 -- (-5834.689) (-5845.721) [-5828.489] (-5832.463) * (-5828.813) (-5851.660) (-5836.095) [-5832.112] -- 9:40:56      1933000 -- (-5835.448) (-5859.974) [-5825.354] (-5841.374) * (-5852.109) [-5838.463] (-5836.733) (-5832.790) -- 9:40:51      1934000 -- (-5827.328) (-5855.555) [-5810.336] (-5834.085) * (-5852.477) (-5831.593) [-5840.237] (-5832.058) -- 9:40:45      1935000 -- (-5857.936) (-5842.015) (-5824.692) [-5826.286] * (-5864.035) (-5853.078) [-5845.407] (-5842.590) -- 9:40:44      Average standard deviation of split frequencies: 0.010749      1936000 -- (-5844.483) [-5822.722] (-5840.420) (-5840.076) * (-5837.065) (-5865.564) [-5843.955] (-5830.142) -- 9:40:38      1937000 -- (-5846.820) (-5830.624) (-5847.448) [-5836.203] * (-5821.536) (-5855.763) (-5832.413) [-5821.509] -- 9:40:32      1938000 -- (-5826.589) [-5828.802] (-5850.491) (-5852.483) * (-5834.254) (-5859.116) (-5834.978) [-5829.075] -- 9:40:27      1939000 -- (-5826.832) [-5836.574] (-5837.084) (-5851.012) * (-5830.959) (-5868.513) (-5835.766) [-5825.843] -- 9:40:25      1940000 -- [-5822.369] (-5842.642) (-5826.226) (-5842.861) * (-5838.529) (-5849.264) (-5836.191) [-5825.459] -- 9:40:20      Average standard deviation of split frequencies: 0.010649      1941000 -- [-5806.652] (-5856.674) (-5827.870) (-5835.273) * (-5840.862) (-5859.089) [-5827.162] (-5839.267) -- 9:40:14      1942000 -- [-5819.720] (-5855.935) (-5828.456) (-5835.978) * (-5846.328) (-5851.705) (-5834.186) [-5838.621] -- 9:40:12      1943000 -- (-5834.310) (-5863.815) (-5841.796) [-5832.385] * (-5847.755) [-5826.660] (-5843.499) (-5854.531) -- 9:40:07      1944000 -- (-5841.937) (-5868.806) [-5842.467] (-5832.838) * (-5862.433) (-5834.713) (-5831.294) [-5824.987] -- 9:40:01      1945000 -- (-5838.671) (-5854.248) (-5850.994) [-5831.308] * (-5833.115) (-5855.574) [-5817.345] (-5835.029) -- 9:40:00      Average standard deviation of split frequencies: 0.010668      1946000 -- [-5828.999] (-5835.711) (-5857.676) (-5837.742) * (-5850.552) (-5848.175) [-5833.724] (-5849.433) -- 9:39:54      1947000 -- (-5821.596) (-5848.926) (-5844.030) [-5838.047] * (-5841.342) (-5860.584) [-5833.877] (-5853.433) -- 9:39:52      1948000 -- [-5829.421] (-5856.745) (-5855.815) (-5832.691) * [-5819.537] (-5836.937) (-5843.955) (-5858.998) -- 9:39:47      1949000 -- (-5837.007) (-5851.545) (-5836.513) [-5830.987] * [-5817.734] (-5830.403) (-5821.773) (-5874.202) -- 9:39:41      1950000 -- (-5843.464) (-5838.376) [-5830.804] (-5836.991) * [-5822.493] (-5828.863) (-5827.569) (-5862.263) -- 9:39:36      Average standard deviation of split frequencies: 0.010626      1951000 -- (-5855.057) (-5830.227) (-5843.317) [-5834.897] * [-5816.574] (-5843.740) (-5829.180) (-5852.650) -- 9:39:30      1952000 -- [-5831.615] (-5873.331) (-5836.048) (-5830.936) * [-5825.844] (-5852.453) (-5827.972) (-5846.471) -- 9:39:28      1953000 -- (-5833.718) (-5852.874) (-5827.100) [-5831.464] * (-5821.970) (-5849.892) [-5822.986] (-5845.827) -- 9:39:23      1954000 -- (-5824.438) [-5834.270] (-5832.953) (-5836.559) * [-5835.990] (-5872.848) (-5828.624) (-5837.777) -- 9:39:17      1955000 -- (-5841.080) (-5833.109) [-5825.305] (-5852.622) * [-5821.331] (-5847.333) (-5825.116) (-5854.912) -- 9:39:16      Average standard deviation of split frequencies: 0.010503      1956000 -- (-5829.765) (-5842.094) [-5825.678] (-5841.802) * (-5848.772) (-5840.265) (-5819.712) [-5817.065] -- 9:39:10      1957000 -- [-5832.457] (-5839.420) (-5865.754) (-5831.468) * (-5854.706) (-5850.067) [-5830.685] (-5852.457) -- 9:39:04      1958000 -- (-5828.884) (-5854.886) (-5843.553) [-5807.873] * (-5833.259) (-5850.385) [-5823.219] (-5845.853) -- 9:38:59      1959000 -- (-5832.766) (-5843.268) (-5851.247) [-5799.576] * (-5838.003) (-5847.642) [-5824.320] (-5857.934) -- 9:38:57      1960000 -- (-5844.725) (-5844.636) (-5833.479) [-5823.085] * (-5830.409) [-5822.843] (-5821.316) (-5838.603) -- 9:38:51      Average standard deviation of split frequencies: 0.010610      1961000 -- (-5850.450) (-5845.734) [-5836.293] (-5827.964) * (-5830.629) [-5834.072] (-5828.849) (-5853.147) -- 9:38:46      1962000 -- (-5838.719) (-5859.574) (-5839.627) [-5833.857] * (-5836.612) (-5827.884) [-5830.683] (-5849.177) -- 9:38:44      1963000 -- (-5844.805) (-5836.686) [-5844.140] (-5835.014) * [-5829.430] (-5830.791) (-5828.115) (-5860.866) -- 9:38:39      1964000 -- (-5839.447) (-5861.521) [-5834.227] (-5854.305) * [-5822.989] (-5833.986) (-5830.001) (-5848.015) -- 9:38:33      1965000 -- (-5848.603) (-5862.522) [-5843.744] (-5856.802) * (-5841.248) (-5860.012) (-5829.403) [-5806.300] -- 9:38:27      Average standard deviation of split frequencies: 0.010717      1966000 -- (-5833.140) (-5875.075) (-5844.711) [-5838.414] * (-5834.343) (-5862.723) (-5819.977) [-5815.801] -- 9:38:26      1967000 -- (-5836.251) (-5846.999) (-5816.491) [-5828.539] * (-5862.115) (-5872.926) (-5832.047) [-5824.867] -- 9:38:20      1968000 -- (-5836.069) (-5841.065) [-5823.783] (-5830.649) * (-5849.733) (-5835.834) (-5821.544) [-5815.599] -- 9:38:15      1969000 -- (-5841.232) (-5826.079) [-5815.990] (-5840.325) * (-5861.506) (-5833.781) (-5836.445) [-5825.819] -- 9:38:09      1970000 -- (-5848.546) (-5822.677) (-5841.722) [-5852.936] * (-5873.976) [-5822.749] (-5846.918) (-5833.441) -- 9:38:07      Average standard deviation of split frequencies: 0.010636      1971000 -- (-5854.022) [-5812.070] (-5832.341) (-5834.411) * (-5877.246) (-5844.186) [-5819.738] (-5832.481) -- 9:38:02      1972000 -- (-5844.687) (-5823.657) [-5818.437] (-5837.254) * (-5884.065) (-5837.821) [-5825.864] (-5835.873) -- 9:37:56      1973000 -- (-5850.370) (-5839.366) [-5828.120] (-5832.111) * (-5879.347) (-5861.795) [-5832.396] (-5833.787) -- 9:37:55      1974000 -- [-5830.645] (-5824.583) (-5828.571) (-5831.887) * (-5875.971) (-5846.171) [-5839.019] (-5845.282) -- 9:37:49      1975000 -- (-5871.223) (-5840.718) [-5824.939] (-5839.172) * (-5866.766) (-5849.439) [-5827.689] (-5854.403) -- 9:37:43      Average standard deviation of split frequencies: 0.010707      1976000 -- (-5844.438) (-5834.330) (-5851.514) [-5832.468] * (-5856.196) (-5839.015) (-5833.819) [-5822.412] -- 9:37:42      1977000 -- (-5866.462) [-5827.004] (-5857.554) (-5826.730) * (-5862.811) [-5843.937] (-5828.236) (-5824.996) -- 9:37:36      1978000 -- (-5848.249) (-5841.531) [-5820.277] (-5836.163) * (-5858.118) (-5863.042) [-5825.260] (-5820.608) -- 9:37:31      1979000 -- (-5850.254) (-5848.529) [-5823.646] (-5847.517) * (-5866.508) (-5856.602) [-5830.335] (-5823.850) -- 9:37:25      1980000 -- (-5845.228) (-5840.681) [-5832.209] (-5845.607) * (-5870.046) [-5840.450] (-5850.118) (-5834.631) -- 9:37:19      Average standard deviation of split frequencies: 0.010650      1981000 -- [-5828.023] (-5833.181) (-5857.976) (-5834.049) * (-5865.509) (-5840.336) [-5822.770] (-5829.382) -- 9:37:18      1982000 -- (-5854.534) [-5829.194] (-5842.072) (-5822.345) * (-5854.680) (-5833.441) [-5823.217] (-5838.902) -- 9:37:12      1983000 -- (-5830.707) [-5818.527] (-5837.403) (-5816.555) * (-5845.208) (-5834.058) [-5824.740] (-5857.824) -- 9:37:07      1984000 -- (-5842.313) [-5816.965] (-5840.799) (-5827.690) * (-5832.606) [-5832.320] (-5832.526) (-5855.159) -- 9:37:05      1985000 -- (-5843.050) [-5822.036] (-5834.255) (-5858.478) * (-5846.170) [-5826.968] (-5843.092) (-5854.230) -- 9:36:59      Average standard deviation of split frequencies: 0.010350      1986000 -- (-5837.261) (-5861.060) (-5844.877) [-5829.118] * (-5837.610) [-5824.181] (-5847.530) (-5859.817) -- 9:36:54      1987000 -- (-5842.537) (-5852.047) (-5838.888) [-5830.501] * (-5853.765) [-5832.249] (-5829.425) (-5860.041) -- 9:36:48      1988000 -- (-5830.920) [-5838.032] (-5846.113) (-5838.053) * (-5847.853) [-5835.798] (-5826.819) (-5838.262) -- 9:36:47      1989000 -- [-5816.530] (-5835.830) (-5828.011) (-5873.962) * (-5847.261) [-5825.401] (-5837.615) (-5863.545) -- 9:36:41      1990000 -- [-5820.988] (-5843.141) (-5836.303) (-5879.421) * (-5821.054) (-5837.265) [-5837.564] (-5853.498) -- 9:36:35      Average standard deviation of split frequencies: 0.010154      1991000 -- (-5832.366) (-5856.390) (-5826.948) [-5844.751] * (-5825.127) (-5833.366) [-5836.500] (-5849.991) -- 9:36:30      1992000 -- (-5840.754) (-5848.777) [-5813.181] (-5856.855) * (-5833.308) (-5845.844) (-5860.304) [-5834.476] -- 9:36:28      1993000 -- (-5819.509) (-5842.654) [-5809.912] (-5843.992) * (-5818.629) (-5832.291) (-5864.200) [-5816.952] -- 9:36:23      1994000 -- (-5820.332) (-5853.572) [-5824.790] (-5837.350) * [-5809.145] (-5845.901) (-5856.533) (-5839.252) -- 9:36:17      1995000 -- (-5835.795) [-5825.716] (-5833.824) (-5833.054) * [-5806.193] (-5862.214) (-5834.642) (-5828.017) -- 9:36:15      Average standard deviation of split frequencies: 0.010057      1996000 -- (-5843.818) [-5829.916] (-5825.495) (-5843.060) * (-5811.539) (-5835.156) [-5827.672] (-5843.044) -- 9:36:10      1997000 -- (-5846.059) (-5837.136) [-5828.216] (-5857.275) * [-5801.523] (-5823.088) (-5822.719) (-5841.762) -- 9:36:04      1998000 -- (-5850.490) [-5836.962] (-5830.219) (-5827.754) * (-5809.285) [-5822.974] (-5850.068) (-5842.392) -- 9:35:59      1999000 -- (-5843.314) (-5828.459) [-5824.870] (-5834.715) * [-5796.432] (-5826.870) (-5840.699) (-5843.275) -- 9:35:57      2000000 -- (-5833.278) (-5850.897) [-5820.444] (-5838.057) * [-5805.980] (-5855.126) (-5824.172) (-5848.891) -- 9:35:52      Average standard deviation of split frequencies: 0.009842      2001000 -- (-5844.398) [-5810.704] (-5828.878) (-5847.060) * [-5802.610] (-5869.760) (-5844.964) (-5847.694) -- 9:35:46      2002000 -- [-5836.283] (-5823.194) (-5822.934) (-5846.250) * (-5830.892) (-5848.167) (-5856.001) [-5832.572] -- 9:35:40      2003000 -- [-5819.358] (-5826.349) (-5838.365) (-5834.515) * [-5822.067] (-5834.359) (-5836.715) (-5822.814) -- 9:35:35      2004000 -- [-5827.248] (-5835.248) (-5868.026) (-5827.079) * (-5837.432) [-5829.484] (-5844.723) (-5841.489) -- 9:35:33      2005000 -- [-5814.720] (-5835.293) (-5845.922) (-5836.113) * (-5836.481) (-5835.155) (-5838.824) [-5832.969] -- 9:35:28      Average standard deviation of split frequencies: 0.009713      2006000 -- [-5821.098] (-5834.926) (-5844.916) (-5825.763) * (-5846.730) (-5827.573) (-5828.884) [-5820.326] -- 9:35:22      2007000 -- (-5852.960) (-5837.166) (-5859.994) [-5835.759] * (-5844.578) (-5825.081) (-5819.434) [-5825.480] -- 9:35:16      2008000 -- (-5852.867) [-5826.903] (-5853.502) (-5834.599) * (-5866.597) (-5838.272) [-5820.893] (-5830.128) -- 9:35:15      2009000 -- (-5842.559) (-5827.742) (-5835.854) [-5834.827] * (-5862.603) (-5866.431) (-5833.831) [-5835.185] -- 9:35:09      2010000 -- (-5847.012) [-5818.827] (-5827.705) (-5814.946) * (-5829.668) (-5848.781) (-5820.246) [-5827.955] -- 9:35:04      Average standard deviation of split frequencies: 0.009547      2011000 -- (-5866.608) [-5830.174] (-5844.987) (-5832.007) * (-5851.040) (-5843.259) (-5829.315) [-5832.800] -- 9:34:58      2012000 -- (-5858.973) (-5829.854) [-5833.351] (-5835.378) * [-5846.981] (-5841.954) (-5834.119) (-5844.996) -- 9:34:56      2013000 -- (-5841.932) [-5828.957] (-5849.290) (-5828.351) * (-5858.957) (-5830.099) [-5822.199] (-5851.416) -- 9:34:51      2014000 -- (-5849.868) [-5817.910] (-5858.998) (-5847.774) * (-5845.616) (-5841.438) [-5821.494] (-5837.447) -- 9:34:45      2015000 -- (-5841.511) [-5829.687] (-5838.898) (-5842.086) * (-5875.846) (-5849.968) [-5829.488] (-5856.423) -- 9:34:40      Average standard deviation of split frequencies: 0.009413      2016000 -- [-5837.037] (-5831.899) (-5849.337) (-5853.643) * (-5863.507) (-5839.563) [-5827.512] (-5841.890) -- 9:34:38      2017000 -- (-5854.566) (-5821.299) (-5867.032) [-5814.292] * (-5851.891) (-5838.667) (-5827.676) [-5841.666] -- 9:34:32      2018000 -- (-5839.179) [-5818.089] (-5870.593) (-5846.363) * (-5841.172) (-5823.600) [-5826.564] (-5847.880) -- 9:34:27      2019000 -- (-5825.078) (-5830.440) (-5875.951) [-5817.787] * (-5845.830) [-5828.605] (-5849.306) (-5840.530) -- 9:34:25      2020000 -- [-5821.158] (-5815.732) (-5883.539) (-5837.976) * (-5840.470) [-5820.406] (-5849.742) (-5835.269) -- 9:34:20      Average standard deviation of split frequencies: 0.009443      2021000 -- [-5830.654] (-5825.958) (-5879.104) (-5847.694) * (-5838.732) [-5810.167] (-5862.391) (-5834.612) -- 9:34:14      2022000 -- (-5837.134) [-5821.781] (-5855.346) (-5864.721) * (-5831.852) [-5809.161] (-5840.319) (-5849.478) -- 9:34:12      2023000 -- (-5838.707) [-5816.069] (-5843.326) (-5843.340) * [-5822.699] (-5817.167) (-5856.853) (-5843.713) -- 9:34:07      2024000 -- (-5845.009) [-5837.964] (-5840.404) (-5840.164) * [-5826.258] (-5832.882) (-5851.964) (-5828.279) -- 9:34:01      2025000 -- (-5834.618) (-5858.149) (-5842.511) [-5829.839] * [-5820.811] (-5816.424) (-5857.593) (-5830.939) -- 9:34:00      Average standard deviation of split frequencies: 0.009515      2026000 -- (-5840.518) (-5862.413) (-5845.200) [-5822.698] * (-5836.686) [-5817.475] (-5861.611) (-5850.237) -- 9:33:58      2027000 -- (-5862.133) (-5830.521) [-5824.888] (-5844.017) * [-5815.285] (-5818.978) (-5877.177) (-5828.048) -- 9:34:00      2028000 -- (-5848.854) [-5821.707] (-5843.605) (-5838.005) * [-5829.027] (-5838.963) (-5876.019) (-5828.120) -- 9:33:55      2029000 -- (-5841.775) [-5838.760] (-5837.979) (-5835.076) * [-5818.326] (-5842.382) (-5889.569) (-5820.412) -- 9:33:53      2030000 -- (-5842.808) (-5846.870) (-5822.812) [-5833.721] * (-5850.887) (-5831.305) (-5847.007) [-5832.959] -- 9:33:55      Average standard deviation of split frequencies: 0.009427      2031000 -- (-5855.002) (-5850.688) [-5827.281] (-5848.236) * (-5838.813) (-5845.577) (-5890.188) [-5819.583] -- 9:33:54      2032000 -- (-5858.990) (-5875.608) [-5832.620] (-5839.013) * [-5820.084] (-5848.824) (-5865.940) (-5831.867) -- 9:33:48      2033000 -- (-5879.365) [-5835.736] (-5836.383) (-5836.118) * [-5824.425] (-5839.318) (-5861.697) (-5839.532) -- 9:33:47      2034000 -- (-5855.241) (-5828.378) (-5847.365) [-5829.112] * [-5814.632] (-5842.857) (-5855.600) (-5834.176) -- 9:33:41      2035000 -- (-5869.777) [-5832.720] (-5854.081) (-5826.188) * (-5818.108) (-5845.412) [-5828.920] (-5859.869) -- 9:33:35      Average standard deviation of split frequencies: 0.009392      2036000 -- (-5873.934) (-5818.118) (-5857.952) [-5829.688] * [-5835.111] (-5834.536) (-5848.510) (-5844.063) -- 9:33:34      2037000 -- (-5866.762) (-5847.240) (-5850.005) [-5814.739] * [-5820.523] (-5851.429) (-5831.474) (-5830.891) -- 9:33:28      2038000 -- (-5851.895) (-5856.270) (-5837.324) [-5826.485] * [-5822.430] (-5839.004) (-5831.357) (-5837.785) -- 9:33:23      2039000 -- (-5845.316) (-5849.517) [-5827.423] (-5820.441) * (-5839.193) (-5849.818) [-5819.437] (-5827.407) -- 9:33:21      2040000 -- (-5837.483) (-5845.842) (-5830.512) [-5824.440] * (-5843.394) (-5863.680) (-5834.454) [-5832.370] -- 9:33:15      Average standard deviation of split frequencies: 0.009334      2041000 -- (-5841.488) [-5835.476] (-5869.875) (-5821.517) * (-5861.159) (-5857.365) [-5828.602] (-5828.992) -- 9:33:10      2042000 -- [-5834.740] (-5883.659) (-5847.290) (-5845.894) * (-5845.586) (-5832.789) (-5859.633) [-5838.552] -- 9:33:04      2043000 -- (-5846.820) [-5836.157] (-5874.580) (-5830.693) * [-5832.941] (-5833.462) (-5828.630) (-5855.556) -- 9:33:02      2044000 -- [-5826.913] (-5835.250) (-5871.168) (-5842.452) * (-5845.278) (-5842.810) (-5873.106) [-5844.626] -- 9:32:57      2045000 -- (-5849.040) [-5837.103] (-5883.709) (-5841.903) * (-5835.485) (-5856.423) (-5874.818) [-5839.188] -- 9:32:51      Average standard deviation of split frequencies: 0.009460      2046000 -- (-5836.511) [-5832.215] (-5873.711) (-5839.701) * (-5851.697) (-5861.280) (-5871.256) [-5817.910] -- 9:32:46      2047000 -- [-5835.150] (-5835.691) (-5863.093) (-5865.397) * (-5827.267) (-5844.304) (-5850.836) [-5825.051] -- 9:32:40      2048000 -- [-5824.206] (-5843.749) (-5863.581) (-5878.127) * (-5836.616) (-5844.415) (-5862.894) [-5834.375] -- 9:32:35      2049000 -- (-5848.829) [-5833.614] (-5899.145) (-5841.475) * [-5831.537] (-5841.049) (-5871.398) (-5832.389) -- 9:32:29      2050000 -- (-5841.672) (-5833.056) (-5863.675) [-5838.476] * [-5824.590] (-5829.646) (-5855.227) (-5849.263) -- 9:32:27      Average standard deviation of split frequencies: 0.009402      2051000 -- [-5850.541] (-5842.932) (-5867.017) (-5828.192) * (-5827.458) (-5840.019) [-5843.724] (-5825.480) -- 9:32:22      2052000 -- (-5837.526) (-5836.262) (-5886.955) [-5813.418] * [-5847.538] (-5839.067) (-5844.312) (-5815.718) -- 9:32:16      2053000 -- (-5841.154) (-5840.099) (-5857.685) [-5837.054] * (-5841.443) (-5819.055) (-5844.872) [-5822.879] -- 9:32:11      2054000 -- (-5843.946) [-5837.657] (-5855.955) (-5813.834) * (-5829.875) (-5837.739) [-5834.781] (-5848.772) -- 9:32:09      2055000 -- (-5844.159) [-5836.145] (-5877.601) (-5827.118) * [-5831.809] (-5845.800) (-5854.894) (-5847.645) -- 9:32:03      Average standard deviation of split frequencies: 0.009309      2056000 -- [-5824.436] (-5845.653) (-5856.699) (-5849.010) * [-5844.819] (-5856.129) (-5844.462) (-5847.194) -- 9:31:58      2057000 -- [-5835.303] (-5833.190) (-5840.375) (-5837.618) * (-5845.488) (-5855.630) [-5831.187] (-5841.940) -- 9:31:52      2058000 -- (-5834.389) (-5830.423) (-5865.393) [-5830.015] * (-5847.631) (-5862.563) (-5842.627) [-5828.196] -- 9:31:51      2059000 -- (-5853.387) (-5829.791) (-5885.000) [-5814.461] * (-5830.137) (-5856.574) (-5846.149) [-5822.516] -- 9:31:45      2060000 -- (-5853.911) (-5834.738) (-5878.263) [-5828.966] * [-5838.695] (-5871.556) (-5875.576) (-5832.978) -- 9:31:40      Average standard deviation of split frequencies: 0.009364      2061000 -- (-5842.244) (-5840.265) (-5865.116) [-5829.792] * (-5833.052) (-5863.381) (-5870.228) [-5829.622] -- 9:31:38      2062000 -- (-5835.766) (-5866.875) (-5847.325) [-5832.497] * [-5829.796] (-5864.914) (-5850.981) (-5843.206) -- 9:31:32      2063000 -- (-5835.332) (-5865.757) (-5840.540) [-5833.128] * (-5831.876) (-5829.866) (-5860.359) [-5847.755] -- 9:31:27      2064000 -- [-5821.743] (-5858.513) (-5835.977) (-5845.812) * [-5820.454] (-5821.972) (-5875.288) (-5845.575) -- 9:31:25      2065000 -- [-5820.813] (-5845.531) (-5839.182) (-5877.046) * [-5814.687] (-5832.188) (-5872.209) (-5843.276) -- 9:31:19      Average standard deviation of split frequencies: 0.009410      2066000 -- (-5841.683) (-5874.459) [-5831.118] (-5883.715) * [-5832.838] (-5838.337) (-5859.364) (-5847.184) -- 9:31:14      2067000 -- [-5829.286] (-5851.610) (-5831.234) (-5861.416) * (-5832.402) [-5821.162] (-5841.231) (-5864.281) -- 9:31:12      2068000 -- [-5831.450] (-5857.522) (-5853.383) (-5823.352) * [-5836.674] (-5842.723) (-5849.644) (-5853.376) -- 9:31:07      2069000 -- (-5835.959) (-5868.810) (-5864.307) [-5828.853] * (-5848.540) [-5827.878] (-5848.869) (-5839.245) -- 9:31:01      2070000 -- (-5825.986) (-5885.922) (-5857.293) [-5830.417] * (-5819.449) [-5821.189] (-5863.943) (-5867.556) -- 9:30:59      Average standard deviation of split frequencies: 0.009394      2071000 -- (-5824.136) (-5856.697) (-5856.060) [-5821.111] * [-5818.900] (-5830.817) (-5845.698) (-5868.295) -- 9:30:54      2072000 -- (-5829.903) (-5854.131) (-5868.114) [-5816.512] * (-5841.116) [-5824.050] (-5842.426) (-5852.297) -- 9:30:48      2073000 -- [-5819.865] (-5857.141) (-5853.427) (-5824.368) * [-5818.471] (-5835.090) (-5855.549) (-5864.181) -- 9:30:43      2074000 -- [-5823.088] (-5843.470) (-5855.184) (-5834.060) * [-5830.793] (-5828.661) (-5860.313) (-5866.470) -- 9:30:41      2075000 -- (-5828.656) [-5833.989] (-5879.114) (-5862.371) * [-5823.899] (-5854.455) (-5868.395) (-5855.114) -- 9:30:36      Average standard deviation of split frequencies: 0.009380      2076000 -- [-5824.647] (-5841.972) (-5877.553) (-5849.805) * [-5825.657] (-5853.732) (-5844.812) (-5867.649) -- 9:30:30      2077000 -- [-5822.943] (-5854.478) (-5867.378) (-5846.367) * [-5819.962] (-5857.328) (-5841.606) (-5862.431) -- 9:30:24      2078000 -- [-5821.282] (-5845.036) (-5850.752) (-5854.281) * [-5816.273] (-5848.076) (-5861.615) (-5861.954) -- 9:30:23      2079000 -- [-5822.850] (-5822.499) (-5850.585) (-5852.828) * (-5835.758) [-5849.275] (-5839.328) (-5867.017) -- 9:30:17      2080000 -- (-5836.427) [-5819.881] (-5859.570) (-5844.657) * [-5832.824] (-5852.113) (-5840.235) (-5865.313) -- 9:30:15      Average standard deviation of split frequencies: 0.009315      2081000 -- [-5829.499] (-5818.706) (-5868.128) (-5848.134) * [-5827.178] (-5846.027) (-5847.446) (-5857.729) -- 9:30:10      2082000 -- (-5843.758) [-5823.960] (-5846.269) (-5854.698) * [-5821.532] (-5852.154) (-5845.914) (-5851.342) -- 9:30:04      2083000 -- (-5850.865) [-5827.726] (-5846.242) (-5860.998) * (-5846.572) [-5829.443] (-5849.284) (-5877.109) -- 9:29:59      2084000 -- (-5850.686) [-5817.549] (-5832.998) (-5872.267) * (-5843.833) [-5811.889] (-5864.276) (-5869.653) -- 9:29:57      2085000 -- (-5834.214) [-5819.899] (-5839.197) (-5856.386) * (-5852.091) [-5816.990] (-5832.525) (-5861.283) -- 9:29:52      Average standard deviation of split frequencies: 0.009390      2086000 -- (-5848.670) [-5830.328] (-5872.339) (-5855.786) * (-5859.688) [-5818.542] (-5833.084) (-5870.428) -- 9:29:46      2087000 -- (-5848.796) [-5818.702] (-5885.500) (-5836.283) * (-5865.802) [-5825.560] (-5827.832) (-5869.760) -- 9:29:44      2088000 -- (-5848.063) [-5826.606] (-5835.338) (-5840.602) * (-5848.727) [-5811.992] (-5846.325) (-5852.650) -- 9:29:39      2089000 -- (-5861.907) (-5826.758) [-5829.945] (-5844.120) * (-5851.682) [-5821.310] (-5832.004) (-5864.186) -- 9:29:33      2090000 -- (-5863.394) (-5827.901) [-5825.877] (-5852.623) * (-5856.457) [-5830.648] (-5836.301) (-5860.956) -- 9:29:31      Average standard deviation of split frequencies: 0.009333      2091000 -- (-5852.187) (-5827.180) [-5826.906] (-5835.397) * [-5836.747] (-5834.336) (-5836.210) (-5867.500) -- 9:29:26      2092000 -- (-5867.335) [-5812.339] (-5831.715) (-5843.992) * [-5832.695] (-5835.427) (-5831.594) (-5844.396) -- 9:29:20      2093000 -- (-5852.576) (-5825.898) [-5832.463] (-5852.556) * (-5854.301) (-5842.040) [-5815.614] (-5845.268) -- 9:29:19      2094000 -- (-5856.062) [-5824.378] (-5825.648) (-5836.354) * (-5852.945) (-5841.118) [-5822.888] (-5833.545) -- 9:29:13      2095000 -- (-5858.623) [-5825.850] (-5821.284) (-5852.825) * (-5851.320) (-5828.609) (-5828.391) [-5821.433] -- 9:29:08      Average standard deviation of split frequencies: 0.009348      2096000 -- (-5866.882) [-5835.067] (-5840.432) (-5852.204) * (-5850.458) (-5828.755) (-5839.334) [-5824.127] -- 9:29:06      2097000 -- (-5860.575) [-5826.853] (-5832.542) (-5843.356) * (-5882.109) (-5828.283) (-5842.605) [-5825.749] -- 9:29:00      2098000 -- (-5838.607) (-5832.707) [-5827.010] (-5837.567) * (-5845.913) (-5825.453) (-5841.889) [-5829.899] -- 9:28:55      2099000 -- (-5853.088) [-5832.678] (-5838.217) (-5838.320) * (-5844.968) [-5831.167] (-5841.442) (-5831.596) -- 9:28:53      2100000 -- (-5853.815) (-5823.226) [-5839.368] (-5852.314) * [-5827.802] (-5827.256) (-5863.686) (-5845.979) -- 9:28:48      Average standard deviation of split frequencies: 0.009451      2101000 -- (-5845.184) [-5832.105] (-5848.354) (-5859.091) * (-5845.318) [-5825.540] (-5862.522) (-5849.559) -- 9:28:42      2102000 -- (-5845.807) (-5836.127) [-5827.668] (-5855.473) * (-5842.511) [-5821.518] (-5845.301) (-5858.491) -- 9:28:40      2103000 -- (-5844.858) (-5844.909) (-5838.804) [-5832.093] * (-5840.934) [-5829.299] (-5862.740) (-5836.079) -- 9:28:35      2104000 -- (-5837.359) (-5841.315) [-5828.727] (-5816.098) * [-5827.315] (-5827.923) (-5862.447) (-5843.858) -- 9:28:29      2105000 -- (-5864.142) (-5836.832) [-5825.388] (-5821.507) * [-5823.142] (-5833.940) (-5847.054) (-5869.410) -- 9:28:27      Average standard deviation of split frequencies: 0.009508      2106000 -- (-5865.584) (-5834.691) [-5831.574] (-5828.491) * [-5831.151] (-5834.213) (-5849.158) (-5875.057) -- 9:28:22      2107000 -- (-5863.193) (-5828.527) [-5821.090] (-5833.979) * (-5842.257) [-5834.299] (-5864.080) (-5865.722) -- 9:28:16      2108000 -- (-5854.799) [-5825.657] (-5843.765) (-5827.315) * (-5840.152) [-5833.018] (-5862.795) (-5849.086) -- 9:28:11      2109000 -- (-5835.238) (-5844.083) [-5838.590] (-5853.412) * (-5841.970) (-5819.060) (-5853.605) [-5851.074] -- 9:28:09      2110000 -- [-5844.901] (-5861.785) (-5828.624) (-5842.669) * (-5826.986) [-5814.676] (-5855.226) (-5856.624) -- 9:28:04      Average standard deviation of split frequencies: 0.009391      2111000 -- (-5865.532) (-5853.650) [-5817.164] (-5833.473) * (-5837.983) [-5821.307] (-5854.146) (-5840.456) -- 9:27:58      2112000 -- (-5848.178) (-5845.570) (-5841.196) [-5841.754] * [-5816.071] (-5848.474) (-5838.709) (-5825.896) -- 9:27:53      2113000 -- [-5827.949] (-5831.534) (-5821.572) (-5846.154) * (-5819.065) (-5847.865) (-5842.699) [-5826.880] -- 9:27:51      2114000 -- (-5834.715) (-5836.891) [-5810.890] (-5838.787) * (-5812.786) [-5836.571] (-5857.811) (-5821.864) -- 9:27:45      2115000 -- (-5847.224) (-5831.275) [-5807.962] (-5849.793) * [-5812.927] (-5834.388) (-5831.536) (-5848.331) -- 9:27:40      Average standard deviation of split frequencies: 0.009349      2116000 -- (-5857.715) (-5838.685) [-5822.672] (-5834.900) * [-5808.286] (-5848.250) (-5835.328) (-5851.796) -- 9:27:38      2117000 -- (-5838.930) (-5850.417) [-5830.466] (-5847.770) * (-5830.308) [-5835.312] (-5835.223) (-5849.334) -- 9:27:32      2118000 -- (-5862.319) [-5826.781] (-5821.574) (-5847.181) * (-5830.991) [-5838.129] (-5824.864) (-5842.988) -- 9:27:27      2119000 -- (-5848.254) [-5813.710] (-5833.251) (-5844.755) * (-5835.672) (-5848.948) (-5834.302) [-5826.022] -- 9:27:21      2120000 -- (-5855.327) [-5815.457] (-5845.597) (-5843.306) * (-5824.506) (-5835.638) [-5821.298] (-5851.110) -- 9:27:20      Average standard deviation of split frequencies: 0.009349      2121000 -- (-5845.966) [-5813.133] (-5864.799) (-5852.917) * (-5840.305) (-5832.691) [-5820.576] (-5850.172) -- 9:27:14      2122000 -- (-5855.327) (-5829.110) (-5862.207) [-5840.762] * (-5831.948) (-5854.830) [-5814.720] (-5877.812) -- 9:27:09      2123000 -- (-5850.542) [-5823.150] (-5841.760) (-5844.999) * (-5829.414) (-5843.286) [-5824.570] (-5875.727) -- 9:27:07      2124000 -- [-5852.186] (-5826.872) (-5842.344) (-5851.250) * (-5833.402) (-5850.952) [-5820.075] (-5862.141) -- 9:27:01      2125000 -- (-5838.698) [-5833.068] (-5868.955) (-5867.871) * [-5820.218] (-5836.720) (-5821.762) (-5871.440) -- 9:26:56      Average standard deviation of split frequencies: 0.009237      2126000 -- (-5831.388) [-5822.713] (-5842.614) (-5848.982) * [-5825.975] (-5821.521) (-5839.573) (-5875.068) -- 9:26:54      2127000 -- (-5852.328) [-5825.904] (-5832.316) (-5856.837) * [-5833.827] (-5823.426) (-5842.052) (-5839.645) -- 9:26:48      2128000 -- (-5823.161) (-5833.318) [-5822.529] (-5883.094) * [-5828.689] (-5820.927) (-5860.406) (-5858.033) -- 9:26:43      2129000 -- (-5869.236) [-5848.194] (-5837.330) (-5869.874) * (-5855.428) [-5821.400] (-5858.867) (-5843.657) -- 9:26:37      2130000 -- (-5861.168) (-5841.479) [-5823.636] (-5850.779) * (-5856.549) [-5805.447] (-5844.886) (-5857.692) -- 9:26:36      Average standard deviation of split frequencies: 0.009029      2131000 -- [-5838.068] (-5853.795) (-5821.822) (-5839.281) * (-5850.460) [-5817.363] (-5866.206) (-5834.959) -- 9:26:30      2132000 -- [-5832.335] (-5850.649) (-5837.199) (-5848.217) * (-5859.047) (-5830.462) (-5858.225) [-5825.941] -- 9:26:25      2133000 -- (-5840.041) (-5871.446) [-5822.059] (-5831.210) * (-5839.472) (-5833.344) (-5856.262) [-5823.670] -- 9:26:19      2134000 -- (-5826.009) (-5865.014) [-5819.921] (-5832.988) * (-5835.442) (-5830.917) (-5849.031) [-5816.923] -- 9:26:17      2135000 -- [-5825.673] (-5879.107) (-5847.748) (-5829.384) * (-5851.204) [-5816.494] (-5849.021) (-5816.778) -- 9:26:12      Average standard deviation of split frequencies: 0.008848      2136000 -- [-5828.645] (-5862.968) (-5857.539) (-5836.040) * (-5864.163) (-5827.407) (-5857.416) [-5822.606] -- 9:26:06      2137000 -- [-5820.115] (-5856.908) (-5870.567) (-5837.386) * (-5879.677) [-5808.384] (-5844.065) (-5847.325) -- 9:26:01      2138000 -- [-5822.867] (-5841.453) (-5856.099) (-5851.102) * (-5878.399) [-5817.742] (-5819.771) (-5843.586) -- 9:25:59      2139000 -- [-5821.571] (-5857.478) (-5856.898) (-5835.452) * (-5869.734) (-5832.893) (-5854.738) [-5827.840] -- 9:25:54      2140000 -- [-5838.260] (-5849.703) (-5844.012) (-5832.839) * (-5862.164) (-5827.716) (-5834.433) [-5829.457] -- 9:25:48      Average standard deviation of split frequencies: 0.008666      2141000 -- [-5824.533] (-5866.604) (-5849.935) (-5837.223) * (-5859.233) (-5843.838) (-5837.291) [-5839.618] -- 9:25:43      2142000 -- [-5824.615] (-5862.315) (-5833.481) (-5841.141) * (-5830.220) (-5828.732) [-5813.601] (-5842.562) -- 9:25:41      2143000 -- [-5831.490] (-5847.897) (-5844.674) (-5872.982) * (-5848.253) [-5840.782] (-5835.718) (-5843.950) -- 9:25:35      2144000 -- [-5828.025] (-5820.874) (-5856.463) (-5859.927) * (-5838.823) [-5818.264] (-5840.853) (-5877.353) -- 9:25:30      2145000 -- [-5823.993] (-5835.226) (-5831.721) (-5853.163) * (-5844.436) [-5817.045] (-5831.893) (-5865.409) -- 9:25:24      Average standard deviation of split frequencies: 0.008524      2146000 -- (-5839.777) [-5830.310] (-5839.160) (-5850.399) * (-5854.671) [-5823.339] (-5841.028) (-5824.814) -- 9:25:22      2147000 -- (-5832.524) [-5822.440] (-5841.589) (-5852.094) * (-5834.494) [-5830.130] (-5859.901) (-5842.269) -- 9:25:17      2148000 -- (-5828.888) [-5820.007] (-5837.120) (-5856.943) * [-5834.812] (-5845.951) (-5870.134) (-5844.070) -- 9:25:12      2149000 -- (-5850.807) (-5829.250) (-5843.960) [-5836.678] * [-5818.679] (-5834.869) (-5862.898) (-5848.959) -- 9:25:06      2150000 -- (-5853.922) [-5835.664] (-5837.466) (-5847.319) * [-5835.897] (-5832.479) (-5853.015) (-5838.110) -- 9:25:04      Average standard deviation of split frequencies: 0.008655      2151000 -- (-5846.671) (-5822.732) (-5835.075) [-5833.010] * (-5836.330) [-5819.683] (-5833.091) (-5846.584) -- 9:24:59      2152000 -- (-5847.084) [-5824.622] (-5833.089) (-5831.642) * (-5823.381) (-5831.666) [-5837.030] (-5870.146) -- 9:24:53      2153000 -- (-5856.301) (-5838.325) (-5841.784) [-5819.053] * [-5820.455] (-5832.586) (-5845.117) (-5866.549) -- 9:24:48      2154000 -- (-5890.895) (-5833.922) (-5828.047) [-5819.024] * (-5824.166) [-5821.157] (-5829.877) (-5875.101) -- 9:24:42      2155000 -- (-5860.523) (-5833.296) (-5829.565) [-5826.961] * (-5828.890) [-5820.171] (-5828.310) (-5837.298) -- 9:24:37      Average standard deviation of split frequencies: 0.008583      2156000 -- (-5856.506) (-5844.053) [-5822.204] (-5841.411) * (-5819.449) [-5821.755] (-5831.576) (-5854.900) -- 9:24:35      2157000 -- (-5855.461) (-5840.267) [-5818.962] (-5848.220) * (-5816.691) (-5831.466) [-5826.502] (-5863.123) -- 9:24:29      2158000 -- (-5852.315) (-5845.529) (-5838.861) [-5837.270] * (-5825.738) (-5831.142) [-5841.205] (-5838.086) -- 9:24:24      2159000 -- (-5846.364) (-5848.488) [-5826.532] (-5839.053) * [-5824.142] (-5838.046) (-5842.092) (-5838.602) -- 9:24:19      2160000 -- (-5848.656) (-5842.753) [-5829.269] (-5846.146) * (-5824.973) (-5840.956) [-5823.265] (-5840.202) -- 9:24:13      Average standard deviation of split frequencies: 0.008578      2161000 -- (-5844.921) (-5849.240) [-5822.249] (-5831.258) * [-5808.875] (-5862.199) (-5825.248) (-5858.554) -- 9:24:11      2162000 -- (-5845.678) (-5853.350) [-5834.783] (-5816.994) * [-5806.731] (-5868.711) (-5837.493) (-5850.502) -- 9:24:06      2163000 -- (-5836.580) (-5846.835) [-5816.558] (-5831.107) * [-5821.069] (-5843.596) (-5843.124) (-5842.732) -- 9:24:00      2164000 -- (-5828.698) (-5867.283) [-5817.640] (-5835.600) * [-5828.720] (-5835.700) (-5855.676) (-5847.288) -- 9:23:55      2165000 -- [-5833.241] (-5858.108) (-5843.701) (-5850.577) * [-5832.396] (-5850.314) (-5861.880) (-5849.490) -- 9:23:53      Average standard deviation of split frequencies: 0.008521      2166000 -- (-5832.533) [-5830.069] (-5838.455) (-5844.769) * [-5817.616] (-5848.985) (-5845.382) (-5835.638) -- 9:23:47      2167000 -- [-5821.554] (-5837.561) (-5829.502) (-5863.027) * (-5826.471) [-5836.628] (-5862.428) (-5844.793) -- 9:23:42      2168000 -- [-5826.218] (-5823.970) (-5845.675) (-5843.338) * [-5835.635] (-5846.457) (-5861.066) (-5837.686) -- 9:23:37      2169000 -- [-5824.960] (-5819.038) (-5861.932) (-5852.156) * [-5821.725] (-5850.894) (-5858.639) (-5832.756) -- 9:23:31      2170000 -- [-5821.108] (-5816.973) (-5858.484) (-5839.748) * (-5832.739) (-5823.949) (-5855.753) [-5832.838] -- 9:23:29      Average standard deviation of split frequencies: 0.008638      2171000 -- [-5814.618] (-5844.178) (-5821.555) (-5844.999) * (-5840.931) [-5845.726] (-5849.173) (-5829.861) -- 9:23:24      2172000 -- [-5820.838] (-5834.678) (-5818.348) (-5843.286) * [-5833.546] (-5831.679) (-5847.848) (-5833.210) -- 9:23:18      2173000 -- (-5816.986) (-5846.803) [-5814.921] (-5844.217) * (-5855.686) (-5841.906) [-5841.998] (-5847.045) -- 9:23:13      2174000 -- [-5817.743] (-5831.523) (-5846.527) (-5835.810) * (-5849.035) [-5829.311] (-5829.853) (-5844.539) -- 9:23:07      2175000 -- (-5825.903) (-5826.392) [-5845.424] (-5835.411) * (-5835.850) (-5843.322) [-5842.808] (-5836.669) -- 9:23:06      Average standard deviation of split frequencies: 0.008823      2176000 -- [-5823.870] (-5833.832) (-5847.908) (-5850.293) * (-5831.350) [-5841.200] (-5832.257) (-5842.014) -- 9:23:00      2177000 -- [-5817.738] (-5851.804) (-5830.110) (-5844.859) * [-5821.188] (-5846.385) (-5819.484) (-5844.314) -- 9:22:55      2178000 -- [-5817.197] (-5859.790) (-5830.789) (-5833.497) * [-5825.079] (-5842.008) (-5839.819) (-5840.887) -- 9:22:49      2179000 -- (-5816.671) (-5864.768) (-5852.812) [-5822.184] * (-5828.256) (-5852.695) (-5850.519) [-5829.785] -- 9:22:44      2180000 -- (-5836.423) (-5838.090) (-5865.443) [-5837.844] * (-5824.195) (-5844.375) (-5864.902) [-5836.444] -- 9:22:42      Average standard deviation of split frequencies: 0.008695      2181000 -- [-5822.420] (-5845.435) (-5868.465) (-5832.412) * [-5821.204] (-5849.445) (-5854.651) (-5832.884) -- 9:22:36      2182000 -- (-5829.061) (-5839.920) (-5848.175) [-5832.081] * (-5826.577) (-5847.911) (-5839.371) [-5814.383] -- 9:22:31      2183000 -- [-5819.186] (-5841.480) (-5850.519) (-5850.854) * (-5850.551) [-5823.032] (-5825.265) (-5838.261) -- 9:22:25      2184000 -- (-5840.559) [-5822.003] (-5843.430) (-5877.417) * (-5850.565) [-5825.622] (-5831.494) (-5857.134) -- 9:22:24      2185000 -- (-5830.361) [-5813.578] (-5849.747) (-5852.760) * (-5828.079) [-5815.642] (-5831.865) (-5858.351) -- 9:22:18      Average standard deviation of split frequencies: 0.008796      2186000 -- (-5818.649) [-5820.893] (-5854.902) (-5861.958) * [-5833.569] (-5819.352) (-5848.862) (-5841.333) -- 9:22:13      2187000 -- [-5815.327] (-5857.872) (-5825.967) (-5865.147) * (-5830.364) (-5832.902) [-5826.664] (-5856.834) -- 9:22:11      2188000 -- [-5814.894] (-5863.639) (-5832.877) (-5856.564) * (-5838.051) (-5846.517) [-5820.584] (-5831.982) -- 9:22:05      2189000 -- [-5812.152] (-5889.220) (-5852.525) (-5863.538) * (-5834.857) [-5817.606] (-5820.944) (-5854.553) -- 9:22:00      2190000 -- [-5806.429] (-5845.016) (-5858.260) (-5865.945) * (-5853.524) (-5833.538) [-5824.222] (-5849.426) -- 9:21:54      Average standard deviation of split frequencies: 0.008833      2191000 -- [-5820.443] (-5848.819) (-5866.050) (-5845.075) * (-5852.546) (-5835.578) [-5825.213] (-5852.002) -- 9:21:49      2192000 -- [-5824.261] (-5830.741) (-5885.597) (-5855.912) * (-5870.644) (-5828.271) (-5846.726) [-5837.060] -- 9:21:47      2193000 -- (-5817.796) (-5833.218) [-5833.399] (-5842.802) * (-5838.113) [-5829.605] (-5853.032) (-5846.140) -- 9:21:42      2194000 -- [-5820.433] (-5835.404) (-5836.472) (-5844.309) * (-5852.179) (-5819.080) (-5868.656) [-5834.302] -- 9:21:36      2195000 -- [-5813.539] (-5830.590) (-5836.681) (-5843.982) * (-5859.718) [-5832.019] (-5832.786) (-5857.252) -- 9:21:34      Average standard deviation of split frequencies: 0.008864      2196000 -- [-5825.040] (-5834.708) (-5837.310) (-5867.072) * (-5845.706) [-5827.474] (-5835.285) (-5864.797) -- 9:21:29      2197000 -- (-5831.653) (-5833.521) [-5833.620] (-5857.408) * (-5844.856) (-5832.536) [-5826.533] (-5871.344) -- 9:21:23      2198000 -- (-5833.071) [-5819.927] (-5850.505) (-5870.226) * (-5822.713) (-5855.680) (-5820.983) [-5834.288] -- 9:21:18      2199000 -- (-5835.174) [-5822.537] (-5830.218) (-5861.539) * (-5847.695) (-5864.575) (-5819.640) [-5839.997] -- 9:21:16      2200000 -- (-5834.878) [-5820.103] (-5839.475) (-5841.026) * (-5855.239) (-5842.891) (-5822.484) [-5835.098] -- 9:21:11      Average standard deviation of split frequencies: 0.008862      2201000 -- (-5856.097) [-5823.996] (-5832.636) (-5835.515) * (-5857.020) (-5841.072) [-5820.522] (-5837.691) -- 9:21:05      2202000 -- (-5838.370) (-5818.176) (-5856.720) [-5836.186] * (-5862.408) (-5833.899) [-5808.360] (-5839.981) -- 9:21:00      2203000 -- (-5827.681) [-5824.963] (-5874.873) (-5833.919) * (-5870.601) [-5815.893] (-5811.196) (-5843.364) -- 9:20:58      2204000 -- (-5830.169) [-5826.360] (-5843.308) (-5832.511) * (-5854.438) (-5844.846) [-5820.013] (-5843.455) -- 9:20:52      2205000 -- (-5834.287) (-5840.384) (-5857.142) [-5841.201] * (-5867.694) (-5852.682) [-5810.075] (-5843.718) -- 9:20:47      Average standard deviation of split frequencies: 0.008805      2206000 -- [-5831.403] (-5837.502) (-5847.033) (-5850.151) * (-5870.119) (-5847.528) (-5823.253) [-5842.928] -- 9:20:45      2207000 -- (-5833.242) (-5844.395) [-5826.294] (-5856.737) * (-5843.084) (-5828.097) [-5815.682] (-5838.591) -- 9:20:40      2208000 -- (-5836.932) (-5859.529) [-5824.132] (-5859.903) * (-5851.031) (-5830.472) [-5798.705] (-5844.806) -- 9:20:34      2209000 -- (-5842.496) (-5859.127) [-5827.803] (-5859.055) * (-5842.004) [-5816.915] (-5819.082) (-5834.171) -- 9:20:29      2210000 -- [-5832.189] (-5869.148) (-5854.267) (-5857.970) * (-5836.567) (-5818.598) (-5841.137) [-5817.083] -- 9:20:27      Average standard deviation of split frequencies: 0.008873      2211000 -- (-5831.128) (-5859.261) [-5833.531] (-5887.767) * (-5853.160) [-5815.828] (-5847.700) (-5817.209) -- 9:20:21      2212000 -- (-5834.314) (-5827.625) [-5836.689] (-5869.843) * (-5867.380) (-5847.142) (-5848.067) [-5810.439] -- 9:20:16      2213000 -- (-5863.637) (-5839.952) [-5818.864] (-5864.826) * (-5840.462) (-5860.310) (-5835.627) [-5808.228] -- 9:20:14      2214000 -- (-5856.191) (-5841.860) (-5830.781) [-5829.100] * (-5832.323) (-5848.139) (-5839.714) [-5818.267] -- 9:20:09      2215000 -- (-5849.005) [-5830.634] (-5839.045) (-5852.510) * (-5824.567) (-5845.034) (-5837.507) [-5810.561] -- 9:20:03      Average standard deviation of split frequencies: 0.008884      2216000 -- (-5861.312) (-5831.355) [-5829.968] (-5852.249) * (-5823.782) (-5845.263) (-5842.148) [-5812.211] -- 9:19:58      2217000 -- [-5818.881] (-5854.059) (-5838.001) (-5847.901) * [-5825.418] (-5859.827) (-5847.654) (-5833.807) -- 9:19:52      2218000 -- [-5821.891] (-5845.012) (-5815.861) (-5874.965) * [-5813.952] (-5845.293) (-5843.727) (-5838.544) -- 9:19:51      2219000 -- (-5823.114) (-5832.069) [-5823.873] (-5844.929) * [-5820.837] (-5850.400) (-5859.824) (-5831.425) -- 9:19:45      2220000 -- (-5825.052) (-5840.668) [-5830.698] (-5855.504) * [-5821.001] (-5829.141) (-5850.628) (-5830.568) -- 9:19:40      Average standard deviation of split frequencies: 0.008929      2221000 -- (-5850.714) (-5832.734) [-5834.552] (-5848.355) * [-5824.379] (-5837.371) (-5869.339) (-5833.426) -- 9:19:38      2222000 -- [-5833.108] (-5826.553) (-5841.823) (-5846.945) * [-5834.395] (-5835.866) (-5850.043) (-5829.773) -- 9:19:32      2223000 -- [-5826.081] (-5857.524) (-5828.080) (-5835.364) * [-5822.431] (-5826.985) (-5842.315) (-5840.189) -- 9:19:27      2224000 -- (-5813.389) [-5841.245] (-5836.761) (-5843.427) * [-5818.503] (-5830.068) (-5851.181) (-5867.728) -- 9:19:21      2225000 -- [-5818.291] (-5846.818) (-5831.423) (-5850.389) * (-5827.431) [-5829.288] (-5860.230) (-5846.713) -- 9:19:20      Average standard deviation of split frequencies: 0.008857      2226000 -- [-5815.265] (-5817.642) (-5837.596) (-5855.343) * (-5844.986) (-5834.525) (-5854.602) [-5832.504] -- 9:19:14      2227000 -- (-5840.314) [-5817.324] (-5841.732) (-5853.799) * (-5834.198) (-5842.549) (-5860.390) [-5826.992] -- 9:19:09      2228000 -- (-5831.186) [-5828.081] (-5854.340) (-5831.468) * (-5844.254) (-5837.366) (-5865.237) [-5825.061] -- 9:19:03      2229000 -- [-5837.470] (-5837.024) (-5844.649) (-5851.180) * (-5838.219) [-5835.266] (-5838.148) (-5830.538) -- 9:19:01      2230000 -- (-5845.342) [-5828.092] (-5849.872) (-5860.946) * (-5839.997) (-5848.391) [-5833.408] (-5845.021) -- 9:18:56      Average standard deviation of split frequencies: 0.008890      2231000 -- (-5846.765) [-5833.373] (-5847.442) (-5869.172) * (-5842.195) [-5836.998] (-5833.676) (-5867.918) -- 9:18:51      2232000 -- (-5841.687) [-5817.464] (-5824.114) (-5865.926) * [-5829.468] (-5837.133) (-5841.845) (-5859.486) -- 9:18:45      2233000 -- (-5845.609) [-5814.160] (-5835.509) (-5857.217) * (-5846.539) [-5818.132] (-5830.052) (-5854.385) -- 9:18:43      2234000 -- [-5830.807] (-5827.444) (-5845.866) (-5851.811) * (-5834.521) [-5822.054] (-5826.390) (-5857.605) -- 9:18:38      2235000 -- [-5824.211] (-5832.072) (-5857.872) (-5875.366) * (-5869.010) (-5825.428) (-5831.669) [-5823.286] -- 9:18:32      Average standard deviation of split frequencies: 0.008970      2236000 -- (-5831.628) [-5832.609] (-5867.847) (-5850.134) * (-5842.069) [-5831.852] (-5846.188) (-5828.225) -- 9:18:27      2237000 -- (-5836.182) [-5825.367] (-5868.556) (-5858.928) * (-5842.054) [-5820.656] (-5854.646) (-5839.055) -- 9:18:25      2238000 -- (-5836.181) [-5825.868] (-5841.518) (-5838.460) * (-5849.227) [-5829.384] (-5843.707) (-5831.661) -- 9:18:20      2239000 -- [-5836.387] (-5823.681) (-5850.397) (-5844.031) * (-5837.262) [-5832.911] (-5857.731) (-5828.149) -- 9:18:14      2240000 -- (-5827.377) [-5810.845] (-5824.512) (-5853.211) * (-5836.233) (-5841.382) (-5848.236) [-5831.593] -- 9:18:09      Average standard deviation of split frequencies: 0.009059      2241000 -- (-5842.523) [-5817.771] (-5846.364) (-5847.603) * (-5832.895) [-5826.298] (-5840.796) (-5838.442) -- 9:18:07      2242000 -- [-5838.211] (-5827.035) (-5884.149) (-5856.075) * [-5820.374] (-5836.027) (-5849.406) (-5845.553) -- 9:18:01      2243000 -- (-5816.113) [-5823.783] (-5860.979) (-5857.298) * [-5828.352] (-5850.059) (-5850.297) (-5840.910) -- 9:17:56      2244000 -- [-5828.408] (-5837.998) (-5835.582) (-5860.785) * (-5844.239) (-5873.725) (-5845.109) [-5821.480] -- 9:17:51      2245000 -- (-5852.810) [-5824.888] (-5837.031) (-5852.889) * (-5859.433) (-5855.287) (-5845.599) [-5837.742] -- 9:17:49      Average standard deviation of split frequencies: 0.009057      2246000 -- (-5844.879) [-5829.582] (-5830.824) (-5838.696) * [-5841.518] (-5847.526) (-5852.961) (-5830.897) -- 9:17:43      2247000 -- (-5856.596) [-5814.509] (-5842.825) (-5866.755) * (-5846.847) [-5821.020] (-5846.533) (-5852.905) -- 9:17:38      2248000 -- (-5831.556) [-5823.782] (-5832.237) (-5859.613) * (-5843.463) [-5828.783] (-5831.479) (-5856.353) -- 9:17:32      2249000 -- (-5821.218) [-5810.849] (-5841.909) (-5847.339) * (-5839.902) (-5817.278) [-5844.149] (-5851.113) -- 9:17:30      2250000 -- [-5823.891] (-5831.411) (-5846.033) (-5844.503) * [-5827.462] (-5834.826) (-5854.108) (-5853.550) -- 9:17:25      Average standard deviation of split frequencies: 0.009096      2251000 -- (-5839.634) (-5840.915) [-5828.500] (-5854.706) * (-5823.908) [-5808.098] (-5842.342) (-5870.611) -- 9:17:20      2252000 -- [-5827.987] (-5838.928) (-5836.818) (-5848.854) * (-5828.711) [-5809.224] (-5860.077) (-5859.513) -- 9:17:14      2253000 -- [-5835.248] (-5851.864) (-5838.710) (-5838.846) * [-5831.091] (-5816.051) (-5837.090) (-5865.768) -- 9:17:12      2254000 -- [-5827.177] (-5847.200) (-5830.069) (-5836.762) * (-5841.621) [-5817.790] (-5826.096) (-5875.475) -- 9:17:07      2255000 -- [-5822.916] (-5836.667) (-5829.029) (-5855.320) * [-5820.966] (-5830.661) (-5829.201) (-5870.846) -- 9:17:01      Average standard deviation of split frequencies: 0.009171      2256000 -- (-5833.820) [-5827.595] (-5813.307) (-5855.382) * (-5841.873) [-5818.045] (-5841.589) (-5856.480) -- 9:16:56      2257000 -- (-5830.841) (-5822.499) [-5808.926] (-5875.981) * (-5817.119) [-5818.764] (-5825.842) (-5864.075) -- 9:16:54      2258000 -- (-5835.173) (-5837.374) [-5822.065] (-5861.600) * [-5816.011] (-5828.429) (-5830.275) (-5848.456) -- 9:16:49      2259000 -- [-5831.764] (-5844.703) (-5845.257) (-5852.078) * [-5823.751] (-5834.286) (-5826.495) (-5852.624) -- 9:16:43      2260000 -- (-5833.661) (-5814.053) [-5819.719] (-5851.816) * (-5836.938) (-5868.767) [-5830.783] (-5855.189) -- 9:16:38      Average standard deviation of split frequencies: 0.009161      2261000 -- (-5839.022) [-5812.785] (-5846.598) (-5838.993) * (-5833.321) (-5859.561) [-5815.347] (-5842.215) -- 9:16:36      2262000 -- (-5828.479) [-5836.601] (-5852.766) (-5860.992) * [-5825.621] (-5857.120) (-5828.007) (-5842.623) -- 9:16:31      2263000 -- [-5821.651] (-5857.665) (-5853.872) (-5841.362) * (-5840.079) (-5842.911) [-5815.338] (-5846.732) -- 9:16:25      2264000 -- [-5822.005] (-5848.444) (-5857.328) (-5840.917) * (-5863.433) (-5854.072) [-5812.596] (-5850.671) -- 9:16:23      2265000 -- [-5818.235] (-5863.898) (-5835.038) (-5845.828) * (-5859.386) (-5834.110) [-5819.678] (-5841.646) -- 9:16:18      Average standard deviation of split frequencies: 0.009196      2266000 -- (-5826.929) (-5851.857) [-5838.449] (-5848.813) * (-5859.287) (-5864.798) [-5818.544] (-5850.927) -- 9:16:12      2267000 -- [-5818.352] (-5842.271) (-5824.048) (-5844.330) * (-5857.739) (-5856.481) (-5825.656) [-5832.526] -- 9:16:10      2268000 -- (-5830.059) [-5818.405] (-5853.246) (-5853.743) * (-5835.585) (-5857.013) [-5831.891] (-5841.282) -- 9:16:05      2269000 -- [-5826.410] (-5838.375) (-5828.747) (-5863.525) * (-5833.707) [-5835.772] (-5827.962) (-5843.306) -- 9:16:00      2270000 -- (-5823.779) (-5835.305) [-5831.247] (-5852.194) * (-5822.962) (-5857.670) [-5814.431] (-5838.592) -- 9:15:58      Average standard deviation of split frequencies: 0.009216      2271000 -- [-5820.960] (-5843.801) (-5813.724) (-5864.481) * (-5831.665) (-5831.531) [-5819.729] (-5832.071) -- 9:15:52      2272000 -- (-5844.641) (-5823.394) [-5818.950] (-5866.901) * (-5827.584) (-5856.678) (-5837.865) [-5829.288] -- 9:15:47      2273000 -- (-5834.930) [-5834.167] (-5822.241) (-5837.227) * [-5829.641] (-5865.585) (-5841.362) (-5844.294) -- 9:15:45      2274000 -- (-5835.384) (-5832.575) [-5821.067] (-5843.490) * (-5816.320) (-5886.498) [-5817.193] (-5848.460) -- 9:15:40      2275000 -- (-5837.719) (-5837.799) [-5829.210] (-5841.753) * [-5827.335] (-5859.241) (-5862.112) (-5856.507) -- 9:15:34      Average standard deviation of split frequencies: 0.009343      2276000 -- [-5819.403] (-5827.341) (-5840.260) (-5866.872) * (-5838.772) (-5858.365) (-5869.092) [-5828.639] -- 9:15:29      2277000 -- [-5826.754] (-5831.531) (-5839.317) (-5854.424) * [-5831.298] (-5880.031) (-5852.791) (-5828.699) -- 9:15:27      2278000 -- [-5825.919] (-5826.390) (-5827.179) (-5850.713) * [-5826.286] (-5876.789) (-5839.951) (-5833.300) -- 9:15:21      2279000 -- (-5824.731) [-5822.199] (-5823.101) (-5860.650) * [-5833.480] (-5857.349) (-5833.277) (-5840.221) -- 9:15:16      2280000 -- (-5838.773) [-5820.954] (-5835.072) (-5857.925) * [-5818.652] (-5857.856) (-5854.343) (-5832.620) -- 9:15:14      Average standard deviation of split frequencies: 0.009377      2281000 -- (-5861.109) [-5816.320] (-5825.806) (-5839.694) * (-5822.243) (-5855.452) [-5838.473] (-5831.472) -- 9:15:09      2282000 -- (-5853.667) [-5817.106] (-5824.174) (-5853.634) * (-5827.350) (-5848.870) (-5844.659) [-5825.194] -- 9:15:03      2283000 -- [-5836.135] (-5827.056) (-5843.445) (-5846.138) * (-5835.822) (-5842.259) (-5831.938) [-5824.474] -- 9:14:58      2284000 -- (-5829.357) [-5833.085] (-5838.309) (-5844.668) * (-5842.312) (-5854.024) (-5832.935) [-5843.763] -- 9:14:56      2285000 -- (-5860.454) (-5852.232) [-5830.824] (-5853.481) * (-5843.707) (-5857.960) (-5832.667) [-5824.016] -- 9:14:50      Average standard deviation of split frequencies: 0.009461      2286000 -- [-5833.192] (-5839.766) (-5854.696) (-5839.734) * (-5864.786) (-5832.842) (-5851.993) [-5818.500] -- 9:14:45      2287000 -- [-5821.589] (-5853.138) (-5834.888) (-5845.454) * (-5882.457) (-5845.761) (-5864.266) [-5822.126] -- 9:14:40      2288000 -- [-5831.166] (-5853.304) (-5839.347) (-5836.750) * (-5862.888) (-5834.826) (-5836.921) [-5822.870] -- 9:14:38      2289000 -- (-5827.806) (-5855.902) [-5823.982] (-5845.371) * (-5854.950) (-5819.271) (-5838.262) [-5822.791] -- 9:14:32      2290000 -- [-5814.190] (-5835.237) (-5839.457) (-5872.854) * (-5854.668) (-5832.645) [-5833.636] (-5832.164) -- 9:14:27      Average standard deviation of split frequencies: 0.009457      2291000 -- [-5811.655] (-5829.802) (-5853.855) (-5874.366) * (-5844.787) [-5829.378] (-5838.990) (-5839.184) -- 9:14:22      2292000 -- [-5818.356] (-5841.625) (-5882.558) (-5846.464) * (-5866.471) [-5822.072] (-5828.022) (-5847.489) -- 9:14:20      2293000 -- [-5831.688] (-5828.752) (-5837.562) (-5853.642) * (-5851.792) (-5829.342) [-5833.204] (-5835.364) -- 9:14:14      2294000 -- [-5826.222] (-5845.363) (-5830.781) (-5846.005) * (-5821.839) [-5812.168] (-5857.182) (-5832.191) -- 9:14:09      2295000 -- (-5830.320) (-5841.970) [-5815.087] (-5826.482) * (-5827.246) [-5820.586] (-5851.044) (-5838.947) -- 9:14:07      Average standard deviation of split frequencies: 0.009538      2296000 -- (-5838.894) [-5838.129] (-5833.650) (-5838.500) * (-5826.597) [-5817.412] (-5835.926) (-5852.707) -- 9:14:01      2297000 -- (-5858.573) (-5838.583) [-5815.599] (-5858.516) * (-5844.930) [-5809.935] (-5845.873) (-5835.399) -- 9:13:56      2298000 -- (-5840.555) [-5823.812] (-5835.802) (-5857.288) * (-5845.657) (-5826.162) (-5846.474) [-5821.390] -- 9:13:51      2299000 -- (-5832.620) [-5823.517] (-5845.083) (-5862.680) * (-5836.318) [-5811.653] (-5833.579) (-5840.411) -- 9:13:49      2300000 -- (-5829.965) [-5822.410] (-5833.518) (-5856.584) * (-5830.994) [-5811.625] (-5860.888) (-5853.175) -- 9:13:43      Average standard deviation of split frequencies: 0.009408      2301000 -- [-5821.413] (-5848.009) (-5838.440) (-5861.265) * (-5846.234) [-5819.002] (-5853.649) (-5847.345) -- 9:13:38      2302000 -- [-5827.083] (-5848.994) (-5866.768) (-5862.223) * (-5833.482) [-5818.101] (-5844.294) (-5844.811) -- 9:13:36      2303000 -- [-5816.992] (-5833.463) (-5847.360) (-5852.813) * (-5840.840) [-5824.340] (-5845.383) (-5840.325) -- 9:13:31      2304000 -- [-5803.855] (-5821.176) (-5854.665) (-5863.424) * (-5835.178) [-5842.758] (-5842.255) (-5865.903) -- 9:13:25      2305000 -- (-5808.005) [-5827.638] (-5866.805) (-5850.497) * (-5844.712) (-5861.395) [-5833.186] (-5836.101) -- 9:13:20      Average standard deviation of split frequencies: 0.009457      2306000 -- [-5825.350] (-5837.822) (-5848.275) (-5832.645) * (-5833.261) (-5856.705) [-5829.256] (-5844.989) -- 9:13:18      2307000 -- (-5845.930) (-5850.080) [-5840.247] (-5843.236) * (-5833.819) (-5851.558) (-5845.255) [-5836.219] -- 9:13:12      2308000 -- [-5829.564] (-5844.719) (-5854.051) (-5838.632) * (-5827.071) (-5848.516) (-5849.737) [-5833.448] -- 9:13:07      2309000 -- [-5820.653] (-5881.121) (-5836.601) (-5859.166) * (-5827.775) (-5853.688) (-5844.417) [-5833.948] -- 9:13:02      2310000 -- (-5831.202) (-5859.153) [-5826.151] (-5857.479) * (-5835.872) (-5859.781) (-5844.400) [-5816.939] -- 9:13:00      Average standard deviation of split frequencies: 0.009490      2311000 -- [-5828.534] (-5873.320) (-5820.902) (-5844.865) * (-5838.797) (-5839.603) (-5875.756) [-5829.597] -- 9:12:54      2312000 -- (-5837.743) (-5865.926) [-5825.385] (-5832.488) * (-5819.531) (-5850.771) (-5871.170) [-5822.153] -- 9:12:49      2313000 -- (-5842.499) (-5833.739) [-5832.572] (-5851.844) * (-5815.736) (-5842.705) (-5833.902) [-5841.259] -- 9:12:44      2314000 -- (-5825.278) (-5836.960) [-5818.359] (-5878.128) * [-5811.738] (-5846.579) (-5845.158) (-5829.141) -- 9:12:38      2315000 -- (-5835.987) (-5840.972) [-5814.782] (-5869.884) * (-5822.858) (-5851.733) [-5827.615] (-5837.581) -- 9:12:36      Average standard deviation of split frequencies: 0.009548      2316000 -- (-5852.005) [-5843.781] (-5824.539) (-5858.525) * (-5841.290) (-5861.302) (-5830.990) [-5823.769] -- 9:12:31      2317000 -- (-5842.121) (-5830.392) [-5820.658] (-5853.324) * [-5844.316] (-5841.009) (-5851.958) (-5821.644) -- 9:12:25      2318000 -- (-5843.122) (-5832.274) [-5821.554] (-5858.809) * (-5850.544) [-5835.776] (-5852.355) (-5836.083) -- 9:12:20      2319000 -- (-5836.224) (-5836.306) [-5819.944] (-5875.103) * (-5836.048) [-5819.724] (-5859.149) (-5832.987) -- 9:12:18      2320000 -- (-5837.030) (-5830.013) [-5820.899] (-5848.289) * [-5829.922] (-5833.950) (-5849.286) (-5811.913) -- 9:12:13      Average standard deviation of split frequencies: 0.009615      2321000 -- (-5839.253) [-5831.982] (-5827.659) (-5857.201) * (-5832.544) (-5848.975) (-5850.978) [-5828.250] -- 9:12:07      2322000 -- (-5846.101) (-5832.943) [-5821.474] (-5861.463) * [-5837.669] (-5855.206) (-5860.974) (-5822.609) -- 9:12:02      2323000 -- (-5837.203) (-5822.941) [-5823.259] (-5859.861) * [-5824.466] (-5830.297) (-5842.632) (-5845.488) -- 9:12:00      2324000 -- [-5819.337] (-5831.817) (-5858.028) (-5850.849) * [-5825.745] (-5837.799) (-5844.444) (-5846.326) -- 9:11:55      2325000 -- [-5822.324] (-5822.766) (-5847.584) (-5840.301) * [-5821.092] (-5849.010) (-5833.206) (-5829.547) -- 9:11:49      Average standard deviation of split frequencies: 0.009655      2326000 -- (-5843.308) (-5818.147) [-5834.479] (-5838.391) * [-5813.910] (-5862.952) (-5841.875) (-5837.971) -- 9:11:44      2327000 -- (-5851.986) (-5832.934) (-5853.321) [-5822.761] * [-5819.972] (-5852.619) (-5834.291) (-5836.749) -- 9:11:42      2328000 -- (-5840.402) [-5829.074] (-5854.848) (-5831.877) * [-5829.143] (-5861.301) (-5829.105) (-5830.545) -- 9:11:37      2329000 -- (-5846.489) [-5827.397] (-5859.810) (-5837.423) * (-5816.800) (-5841.240) (-5843.222) [-5830.086] -- 9:11:31      2330000 -- (-5843.119) [-5812.762] (-5843.162) (-5840.416) * (-5824.311) (-5825.695) [-5837.093] (-5856.098) -- 9:11:26      Average standard deviation of split frequencies: 0.009563      2331000 -- (-5845.452) (-5829.783) (-5831.891) [-5824.494] * [-5821.041] (-5840.310) (-5837.026) (-5835.684) -- 9:11:24      2332000 -- (-5838.256) (-5836.203) (-5838.887) [-5826.227] * (-5837.457) (-5870.688) (-5868.544) [-5828.858] -- 9:11:18      2333000 -- (-5838.964) (-5824.965) (-5828.077) [-5835.576] * [-5839.940] (-5855.722) (-5871.212) (-5863.588) -- 9:11:13      2334000 -- (-5842.585) [-5823.440] (-5835.116) (-5842.015) * (-5835.918) (-5868.375) (-5865.730) [-5838.567] -- 9:11:08      2335000 -- (-5871.731) [-5819.997] (-5820.292) (-5845.709) * [-5831.575] (-5863.521) (-5843.307) (-5833.831) -- 9:11:02      Average standard deviation of split frequencies: 0.009668      2336000 -- (-5856.357) (-5834.837) [-5837.895] (-5837.104) * [-5826.932] (-5861.088) (-5835.628) (-5834.079) -- 9:11:00      2337000 -- (-5845.652) [-5814.582] (-5835.258) (-5852.349) * [-5819.378] (-5855.764) (-5849.897) (-5841.854) -- 9:10:55      2338000 -- (-5855.409) [-5811.520] (-5838.857) (-5850.440) * [-5805.590] (-5830.007) (-5851.474) (-5838.603) -- 9:10:50      2339000 -- (-5857.369) [-5820.748] (-5845.715) (-5847.338) * [-5804.943] (-5828.450) (-5851.175) (-5827.344) -- 9:10:48      2340000 -- (-5883.270) (-5829.553) [-5848.208] (-5843.567) * (-5817.878) [-5811.286] (-5846.142) (-5837.556) -- 9:10:42      Average standard deviation of split frequencies: 0.009712      2341000 -- (-5867.980) [-5824.541] (-5843.155) (-5833.105) * [-5807.645] (-5824.385) (-5828.138) (-5846.512) -- 9:10:37      2342000 -- (-5860.070) [-5816.778] (-5856.943) (-5843.704) * [-5813.661] (-5840.509) (-5820.528) (-5868.311) -- 9:10:32      2343000 -- (-5866.456) [-5840.873] (-5858.627) (-5841.292) * (-5826.094) (-5830.344) [-5823.942] (-5846.311) -- 9:10:30      2344000 -- (-5866.282) [-5833.034] (-5827.049) (-5843.835) * (-5821.785) (-5847.757) [-5822.501] (-5842.441) -- 9:10:24      2345000 -- (-5860.189) (-5837.996) [-5820.085] (-5831.050) * (-5833.172) (-5841.327) (-5836.417) [-5825.377] -- 9:10:22      Average standard deviation of split frequencies: 0.009862      2346000 -- (-5858.987) (-5836.852) [-5823.037] (-5835.418) * (-5835.919) (-5861.407) [-5822.165] (-5822.098) -- 9:10:17      2347000 -- (-5853.550) (-5849.745) (-5819.408) [-5826.170] * (-5853.461) (-5852.638) (-5830.308) [-5817.474] -- 9:10:11      2348000 -- (-5843.976) (-5838.814) [-5822.696] (-5828.690) * [-5841.581] (-5863.697) (-5844.127) (-5835.955) -- 9:10:06      2349000 -- (-5868.618) (-5860.126) (-5828.744) [-5830.480] * [-5836.320] (-5864.946) (-5833.638) (-5832.518) -- 9:10:04      2350000 -- (-5839.848) (-5826.406) [-5819.637] (-5823.101) * [-5834.587] (-5831.494) (-5836.794) (-5852.245) -- 9:09:59      Average standard deviation of split frequencies: 0.010007      2351000 -- (-5835.973) [-5826.628] (-5845.659) (-5837.088) * [-5828.838] (-5849.197) (-5833.726) (-5855.405) -- 9:09:53      2352000 -- (-5841.530) (-5832.130) (-5849.873) [-5824.175] * (-5857.575) (-5855.032) [-5828.845] (-5856.990) -- 9:09:48      2353000 -- (-5820.406) (-5848.747) [-5829.099] (-5834.557) * [-5839.547] (-5848.611) (-5814.971) (-5860.614) -- 9:09:46      2354000 -- (-5833.571) (-5855.121) [-5821.345] (-5839.429) * (-5851.827) (-5836.075) [-5823.590] (-5834.605) -- 9:09:41      2355000 -- (-5842.788) (-5833.232) [-5815.209] (-5854.403) * (-5852.935) (-5825.186) (-5839.127) [-5829.905] -- 9:09:35      Average standard deviation of split frequencies: 0.010033      2356000 -- [-5831.306] (-5846.968) (-5833.215) (-5857.982) * (-5846.981) [-5816.093] (-5826.046) (-5840.367) -- 9:09:33      2357000 -- (-5839.767) (-5832.814) [-5812.863] (-5867.676) * (-5861.441) (-5830.051) [-5823.810] (-5832.032) -- 9:09:28      2358000 -- (-5829.325) (-5843.540) [-5809.614] (-5862.335) * (-5842.226) (-5839.568) [-5829.138] (-5858.158) -- 9:09:23      2359000 -- [-5811.499] (-5845.079) (-5825.708) (-5865.276) * (-5841.317) [-5818.195] (-5855.778) (-5885.338) -- 9:09:17      2360000 -- [-5829.401] (-5838.641) (-5843.831) (-5851.872) * (-5836.574) [-5831.773] (-5850.619) (-5858.144) -- 9:09:12      Average standard deviation of split frequencies: 0.009960      2361000 -- (-5822.372) (-5850.697) [-5830.931] (-5834.758) * (-5869.308) [-5829.767] (-5824.986) (-5846.793) -- 9:09:10      2362000 -- (-5831.880) (-5865.196) (-5827.357) [-5836.203] * (-5864.027) (-5831.199) [-5809.933] (-5837.557) -- 9:09:04      2363000 -- (-5840.243) (-5843.723) [-5827.967] (-5851.324) * (-5858.100) (-5818.484) [-5827.812] (-5842.402) -- 9:08:59      2364000 -- (-5837.481) (-5846.197) [-5834.738] (-5841.293) * (-5852.682) [-5838.375] (-5840.029) (-5848.958) -- 9:08:54      2365000 -- [-5821.014] (-5860.490) (-5841.100) (-5842.241) * (-5857.330) [-5838.644] (-5836.071) (-5834.289) -- 9:08:52      Average standard deviation of split frequencies: 0.009822      2366000 -- (-5831.235) (-5857.141) (-5850.251) [-5827.253] * (-5825.011) (-5850.911) [-5839.640] (-5856.438) -- 9:08:46      2367000 -- [-5825.411] (-5831.588) (-5853.660) (-5834.856) * [-5824.522] (-5845.410) (-5829.938) (-5836.810) -- 9:08:41      2368000 -- (-5849.810) [-5822.870] (-5853.560) (-5830.406) * [-5822.250] (-5858.247) (-5851.141) (-5854.495) -- 9:08:36      2369000 -- (-5859.533) (-5823.208) (-5852.531) [-5814.205] * [-5826.489] (-5861.252) (-5831.366) (-5850.639) -- 9:08:34      2370000 -- (-5854.116) (-5825.802) (-5847.014) [-5819.588] * [-5819.964] (-5839.689) (-5830.429) (-5848.715) -- 9:08:28      Average standard deviation of split frequencies: 0.009890      2371000 -- (-5852.121) (-5835.042) (-5849.716) [-5817.608] * (-5822.144) (-5834.968) (-5852.637) [-5833.718] -- 9:08:23      2372000 -- (-5842.207) (-5828.188) (-5863.518) [-5821.275] * (-5820.918) (-5839.447) (-5867.619) [-5833.837] -- 9:08:21      2373000 -- (-5861.378) (-5854.232) (-5844.372) [-5831.619] * [-5827.338] (-5840.871) (-5839.882) (-5826.526) -- 9:08:16      2374000 -- [-5835.809] (-5835.574) (-5854.023) (-5832.973) * (-5829.722) [-5826.164] (-5839.275) (-5845.297) -- 9:08:10      2375000 -- (-5846.242) (-5846.780) (-5855.538) [-5824.906] * [-5825.276] (-5844.713) (-5832.152) (-5837.749) -- 9:08:05      Average standard deviation of split frequencies: 0.009859      2376000 -- (-5834.512) (-5841.696) (-5854.474) [-5812.903] * (-5832.485) (-5848.606) [-5821.318] (-5843.167) -- 9:08:03      2377000 -- (-5842.876) (-5842.068) (-5876.870) [-5805.786] * (-5825.490) (-5828.222) [-5825.393] (-5843.864) -- 9:07:57      2378000 -- (-5849.697) (-5841.608) (-5856.178) [-5817.935] * (-5832.992) (-5808.366) [-5814.330] (-5863.871) -- 9:07:52      2379000 -- (-5847.838) (-5831.153) (-5843.250) [-5823.494] * (-5854.203) [-5814.788] (-5832.647) (-5857.184) -- 9:07:50      2380000 -- (-5825.532) (-5848.838) (-5854.081) [-5817.553] * [-5828.548] (-5841.166) (-5820.132) (-5872.044) -- 9:07:45      Average standard deviation of split frequencies: 0.009826      2381000 -- (-5850.313) (-5840.222) (-5849.087) [-5822.095] * [-5827.110] (-5852.514) (-5831.767) (-5844.946) -- 9:07:39      2382000 -- (-5849.849) (-5835.293) (-5857.229) [-5831.186] * (-5837.500) (-5847.877) [-5839.739] (-5865.186) -- 9:07:37      2383000 -- (-5834.740) (-5839.438) (-5856.370) [-5818.900] * (-5829.261) [-5829.660] (-5838.316) (-5852.702) -- 9:07:32      2384000 -- (-5828.061) (-5840.845) (-5866.380) [-5819.413] * (-5835.649) (-5855.952) [-5841.972] (-5838.443) -- 9:07:27      2385000 -- [-5825.296] (-5814.672) (-5856.446) (-5823.775) * [-5827.944] (-5853.307) (-5842.340) (-5856.883) -- 9:07:21      Average standard deviation of split frequencies: 0.009854      2386000 -- (-5825.183) [-5821.887] (-5839.542) (-5849.422) * (-5837.377) (-5844.455) [-5830.843] (-5840.390) -- 9:07:16      2387000 -- (-5844.148) [-5821.428] (-5843.562) (-5867.891) * (-5846.916) (-5850.052) [-5819.190] (-5847.657) -- 9:07:11      2388000 -- (-5863.537) [-5820.365] (-5827.230) (-5854.828) * (-5840.031) (-5844.788) [-5833.316] (-5836.886) -- 9:07:09      2389000 -- (-5853.857) (-5834.593) [-5827.373] (-5865.708) * (-5865.274) (-5848.222) [-5821.315] (-5841.437) -- 9:07:03      2390000 -- (-5844.614) [-5823.287] (-5839.867) (-5858.007) * (-5842.811) (-5841.322) [-5824.462] (-5832.249) -- 9:06:58      Average standard deviation of split frequencies: 0.009771      2391000 -- (-5850.920) [-5822.470] (-5820.567) (-5854.283) * (-5875.912) (-5843.624) [-5837.758] (-5825.524) -- 9:06:56      2392000 -- (-5841.478) (-5831.328) [-5826.322] (-5844.121) * (-5850.509) (-5866.054) (-5833.740) [-5813.750] -- 9:06:51      2393000 -- [-5832.667] (-5845.255) (-5828.356) (-5850.933) * (-5852.583) (-5866.039) (-5826.899) [-5819.512] -- 9:06:45      2394000 -- (-5832.767) (-5841.729) [-5826.455] (-5848.352) * [-5843.909] (-5852.860) (-5832.869) (-5848.462) -- 9:06:40      2395000 -- (-5839.379) (-5843.664) [-5833.609] (-5833.224) * [-5825.059] (-5849.331) (-5835.556) (-5825.856) -- 9:06:35      Average standard deviation of split frequencies: 0.009698      2396000 -- (-5853.701) (-5851.162) [-5834.185] (-5853.727) * (-5829.793) (-5858.166) [-5827.365] (-5842.257) -- 9:06:33      2397000 -- (-5831.803) (-5844.271) [-5826.800] (-5840.130) * (-5821.062) (-5849.055) [-5818.001] (-5863.743) -- 9:06:27      2398000 -- (-5834.463) (-5848.302) [-5818.742] (-5856.860) * [-5821.064] (-5866.413) (-5828.972) (-5845.882) -- 9:06:22      2399000 -- (-5827.842) (-5853.634) [-5822.937] (-5843.834) * (-5845.630) (-5851.052) [-5820.820] (-5823.088) -- 9:06:20      2400000 -- (-5855.381) (-5845.226) (-5821.263) [-5835.516] * (-5858.893) (-5839.751) [-5822.693] (-5833.313) -- 9:06:15      Average standard deviation of split frequencies: 0.009612      2401000 -- (-5839.876) [-5827.420] (-5851.056) (-5839.457) * (-5857.572) (-5845.817) [-5815.328] (-5834.257) -- 9:06:09      2402000 -- (-5839.313) (-5835.454) [-5849.367] (-5847.818) * (-5843.039) (-5857.628) [-5816.695] (-5844.444) -- 9:06:07      2403000 -- (-5848.278) [-5819.321] (-5873.448) (-5868.543) * (-5843.154) (-5831.405) [-5822.082] (-5842.962) -- 9:06:02      2404000 -- (-5841.139) [-5832.138] (-5847.923) (-5857.196) * (-5854.708) (-5815.287) [-5824.280] (-5854.193) -- 9:05:56      2405000 -- (-5865.945) [-5831.815] (-5848.289) (-5843.392) * (-5845.976) (-5841.410) [-5828.011] (-5859.864) -- 9:05:51      Average standard deviation of split frequencies: 0.009616      2406000 -- (-5846.075) [-5819.987] (-5875.656) (-5860.933) * (-5825.679) (-5836.109) [-5837.266] (-5843.133) -- 9:05:49      2407000 -- (-5826.604) [-5829.408] (-5874.430) (-5852.343) * (-5832.577) (-5869.249) [-5828.358] (-5845.164) -- 9:05:44      2408000 -- (-5836.959) (-5843.340) (-5871.149) [-5834.324] * [-5825.706] (-5841.687) (-5846.903) (-5840.968) -- 9:05:38      2409000 -- (-5836.170) [-5835.215] (-5847.517) (-5836.281) * (-5848.628) [-5820.155] (-5830.288) (-5865.347) -- 9:05:33      2410000 -- (-5821.885) (-5840.651) (-5872.992) [-5821.453] * (-5836.059) [-5817.947] (-5818.397) (-5837.960) -- 9:05:31      Average standard deviation of split frequencies: 0.009580      2411000 -- (-5820.705) (-5841.339) (-5877.885) [-5818.971] * (-5844.552) (-5814.532) [-5813.922] (-5843.537) -- 9:05:26      2412000 -- (-5833.030) (-5839.689) (-5837.942) [-5808.764] * (-5859.848) (-5819.162) [-5822.609] (-5843.256) -- 9:05:20      2413000 -- (-5846.633) (-5858.350) (-5835.163) [-5808.036] * (-5865.715) [-5827.195] (-5844.465) (-5845.098) -- 9:05:15      2414000 -- (-5850.374) (-5841.832) (-5834.127) [-5813.144] * (-5853.031) (-5831.212) [-5842.965] (-5838.231) -- 9:05:13      2415000 -- (-5838.055) (-5845.750) (-5840.582) [-5808.302] * (-5877.560) [-5833.522] (-5842.592) (-5848.970) -- 9:05:08      Average standard deviation of split frequencies: 0.009696      2416000 -- (-5843.022) (-5856.613) (-5846.232) [-5824.521] * (-5872.459) [-5841.686] (-5852.917) (-5839.247) -- 9:05:02      2417000 -- (-5851.324) [-5837.840] (-5841.580) (-5838.488) * (-5853.839) (-5852.202) (-5861.876) [-5826.484] -- 9:04:57      2418000 -- [-5837.763] (-5828.338) (-5853.222) (-5844.351) * (-5822.732) [-5822.260] (-5832.978) (-5839.276) -- 9:04:55      2419000 -- [-5828.003] (-5837.948) (-5843.014) (-5853.430) * (-5850.630) [-5832.784] (-5856.529) (-5834.489) -- 9:04:50      2420000 -- (-5850.224) [-5828.126] (-5837.404) (-5850.082) * (-5838.048) (-5833.077) (-5856.346) [-5822.311] -- 9:04:44      Average standard deviation of split frequencies: 0.009720      2421000 -- (-5842.380) [-5821.749] (-5828.830) (-5840.861) * (-5841.169) (-5831.305) (-5857.047) [-5815.523] -- 9:04:42      2422000 -- [-5830.912] (-5849.435) (-5856.953) (-5838.015) * (-5832.204) (-5848.906) (-5832.455) [-5827.145] -- 9:04:37      2423000 -- [-5837.887] (-5852.095) (-5852.601) (-5829.181) * [-5828.075] (-5853.429) (-5845.235) (-5851.572) -- 9:04:32      2424000 -- [-5825.321] (-5835.737) (-5856.821) (-5843.644) * (-5833.641) (-5823.829) [-5830.223] (-5839.888) -- 9:04:26      2425000 -- (-5836.092) (-5837.864) [-5838.249] (-5846.394) * (-5834.955) (-5836.691) (-5863.522) [-5821.674] -- 9:04:24      Average standard deviation of split frequencies: 0.009731      2426000 -- [-5843.856] (-5851.180) (-5856.529) (-5836.440) * (-5855.884) (-5843.897) (-5830.927) [-5808.507] -- 9:04:19      2427000 -- (-5832.623) (-5853.142) (-5855.042) [-5821.273] * (-5852.401) (-5840.068) [-5824.452] (-5845.244) -- 9:04:14      2428000 -- [-5831.246] (-5853.557) (-5842.624) (-5837.760) * (-5844.465) (-5840.691) [-5822.471] (-5835.997) -- 9:04:08      2429000 -- (-5821.167) (-5853.215) [-5829.575] (-5839.871) * (-5830.463) (-5836.115) [-5841.043] (-5842.932) -- 9:04:06      2430000 -- [-5826.133] (-5862.791) (-5860.583) (-5847.304) * (-5843.340) [-5810.184] (-5852.342) (-5841.920) -- 9:04:01      Average standard deviation of split frequencies: 0.009871      2431000 -- (-5838.319) (-5882.915) (-5851.654) [-5835.358] * (-5835.928) [-5839.262] (-5840.454) (-5868.351) -- 9:03:56      2432000 -- (-5851.335) (-5835.803) [-5824.422] (-5843.772) * (-5838.016) (-5846.227) [-5828.688] (-5855.016) -- 9:03:50      2433000 -- (-5850.025) [-5832.249] (-5835.111) (-5840.147) * (-5846.339) [-5826.046] (-5844.536) (-5853.487) -- 9:03:48      2434000 -- (-5867.226) (-5848.552) (-5831.891) [-5838.651] * (-5850.219) [-5833.413] (-5838.077) (-5855.151) -- 9:03:43      2435000 -- (-5861.442) (-5847.115) (-5852.064) [-5844.808] * (-5860.580) (-5828.514) (-5858.596) [-5838.521] -- 9:03:38      Average standard deviation of split frequencies: 0.009887      2436000 -- (-5848.254) (-5857.055) [-5835.240] (-5820.631) * (-5855.465) (-5840.591) [-5841.159] (-5848.879) -- 9:03:35      2437000 -- (-5821.242) (-5856.071) (-5855.353) [-5832.962] * (-5858.968) [-5830.334] (-5856.267) (-5827.204) -- 9:03:30      2438000 -- (-5821.110) [-5835.359] (-5846.853) (-5852.658) * (-5844.150) (-5839.595) (-5841.045) [-5831.476] -- 9:03:25      2439000 -- [-5828.414] (-5836.272) (-5840.601) (-5839.961) * (-5858.626) (-5866.309) (-5844.655) [-5825.890] -- 9:03:20      2440000 -- (-5825.752) (-5844.996) (-5852.057) [-5817.347] * (-5838.076) (-5861.738) [-5829.056] (-5866.456) -- 9:03:17      Average standard deviation of split frequencies: 0.009925      2441000 -- (-5829.725) (-5852.495) (-5864.142) [-5824.980] * (-5852.645) (-5850.527) [-5830.603] (-5845.562) -- 9:03:12      2442000 -- (-5829.453) (-5854.915) (-5863.464) [-5828.490] * (-5866.553) (-5844.478) [-5843.170] (-5841.480) -- 9:03:07      2443000 -- (-5830.002) (-5862.907) (-5874.234) [-5820.444] * [-5849.874] (-5863.608) (-5848.881) (-5837.146) -- 9:03:05      2444000 -- [-5822.954] (-5870.792) (-5836.413) (-5827.233) * (-5850.451) (-5838.691) (-5838.092) [-5837.859] -- 9:02:59      2445000 -- (-5834.223) (-5885.245) [-5812.516] (-5846.211) * (-5841.374) [-5827.982] (-5854.728) (-5829.933) -- 9:02:54      Average standard deviation of split frequencies: 0.009907      2446000 -- [-5827.972] (-5901.326) (-5810.686) (-5850.413) * (-5832.231) [-5822.161] (-5857.711) (-5854.111) -- 9:02:52      2447000 -- (-5820.585) (-5860.308) [-5823.647] (-5848.421) * (-5853.032) [-5814.761] (-5850.229) (-5831.091) -- 9:02:47      2448000 -- [-5838.488] (-5876.726) (-5833.838) (-5848.351) * (-5838.696) [-5827.090] (-5865.151) (-5857.338) -- 9:02:41      2449000 -- [-5826.709] (-5877.074) (-5829.052) (-5849.422) * (-5839.220) [-5818.752] (-5864.366) (-5847.083) -- 9:02:39      2450000 -- [-5839.554] (-5871.793) (-5836.014) (-5844.928) * (-5837.549) [-5816.603] (-5841.631) (-5859.950) -- 9:02:34      Average standard deviation of split frequencies: 0.009829      2451000 -- [-5839.951] (-5869.925) (-5846.089) (-5833.640) * (-5837.182) [-5821.921] (-5863.057) (-5843.823) -- 9:02:29      2452000 -- (-5853.154) (-5876.502) [-5829.120] (-5830.776) * (-5835.536) (-5829.488) (-5844.129) [-5820.414] -- 9:02:26      2453000 -- (-5843.292) (-5902.739) (-5832.192) [-5824.284] * (-5836.077) (-5842.243) (-5869.423) [-5810.020] -- 9:02:21      2454000 -- [-5826.765] (-5875.053) (-5831.673) (-5841.224) * (-5831.089) (-5842.775) (-5859.490) [-5823.549] -- 9:02:16      2455000 -- (-5823.606) (-5867.985) [-5819.348] (-5832.514) * [-5828.644] (-5846.011) (-5834.843) (-5834.101) -- 9:02:14      Average standard deviation of split frequencies: 0.009761      2456000 -- (-5823.896) (-5858.870) [-5816.667] (-5840.843) * [-5811.248] (-5831.798) (-5847.970) (-5830.735) -- 9:02:08      2457000 -- (-5839.919) (-5831.541) [-5818.001] (-5839.283) * [-5815.522] (-5845.245) (-5859.448) (-5820.310) -- 9:02:03      2458000 -- (-5849.619) (-5847.885) [-5816.563] (-5847.266) * (-5826.707) (-5836.064) (-5857.074) [-5810.078] -- 9:01:58      2459000 -- (-5841.939) (-5855.352) [-5818.575] (-5827.399) * (-5844.211) (-5838.959) (-5857.333) [-5812.489] -- 9:01:56      2460000 -- (-5866.087) (-5858.567) (-5825.423) [-5826.354] * (-5834.620) (-5882.697) (-5858.135) [-5814.902] -- 9:01:50      Average standard deviation of split frequencies: 0.009695      2461000 -- (-5853.310) (-5852.082) [-5826.797] (-5841.158) * (-5831.466) (-5871.388) (-5837.121) [-5822.607] -- 9:01:45      2462000 -- (-5871.035) (-5853.204) [-5834.791] (-5854.217) * (-5852.629) (-5858.173) (-5858.143) [-5817.806] -- 9:01:43      2463000 -- (-5858.064) (-5852.618) (-5841.958) [-5853.975] * (-5850.254) (-5841.474) (-5846.000) [-5824.343] -- 9:01:38      2464000 -- [-5829.640] (-5835.889) (-5835.142) (-5837.551) * (-5859.038) (-5853.930) (-5843.674) [-5828.616] -- 9:01:32      2465000 -- (-5838.982) (-5849.711) [-5815.452] (-5846.556) * (-5851.840) (-5840.979) [-5843.811] (-5850.776) -- 9:01:30      Average standard deviation of split frequencies: 0.009562      2466000 -- [-5818.336] (-5842.373) (-5838.001) (-5841.616) * (-5827.011) [-5841.904] (-5832.244) (-5861.175) -- 9:01:25      2467000 -- [-5809.499] (-5844.186) (-5823.531) (-5840.638) * (-5829.724) (-5838.012) (-5849.994) [-5835.361] -- 9:01:20      2468000 -- (-5817.756) (-5850.480) (-5838.794) [-5818.651] * (-5835.177) (-5826.029) (-5847.424) [-5821.990] -- 9:01:14      2469000 -- (-5815.001) (-5846.583) [-5825.830] (-5858.423) * (-5845.654) (-5831.724) (-5866.006) [-5820.137] -- 9:01:12      2470000 -- [-5821.855] (-5846.629) (-5833.677) (-5861.201) * [-5822.757] (-5824.869) (-5862.890) (-5824.963) -- 9:01:07      Average standard deviation of split frequencies: 0.009632      2471000 -- [-5813.328] (-5831.323) (-5837.019) (-5858.572) * (-5833.097) (-5838.401) (-5852.058) [-5824.060] -- 9:01:02      2472000 -- [-5816.426] (-5851.305) (-5826.754) (-5858.056) * [-5829.289] (-5849.007) (-5852.957) (-5851.860) -- 9:00:59      2473000 -- (-5838.782) (-5845.608) [-5841.839] (-5886.471) * [-5833.245] (-5817.660) (-5847.848) (-5848.592) -- 9:00:54      2474000 -- (-5817.648) [-5848.200] (-5845.339) (-5869.938) * (-5826.304) [-5811.526] (-5846.899) (-5831.025) -- 9:00:49      2475000 -- (-5814.496) (-5851.012) [-5836.890] (-5875.751) * (-5852.654) [-5820.319] (-5822.774) (-5852.031) -- 9:00:47      Average standard deviation of split frequencies: 0.009644      2476000 -- (-5837.430) (-5861.223) [-5819.346] (-5868.212) * (-5847.026) [-5817.501] (-5845.716) (-5858.298) -- 9:00:41      2477000 -- [-5829.757] (-5864.802) (-5821.623) (-5874.996) * (-5837.047) (-5826.989) [-5813.348] (-5855.347) -- 9:00:36      2478000 -- [-5823.688] (-5869.419) (-5829.087) (-5856.409) * (-5848.361) [-5832.518] (-5838.844) (-5848.308) -- 9:00:31      2479000 -- (-5834.215) (-5866.965) [-5821.530] (-5868.442) * (-5848.194) (-5838.192) [-5823.567] (-5849.674) -- 9:00:29      2480000 -- (-5838.908) (-5837.953) [-5822.291] (-5851.722) * (-5847.329) (-5824.355) [-5827.517] (-5868.680) -- 9:00:23      Average standard deviation of split frequencies: 0.009710      2481000 -- (-5869.520) (-5835.120) [-5834.684] (-5846.438) * (-5854.579) [-5840.562] (-5830.178) (-5849.944) -- 9:00:18      2482000 -- (-5861.843) (-5840.392) [-5829.697] (-5856.746) * (-5861.498) (-5853.230) [-5841.050] (-5837.552) -- 9:00:13      2483000 -- (-5850.571) (-5845.638) [-5830.978] (-5846.156) * (-5853.321) (-5844.136) (-5837.397) [-5818.414] -- 9:00:11      2484000 -- (-5848.159) (-5853.612) [-5833.294] (-5834.873) * (-5846.272) (-5839.736) (-5853.436) [-5813.385] -- 9:00:05      2485000 -- (-5843.630) (-5874.395) [-5825.091] (-5837.833) * (-5859.893) [-5836.205] (-5836.271) (-5830.558) -- 9:00:03      Average standard deviation of split frequencies: 0.009641      2486000 -- (-5852.772) (-5863.138) [-5823.297] (-5841.569) * (-5846.396) (-5848.814) [-5828.106] (-5850.250) -- 8:59:58      2487000 -- (-5859.330) (-5868.629) [-5832.326] (-5850.491) * (-5856.842) (-5840.157) [-5824.039] (-5840.942) -- 8:59:53      2488000 -- [-5823.099] (-5850.719) (-5833.741) (-5852.624) * (-5862.630) (-5843.724) [-5828.388] (-5842.455) -- 8:59:47      2489000 -- [-5828.148] (-5827.561) (-5830.489) (-5869.023) * (-5870.502) (-5838.269) [-5834.881] (-5862.859) -- 8:59:45      2490000 -- (-5818.090) (-5823.199) [-5830.127] (-5848.723) * (-5831.565) [-5831.344] (-5844.333) (-5853.974) -- 8:59:40      Average standard deviation of split frequencies: 0.009572      2491000 -- (-5817.094) (-5830.454) [-5815.919] (-5838.008) * [-5820.163] (-5838.030) (-5847.244) (-5829.860) -- 8:59:35      2492000 -- [-5814.885] (-5843.755) (-5829.912) (-5829.560) * (-5834.980) [-5840.510] (-5829.847) (-5820.247) -- 8:59:32      2493000 -- [-5835.999] (-5848.922) (-5838.840) (-5830.107) * (-5833.832) (-5840.949) (-5837.633) [-5821.577] -- 8:59:27      2494000 -- [-5823.702] (-5869.123) (-5845.972) (-5826.824) * (-5850.026) [-5816.602] (-5828.497) (-5833.996) -- 8:59:22      2495000 -- (-5857.174) (-5856.021) (-5828.173) [-5818.705] * (-5867.776) (-5823.090) [-5819.955] (-5851.366) -- 8:59:20      Average standard deviation of split frequencies: 0.009481      2496000 -- (-5847.739) (-5842.072) (-5819.242) [-5827.758] * (-5849.038) [-5833.136] (-5824.667) (-5837.825) -- 8:59:14      2497000 -- (-5841.763) (-5843.700) [-5833.249] (-5822.581) * (-5854.988) (-5859.843) [-5819.477] (-5833.890) -- 8:59:09      2498000 -- (-5844.054) (-5854.120) (-5824.097) [-5835.434] * (-5853.107) (-5844.700) [-5820.688] (-5834.091) -- 8:59:04      2499000 -- [-5836.563] (-5854.878) (-5839.110) (-5829.308) * (-5839.472) (-5845.539) [-5822.614] (-5837.748) -- 8:59:02      2500000 -- (-5836.810) (-5875.820) (-5843.406) [-5820.640] * (-5833.365) (-5836.151) [-5821.296] (-5845.018) -- 8:58:56      Average standard deviation of split frequencies: 0.009437      2501000 -- [-5815.075] (-5848.488) (-5840.745) (-5838.708) * (-5826.131) (-5832.706) [-5818.303] (-5868.118) -- 8:58:51      2502000 -- [-5823.835] (-5857.346) (-5835.291) (-5823.702) * (-5837.006) (-5831.317) [-5812.413] (-5860.304) -- 8:58:46      2503000 -- (-5831.267) (-5877.947) (-5838.042) [-5820.948] * (-5837.425) (-5826.983) [-5816.360] (-5853.679) -- 8:58:44      2504000 -- (-5826.019) (-5853.452) (-5854.128) [-5829.943] * (-5855.009) (-5832.508) [-5820.403] (-5848.831) -- 8:58:39      2505000 -- [-5829.408] (-5856.614) (-5851.699) (-5834.573) * (-5836.514) (-5832.544) [-5818.888] (-5847.847) -- 8:58:33      Average standard deviation of split frequencies: 0.009280      2506000 -- (-5841.138) [-5829.296] (-5862.164) (-5830.589) * (-5853.262) (-5827.300) [-5833.725] (-5835.043) -- 8:58:31      2507000 -- (-5825.127) (-5856.014) (-5851.237) [-5821.960] * (-5837.866) [-5823.672] (-5834.895) (-5835.152) -- 8:58:26      2508000 -- (-5830.833) [-5818.103] (-5822.811) (-5841.370) * (-5846.441) (-5826.326) (-5851.372) [-5831.236] -- 8:58:21      2509000 -- (-5843.811) [-5817.877] (-5822.269) (-5844.681) * (-5839.797) (-5828.860) (-5830.361) [-5818.576] -- 8:58:15      2510000 -- (-5839.423) [-5813.596] (-5842.310) (-5847.623) * [-5842.121] (-5841.001) (-5825.880) (-5835.214) -- 8:58:10      Average standard deviation of split frequencies: 0.009219      2511000 -- (-5838.331) [-5814.002] (-5859.398) (-5836.589) * (-5836.432) [-5828.710] (-5841.226) (-5854.656) -- 8:58:08      2512000 -- (-5835.881) [-5809.936] (-5863.855) (-5837.446) * (-5830.693) (-5824.416) [-5825.474] (-5868.693) -- 8:58:03      2513000 -- (-5851.002) (-5826.902) (-5868.403) [-5853.152] * (-5824.111) [-5826.752] (-5842.101) (-5880.901) -- 8:57:57      2514000 -- (-5852.814) [-5831.054] (-5866.116) (-5843.950) * [-5819.013] (-5838.304) (-5839.114) (-5861.176) -- 8:57:52      2515000 -- (-5835.236) [-5825.117] (-5854.282) (-5852.644) * [-5819.241] (-5852.127) (-5839.512) (-5844.863) -- 8:57:50      Average standard deviation of split frequencies: 0.009177      2516000 -- (-5838.576) [-5840.743] (-5864.423) (-5838.477) * (-5825.447) (-5846.971) [-5830.708] (-5845.659) -- 8:57:45      2517000 -- (-5825.416) [-5833.668] (-5851.630) (-5836.361) * [-5828.027] (-5841.041) (-5850.940) (-5864.667) -- 8:57:39      2518000 -- (-5819.388) [-5825.443] (-5858.483) (-5834.315) * [-5821.655] (-5840.150) (-5850.564) (-5830.099) -- 8:57:37      2519000 -- (-5843.135) (-5824.363) (-5853.867) [-5825.517] * (-5821.670) (-5853.193) (-5841.007) [-5843.936] -- 8:57:32      2520000 -- (-5857.714) (-5834.646) (-5846.334) [-5829.215] * (-5840.881) (-5849.164) (-5823.854) [-5853.015] -- 8:57:27      Average standard deviation of split frequencies: 0.009114      2521000 -- (-5864.272) [-5817.345] (-5854.015) (-5872.936) * (-5830.760) (-5820.765) [-5845.527] (-5862.755) -- 8:57:24      2522000 -- (-5855.952) [-5826.802] (-5852.482) (-5843.765) * [-5827.115] (-5830.256) (-5839.874) (-5863.565) -- 8:57:19      2523000 -- (-5841.259) [-5831.579] (-5864.859) (-5831.298) * (-5843.927) (-5845.312) [-5832.339] (-5842.220) -- 8:57:14      2524000 -- (-5855.620) [-5826.441] (-5853.078) (-5832.739) * (-5872.985) (-5839.972) [-5838.617] (-5839.976) -- 8:57:09      2525000 -- (-5842.418) (-5861.664) (-5849.580) [-5831.721] * (-5824.134) (-5838.361) (-5847.724) [-5827.454] -- 8:57:06      Average standard deviation of split frequencies: 0.009152      2526000 -- [-5829.853] (-5842.411) (-5853.354) (-5852.503) * (-5853.263) [-5833.343] (-5827.127) (-5826.890) -- 8:57:01      2527000 -- (-5828.670) (-5845.442) [-5839.604] (-5863.029) * (-5855.352) (-5835.795) [-5822.104] (-5826.242) -- 8:56:56      2528000 -- [-5835.402] (-5840.320) (-5854.018) (-5850.847) * (-5846.094) (-5833.830) (-5839.470) [-5827.534] -- 8:56:54      2529000 -- (-5839.433) [-5835.986] (-5855.212) (-5859.332) * (-5835.806) (-5842.246) (-5835.008) [-5830.292] -- 8:56:48      2530000 -- (-5834.074) (-5823.799) [-5829.790] (-5847.778) * (-5868.792) (-5851.946) (-5835.925) [-5830.795] -- 8:56:43      Average standard deviation of split frequencies: 0.009069      2531000 -- (-5850.902) [-5820.574] (-5855.156) (-5864.615) * (-5868.852) (-5832.476) (-5842.128) [-5821.591] -- 8:56:41      2532000 -- (-5840.809) [-5812.859] (-5833.328) (-5832.814) * (-5860.591) (-5829.428) [-5836.747] (-5825.748) -- 8:56:36      2533000 -- (-5847.363) [-5823.127] (-5843.205) (-5827.155) * (-5862.796) (-5842.694) (-5868.739) [-5817.034] -- 8:56:30      2534000 -- (-5858.683) (-5824.333) (-5845.464) [-5815.731] * (-5843.381) (-5832.761) (-5869.195) [-5821.367] -- 8:56:28      2535000 -- (-5862.306) [-5827.248] (-5823.762) (-5831.348) * (-5836.690) [-5828.104] (-5869.540) (-5833.001) -- 8:56:23      Average standard deviation of split frequencies: 0.009049      2536000 -- (-5847.605) (-5825.487) (-5826.763) [-5845.246] * (-5842.833) (-5835.771) (-5868.480) [-5836.745] -- 8:56:18      2537000 -- (-5865.208) [-5824.215] (-5829.791) (-5837.099) * (-5857.152) (-5856.317) (-5848.435) [-5842.618] -- 8:56:15      2538000 -- (-5861.300) [-5815.179] (-5829.045) (-5821.946) * (-5845.590) (-5865.034) [-5848.227] (-5831.218) -- 8:56:10      2539000 -- (-5865.721) (-5831.279) (-5820.036) [-5814.991] * (-5844.617) (-5843.055) (-5844.211) [-5823.687] -- 8:56:05      2540000 -- (-5850.639) (-5853.244) (-5822.986) [-5830.547] * (-5838.372) [-5839.391] (-5841.825) (-5823.459) -- 8:56:03      Average standard deviation of split frequencies: 0.009016      2541000 -- (-5835.618) [-5816.650] (-5840.535) (-5844.608) * (-5858.731) (-5826.102) (-5842.265) [-5829.694] -- 8:55:57      2542000 -- (-5842.604) [-5825.936] (-5843.554) (-5847.415) * (-5859.665) (-5839.490) (-5823.516) [-5809.391] -- 8:55:52      2543000 -- [-5831.519] (-5836.779) (-5849.748) (-5848.683) * (-5854.474) (-5846.869) (-5815.510) [-5822.181] -- 8:55:47      2544000 -- (-5831.673) (-5842.747) (-5843.447) [-5838.409] * (-5838.659) (-5860.668) [-5820.652] (-5839.841) -- 8:55:45      2545000 -- (-5844.034) (-5836.571) (-5842.951) [-5819.961] * (-5841.512) (-5857.918) [-5822.336] (-5842.747) -- 8:55:39      Average standard deviation of split frequencies: 0.009021      2546000 -- [-5845.072] (-5852.747) (-5837.996) (-5848.998) * (-5823.665) (-5866.105) [-5824.256] (-5842.682) -- 8:55:34      2547000 -- (-5835.651) (-5839.537) (-5855.075) [-5820.902] * (-5849.503) [-5845.979] (-5829.236) (-5850.289) -- 8:55:29      2548000 -- [-5825.701] (-5846.751) (-5834.081) (-5819.321) * (-5837.630) (-5842.848) [-5834.872] (-5836.712) -- 8:55:27      2549000 -- [-5827.415] (-5842.039) (-5855.550) (-5830.252) * (-5840.720) [-5841.352] (-5845.351) (-5832.034) -- 8:55:22      2550000 -- (-5845.797) (-5851.250) (-5852.682) [-5832.435] * (-5843.731) (-5847.246) (-5855.699) [-5823.092] -- 8:55:16      Average standard deviation of split frequencies: 0.008977      2551000 -- (-5849.979) (-5855.624) (-5857.751) [-5823.137] * [-5832.651] (-5836.056) (-5845.108) (-5833.207) -- 8:55:11      2552000 -- [-5813.687] (-5850.846) (-5861.208) (-5825.960) * (-5828.346) [-5822.773] (-5842.988) (-5837.644) -- 8:55:09      2553000 -- (-5825.519) (-5838.364) (-5849.957) [-5836.301] * (-5842.030) [-5827.635] (-5838.502) (-5836.893) -- 8:55:04      2554000 -- [-5830.044] (-5847.123) (-5850.365) (-5828.983) * [-5827.046] (-5833.215) (-5848.516) (-5846.788) -- 8:54:58      2555000 -- (-5842.274) (-5832.088) (-5845.087) [-5819.742] * (-5832.613) [-5818.444] (-5865.170) (-5857.167) -- 8:54:53      Average standard deviation of split frequencies: 0.008990      2556000 -- (-5841.403) (-5839.451) [-5841.541] (-5819.840) * (-5847.965) (-5818.886) (-5875.997) [-5841.885] -- 8:54:48      2557000 -- [-5835.670] (-5843.983) (-5820.072) (-5825.424) * (-5838.489) [-5825.561] (-5858.260) (-5858.809) -- 8:54:46      2558000 -- (-5847.380) (-5838.262) [-5839.259] (-5836.223) * (-5840.848) [-5818.536] (-5843.576) (-5843.826) -- 8:54:40      2559000 -- [-5834.323] (-5849.433) (-5838.341) (-5842.938) * (-5843.799) [-5805.978] (-5853.985) (-5832.164) -- 8:54:35      2560000 -- (-5851.598) [-5826.293] (-5846.667) (-5836.208) * (-5830.221) [-5813.204] (-5848.343) (-5828.743) -- 8:54:30      Average standard deviation of split frequencies: 0.008905      2561000 -- (-5841.734) (-5835.001) (-5844.205) [-5828.809] * (-5826.528) (-5834.614) (-5856.061) [-5829.660] -- 8:54:28      2562000 -- (-5851.457) [-5831.491] (-5870.598) (-5827.859) * (-5842.570) [-5822.302] (-5852.161) (-5837.962) -- 8:54:22      2563000 -- (-5838.428) (-5831.584) (-5861.684) [-5822.217] * (-5841.700) (-5826.880) (-5847.931) [-5807.702] -- 8:54:17      2564000 -- (-5849.144) (-5836.787) (-5861.300) [-5830.995] * (-5836.385) [-5823.207] (-5836.426) (-5820.416) -- 8:54:12      2565000 -- [-5833.705] (-5844.130) (-5857.816) (-5826.187) * (-5833.966) (-5834.282) (-5831.539) [-5810.561] -- 8:54:10      Average standard deviation of split frequencies: 0.008833      2566000 -- (-5841.692) (-5825.900) (-5846.866) [-5825.110] * (-5840.918) (-5843.135) (-5837.881) [-5810.367] -- 8:54:05      2567000 -- (-5841.683) (-5833.026) [-5829.872] (-5818.177) * (-5840.280) (-5864.784) [-5830.311] (-5818.650) -- 8:53:59      2568000 -- (-5842.380) (-5842.560) (-5818.662) [-5824.950] * (-5848.076) (-5832.131) (-5844.457) [-5820.773] -- 8:53:54      2569000 -- [-5822.979] (-5850.410) (-5818.276) (-5831.402) * (-5843.049) [-5819.839] (-5837.997) (-5822.233) -- 8:53:52      2570000 -- (-5828.974) (-5851.878) (-5862.931) [-5823.529] * (-5834.502) (-5824.175) (-5838.714) [-5831.217] -- 8:53:47      Average standard deviation of split frequencies: 0.008784      2571000 -- [-5818.398] (-5849.313) (-5855.514) (-5823.736) * (-5826.873) (-5854.651) [-5831.635] (-5836.579) -- 8:53:41      2572000 -- [-5816.076] (-5860.851) (-5849.391) (-5823.877) * (-5848.450) (-5848.224) [-5828.980] (-5831.506) -- 8:53:39      2573000 -- [-5824.446] (-5856.087) (-5870.715) (-5830.591) * (-5844.462) (-5861.460) (-5834.963) [-5813.510] -- 8:53:34      2574000 -- (-5835.427) (-5858.757) (-5847.351) [-5819.338] * (-5832.775) (-5871.206) (-5851.661) [-5828.938] -- 8:53:29      2575000 -- (-5829.674) (-5830.439) (-5840.339) [-5813.869] * [-5816.688] (-5848.128) (-5830.888) (-5827.051) -- 8:53:23      Average standard deviation of split frequencies: 0.008609      2576000 -- [-5824.205] (-5838.697) (-5845.891) (-5832.661) * (-5836.970) (-5867.750) (-5834.998) [-5821.002] -- 8:53:18      2577000 -- (-5847.674) (-5837.238) [-5838.853] (-5826.982) * (-5859.028) (-5868.899) (-5841.013) [-5826.090] -- 8:53:16      2578000 -- (-5856.779) [-5837.029] (-5839.340) (-5836.633) * (-5844.981) (-5853.981) (-5838.575) [-5827.021] -- 8:53:11      2579000 -- [-5839.382] (-5838.183) (-5844.086) (-5843.296) * (-5853.759) (-5855.389) [-5832.379] (-5839.444) -- 8:53:05      2580000 -- (-5845.225) (-5858.956) [-5827.542] (-5852.233) * [-5824.585] (-5871.995) (-5830.819) (-5841.340) -- 8:53:00      Average standard deviation of split frequencies: 0.008420      2581000 -- (-5833.348) (-5857.126) [-5827.137] (-5843.135) * (-5827.135) (-5847.032) (-5849.948) [-5830.965] -- 8:52:58      2582000 -- [-5839.156] (-5867.343) (-5854.344) (-5829.552) * (-5824.526) (-5837.330) (-5860.455) [-5835.328] -- 8:52:53      2583000 -- [-5833.828] (-5862.296) (-5830.967) (-5841.195) * [-5832.540] (-5836.637) (-5853.292) (-5846.049) -- 8:52:48      2584000 -- [-5818.495] (-5851.764) (-5831.700) (-5854.475) * [-5820.466] (-5843.085) (-5855.939) (-5843.029) -- 8:52:42      2585000 -- [-5813.128] (-5851.192) (-5833.381) (-5860.904) * [-5810.337] (-5835.008) (-5871.727) (-5849.533) -- 8:52:40      Average standard deviation of split frequencies: 0.008350      2586000 -- (-5818.511) [-5831.871] (-5837.105) (-5845.312) * [-5817.962] (-5842.215) (-5863.088) (-5845.537) -- 8:52:35      2587000 -- [-5829.922] (-5833.882) (-5857.413) (-5841.949) * (-5829.066) [-5827.232] (-5853.718) (-5847.842) -- 8:52:30      2588000 -- (-5850.080) [-5840.038] (-5864.741) (-5824.134) * [-5824.256] (-5835.654) (-5860.495) (-5844.120) -- 8:52:24      2589000 -- (-5845.969) [-5825.851] (-5854.646) (-5817.601) * [-5825.023] (-5833.694) (-5858.194) (-5850.015) -- 8:52:22      2590000 -- (-5850.110) (-5834.949) (-5859.491) [-5834.478] * [-5824.895] (-5844.436) (-5847.845) (-5865.823) -- 8:52:17      Average standard deviation of split frequencies: 0.008211      2591000 -- (-5843.076) [-5822.006] (-5861.004) (-5828.775) * [-5828.761] (-5840.943) (-5829.734) (-5844.633) -- 8:52:12      2592000 -- (-5836.901) [-5822.411] (-5861.963) (-5824.562) * (-5839.357) (-5839.680) [-5819.192] (-5859.089) -- 8:52:09      2593000 -- (-5856.184) [-5821.072] (-5863.779) (-5825.375) * (-5831.432) (-5833.476) [-5816.733] (-5853.463) -- 8:52:04      2594000 -- (-5867.482) [-5815.906] (-5839.484) (-5836.477) * (-5841.519) (-5845.213) [-5830.793] (-5844.086) -- 8:51:59      2595000 -- (-5863.368) [-5819.562] (-5852.603) (-5838.631) * (-5826.214) (-5841.934) [-5828.553] (-5866.412) -- 8:51:54      Average standard deviation of split frequencies: 0.008125      2596000 -- (-5859.757) (-5823.352) (-5869.976) [-5824.465] * (-5837.004) [-5839.684] (-5830.149) (-5854.350) -- 8:51:51      2597000 -- (-5846.118) [-5836.765] (-5860.705) (-5834.664) * (-5839.969) (-5848.193) [-5819.913] (-5863.712) -- 8:51:46      2598000 -- (-5845.842) [-5832.748] (-5836.651) (-5846.259) * (-5838.057) (-5832.757) [-5828.536] (-5859.878) -- 8:51:41      2599000 -- (-5840.223) (-5845.211) [-5826.683] (-5839.439) * (-5845.621) (-5839.140) [-5817.673] (-5856.399) -- 8:51:39      2600000 -- (-5831.562) (-5856.417) [-5821.736] (-5847.066) * (-5850.205) (-5827.857) [-5821.571] (-5833.901) -- 8:51:33      Average standard deviation of split frequencies: 0.008103      2601000 -- (-5858.222) (-5860.956) [-5821.370] (-5821.808) * (-5871.516) (-5828.650) [-5820.846] (-5840.730) -- 8:51:28      2602000 -- (-5846.128) (-5859.159) (-5821.689) [-5812.172] * (-5877.456) [-5823.370] (-5830.107) (-5837.437) -- 8:51:23      2603000 -- (-5837.606) (-5840.984) [-5819.888] (-5837.529) * (-5865.813) (-5821.645) [-5826.794] (-5849.443) -- 8:51:18      2604000 -- (-5855.520) (-5843.076) [-5815.609] (-5833.972) * (-5857.773) (-5835.057) [-5825.836] (-5848.711) -- 8:51:16      2605000 -- (-5863.254) (-5839.790) (-5839.056) [-5827.002] * (-5839.037) (-5830.863) [-5820.818] (-5837.950) -- 8:51:10      Average standard deviation of split frequencies: 0.008050      2606000 -- (-5847.304) [-5832.397] (-5839.471) (-5840.753) * (-5834.088) (-5854.470) [-5838.118] (-5851.685) -- 8:51:05      2607000 -- (-5870.518) (-5837.927) (-5860.271) [-5831.016] * [-5828.350] (-5856.289) (-5831.696) (-5864.636) -- 8:51:00      2608000 -- [-5838.465] (-5842.227) (-5834.493) (-5853.043) * [-5838.413] (-5869.212) (-5830.362) (-5842.885) -- 8:50:58      2609000 -- [-5821.656] (-5839.503) (-5837.937) (-5853.775) * (-5835.388) (-5860.593) [-5824.136] (-5839.326) -- 8:50:52      2610000 -- [-5846.039] (-5849.432) (-5854.591) (-5841.075) * (-5835.804) (-5869.071) [-5819.961] (-5833.520) -- 8:50:47      Average standard deviation of split frequencies: 0.007964      2611000 -- (-5845.678) (-5855.832) [-5831.745] (-5854.393) * (-5848.725) (-5856.762) [-5813.785] (-5822.022) -- 8:50:42      2612000 -- (-5836.855) [-5833.939] (-5844.401) (-5865.405) * (-5860.222) (-5848.421) (-5831.644) [-5819.266] -- 8:50:40      2613000 -- [-5827.513] (-5835.451) (-5862.802) (-5849.490) * (-5855.246) (-5845.423) (-5840.558) [-5822.776] -- 8:50:35      2614000 -- (-5824.124) [-5825.014] (-5863.669) (-5870.289) * (-5836.798) (-5849.714) (-5831.958) [-5824.964] -- 8:50:29      2615000 -- (-5828.166) [-5821.516] (-5880.705) (-5863.934) * (-5860.433) (-5854.085) (-5836.328) [-5833.912] -- 8:50:27      Average standard deviation of split frequencies: 0.007892      2616000 -- [-5824.454] (-5832.202) (-5875.086) (-5847.964) * (-5862.100) (-5861.490) [-5814.091] (-5848.549) -- 8:50:22      2617000 -- (-5838.320) [-5839.112] (-5868.032) (-5846.518) * (-5830.577) (-5846.170) [-5831.132] (-5849.235) -- 8:50:17      2618000 -- (-5848.259) (-5828.827) (-5847.760) [-5828.757] * [-5822.020] (-5837.784) (-5851.605) (-5842.053) -- 8:50:14      2619000 -- (-5865.154) [-5820.150] (-5831.054) (-5823.366) * [-5800.612] (-5831.290) (-5842.390) (-5844.340) -- 8:50:09      2620000 -- (-5845.391) (-5842.238) [-5823.759] (-5846.109) * [-5817.506] (-5820.907) (-5837.835) (-5851.607) -- 8:50:04      Average standard deviation of split frequencies: 0.007860      2621000 -- (-5831.332) (-5842.278) [-5815.688] (-5854.430) * (-5824.336) [-5835.752] (-5830.404) (-5852.900) -- 8:50:02      2622000 -- (-5834.818) (-5849.486) [-5813.676] (-5825.343) * (-5838.872) [-5821.411] (-5839.939) (-5845.043) -- 8:49:56      2623000 -- (-5840.981) (-5849.959) (-5824.461) [-5815.370] * (-5853.213) [-5813.384] (-5854.502) (-5841.431) -- 8:49:51      2624000 -- (-5851.862) (-5860.642) (-5815.009) [-5829.606] * [-5818.383] (-5828.018) (-5863.836) (-5844.249) -- 8:49:49      2625000 -- (-5850.067) (-5852.487) [-5819.370] (-5834.799) * [-5837.268] (-5818.031) (-5857.786) (-5838.437) -- 8:49:44      Average standard deviation of split frequencies: 0.007808      2626000 -- (-5848.359) (-5867.286) [-5812.555] (-5835.882) * (-5838.677) (-5825.619) (-5867.532) [-5824.971] -- 8:49:38      2627000 -- (-5831.975) (-5833.275) [-5826.130] (-5830.931) * [-5834.877] (-5845.529) (-5865.855) (-5821.485) -- 8:49:36      2628000 -- (-5827.099) [-5828.585] (-5820.979) (-5836.255) * (-5840.043) (-5849.368) [-5830.668] (-5847.715) -- 8:49:31      2629000 -- (-5869.570) (-5842.260) [-5821.939] (-5824.994) * (-5846.793) [-5845.414] (-5839.377) (-5850.980) -- 8:49:26      2630000 -- (-5859.307) (-5829.713) (-5816.982) [-5813.918] * (-5836.996) [-5836.665] (-5846.558) (-5853.734) -- 8:49:21      Average standard deviation of split frequencies: 0.007752      2631000 -- (-5861.800) (-5823.618) (-5840.043) [-5816.972] * (-5867.633) [-5831.915] (-5845.185) (-5865.581) -- 8:49:18      2632000 -- (-5840.945) (-5843.110) (-5867.200) [-5812.269] * (-5863.837) [-5830.609] (-5838.104) (-5855.876) -- 8:49:13      2633000 -- (-5846.491) (-5841.941) (-5844.306) [-5811.813] * (-5856.338) [-5827.545] (-5831.118) (-5853.340) -- 8:49:08      2634000 -- (-5856.522) (-5827.850) (-5838.397) [-5804.657] * [-5837.634] (-5820.907) (-5837.051) (-5868.369) -- 8:49:03      2635000 -- (-5846.999) (-5833.703) (-5829.174) [-5834.673] * (-5823.639) [-5816.871] (-5844.333) (-5862.345) -- 8:49:00      Average standard deviation of split frequencies: 0.007695      2636000 -- (-5850.670) [-5830.922] (-5851.347) (-5852.997) * (-5818.365) [-5812.578] (-5827.323) (-5867.984) -- 8:48:55      2637000 -- (-5852.330) [-5825.369] (-5836.769) (-5836.786) * (-5830.274) [-5814.932] (-5832.911) (-5863.930) -- 8:48:50      2638000 -- (-5858.770) [-5817.257] (-5845.331) (-5840.086) * (-5832.450) [-5823.960] (-5828.503) (-5850.745) -- 8:48:48      2639000 -- (-5834.407) [-5826.762] (-5856.934) (-5834.130) * [-5826.403] (-5834.829) (-5840.505) (-5863.808) -- 8:48:42      2640000 -- (-5855.185) [-5833.253] (-5862.121) (-5833.869) * [-5832.627] (-5840.812) (-5854.846) (-5852.540) -- 8:48:37      Average standard deviation of split frequencies: 0.007637      2641000 -- (-5877.076) (-5832.773) (-5840.923) [-5827.507] * (-5826.594) [-5827.124] (-5849.289) (-5857.666) -- 8:48:35      2642000 -- (-5849.843) (-5837.826) (-5859.988) [-5829.161] * (-5827.453) [-5821.877] (-5874.474) (-5845.142) -- 8:48:30      2643000 -- (-5843.961) (-5829.137) (-5846.816) [-5811.606] * (-5830.180) [-5826.437] (-5867.420) (-5850.809) -- 8:48:24      2644000 -- (-5845.396) (-5852.161) (-5836.682) [-5801.637] * (-5842.396) [-5842.518] (-5853.614) (-5850.847) -- 8:48:22      2645000 -- (-5855.880) (-5838.733) [-5817.286] (-5824.873) * [-5825.376] (-5828.904) (-5840.303) (-5866.941) -- 8:48:17      Average standard deviation of split frequencies: 0.007723      2646000 -- (-5835.869) (-5836.738) (-5825.949) [-5827.005] * [-5831.404] (-5849.299) (-5845.757) (-5838.772) -- 8:48:12      2647000 -- (-5836.790) (-5846.562) (-5829.421) [-5816.698] * (-5867.777) (-5835.949) [-5829.965] (-5844.388) -- 8:48:09      2648000 -- (-5853.591) (-5861.786) [-5813.816] (-5825.022) * (-5887.879) (-5818.028) [-5837.053] (-5845.689) -- 8:48:04      2649000 -- (-5856.910) (-5830.339) [-5817.813] (-5828.975) * (-5843.562) (-5826.315) [-5827.278] (-5843.163) -- 8:47:59      2650000 -- (-5857.621) (-5831.327) [-5822.050] (-5839.861) * (-5848.541) [-5825.442] (-5848.614) (-5841.592) -- 8:47:57      Average standard deviation of split frequencies: 0.007691      2651000 -- (-5845.059) (-5830.599) (-5829.460) [-5807.531] * [-5833.699] (-5832.003) (-5846.525) (-5838.147) -- 8:47:51      2652000 -- (-5827.408) (-5827.589) (-5850.270) [-5826.877] * [-5823.843] (-5835.255) (-5857.305) (-5836.510) -- 8:47:46      2653000 -- (-5831.298) [-5837.833] (-5838.569) (-5832.522) * [-5814.172] (-5830.167) (-5866.601) (-5830.435) -- 8:47:44      2654000 -- (-5836.274) [-5831.872] (-5849.035) (-5828.756) * (-5812.761) (-5850.336) (-5861.425) [-5814.778] -- 8:47:39      2655000 -- (-5827.514) (-5831.699) [-5825.527] (-5843.583) * [-5802.584] (-5843.166) (-5845.079) (-5837.734) -- 8:47:34      Average standard deviation of split frequencies: 0.007637      2656000 -- (-5830.319) (-5851.363) (-5851.514) [-5833.598] * [-5819.124] (-5832.676) (-5829.568) (-5857.479) -- 8:47:28      2657000 -- (-5838.022) (-5854.241) (-5842.906) [-5814.397] * (-5844.531) [-5846.507] (-5839.003) (-5851.081) -- 8:47:26      2658000 -- [-5831.640] (-5849.033) (-5840.297) (-5836.058) * [-5847.646] (-5850.034) (-5836.867) (-5864.952) -- 8:47:21      2659000 -- [-5821.423] (-5853.015) (-5826.023) (-5841.124) * (-5848.331) (-5867.559) (-5846.078) [-5839.290] -- 8:47:16      2660000 -- [-5821.516] (-5835.793) (-5848.867) (-5829.909) * (-5838.705) (-5855.863) (-5838.807) [-5840.230] -- 8:47:13      Average standard deviation of split frequencies: 0.007667      2661000 -- (-5828.069) (-5841.857) (-5835.103) [-5819.219] * (-5867.744) [-5822.263] (-5829.677) (-5837.518) -- 8:47:08      2662000 -- (-5826.501) (-5829.741) [-5828.026] (-5851.626) * (-5863.978) (-5839.432) [-5813.949] (-5847.609) -- 8:47:03      2663000 -- [-5821.654] (-5835.823) (-5843.922) (-5838.270) * (-5861.687) (-5839.709) [-5819.266] (-5833.004) -- 8:46:58      2664000 -- (-5821.779) (-5861.792) (-5850.208) [-5840.201] * (-5864.055) [-5825.097] (-5827.444) (-5854.612) -- 8:46:55      2665000 -- (-5841.653) (-5845.895) (-5846.385) [-5852.078] * (-5886.252) (-5831.867) [-5828.491] (-5838.670) -- 8:46:50      Average standard deviation of split frequencies: 0.007635      2666000 -- (-5852.853) (-5831.451) (-5885.496) [-5840.702] * (-5862.440) (-5816.462) [-5828.041] (-5852.636) -- 8:46:45      2667000 -- [-5820.345] (-5842.409) (-5839.331) (-5851.086) * (-5852.535) [-5825.119] (-5818.054) (-5839.267) -- 8:46:40      2668000 -- [-5841.758] (-5866.488) (-5815.361) (-5848.586) * (-5847.020) (-5830.047) [-5803.579] (-5815.581) -- 8:46:37      2669000 -- (-5865.616) (-5852.019) (-5830.388) [-5826.314] * (-5849.645) (-5834.737) [-5811.926] (-5832.289) -- 8:46:32      2670000 -- (-5850.554) (-5832.414) [-5819.479] (-5829.270) * (-5831.002) (-5826.662) [-5814.164] (-5858.187) -- 8:46:27      Average standard deviation of split frequencies: 0.007555      2671000 -- (-5847.924) (-5834.754) [-5818.925] (-5835.163) * (-5842.085) (-5817.645) [-5812.765] (-5876.093) -- 8:46:22      2672000 -- (-5860.234) (-5845.872) (-5835.936) [-5838.167] * (-5858.687) (-5822.886) [-5833.944] (-5868.107) -- 8:46:20      2673000 -- (-5844.902) (-5828.101) (-5830.410) [-5824.357] * [-5846.428] (-5838.548) (-5851.587) (-5863.454) -- 8:46:14      2674000 -- (-5847.085) (-5836.790) (-5838.080) [-5833.449] * (-5851.295) [-5826.569] (-5843.132) (-5845.934) -- 8:46:09      2675000 -- (-5853.758) [-5826.107] (-5852.095) (-5840.994) * (-5866.072) [-5827.334] (-5831.505) (-5844.234) -- 8:46:04      Average standard deviation of split frequencies: 0.007509      2676000 -- (-5824.863) [-5813.325] (-5838.758) (-5857.996) * (-5861.474) (-5830.349) [-5822.706] (-5831.031) -- 8:46:02      2677000 -- (-5830.391) [-5829.127] (-5840.789) (-5849.408) * (-5872.672) (-5837.107) (-5847.440) [-5855.555] -- 8:45:57      2678000 -- (-5858.323) [-5826.591] (-5835.071) (-5841.722) * (-5878.086) (-5846.664) [-5830.976] (-5834.217) -- 8:45:54      2679000 -- (-5844.050) (-5839.766) [-5833.960] (-5853.343) * (-5868.467) (-5840.653) [-5824.902] (-5834.537) -- 8:45:49      2680000 -- [-5830.845] (-5863.839) (-5835.013) (-5840.968) * (-5853.891) (-5830.306) [-5830.738] (-5835.003) -- 8:45:44      Average standard deviation of split frequencies: 0.007542      2681000 -- (-5817.020) (-5866.799) (-5857.505) [-5817.920] * (-5854.263) [-5814.663] (-5841.074) (-5858.822) -- 8:45:39      2682000 -- [-5826.972] (-5863.743) (-5838.145) (-5835.292) * (-5849.943) [-5826.169] (-5850.004) (-5855.137) -- 8:45:36      2683000 -- [-5815.408] (-5842.576) (-5854.447) (-5836.386) * (-5852.329) [-5826.153] (-5842.347) (-5855.850) -- 8:45:31      2684000 -- [-5814.955] (-5870.708) (-5844.339) (-5847.238) * (-5847.545) (-5835.972) [-5824.145] (-5841.965) -- 8:45:26      2685000 -- (-5841.307) (-5878.653) [-5829.819] (-5848.735) * (-5850.632) [-5825.185] (-5835.473) (-5844.462) -- 8:45:23      Average standard deviation of split frequencies: 0.007485      2686000 -- (-5841.396) (-5834.400) [-5827.760] (-5831.207) * (-5834.802) [-5824.512] (-5828.939) (-5847.531) -- 8:45:18      2687000 -- (-5836.009) (-5842.549) (-5824.744) [-5833.654] * (-5828.739) [-5828.004] (-5842.141) (-5845.351) -- 8:45:13      2688000 -- (-5833.791) (-5847.125) (-5841.612) [-5824.634] * [-5830.685] (-5836.510) (-5878.128) (-5839.612) -- 8:45:08      2689000 -- (-5856.378) (-5825.676) (-5853.012) [-5821.109] * (-5851.822) (-5849.255) (-5843.724) [-5826.436] -- 8:45:06      2690000 -- (-5866.785) [-5813.411] (-5847.972) (-5825.106) * (-5841.283) (-5834.766) (-5847.496) [-5825.521] -- 8:45:00      Average standard deviation of split frequencies: 0.007496      2691000 -- (-5843.422) (-5838.942) (-5845.491) [-5825.193] * [-5826.736] (-5831.756) (-5859.480) (-5834.668) -- 8:44:55      2692000 -- (-5858.469) [-5816.543] (-5850.589) (-5824.111) * (-5812.189) [-5820.219] (-5869.798) (-5819.961) -- 8:44:53      2693000 -- (-5843.530) [-5815.943] (-5837.515) (-5817.223) * (-5823.853) [-5822.140] (-5853.985) (-5830.570) -- 8:44:48      2694000 -- (-5846.510) [-5809.214] (-5840.586) (-5823.974) * [-5827.080] (-5841.907) (-5834.802) (-5832.920) -- 8:44:43      2695000 -- (-5836.301) [-5819.802] (-5849.290) (-5831.987) * [-5821.204] (-5833.853) (-5833.477) (-5841.756) -- 8:44:37      Average standard deviation of split frequencies: 0.007414      2696000 -- (-5850.974) [-5809.812] (-5850.617) (-5843.934) * (-5829.604) [-5837.051] (-5831.833) (-5832.840) -- 8:44:35      2697000 -- [-5825.751] (-5830.213) (-5839.074) (-5834.547) * (-5833.857) [-5832.347] (-5854.401) (-5849.687) -- 8:44:30      2698000 -- (-5847.884) (-5838.908) (-5837.941) [-5827.839] * (-5835.609) [-5832.633] (-5852.513) (-5859.404) -- 8:44:25      2699000 -- (-5822.035) (-5842.951) (-5837.868) [-5831.138] * (-5833.585) [-5828.155] (-5849.056) (-5836.497) -- 8:44:22      2700000 -- (-5836.091) (-5834.774) (-5837.549) [-5835.028] * [-5820.144] (-5822.930) (-5853.046) (-5846.359) -- 8:44:17      Average standard deviation of split frequencies: 0.007381      2701000 -- (-5839.349) (-5838.754) (-5876.605) [-5833.991] * (-5820.281) [-5823.120] (-5842.043) (-5852.427) -- 8:44:12      2702000 -- (-5838.917) [-5839.496] (-5893.905) (-5849.110) * [-5820.905] (-5817.223) (-5848.072) (-5874.426) -- 8:44:07      2703000 -- (-5820.070) (-5838.430) (-5873.694) [-5828.460] * [-5824.466] (-5824.714) (-5850.772) (-5859.248) -- 8:44:02      2704000 -- (-5840.815) (-5840.703) (-5850.529) [-5822.661] * (-5846.429) [-5824.643] (-5832.944) (-5859.033) -- 8:43:59      2705000 -- (-5846.158) (-5832.264) (-5850.081) [-5812.365] * [-5829.573] (-5839.256) (-5834.945) (-5846.921) -- 8:43:54      Average standard deviation of split frequencies: 0.007430      2706000 -- [-5826.091] (-5832.309) (-5846.030) (-5827.886) * [-5829.432] (-5838.190) (-5842.702) (-5887.296) -- 8:43:49      2707000 -- (-5830.074) (-5849.988) (-5845.152) [-5816.653] * [-5836.371] (-5841.530) (-5860.555) (-5847.032) -- 8:43:44      2708000 -- (-5833.923) (-5845.346) (-5849.871) [-5817.683] * (-5855.947) (-5819.013) (-5854.024) [-5833.941] -- 8:43:39      2709000 -- (-5823.921) (-5857.106) (-5852.685) [-5812.243] * (-5830.537) (-5844.941) (-5845.056) [-5822.705] -- 8:43:36      2710000 -- (-5845.288) (-5853.923) (-5829.158) [-5811.144] * [-5822.282] (-5830.491) (-5844.124) (-5851.755) -- 8:43:31      Average standard deviation of split frequencies: 0.007436      2711000 -- (-5834.212) [-5835.012] (-5836.650) (-5846.031) * [-5836.321] (-5853.051) (-5841.349) (-5840.581) -- 8:43:26      2712000 -- (-5840.272) (-5830.982) (-5842.573) [-5845.959] * [-5816.424] (-5841.737) (-5838.618) (-5835.468) -- 8:43:23      2713000 -- (-5856.260) (-5841.467) (-5836.887) [-5831.386] * (-5836.550) (-5843.937) (-5849.186) [-5830.318] -- 8:43:18      2714000 -- (-5846.896) (-5829.879) [-5820.801] (-5840.321) * [-5830.408] (-5830.003) (-5862.750) (-5840.584) -- 8:43:13      2715000 -- (-5830.346) (-5846.137) [-5825.272] (-5848.213) * [-5851.373] (-5829.567) (-5867.381) (-5833.549) -- 8:43:11      Average standard deviation of split frequencies: 0.007422      2716000 -- (-5847.280) (-5838.552) (-5848.860) [-5830.838] * (-5837.048) [-5809.522] (-5845.048) (-5857.434) -- 8:43:06      2717000 -- [-5857.671] (-5852.046) (-5837.704) (-5847.790) * (-5840.080) [-5822.430] (-5861.579) (-5860.606) -- 8:43:00      2718000 -- (-5866.875) [-5835.423] (-5842.997) (-5830.711) * (-5852.048) [-5834.485] (-5837.628) (-5856.621) -- 8:42:58      2719000 -- (-5852.043) [-5813.814] (-5850.603) (-5828.152) * (-5840.402) (-5848.543) [-5818.006] (-5864.827) -- 8:42:53      2720000 -- (-5857.963) [-5815.219] (-5864.235) (-5826.594) * (-5836.976) (-5849.615) [-5815.555] (-5867.745) -- 8:42:48      Average standard deviation of split frequencies: 0.007460      2721000 -- (-5831.727) (-5827.072) (-5852.815) [-5803.304] * (-5860.775) [-5826.811] (-5830.359) (-5859.245) -- 8:42:45      2722000 -- (-5820.867) (-5829.436) (-5852.205) [-5808.665] * [-5853.962] (-5864.792) (-5832.314) (-5856.662) -- 8:42:40      2723000 -- (-5840.381) (-5850.670) (-5835.783) [-5810.115] * (-5843.852) (-5863.752) [-5817.406] (-5853.618) -- 8:42:35      2724000 -- (-5843.831) (-5852.149) (-5828.877) [-5819.885] * (-5822.346) (-5868.273) [-5827.374] (-5859.827) -- 8:42:30      2725000 -- [-5828.440] (-5848.418) (-5834.077) (-5838.796) * (-5832.346) (-5848.878) [-5817.467] (-5867.277) -- 8:42:27      Average standard deviation of split frequencies: 0.007492      2726000 -- [-5814.245] (-5840.899) (-5852.929) (-5832.159) * (-5823.966) (-5848.670) [-5806.265] (-5872.393) -- 8:42:22      2727000 -- [-5833.713] (-5831.000) (-5854.668) (-5828.250) * [-5833.290] (-5860.328) (-5843.620) (-5847.359) -- 8:42:17      2728000 -- (-5840.952) (-5839.115) (-5849.693) [-5833.509] * (-5820.810) (-5856.605) [-5830.354] (-5831.557) -- 8:42:12      2729000 -- (-5842.377) (-5844.245) (-5852.537) [-5833.134] * (-5851.629) (-5844.759) (-5828.741) [-5825.941] -- 8:42:10      2730000 -- (-5834.497) [-5827.929] (-5858.886) (-5859.376) * (-5841.242) (-5853.885) [-5815.969] (-5833.438) -- 8:42:04      Average standard deviation of split frequencies: 0.007439      2731000 -- (-5829.877) [-5837.790] (-5838.525) (-5852.407) * (-5862.805) [-5832.033] (-5823.681) (-5836.272) -- 8:41:59      2732000 -- [-5829.573] (-5836.627) (-5855.591) (-5850.933) * (-5863.361) [-5833.531] (-5828.177) (-5828.163) -- 8:41:57      2733000 -- [-5820.901] (-5828.666) (-5850.204) (-5847.334) * (-5857.412) [-5840.785] (-5848.973) (-5846.107) -- 8:41:52      2734000 -- [-5821.237] (-5821.519) (-5876.818) (-5855.264) * [-5828.529] (-5837.803) (-5868.807) (-5837.368) -- 8:41:47      2735000 -- [-5827.742] (-5844.877) (-5889.055) (-5846.724) * [-5826.196] (-5834.219) (-5887.940) (-5826.443) -- 8:41:44      Average standard deviation of split frequencies: 0.007469      2736000 -- (-5833.862) [-5835.070] (-5872.451) (-5840.203) * [-5824.251] (-5844.575) (-5872.505) (-5823.294) -- 8:41:39      2737000 -- (-5826.972) [-5821.987] (-5882.614) (-5825.743) * [-5812.373] (-5844.685) (-5859.560) (-5846.254) -- 8:41:34      2738000 -- (-5821.311) (-5832.521) (-5856.869) [-5827.316] * (-5820.225) (-5845.700) (-5853.642) [-5816.467] -- 8:41:29      2739000 -- (-5837.358) [-5823.109] (-5847.645) (-5833.271) * [-5818.753] (-5848.380) (-5868.699) (-5830.744) -- 8:41:26      2740000 -- [-5816.847] (-5836.206) (-5868.942) (-5839.567) * (-5845.773) (-5833.065) (-5851.276) [-5829.626] -- 8:41:21      Average standard deviation of split frequencies: 0.007490      2741000 -- [-5825.256] (-5837.934) (-5873.780) (-5844.278) * (-5856.260) (-5837.624) [-5838.486] (-5812.599) -- 8:41:16      2742000 -- (-5840.798) [-5840.177] (-5846.812) (-5830.165) * (-5838.212) (-5843.240) (-5845.393) [-5827.180] -- 8:41:11      2743000 -- (-5847.921) [-5812.754] (-5836.684) (-5844.326) * [-5826.167] (-5843.813) (-5843.743) (-5826.638) -- 8:41:08      2744000 -- (-5834.992) (-5830.088) (-5853.838) [-5829.937] * (-5838.726) (-5838.798) (-5856.500) [-5826.436] -- 8:41:03      2745000 -- (-5839.653) (-5833.024) (-5845.556) [-5815.566] * (-5867.910) (-5846.861) (-5849.147) [-5825.857] -- 8:40:58      Average standard deviation of split frequencies: 0.007415      2746000 -- (-5836.926) (-5836.307) (-5837.474) [-5817.968] * (-5839.750) (-5831.180) (-5845.169) [-5826.651] -- 8:40:56      2747000 -- [-5827.054] (-5841.012) (-5820.607) (-5852.851) * (-5824.211) (-5828.027) (-5855.496) [-5810.427] -- 8:40:51      2748000 -- [-5839.511] (-5836.987) (-5831.783) (-5839.476) * [-5831.718] (-5827.036) (-5850.168) (-5824.195) -- 8:40:45      2749000 -- (-5844.379) [-5829.471] (-5859.185) (-5837.912) * (-5829.094) [-5839.647] (-5866.549) (-5832.876) -- 8:40:40      2750000 -- [-5836.588] (-5857.171) (-5833.063) (-5849.184) * [-5820.457] (-5835.518) (-5850.307) (-5824.501) -- 8:40:38      Average standard deviation of split frequencies: 0.007312      2751000 -- [-5828.324] (-5839.003) (-5873.456) (-5837.315) * (-5818.068) (-5835.323) (-5844.283) [-5818.514] -- 8:40:33      2752000 -- [-5822.658] (-5832.125) (-5840.246) (-5853.587) * [-5823.761] (-5857.373) (-5830.053) (-5829.990) -- 8:40:28      2753000 -- (-5827.516) [-5823.442] (-5838.665) (-5850.202) * (-5838.670) (-5847.986) (-5840.370) [-5830.721] -- 8:40:25      2754000 -- [-5829.810] (-5820.521) (-5845.890) (-5838.074) * (-5845.281) (-5843.310) (-5828.199) [-5851.572] -- 8:40:20      2755000 -- (-5830.923) [-5821.283] (-5853.771) (-5846.585) * (-5829.662) [-5824.116] (-5844.504) (-5848.638) -- 8:40:15      Average standard deviation of split frequencies: 0.007298      2756000 -- (-5849.073) [-5819.059] (-5859.469) (-5823.864) * [-5823.543] (-5835.431) (-5828.351) (-5854.064) -- 8:40:12      2757000 -- (-5851.541) (-5851.286) (-5868.895) [-5833.421] * [-5823.802] (-5870.258) (-5823.710) (-5853.896) -- 8:40:07      2758000 -- [-5835.518] (-5844.358) (-5848.733) (-5836.168) * (-5820.344) (-5858.976) [-5824.231] (-5849.325) -- 8:40:02      2759000 -- [-5828.157] (-5829.414) (-5842.330) (-5822.284) * [-5813.561] (-5852.473) (-5836.469) (-5864.998) -- 8:39:57      2760000 -- [-5820.560] (-5833.494) (-5850.139) (-5835.665) * [-5816.100] (-5847.025) (-5841.071) (-5868.529) -- 8:39:52      Average standard deviation of split frequencies: 0.007306      2761000 -- (-5846.281) [-5818.654] (-5858.247) (-5833.515) * (-5814.388) (-5841.802) [-5842.565] (-5851.178) -- 8:39:49      2762000 -- (-5854.493) [-5815.765] (-5846.953) (-5834.748) * [-5822.605] (-5849.136) (-5834.554) (-5862.845) -- 8:39:44      2763000 -- (-5844.218) (-5818.352) (-5857.069) [-5826.225] * [-5812.287] (-5847.166) (-5829.752) (-5841.546) -- 8:39:39      2764000 -- (-5851.475) [-5822.043] (-5841.638) (-5833.011) * [-5817.372] (-5865.054) (-5827.262) (-5842.454) -- 8:39:34      2765000 -- (-5857.184) [-5826.116] (-5856.197) (-5835.692) * [-5819.785] (-5870.005) (-5822.532) (-5852.558) -- 8:39:31      Average standard deviation of split frequencies: 0.007339      2766000 -- (-5852.138) [-5826.649] (-5830.965) (-5847.577) * (-5843.330) (-5841.547) [-5816.889] (-5853.469) -- 8:39:26      2767000 -- (-5852.850) (-5821.839) [-5811.493] (-5855.442) * (-5835.257) (-5834.415) [-5818.002] (-5859.887) -- 8:39:21      2768000 -- (-5845.677) [-5817.150] (-5815.140) (-5853.216) * (-5841.561) (-5837.190) [-5814.082] (-5865.344) -- 8:39:16      2769000 -- (-5850.113) [-5822.248] (-5824.723) (-5844.553) * (-5827.758) (-5844.952) [-5824.114] (-5852.051) -- 8:39:11      2770000 -- (-5859.573) (-5838.915) [-5820.940] (-5837.923) * [-5829.597] (-5836.748) (-5840.013) (-5860.848) -- 8:39:06      Average standard deviation of split frequencies: 0.007335      2771000 -- (-5853.187) (-5828.583) (-5825.799) [-5836.084] * [-5820.372] (-5831.897) (-5846.462) (-5883.897) -- 8:39:03      2772000 -- (-5842.968) (-5835.281) (-5823.237) [-5822.402] * [-5818.155] (-5835.502) (-5840.281) (-5861.834) -- 8:38:58      2773000 -- (-5850.090) (-5842.659) (-5814.781) [-5814.947] * (-5841.790) (-5823.097) [-5835.190] (-5847.269) -- 8:38:53      2774000 -- (-5844.652) (-5866.177) [-5807.894] (-5830.992) * (-5853.562) (-5839.753) (-5825.163) [-5827.375] -- 8:38:48      2775000 -- (-5844.004) (-5868.419) (-5829.799) [-5834.102] * [-5829.322] (-5861.223) (-5834.171) (-5853.217) -- 8:38:46      Average standard deviation of split frequencies: 0.007267      2776000 -- (-5836.432) (-5862.074) (-5849.366) [-5822.555] * (-5837.974) (-5843.675) [-5826.239] (-5852.222) -- 8:38:40      2777000 -- (-5828.513) (-5850.418) (-5847.241) [-5833.366] * (-5819.001) (-5845.442) [-5825.437] (-5873.482) -- 8:38:35      2778000 -- (-5828.761) (-5842.282) (-5853.467) [-5836.962] * [-5830.747] (-5867.330) (-5816.147) (-5856.018) -- 8:38:30      2779000 -- [-5825.809] (-5858.290) (-5850.029) (-5826.863) * (-5825.863) (-5878.290) [-5815.858] (-5833.619) -- 8:38:28      2780000 -- (-5861.720) (-5856.676) [-5820.063] (-5846.240) * [-5823.168] (-5864.932) (-5821.480) (-5861.092) -- 8:38:23      Average standard deviation of split frequencies: 0.007297      2781000 -- (-5846.559) (-5879.167) (-5835.048) [-5832.221] * (-5813.443) (-5862.785) [-5840.046] (-5844.470) -- 8:38:18      2782000 -- (-5838.301) (-5897.240) [-5830.382] (-5834.015) * [-5803.726] (-5862.043) (-5842.133) (-5843.288) -- 8:38:12      2783000 -- (-5846.176) (-5873.653) (-5845.115) [-5820.574] * [-5817.423] (-5846.416) (-5839.828) (-5867.415) -- 8:38:10      2784000 -- (-5844.985) (-5868.253) (-5816.785) [-5833.358] * [-5807.987] (-5844.062) (-5822.270) (-5839.735) -- 8:38:05      2785000 -- (-5853.814) (-5879.906) (-5821.236) [-5818.950] * [-5819.913] (-5846.400) (-5832.265) (-5842.837) -- 8:38:00      Average standard deviation of split frequencies: 0.007179      2786000 -- (-5859.951) (-5846.296) [-5823.713] (-5819.254) * (-5826.042) (-5852.914) (-5842.648) [-5830.573] -- 8:37:57      2787000 -- (-5865.117) (-5859.927) (-5827.217) [-5812.707] * (-5820.655) (-5867.922) [-5831.872] (-5829.353) -- 8:37:52      2788000 -- (-5870.299) (-5829.779) (-5843.691) [-5822.857] * [-5834.299] (-5864.382) (-5847.384) (-5842.081) -- 8:37:47      2789000 -- (-5841.565) (-5844.567) (-5837.455) [-5816.370] * (-5851.081) (-5850.991) (-5852.398) [-5835.989] -- 8:37:42      2790000 -- (-5853.789) (-5864.357) (-5829.737) [-5821.510] * [-5833.829] (-5838.729) (-5878.670) (-5844.579) -- 8:37:39      Average standard deviation of split frequencies: 0.007119      2791000 -- (-5844.081) (-5867.158) (-5831.766) [-5809.637] * [-5833.864] (-5834.690) (-5867.691) (-5836.324) -- 8:37:34      2792000 -- (-5839.348) (-5879.157) (-5830.499) [-5807.735] * [-5831.617] (-5842.136) (-5870.085) (-5846.663) -- 8:37:29      2793000 -- (-5837.882) (-5873.414) [-5841.383] (-5826.742) * [-5816.907] (-5845.753) (-5865.510) (-5847.923) -- 8:37:24      2794000 -- (-5842.287) (-5895.490) (-5847.053) [-5822.792] * [-5827.295] (-5829.597) (-5838.090) (-5851.801) -- 8:37:19      2795000 -- (-5844.286) (-5859.639) [-5828.177] (-5816.821) * (-5825.589) (-5859.594) [-5810.597] (-5862.682) -- 8:37:14      Average standard deviation of split frequencies: 0.007118      2796000 -- (-5840.407) (-5857.065) [-5828.148] (-5830.918) * (-5840.971) (-5858.520) [-5823.669] (-5867.057) -- 8:37:11      2797000 -- [-5831.785] (-5844.609) (-5833.943) (-5828.751) * (-5847.005) (-5834.774) [-5821.865] (-5847.756) -- 8:37:06      2798000 -- [-5839.275] (-5853.085) (-5846.964) (-5828.534) * (-5836.097) (-5841.236) [-5824.093] (-5836.723) -- 8:37:01      2799000 -- (-5848.873) (-5832.776) [-5834.964] (-5838.291) * (-5849.798) (-5837.198) [-5810.559] (-5826.101) -- 8:36:56      2800000 -- (-5853.022) [-5823.953] (-5836.956) (-5822.058) * (-5855.569) (-5846.668) [-5807.164] (-5853.830) -- 8:36:53      Average standard deviation of split frequencies: 0.006934      2801000 -- (-5859.268) [-5826.615] (-5847.995) (-5816.093) * (-5851.153) (-5825.076) [-5832.329] (-5854.296) -- 8:36:48      2802000 -- (-5855.792) [-5822.814] (-5834.746) (-5837.138) * [-5819.377] (-5829.870) (-5858.117) (-5835.623) -- 8:36:43      2803000 -- (-5870.137) (-5859.525) [-5815.784] (-5831.705) * (-5832.241) (-5828.107) (-5853.380) [-5831.449] -- 8:36:41      2804000 -- (-5862.576) (-5851.405) [-5824.193] (-5833.549) * (-5830.915) (-5835.616) (-5860.249) [-5821.603] -- 8:36:36      2805000 -- (-5850.046) (-5878.257) [-5830.263] (-5848.582) * (-5834.442) (-5848.112) (-5849.766) [-5819.305] -- 8:36:31      Average standard deviation of split frequencies: 0.006877      2806000 -- (-5843.451) (-5867.741) [-5812.907] (-5843.514) * (-5831.390) [-5841.141] (-5861.399) (-5823.475) -- 8:36:25      2807000 -- (-5841.569) (-5866.088) [-5816.250] (-5841.504) * (-5830.689) (-5834.884) (-5839.133) [-5825.473] -- 8:36:23      2808000 -- (-5850.711) (-5849.700) [-5817.921] (-5838.437) * (-5853.305) (-5824.821) (-5823.169) [-5819.058] -- 8:36:18      2809000 -- (-5833.960) (-5889.005) [-5802.338] (-5844.689) * (-5848.301) (-5836.041) (-5824.301) [-5818.985] -- 8:36:13      2810000 -- (-5842.968) (-5868.317) [-5822.106] (-5831.999) * (-5861.007) (-5844.404) (-5831.489) [-5820.641] -- 8:36:08      Average standard deviation of split frequencies: 0.006811      2811000 -- [-5845.803] (-5850.428) (-5837.042) (-5844.916) * (-5876.232) [-5820.188] (-5845.632) (-5835.846) -- 8:36:03      2812000 -- [-5820.319] (-5849.434) (-5820.860) (-5863.810) * (-5856.201) [-5821.013] (-5848.486) (-5819.770) -- 8:35:57      2813000 -- [-5822.914] (-5839.780) (-5821.122) (-5861.553) * (-5851.238) (-5815.732) (-5836.309) [-5827.904] -- 8:35:55      2814000 -- (-5833.223) (-5837.218) [-5829.035] (-5854.463) * (-5853.637) [-5835.463] (-5838.471) (-5849.591) -- 8:35:50      2815000 -- (-5843.210) (-5841.869) [-5836.978] (-5857.413) * (-5829.951) [-5824.219] (-5854.554) (-5862.309) -- 8:35:45      Average standard deviation of split frequencies: 0.006705      2816000 -- (-5835.745) (-5849.667) [-5848.748] (-5858.131) * (-5837.271) [-5821.193] (-5853.084) (-5879.080) -- 8:35:40      2817000 -- [-5834.008] (-5858.962) (-5838.439) (-5843.770) * [-5832.892] (-5830.703) (-5858.046) (-5848.700) -- 8:35:35      2818000 -- (-5851.000) (-5856.090) [-5823.285] (-5824.921) * (-5856.621) [-5824.400] (-5852.416) (-5859.940) -- 8:35:32      2819000 -- (-5844.163) (-5847.081) [-5817.492] (-5820.039) * (-5849.537) [-5820.432] (-5836.208) (-5879.673) -- 8:35:27      2820000 -- (-5843.424) (-5859.857) (-5815.519) [-5822.753] * (-5827.064) [-5826.566] (-5850.733) (-5868.805) -- 8:35:22      Average standard deviation of split frequencies: 0.006739      2821000 -- (-5827.157) (-5860.898) [-5812.308] (-5841.718) * (-5831.301) [-5835.822] (-5859.882) (-5861.281) -- 8:35:17      2822000 -- (-5835.266) (-5834.980) [-5816.120] (-5841.313) * (-5849.500) (-5829.812) (-5859.573) [-5824.605] -- 8:35:14      2823000 -- (-5833.193) (-5852.813) [-5817.413] (-5836.200) * (-5847.168) (-5859.061) (-5870.713) [-5832.889] -- 8:35:09      2824000 -- (-5854.402) (-5845.548) [-5816.978] (-5842.779) * (-5828.838) (-5862.772) (-5847.805) [-5824.384] -- 8:35:04      2825000 -- (-5851.490) (-5853.124) [-5832.712] (-5845.455) * (-5839.435) (-5869.730) [-5813.897] (-5823.447) -- 8:34:59      Average standard deviation of split frequencies: 0.006760      2826000 -- (-5846.559) (-5831.788) [-5820.773] (-5856.818) * (-5850.088) (-5850.481) [-5839.732] (-5839.698) -- 8:34:56      2827000 -- (-5843.620) (-5840.358) (-5841.901) [-5827.700] * (-5843.577) (-5855.425) [-5828.600] (-5827.770) -- 8:34:51      2828000 -- (-5839.799) (-5841.275) [-5834.550] (-5834.102) * (-5852.615) (-5835.809) (-5832.352) [-5836.372] -- 8:34:46      2829000 -- (-5850.123) (-5847.299) (-5837.516) [-5826.890] * (-5840.392) (-5836.979) [-5827.925] (-5840.393) -- 8:34:44      2830000 -- (-5854.392) (-5834.919) (-5826.646) [-5817.519] * [-5835.279] (-5851.072) (-5833.713) (-5823.354) -- 8:34:39      Average standard deviation of split frequencies: 0.006806      2831000 -- (-5864.051) (-5826.856) [-5816.841] (-5823.299) * (-5849.903) (-5845.537) [-5842.005] (-5825.362) -- 8:34:34      2832000 -- (-5842.623) (-5835.876) [-5832.759] (-5845.835) * (-5850.863) (-5847.049) (-5849.538) [-5835.755] -- 8:34:31      2833000 -- (-5839.476) (-5858.137) [-5817.155] (-5830.739) * (-5864.529) (-5854.289) (-5832.423) [-5821.112] -- 8:34:26      2834000 -- (-5849.154) (-5880.656) [-5810.418] (-5839.559) * (-5860.053) (-5842.952) (-5823.318) [-5836.230] -- 8:34:21      2835000 -- (-5837.963) (-5868.659) [-5812.526] (-5844.499) * (-5843.432) (-5837.110) (-5835.882) [-5829.594] -- 8:34:16      Average standard deviation of split frequencies: 0.006772      2836000 -- (-5873.206) (-5859.978) [-5822.675] (-5853.557) * (-5855.845) [-5828.867] (-5847.971) (-5843.189) -- 8:34:13      2837000 -- (-5860.000) (-5847.257) [-5820.938] (-5845.112) * (-5864.533) (-5817.525) (-5839.837) [-5822.005] -- 8:34:08      2838000 -- (-5859.433) (-5865.612) [-5806.688] (-5829.690) * (-5845.696) (-5832.844) (-5853.380) [-5822.480] -- 8:34:03      2839000 -- (-5864.179) (-5856.960) (-5827.589) [-5826.620] * (-5853.585) (-5842.325) [-5839.936] (-5843.669) -- 8:33:58      2840000 -- (-5857.628) (-5869.387) (-5837.305) [-5813.185] * (-5867.258) [-5812.337] (-5829.107) (-5840.881) -- 8:33:53      Average standard deviation of split frequencies: 0.006772      2841000 -- (-5844.073) (-5876.415) (-5827.759) [-5826.392] * (-5849.109) (-5819.498) (-5839.513) [-5815.456] -- 8:33:50      2842000 -- (-5850.302) (-5851.614) [-5831.982] (-5829.805) * (-5831.682) [-5848.363] (-5843.451) (-5826.995) -- 8:33:45      2843000 -- (-5850.531) (-5867.914) (-5823.307) [-5821.816] * (-5841.662) (-5832.651) (-5853.444) [-5839.525] -- 8:33:40      2844000 -- [-5817.167] (-5849.178) (-5835.422) (-5829.735) * [-5824.996] (-5824.140) (-5853.739) (-5841.864) -- 8:33:35      2845000 -- [-5826.463] (-5844.264) (-5836.279) (-5865.971) * (-5836.129) [-5824.512] (-5841.933) (-5841.069) -- 8:33:33      Average standard deviation of split frequencies: 0.006747      2846000 -- [-5820.902] (-5856.307) (-5841.521) (-5851.486) *
[truncated: 1,063,181 more chars]
