# Supplementary figures and images for: A review of Coelostegus prothales Carroll and Baird, 1972 from the Upper Carboniferous of the Czech Republic and the interrelationships of basal eureptiles
Source: PLoS One. 2023 Sep 21;18(9):e0291687. doi: 10.1371/journal.pone.0291687 (PMC10513281; doi:10.1371/journal.pone.0291687)

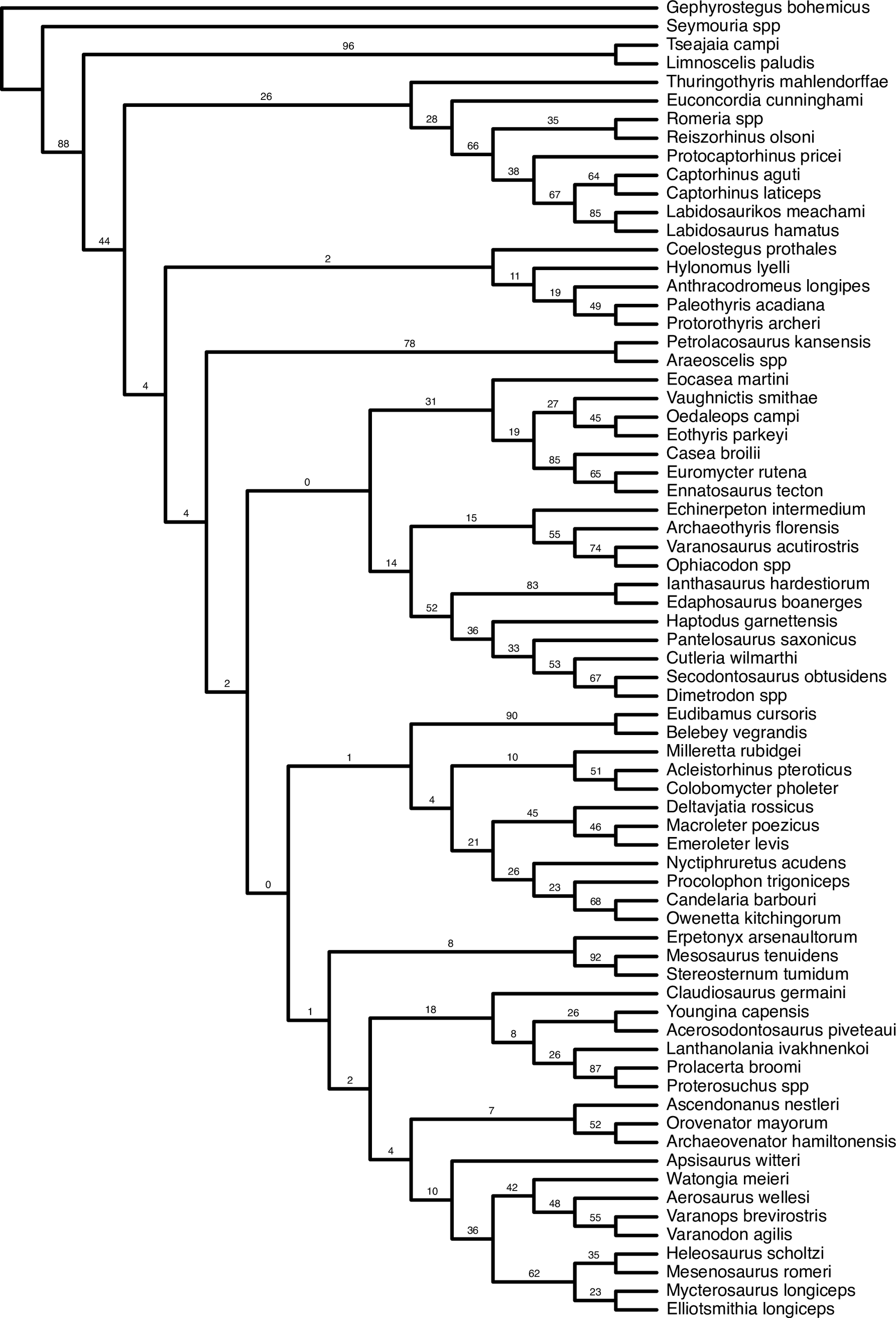

Supplement: S1 Fig — Bootstrap 50% majority-rule consensus from Ford and Benson’s [7] dataset, with bootstrap percentage support appended to branches. (TIF) [file pone.0291687.s005.tif]

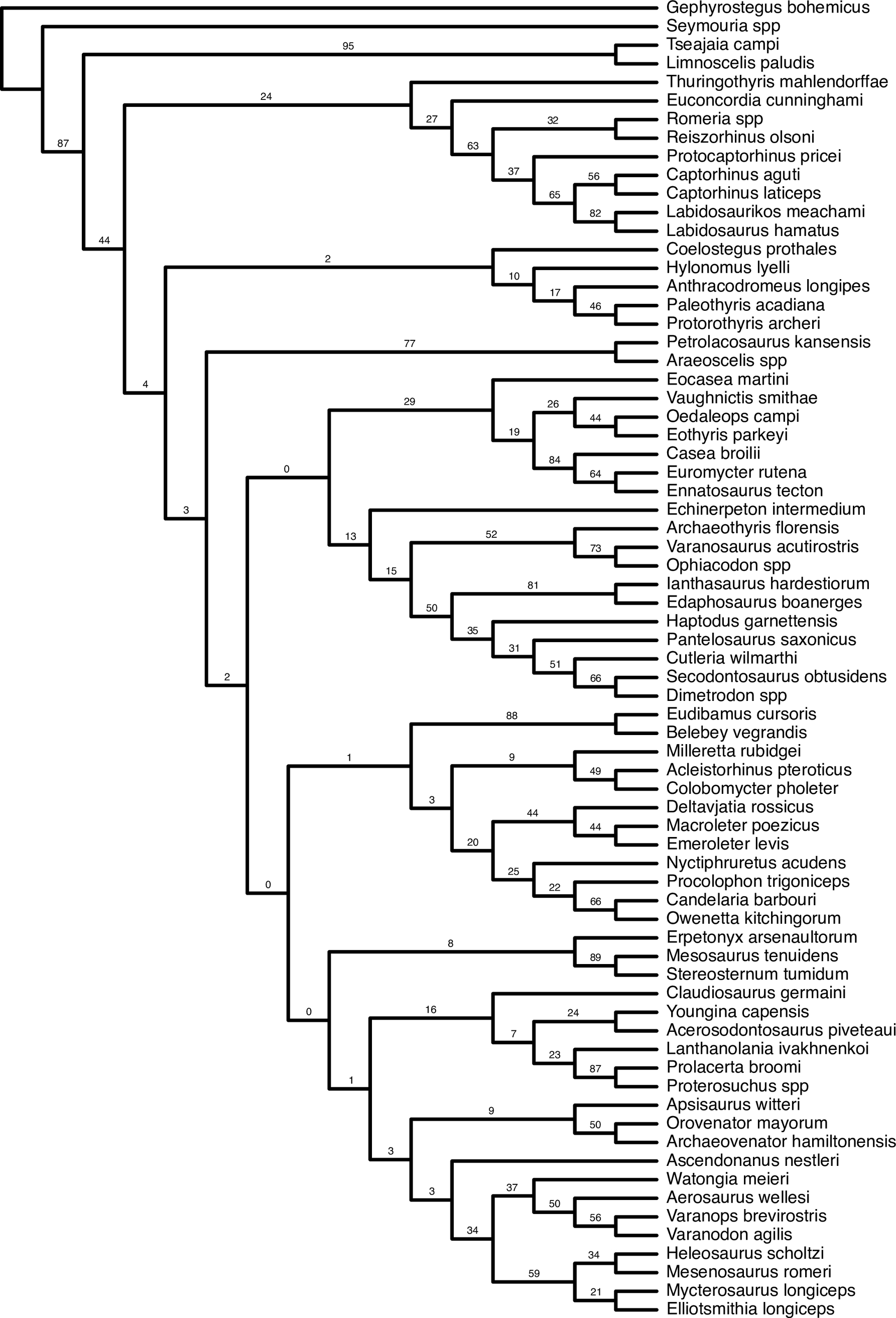

Supplement: S2 Fig — Jackknife 50% majority-rule consensus from Ford and Benson’s [7] dataset, with jackknife percentage support appended to branches. (TIF) [file pone.0291687.s006.tif]

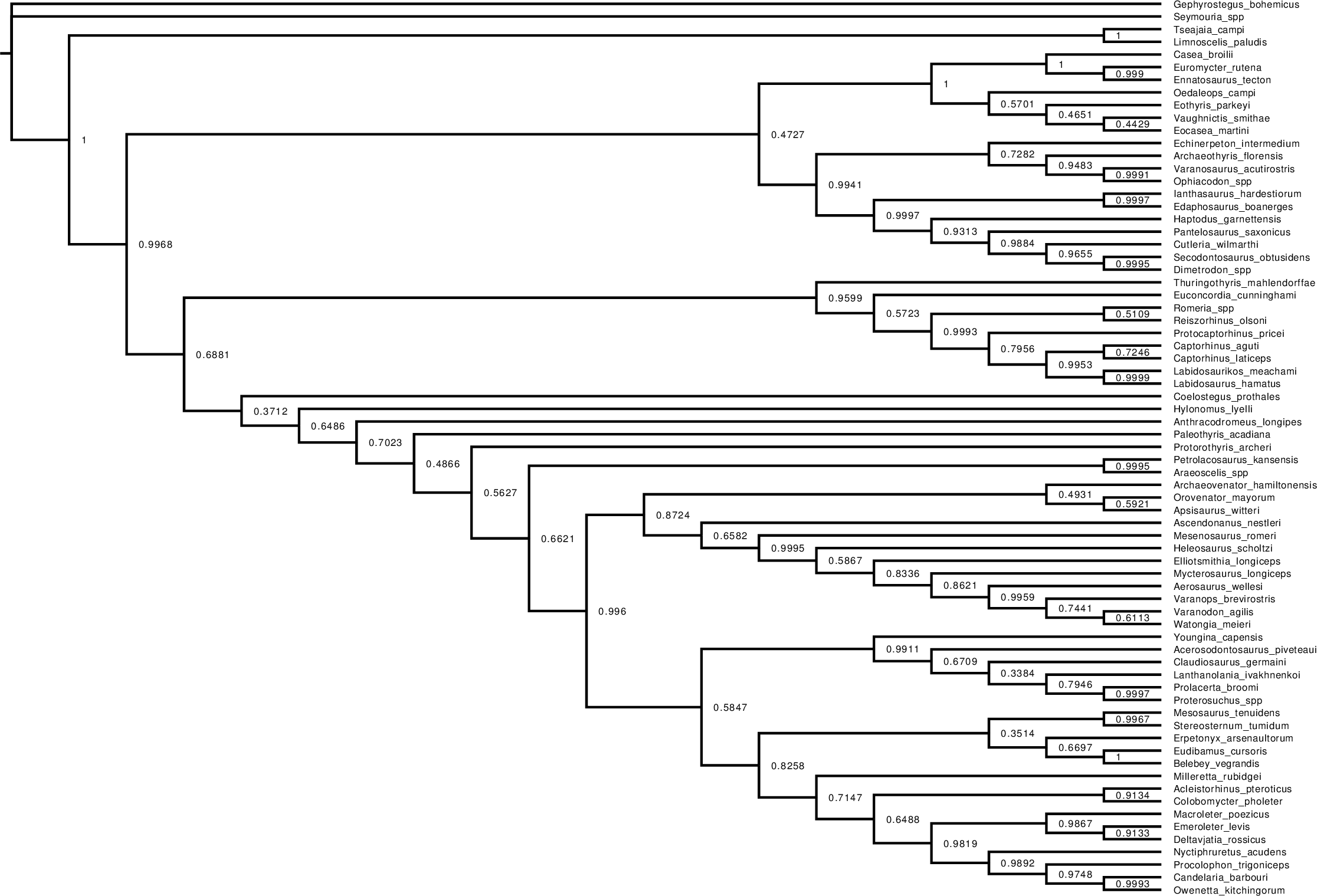

Supplement: S3 Fig — Bayesian 50% majority-rule consensus from Ford and Benson’s [7] dataset, including groups compatible with consensus, and with Bayesian posterior probabilities appended to branches. (TIF) [file pone.0291687.s007.tif]

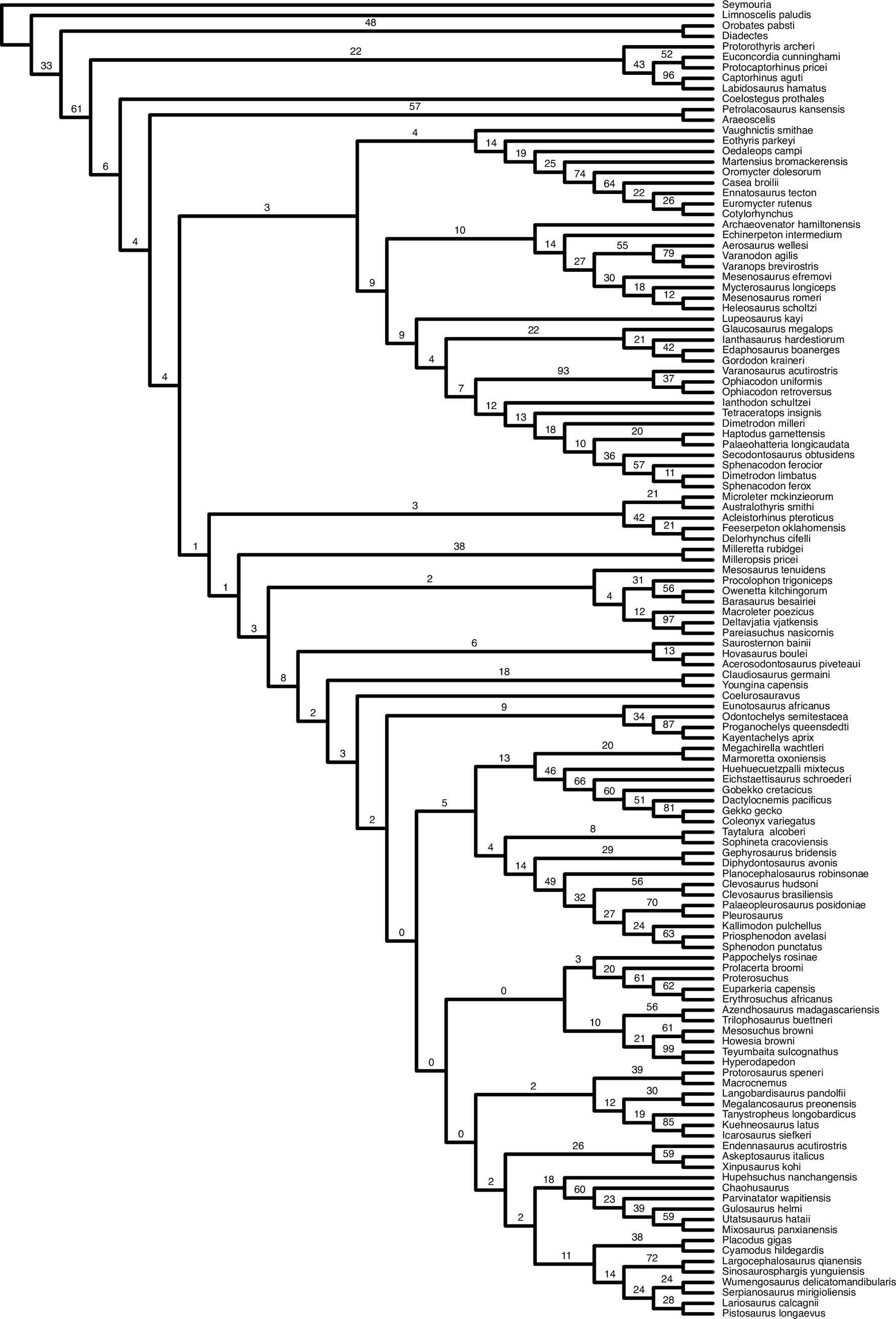

Supplement: S4 Fig — Bootstrap 50% majority-rule consensus from Simoes et al.’s [8] dataset, with bootstrap percentage support appended to branches. (TIF) [file pone.0291687.s008.tif]

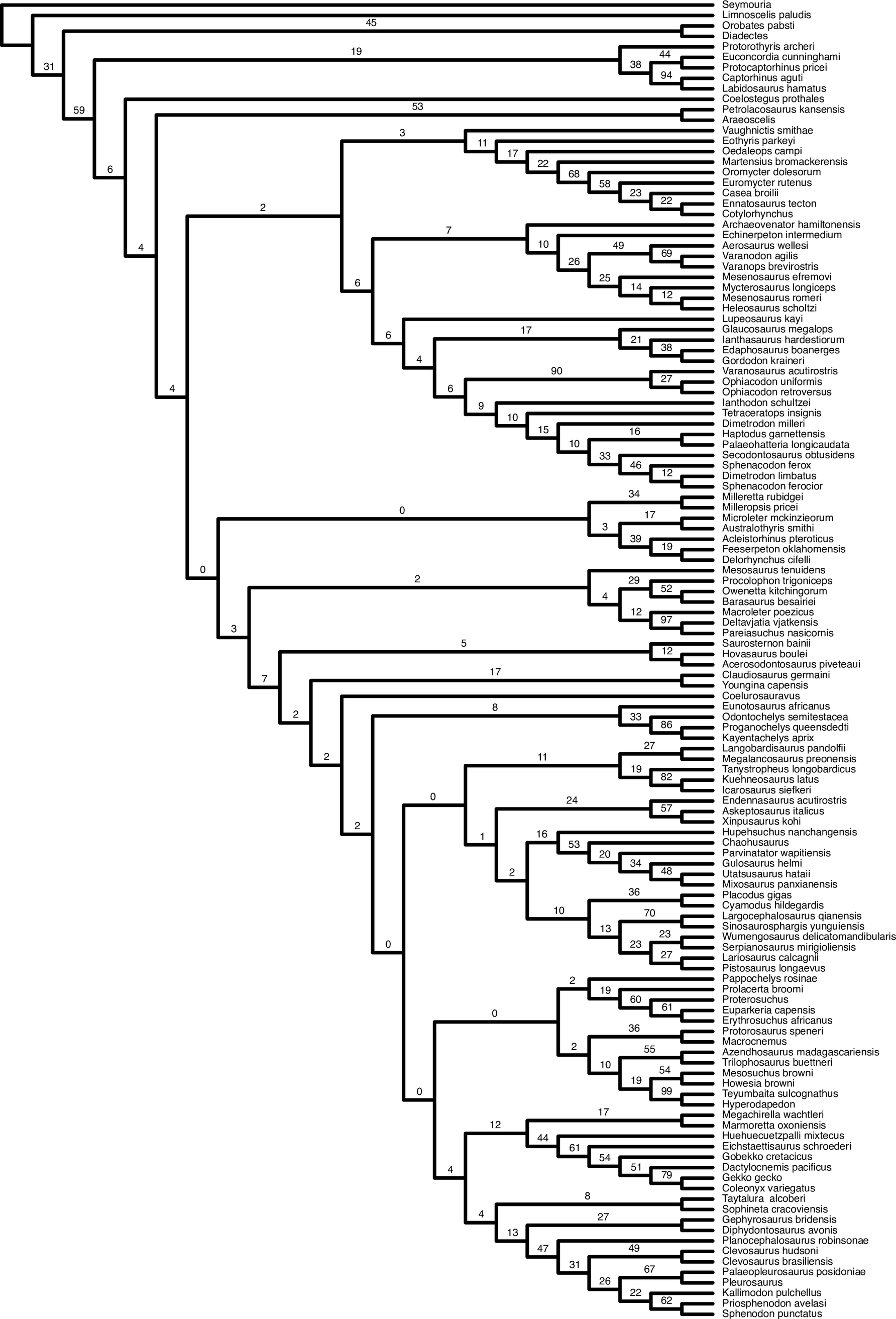

Supplement: S5 Fig — Jackknife 50% majority-rule consensus from Simoes et al.’s [8] dataset, with jackknife percentage support appended to branches. (TIF) [file pone.0291687.s009.tif]

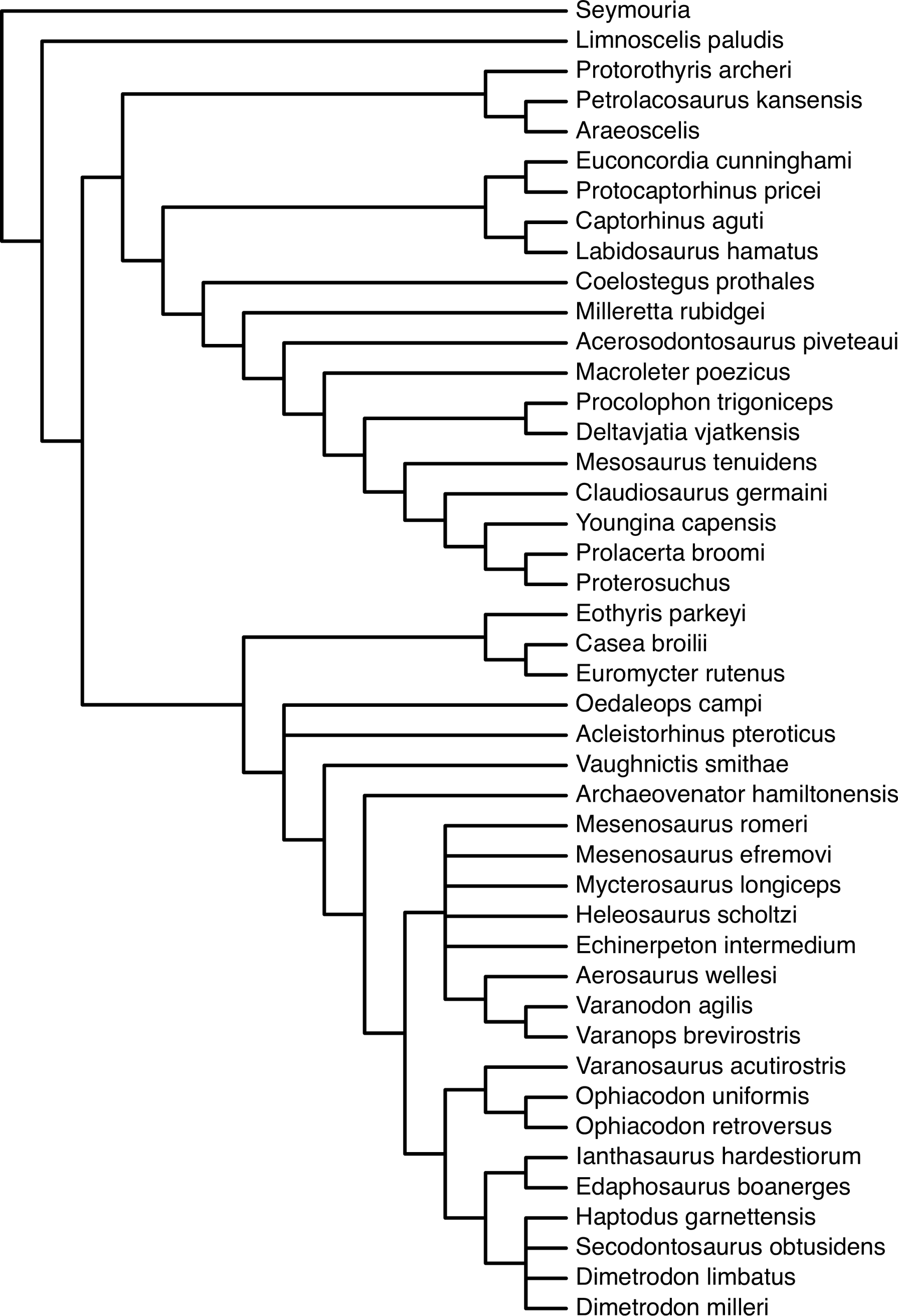

Supplement: S6 Fig — A. Strict consensus of the 206 shortest trees from a maximum parsimony analysis of the taxonomically pruned version of Ford and Benson’s [7] dataset, using only taxa in common with the Simoes et al.’s [8] dataset, and with all characters having equal unit weight. B. Strict consensus of the 27 shortest trees from a maximum parsimony analysis of the taxonomically pruned version of the Simoes et al.’s [8] dataset, using only taxa in common with the Ford and Benson’s [7] dataset, and with all characters having equal unit weight. (ZIP) [file pone.0291687.s010.zip › S6B Fig.tif]

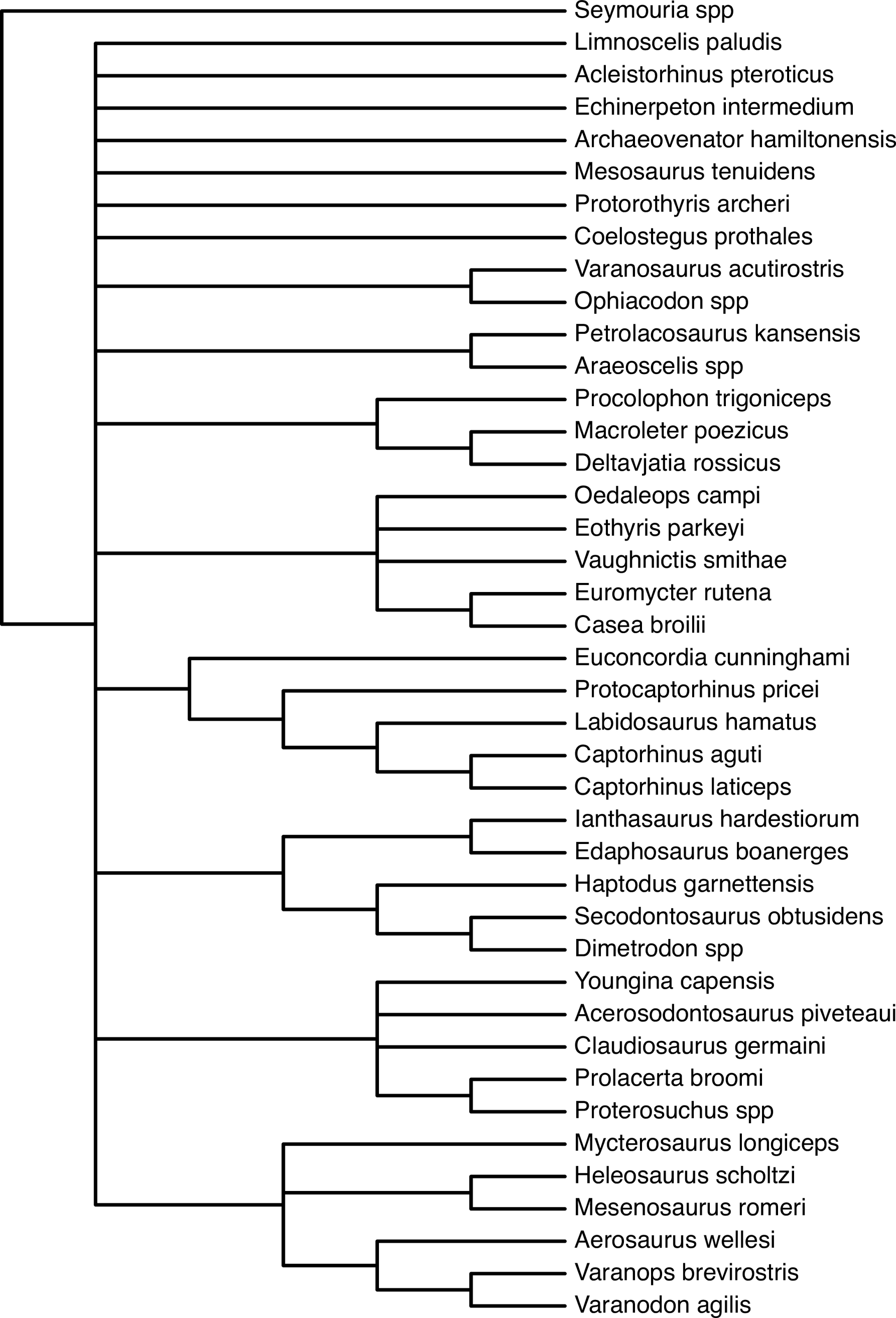

Supplement: S6 Fig — A. Strict consensus of the 206 shortest trees from a maximum parsimony analysis of the taxonomically pruned version of Ford and Benson’s [7] dataset, using only taxa in common with the Simoes et al.’s [8] dataset, and with all characters having equal unit weight. B. Strict consensus of the 27 shortest trees from a maximum parsimony analysis of the taxonomically pruned version of the Simoes et al.’s [8] dataset, using only taxa in common with the Ford and Benson’s [7] dataset, and with all characters having equal unit weight. (ZIP) [file pone.0291687.s010.zip › S6A Fig.tif]
